# Revisiting the Baddeley Reaction: Access to Functionalized Decalins by Charge‐Promoted Alkane Functionalization

**DOI:** 10.1002/anie.202418067

**Published:** 2024-11-20

**Authors:** Miloš Vavrík, Phillip S. Grant, Daniel Kaiser, Tim Gruene, Nuno Maulide

**Affiliations:** ^1^ Institute of Organic Chemistry University of Vienna Währinger Straße 38 1090 Vienna Austria; ^2^ Core Facility for Crystal Structure Analysis University of Vienna Währinger Straße 42 1090 Vienna Austria

**Keywords:** Baddeley reaction, decalin, alkane functionalization, acylium ion, octalin

## Abstract

C−H functionalization of purely aliphatic substrates is a challenging endeavor, as the absence of directing groups generally thwarts attempts at regiocontrol. This is particularly true for difunctionalization reactions, where the control of relative stereochemistry poses an additional obstacle. The Baddeley reaction of decalins, despite suffering from strong limitations with regard to yield and generality, stands as one of only few known transformations capable of regio‐ and stereocontrol in aliphatic C−H functionalization. Herein, we report a regio‐ and diastereoselective method for the double functionalization of decalins enabling access to a novel, unreported regioisomer in synthetically useful yields. This method was also successfully applied to a range of other alkane substrates, enabling a straightforward synthesis of keto alcohols from the simplest alkane building blocks.

## Graphical abstract

We report a new strategy for the C−H difunctionalization of purely aliphatic alkane substrates. The reaction proceeds with complete regio‐ and diastereoselectivity and enables access to a wide range of previously inaccessible acyl 9‐decalinols. Additionally, the application of this method translated well to simple monocyclic alkane substrates.

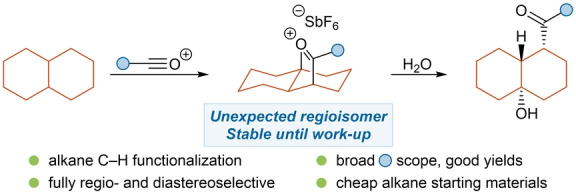

The decalin system (bicyclo[4.4.0]decane) is prevalent in numerous natural products, encompassing isoprenoids, such as sesquiterpenoids and diterpenoids, as well as polyketides (Figure 1a).[^1^, ^2^] The high complexity of most bioactive compounds containing a decalin core translates into a vast structural and functional diversity, which has inspired considerable efforts into the exploration of chemical synthesis and therapeutic potential.[^3^, ^4^]

**Figure 1:**
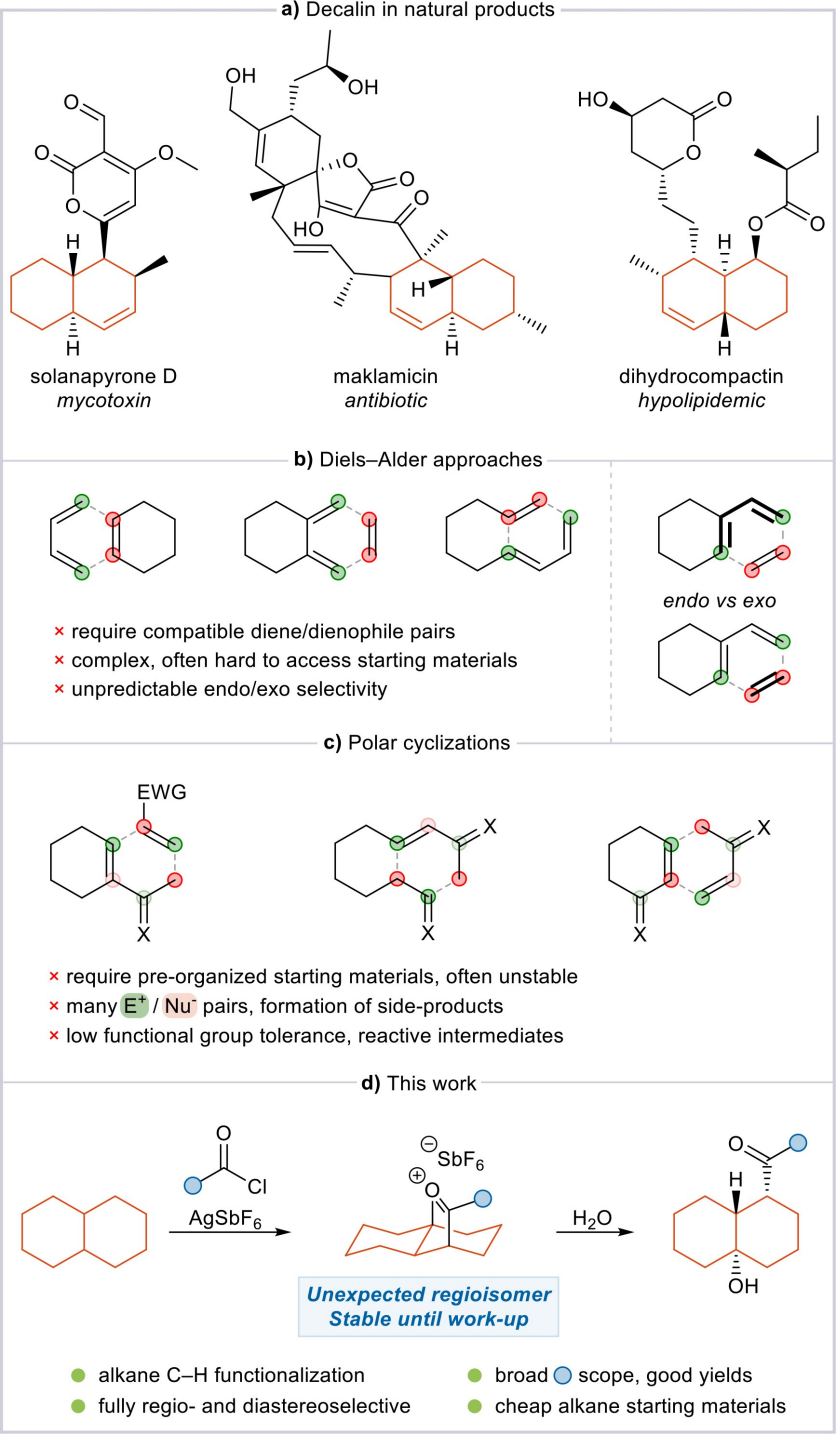
**a**: Selected examples of natural compounds containing a decalin core. **b/c**: Examples of fragments used in Diels–Alder reactions and domino Michael/aldol additions for the construction of substituted decalins. **d**: This work: Development of an improved method for C−H difunctionalization of unfunctionalized decalin.

Indeed, the literature is rich with syntheses of decalin‐containing fragments or products.[^5^, ^6^, ^7^, ^8^, ^9^] Interestingly, however, the high complexity of these molecules means that no unified strategy has emerged but rather a vast array of approaches exists, each enabling the synthesis of a different substitution pattern. Diels–Alder reactions (Figure 1b) are perhaps the most commonly featured method (e.g., in the syntheses of solanapyrone D or maklamicin),[^5^, ^6^] while domino Michael additions have also been described (Figure 1c), such as in the synthesis of dihydrocompactin.[^7^, ^9^, ^10^] Despite their efficiency, these methods notoriously mandate complex starting materials featuring pre‐organized, carefully designed diene/dienophile or nucleophile/Michael acceptor arrays, the synthesis of which often requires intricate multistep procedures. Additionally, unpredictable *endo*/*exo* selectivity (in the Diels–Alder approaches),^[11]^ or low functional group tolerance (in the domino Michael protocols) highlight the advantages that could result from the development of new methods relying on simple and unfunctionalized starting materials.

An example of such a method is the Baddeley reaction, a rare type of C−H functionalization of a fully unsubstituted alkane. Unfortunately, poor reaction yields, low selectivity and limited substrate scope have imposed major limitations to the practicability of this reaction, as evidenced by a singular report mentioning its use in the synthesis of more complex molecules.^[12]^ Thus, alternative reactivity profiles that might eventually offer access to complex decalins remain in high demand.

Herein, we report a first step in this direction, describing our investigations in this area and the discovery of an adapted protocol, which provides an unprecedented product regioisomer in high yields and with full diastereocontrol (Figure 1d).

We were initially intrigued by the formal oxidative functionalization of unsubstituted decalin reported by Baddeley and co‐workers,[^13^, ^14^, ^15^, ^16^, ^17^, ^18^] reminiscent of the second phase of the “two‐phase” (cyclase phase, followed by oxidase phase) strategy conceptualized by Baran decades later.^[19]^ In the 1959 report, Baddeley observed that treatment of decalin with acetyl chloride and aluminium chloride, “à la” Friedel–Crafts, results in the formation of a complex mixture of products (Scheme 1a, conditions 1, see Supporting Information Section 1 for a detailed discussion).^[13]^

**Scheme 1:**
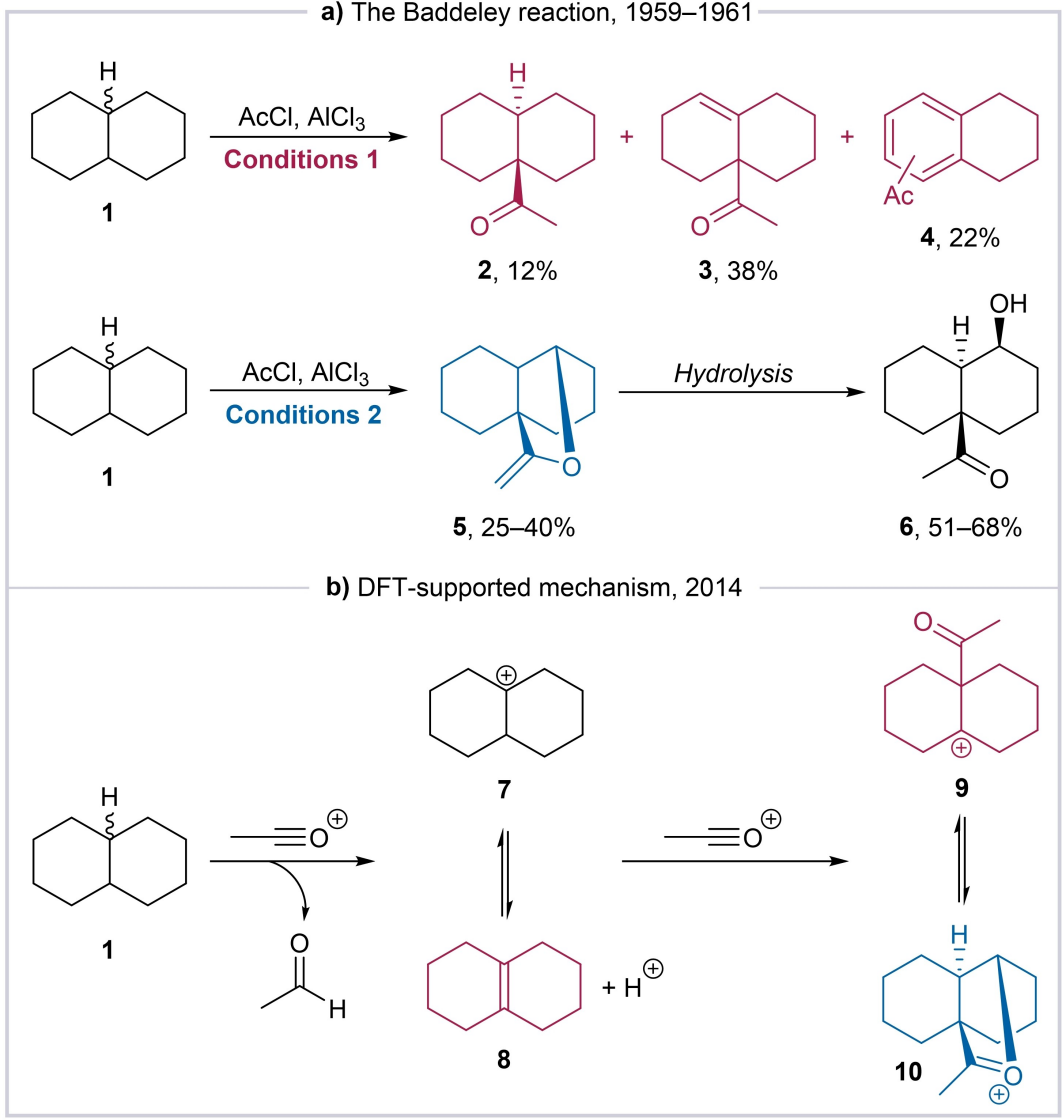
**a**: Two different outcomes of the Baddeley reaction, depending on the reaction conditions. **b**: The proposed mechanism of the Baddeley reaction.^[20]^ Conditions 1: decalin (3.6 mol), AcCl (1.1 equiv.), AlCl_3_ (1.8 equiv.), DCM (5.1 M), room temperature, 2 days. Conditions 2: decalin (5.0 mol), AcCl (2.4 equiv.), AlCl_3_ (1.5 equiv.), DCE (5.3 M), 10 °C, 5 h.

A similar set of conditions, under which the reaction afforded a single product, the vinyl ether **5**, was described later (Scheme 1a, conditions 2).^[14]^ A detailed, mostly computational mechanistic investigation by Lewis in 2014^[20]^ revealed that the reaction involves two key steps: a) a hydride abstraction event and b) a nucleophilic attack by the in situ generated octalin **8** on the acylium species (Scheme 1b).

However promising, this transformation has only found scarce application beyond these reports,[^12^, ^18^, ^21^, ^22^, ^23^, ^24^] presumably due to the low reaction yield and selectivity (see SI, Section 1). Furthermore, product isolation was reported with only three aliphatic acyl chlorides (see SI, Scheme S3).

A pivotal aspect of the Baddeley reaction involves the generation of acylium ions. We speculated that the shortcomings of the processes shown in Scheme 1 might be remedied by deploying two key ideas, *viz*.:

1. **The use of non‐basic counterions**. We hypothesized that the presence of basic species (such as AlCl_4_^−^) could have a deleterious effect on reaction performance;
2. **Switching to a method which does not require AlCl_3_ to generate acylium ions**. The presence of aluminium(III) chloride was correlated with unavoidable product‐isomerization reactions.^[25]^ As a result, product distribution, as shown in Scheme 1a, is strongly dependent on the reaction conditions and stoichiometry (see SI, Schemes S1 and S3).

With these ideas in mind, we envisioned the use of silver salts for acylium generation, as this would simultaneously omit the need for AlCl_3_ and allow the use of counterions with significantly reduced basicity.^[26]^ Following this strategy, we screened multiple sets of reaction conditions (see Supporting Information for a full optimization table) and found that, by treating decalin with *p‐*toluoyl chloride and AgSbF_6_, keto alcohol **12** was formed (Scheme 2), albeit in a moderate 36 % yield.

**Scheme 2:**
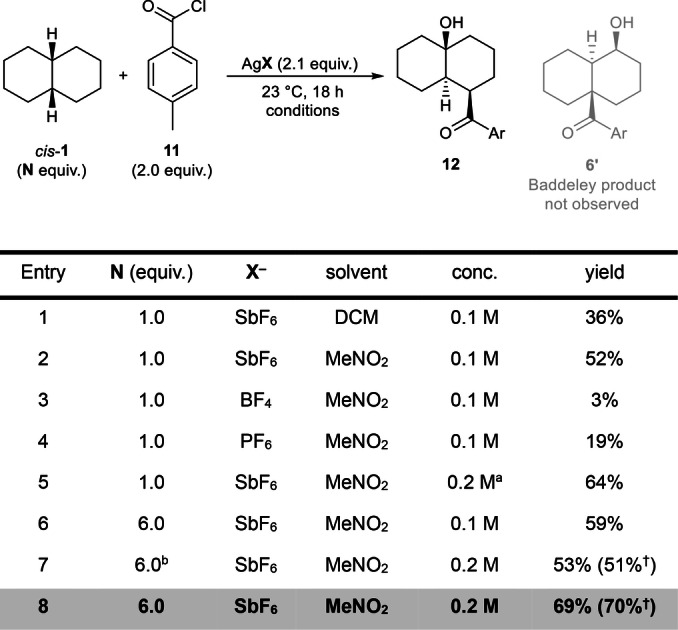
Conditions: decalin (1.0 equiv.=0.1 mmol, *cis* unless otherwise specified), *p*‐toluoyl chloride (0.2 mmol), AgX (0.21 mmol), solvent (0.5–2.0 mL), 23 °C, 18 h. Yields of **12** were determined by ^1^H NMR analysis of the crude reaction mixtures using mesitylene as internal standard (for details on yield calculation, see SI, Section 2). ^a^0.05 M concentration gave a lower yield of 31 %. ^b^Technical mixture of isomers, *cis*:*trans*=1.2 : 1. ^†^Isolated yield. For a full optimization table see the Supporting Information (Table S1).

Intriguingly, this unprecedented product had inverted regiochemistry with respect to the position of the substituents (OH and acyl) compared to the “classical” Baddeley reaction product (**12** vs. **6’**, Scheme 2).

Furthermore, we confirmed that the new product **12** was *not* formed when the reaction was conducted using the Baddeley reaction conditions (see SI, Scheme S4). This peculiar observation formed the basis for further investigations (Scheme 2). In these studies, we observed that the solvent plays a crucial role, with anhydrous nitromethane being unrivalled by all other solvents in terms of reaction yield and purity of the crude products (Entries 1–2, see Supporting Information for a full survey of solvents). AgBF_4_ and AgPF_6_ were found to be inferior to AgSbF_6_ (Entries 3–4)—likely a consequence of the more basic (or nucleophilic) nature of those counterions and of the increased stability of acylium hexafluoroantimonates.^[27]^

Concentration was found to also play a major role, as the yield dropped with high dilutions and reached a maximum at 0.2 m (Entry 5). Using an excess of decalin had a slightly beneficial impact on yield (Entry 6); in addition to the increased yield, we consider this stoichiometry favorable due to the good availability of decalin, as well as its ease‐of‐handling and low price compared to the other reactants. *cis*‐Decalin was found to be superior to *trans*‐decalin;^[28]^ however, using a technical isomeric mixture (*cis*:*trans*=1.2 : 1) still afforded the product in a satisfactory 51 % yield (Entry 7).

We then applied the optimized conditions (Entry 8) to a wider array of acyl chlorides (Scheme 3).

**Scheme 3:**
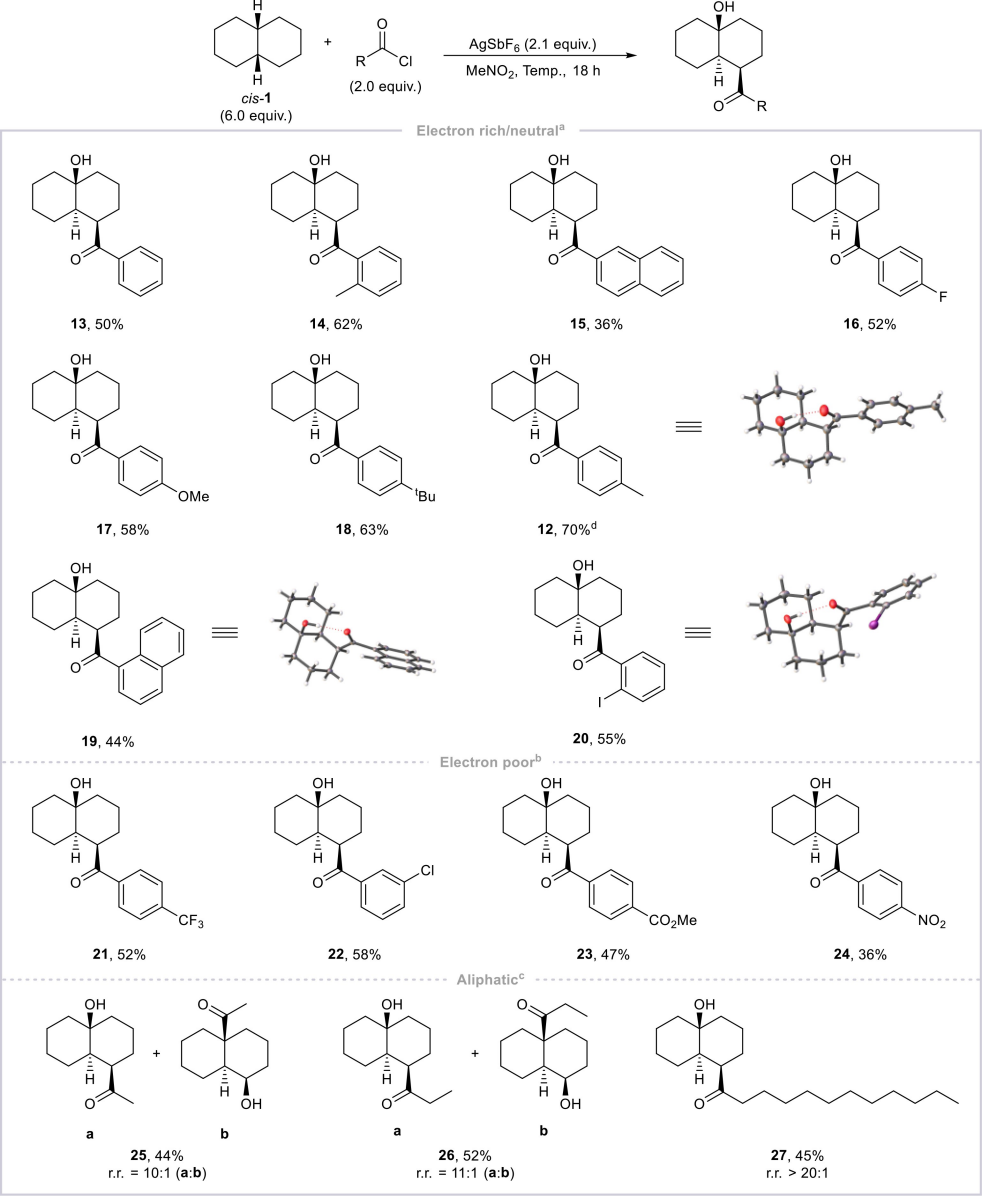
Scope of acyl chlorides. Conditions: ^a^electron‐rich/neutral: *cis*‐decalin (0.6 mmol), acyl chloride (0.2 mmol), AgSbF_6_ (0.21 mmol) in MeNO_2_ (0.5 mL), 23 °C, 18 h. ^b^electron‐poor: *cis*‐decalin (0.6 mmol), acyl chloride (0.2 mmol), AgSbF_6_ (0.21 mmol) in MeNO_2_ (0.5 mL), −20 to 23 °C, 18 h. ^c^aliphatic: *cis*‐decalin (0.1 mmol), acyl chloride (0.4 mmol), AgSbF_6_ (0.42 mmol) in MeNO_2_ (0.5 mL), 55 °C, 18 h. ^d^Scale‐up preparation (0.5 g=3.2 mmol acyl chloride) of product **12** proceeded with an identical yield of 70 %. r.r.=regioisomeric ratio, determined by ^1^H NMR analysis of the crude reaction mixture using mesitylene as internal standard. The yields reported correspond to pure, isolated product. For details on yield calculation, see Supporting Information (Section 2).

The reaction is compatible with aromatic acyl chlorides bearing both electron‐rich and electron‐deficient substituents and affords the products in good yields without requiring any changes in reaction stoichiometry.^[29]^ Crystallization of three keto alcohols (**12**, **19** and **20**) provided unambiguous proof of structural assignment and revealed a hydrogen bond between the hydroxy group and the carbonyl functionality (Scheme 3).^[30]^ An interesting observation was made with acetyl chloride, which afforded a regioisomeric mixture of keto alcohols at 23 °C (**25 a** and **25 b**, r.r.=2 : 1 at 23 °C, Scheme 3). Pleasingly, increasing the reaction temperature to 55 °C led to a significant increase in selectivity for formation of the unprecedented isomer **25 a** (r.r.=10 : 1). This modification translated well to other aliphatic acyl chlorides (**26** and **27**).

We were eager to better understand the mechanism of this transformation, as the product structure suggests a significant mechanistic divergence from the Baddeley reaction. First, we performed NMR experiments which showed oxocarbenium species *trans*‐**28** as the only decalin‐containing product formed during the reaction (Scheme 4a).

**Scheme 4:**
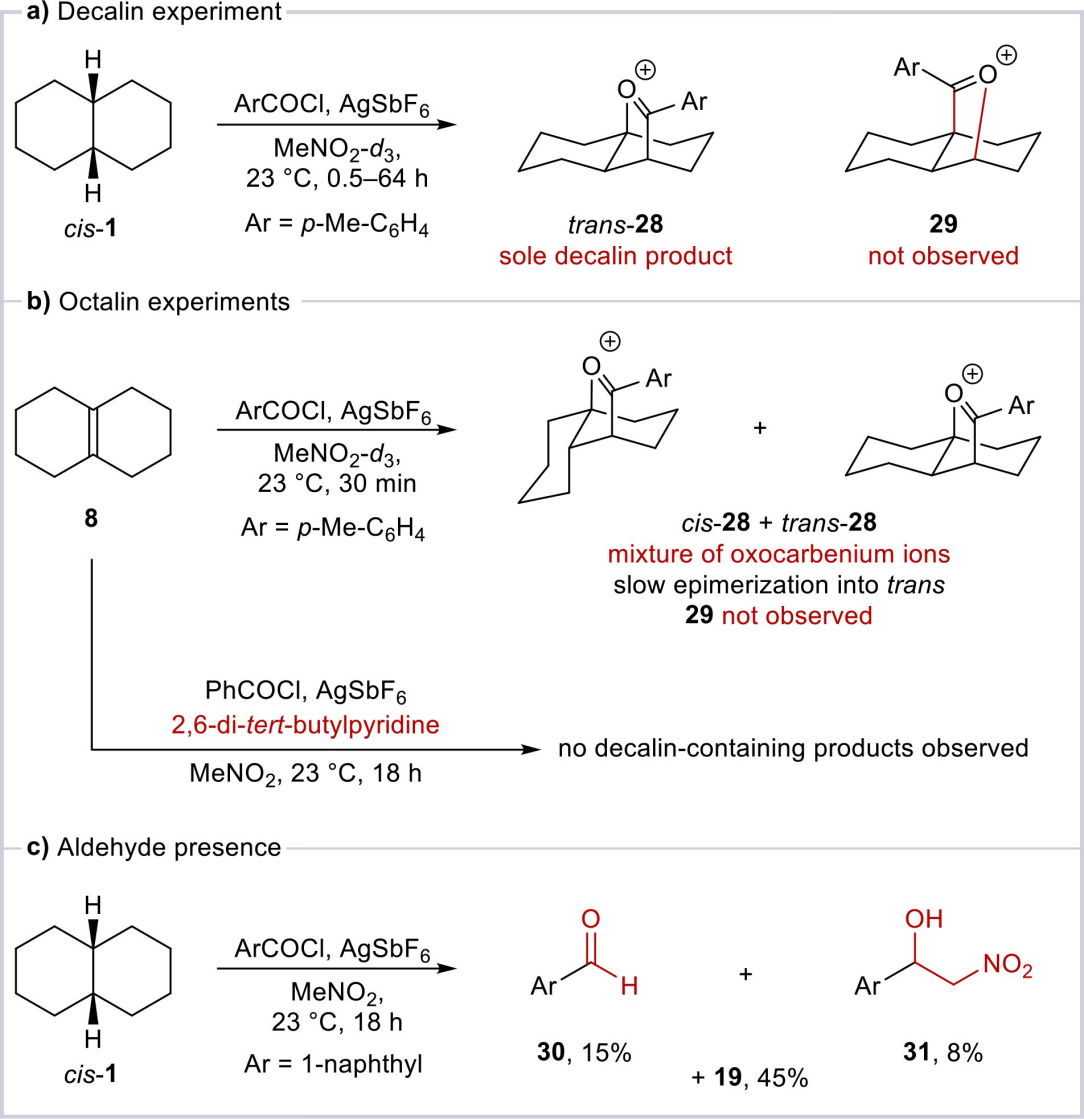
Mechanistic experiments provide insight into the intermediates formed during the reaction. For experimental details see Supporting Information (Section 4.3).

A similar NMR experiment, albeit with Δ^9,10^‐octalin (**8**)—a putative intermediate of the Baddeley reaction^[20]^—as the substrate revealed the formation of a mixture of oxocarbenium ions *cis*‐**28** and *trans*‐**28**, which slowly equilibrated to *trans*‐**28** (Scheme 4b). Notably, despite being known to afford the Baddeley regioisomer **29** under previously reported conditions, octalin **8** formed only *cis*‐**28** and *trans‐* **28** under our reaction conditions. Another experiment with octalin **8** revealed that, in the presence of a non‐nucleophilic base, no reaction occurred.

When naphthoyl chloride was employed, the anticipated aldehyde byproduct **30**—the genesis of which will be discussed in Scheme 5—was observed, together with **31**, the product of Henry addition, arising from reaction of **30** with MeNO_2_ (Scheme 4c).

**Scheme 5:**
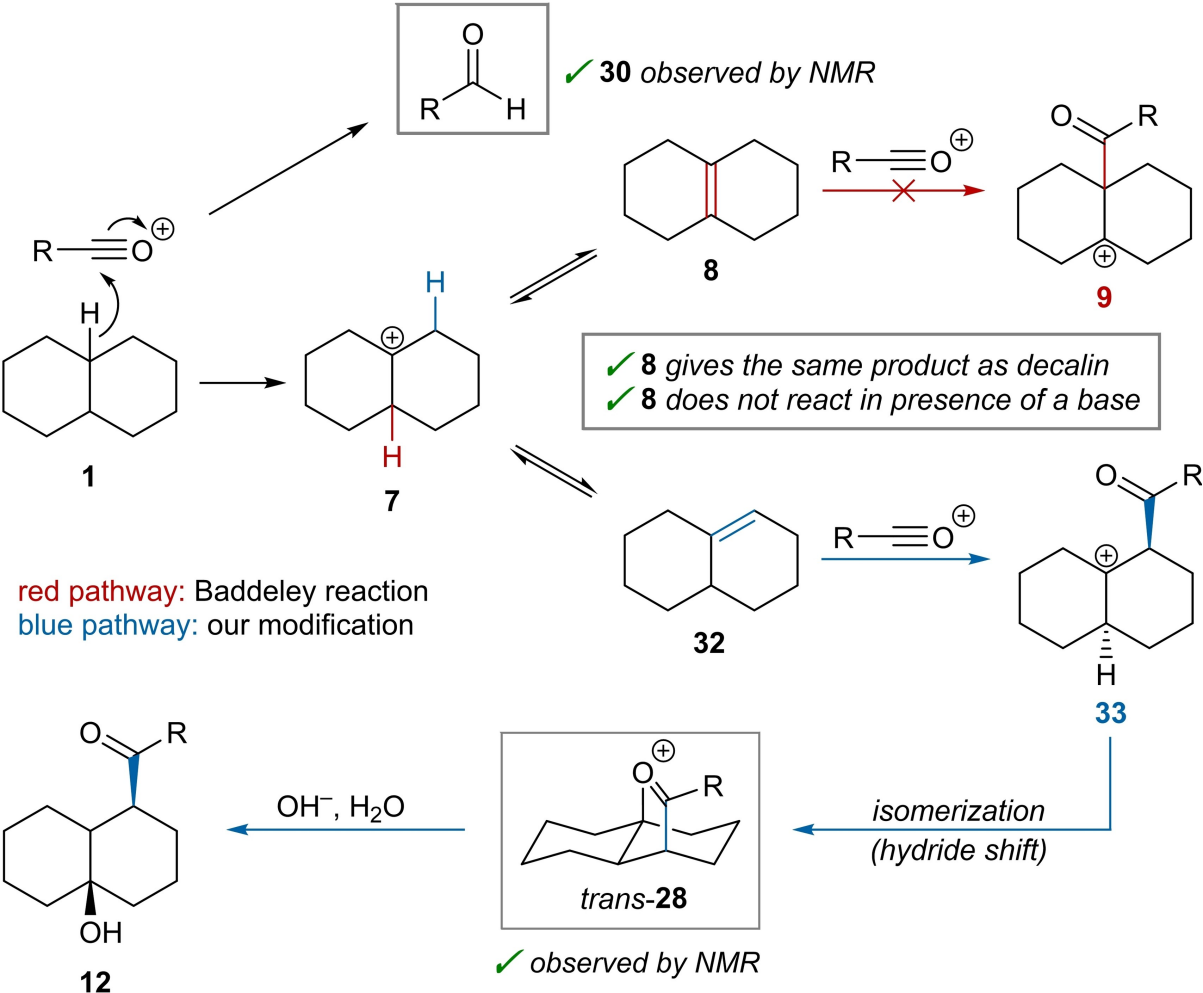
Proposed reaction mechanism involving hydride abstraction to form decalyl cation **7**, followed by deprotonation, acylation, isomerization, and a terminating capture of the resulting cation as oxocarbenium species *trans‐* **28**.

These observations lead us to propose the following reaction mechanism (Scheme 5): Initial hydride abstraction from decalin results in decalyl cation **7** and an aldehyde (such as **30**, see Scheme 4c) as a by‐product. This is followed by proton elimination, capable of affording two different octalin isomers: **8**, bearing a tetrasubstituted alkene, and **32**, bearing a trisubstituted alkene. However, the formation of this olefin mixture is inconsequential under our conditions, as the double bond can isomerize and Δ^1,9^‐octalin (**32**) attacks the acylium ion preferentially, as shown by the first octalin experiment.^[31]^ In the presence of a base, double bond isomerization between **8** and **32** is thwarted and hence no reaction occurs (Scheme 4b; see SI, Section 3 for more details). Following nucleophilic attack of **32** onto the acylium ion (see SI, Section 3 for a discussion regarding possible reversibility of this step), acyl‐substituted cation **33** is formed, subsequently undergoing isomerization (through a hydride shift), and capture of the carbocation by the carbonyl group to yield oxocarbenium ion *trans*‐**28**, the final product preceding aqueous workup (observed by NMR, Scheme 4a). This outcome, also observed with acylium ions bearing aliphatic substituents with α‐protons, stands in contrast to the classical Baddeley reaction, in which a subsequent deprotonation event occurs, and a vinyl ether is formed (see SI, Section 3 for more details).^[32]^

Seeking to expand the scope of this reaction beyond decalin, we applied the developed conditions to an array of other alkane substrates (Scheme 6a; see SI, Section 4.7, page S90 for the full scope of successful and unsuccessful alkane substrates). Notably, several simple alkanes, such as methylcyclohexane and dimethylcyclohexanes, afforded the corresponding keto alcohol products **35**, **37** and **39** in moderate to good yields (Scheme 6a). For disubstituted cyclohexanes the reaction generated only two diastereomers, differing in the orientation of the methyl group at C2. The structure of product **39** indicates the occurrence of an additional hydride shift, likely driven by the increased stability of the resulting tertiary carbocation.

**Scheme 6:**
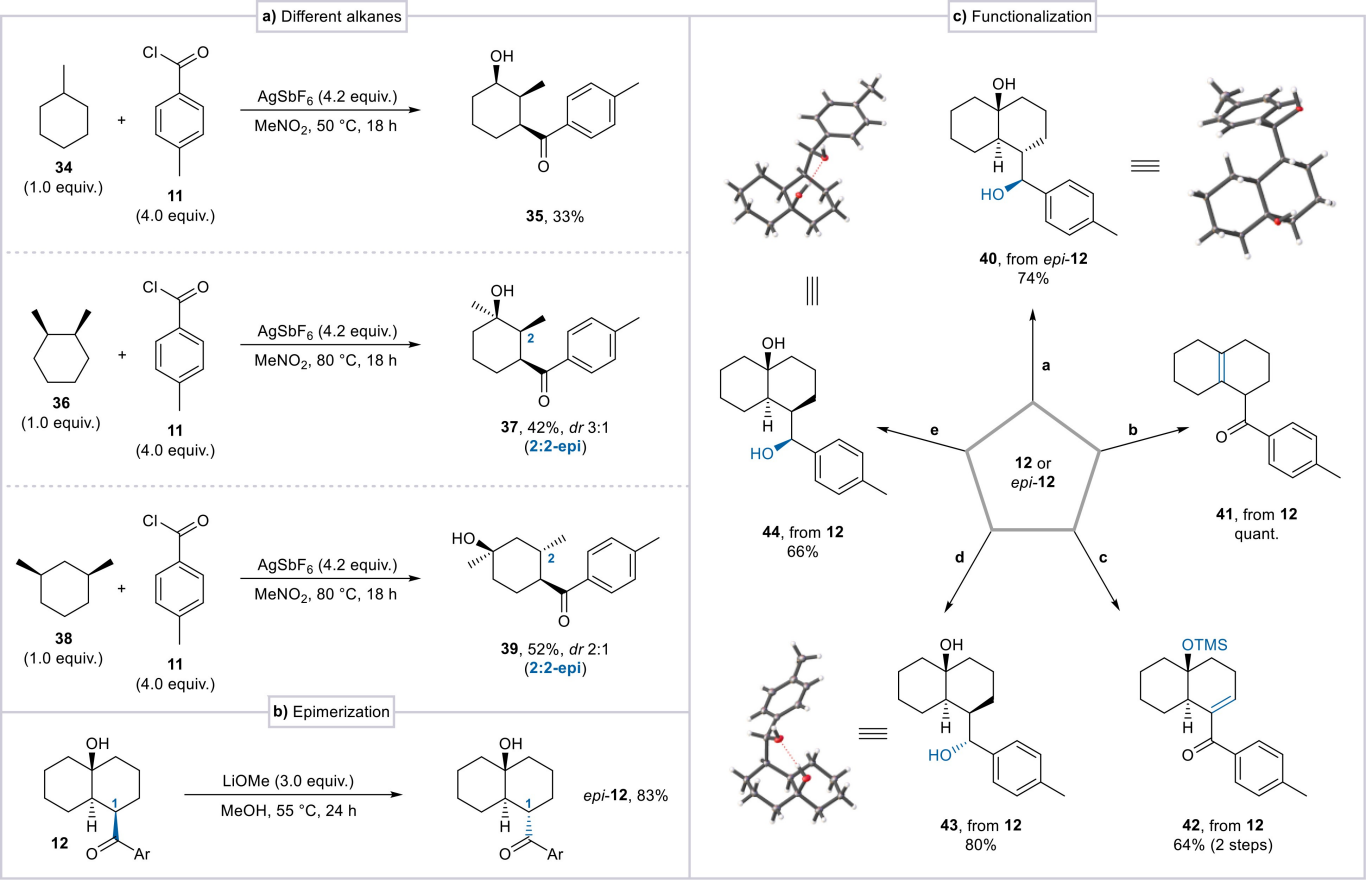
Application to different alkane substrates and product transformations. Conditions **a**: *epi*‐**12** (0.05 mmol), L‐selectride (3.0 equiv.), THF (0.1 M), −78 °C to 23 °C, 18 h. Conditions **b**: **12** (0.2 mmol), HC(OMe)_3_ (3.0 equiv.), *p*‐TsOH (0.3 equiv.), PhMe (0.1 M), 50 °C, 3 h. Conditions **c**: 1.) **12** (0.1 mmol), TMSOTf (3.0 equiv.), NEt_3_ (5.0 equiv.), DCM (0.1 M), 0 °C to 23 °C, 18 h; 2.) Pd(OAc)_2_ (2.0 equiv.), DMSO (0.1 M), 80 °C, 18 h. Conditions **d**: **12** (0.05 mmol), L‐selectride (3.0 equiv.), THF (0.1 M), −78 °C to 23 °C, 18 h. Conditions **e**: **12** (0.05 mmol), DIBAL−H (3.0 equiv.), DCM (0.1 M), −78 °C to 23 °C, 18 h.

After ascertaining that scale‐up preparation of product **12** (3.2 mmol) proceeds with an identical yield as in our scope studies (Scheme 3), we hypothesized that the rich functionality of the products obtained might lend itself to further manipulations. We eventually found that treatment of **12** with an excess of LiOMe led to full epimerization at C1, giving access to the diastereomer *epi*‐**12** in excellent 83 % yield (Scheme 6b). We subsequently investigated reductive conditions and successfully achieved a stereoselective reduction of both epimers, yielding three distinct diastereomeric diols (**40**, **43**, **44**, Scheme 6c).^[33]^ This array of simple transformations demonstrates the power of this transformation: four stereogenic centers installed on an achiral, unfunctionalized alkane^[34]^ in two steps with complete diastereoselectivity. Furthermore, formation of a silyl enol ether, followed by Saegusa‐Ito oxidation afforded the TMS‐protected enone **42** in overall 64 % yield, while water elimination afforded octalin **41** in a quantitative yield.

In summary, we have explored the reaction of highly electrophilic acylium ions with decalin, resulting in a regio‐ and diastereoselective difunctionalization of this fully aliphatic substrate. Not only do the exquisite selectivity and generally high yield of this transformation set it apart from the previously developed Baddeley reaction, but, under our conditions, we are able to obtain an unprecedented difunctionalized decalin regioisomer. Additionally, the application of this method translated well to a range of simpler alkane substrates. Mechanistic studies suggest that the reaction follows a pathway involving hydride abstraction and electrophilic addition steps, albeit, in comparison with the Baddeley reaction, with a difference in the regioselectivity of the acylium addition. The amenability of the products to further transformations harnessing their rich decoration suggests that further developments of this reaction hold the potential to afford new entries into target‐oriented synthesis.

## Supporting Information

The authors have cited additional references within the Supporting Information.[^35^, ^36^, ^37^, ^38^, ^39^, ^40^, ^41^, ^42^, ^43^, ^44^, ^45^, ^46^, ^47^, ^48^, ^49^, ^50^, ^51^, ^52^, ^53^]

## Conflict of Interests

The authors declare no conflict of interest.

## Supporting information

As a service to our authors and readers, this journal provides supporting information supplied by the authors. Such materials are peer reviewed and may be re‐organized for online delivery, but are not copy‐edited or typeset. Technical support issues arising from supporting information (other than missing files) should be addressed to the authors.

Supporting Information

## Data Availability

The data that support the findings of this study are available in the supplementary material of this article.
