# Supplementary material for: Revisiting the Baddeley Reaction: Access to Functionalized Decalins by Charge‐Promoted Alkane Functionalization
Source: Angew Chem Int Ed Engl. 2024 Nov 20;64(5):e202418067. doi: 10.1002/anie.202418067 (PMC11773124; doi:10.1002/anie.202418067)
Supplement: Supplementary file 1 — Supporting Information [file ANIE-64-e202418067-s001.pdf]

Angewandte Chemie
Eine Zeitschrift der Gesellschaft Deutscher Chemiker

Supporting Information

Revisiting the Baddeley Reaction: Access to Functionalized Decalins by Charge-Promoted Alkane Functionalization

*M. Vavřík, P. S. Grant, D. Kaiser, T. Gruene, N. Maulide**

Revisiting the Baddeley Reaction: Access to Functionalized Decalins by Charge-promoted Alkane Functionalization

Miloš Vavrík[a], Phillip S. Grant[a], Daniel Kaiser[a], Tim Grüne[b] and Nuno Maulide*[a]

[a] Institute of Organic Chemistry, University of Vienna, Währinger Straße 38, 1090 Vienna (Austria) E-mail: nuno.maulide@univie.ac.at

[b] X-Ray Structure Analysis Centre, University of Vienna, Währinger Straße 38, 1090 Vienna (Austria)

Supporting Information

Table of Contents

1. Detailed discussion on limitations of the Baddeley reaction 2

2. Optimization 4

3. Mechanistic proposal & additional experiments 6

4. Experimental Section 8

4.1 General Information 8

4.2 Nitromethane purification 9

4.3 Mechanistic Experiments 10

4.4 Modified Baddeley reaction 35

General Procedure A: Modified Baddeley reaction for electron rich & electron neutral acyl chlorides 35

General Procedure B: Modified Baddeley reaction for electron poor acyl chlorides 55

General Procedure C: Modified Baddeley reaction for aliphatic acyl chlorides 65

4.5 Large scale preparation of 12 73

4.6 Modified Baddeley reaction with different alkane substrates 74

4.7 Full scope of alkane substrates 90

4.8 Synthesis of miscellaneous alkane substrates 98

4.9 Functionalization reactions 114

Epimerization of 12 (*epi*-12) 114

Reduction of *epi*-12 with L-selectride (40) 116

Water elimination to octalin (41) 118

Saegusa–Ito oxidation (42) 120

Reduction of 12 with L-selectride (43) 122

Reduction of 12 with DIBAL-H (44) 124

5. X-ray Analysis 126

6. References 157

1. Detailed discussion on limitations of the Baddeley reaction

The Baddeley reaction with acetyl chloride was first reported in 1959 by G. Baddeley, who found that the reaction affords a mixture of products **2**, **3** and **4** in low to moderate yields (Scheme **S1a**).[13] In 1960, a variation of the reaction was described, with altered stoichiometry and reaction temperature, affording the vinyl ether **5** in 35–40% yield as a single isolable product (Scheme **S1b**).[14] Acidic hydrolysis of this compound was reported to give keto alcohol **6** in a yield of 68% (Scheme **S1b**).[35] In 1961, yet another variation was introduced, affording small amounts (4%) of regioisomeric keto alcohol **S1** as a by-product (Scheme **S1c**).[25]

In 2013, Lewis *et al*. reinvestigated the reaction, reporting the isolation of **5** in a reduced yield of only 25% (Scheme **S1b**).[36] Additionally, the hydrolysis of **5** was reported in 2011 by the same group to afford **6** in a yield of 51% (Scheme **S1b**).[21] Therefore, the overall yield of keto alcohol **6** has been reported as 13% or 27%, depending on the source.

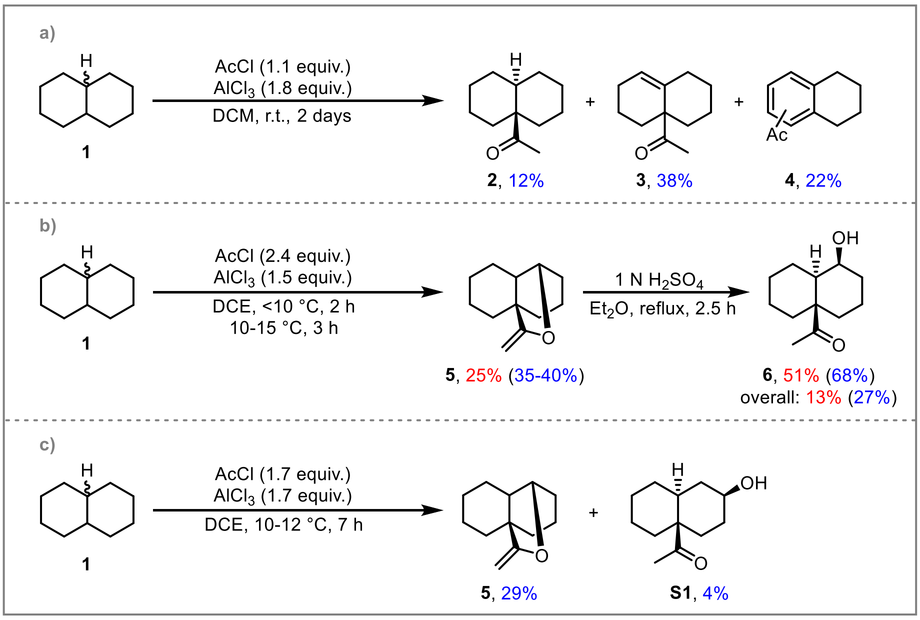

Scheme S1. Summary of experimental conditions and results reported by Baddeley *et al.* (blue) and Lewis *et al*. (red).

In 2014, Lewis et al. delved into the reaction mechanism through a computational study, the results of which are illustrated in Scheme **S2**.[20] The findings conclusively affirmed that the reaction progresses through two stages: a hydride abstraction mediated by the acylium ion (Step 1), and an electrophilic acylium addition to $\Delta^{9,10}$-octalin (Step 2).

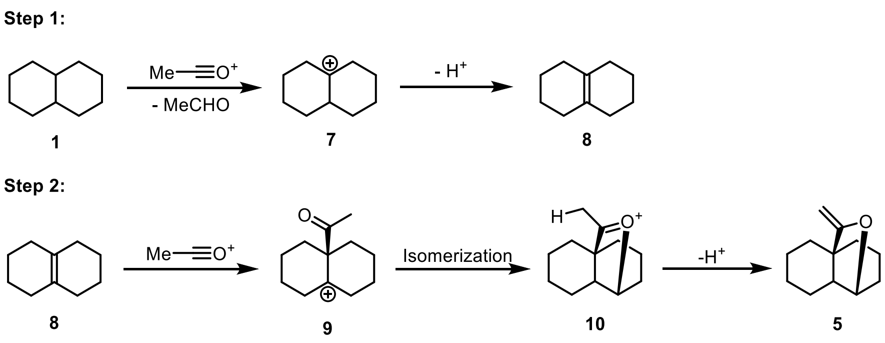

Scheme S2. Mechanism of formation of **5** (S. Lewis), supported by computational evidence.

The Baddeley reaction was reported with only three acyl chloride substrates: acetyl chloride, propionyl chloride and isobutyryl chloride (Scheme **S3**).[13–16,25,35] A noteworthy observation is the considerable variation in reaction stoichiometries across these substrates. This, in conjunction with the outcomes delineated in Scheme **S1**, suggests that the conditions were optimized for each acyl chloride individually, indicating a lack of general applicability.

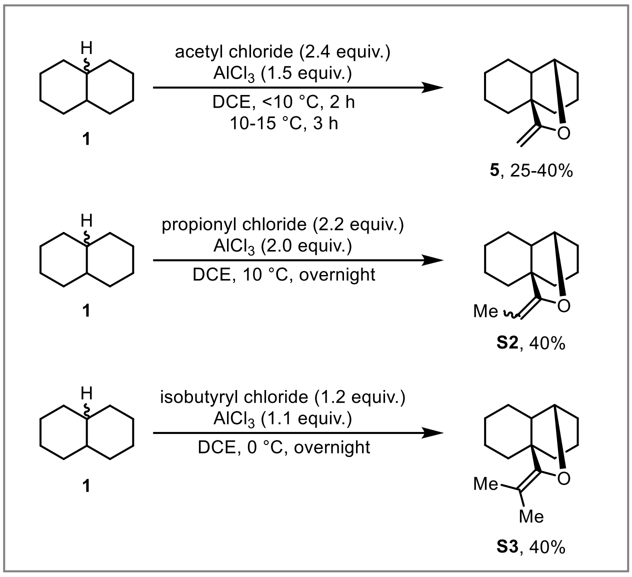

Scheme S3. Full scope of the Baddeley reaction reported in the literature.

Following our initial observation of the regioisomeric keto alcohol **12**, we attempted to conduct the reaction with the same aromatic acyl chloride using standard Baddeley conditions (Scheme **S4**). In this case the reaction afforded a complex mixture of products, with an overall yield (based on ^{1}H NMR analysis of the crude reaction product using mesitylene as internal standard) of less than 10%.

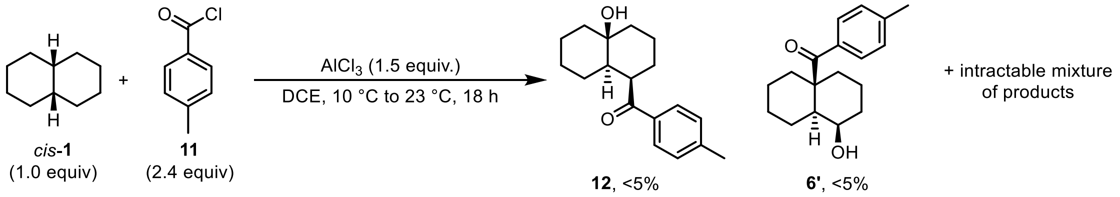

Scheme S4. The Baddeley reaction failed with a non-enolizable aromatic acyl chloride. The reaction was conducted as described in the literature, replacing acetyl chloride with **11**.[14,36]

2. Optimization

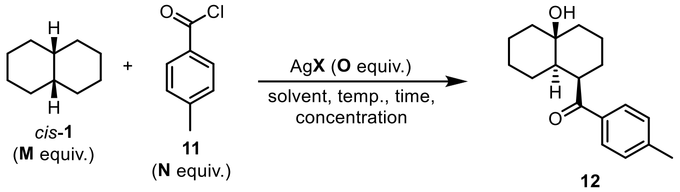

Entry	M	N	O	X⁻	solvent	temp. (°C)	time (h)	concentration	yield
1	1.0	2.0	2.1	SbF_6	DCM	23	18	0.1 M	36%
2	1.0	2.0	2.1	SbF_6	DCM	23	**0.25**	0.1 M	15%
3	1.0	2.0	2.1	SbF_6	DCM	23	**6**	0.1 M	26%
4	1.0	2.0	2.1	SbF_6	DCM	**35**	6	0.1 M	26%
5	1.0	2.0	2.1	SbF_6	DCM	**-78 to 23**	18	0.1 M	1%
6	1.0	**3.2**	**3.3**	SbF_6	DCM	23	18	0.1 M	39%
7	1.0	**6.0**	**6.0**	SbF_6	DCM	23	18	0.1 M	49%
8	1.0	**5.0**	**3.3**	SbF_6	DCM	23	18	0.1 M	37%
9	1.0	2.0	2.1	SbF_6	**$CHCl_3$**	23	18	0.1 M	40%
10	1.0	2.0	2.1	SbF_6	**DCE**	23	18	0.1 M	22%
11	1.0	2.0	2.1	SbF_6	**C_6F_6**	23	18	0.1 M	6%
12	1.0	2.0	2.1	SbF_6	**$MeNO_2$ (wet)[a]**	23	18	0.1 M	3%
13	1.0	2.0	2.1	SbF_6	**$MeNO_2$[b]**	23	18	0.1 M	52%
14	1.0	2.0	2.1	SbF_6	**$EtNO_2$**	23	18	0.1 M	54%
15	1.0	**3.0**	**3.1**	SbF_6	$MeNO_2$[b]	23	18	0.1 M	75%
16	1.0	2.0	2.1	SbF_6	$MeNO_2$[b]	23	18	**0.2 M**	64%
17	1.0	2.0	2.1	SbF_6	$MeNO_2$[b]	23	18	**0.3 M**	58%
18	1.0	2.0	2.1	SbF_6	$MeNO_2$[b]	23	18	**0.05 M**	31%
19	1.0	2.0	2.1	SbF_6	$MeNO_2$[b]	**50**	18	0.1 M	54%
20	1.0	2.0	2.1	SbF_6	$MeNO_2$[b]	**-20 to 23**	18	0.1 M	58%
21	1.0	2.0	2.1	**BF_4**	$MeNO_2$[b]	23	18	0.1 M	3%
22	1.0	2.0	2.1	**PF_6**	$MeNO_2$[b]	23	18	0.1 M	19%
23	**6.0**	2.0	2.1	SbF_6	$MeNO_2$[b]	23	18	0.1 M	59%
24	**6.0[c]**	2.0	2.1	SbF_6	$MeNO_2$[b]	23	18	0.2 M	53% (51%†)
25	**6.0[d]**	2.0	2.1	SbF_6	$MeNO_2$[b]	23	18	0.2 M	27% (26%†)
26	6.0	2.0	2.1	SbF_6	$MeNO_2$[b]	23	18	0.2 M	69% (70%†)

Table S1. Reaction optimization. 1.0 equiv = 0.1 mmol. Concentrations are given with respect to the theoretical product yield (0.1 mmol in all cases). Conditions: decalin (**M** equiv.; *cis*-configured, unless otherwise specified), *p*-toluoyl chloride (**N** equiv.), AgX (**O** equiv.), solvent (0.33–2.0 mL). The reactions were conducted as described in **General Procedure A**, see section **4.4**. Yields were determined by ^{1}H NMR analysis of the crude reaction products using mesitylene as internal standard. [a]As received from commercial suppliers. [b]Dried over CaH_2 and distilled, see Section **4.2** for more details. [c]Technical mixture of isomers (*cis*:*trans* = 1.2:1). [d]*trans*-Decalin. †Isolated yield.

In cases where an excess of *cis*-**1** is employed, yields are determined based on 1.0 equiv. **11** as the limiting reagent (2.0 equiv. of which are used for this reaction). This is a result of, in an ideal case, 1.0 equiv. acylium ion (generated from **11**) being consumed by hydride abstraction and octalin formation (see below for a detailed discussion).

Notably, three alternative scenarios are possible:

1) **Hydride abstraction is *slower* than addition of the acylium ion to octalin**
   In this case, any octalin that is formed would be rapidly consumed by a relative excess of acylium ion. However, seeing as product can only be formed by reaction of acylium ion with octalin, the relative excess of the electrophilic species is ultimately irrelevant. This step can only reach completion after quantitative conversion of 1.0 equiv. *cis*-**1** and 1.0 equiv. **11** to give 1.0 equiv. octalin.
2) **Hydride abstraction is *faster* than addition of the acylium ion to octalin**
   In this case, a relative rate increase of octalin formation would deplete the reservoir of acylium ion, leading to a lower amount of this limiting reagent available for product formation. The yield of the desired product **12** would be lower and the yield of the unreacted intermediate octalin would be higher.
3) **Hydride abstraction is not quantitative**
   In this scenario, the maximum yield would be limited by the amount of octalin formed, despite the fact that some acylium ion would remain in the reaction mixture.

Thus, we believe that calculating the yield in the manner described above is the most accurate and honest approach, as the determined value is either the result of scenario 1 (ideal; maximum yield 100%) or scenarios 2 and 3 (not ideal; maximum yield <100%).

3. Mechanistic proposal & additional experiments

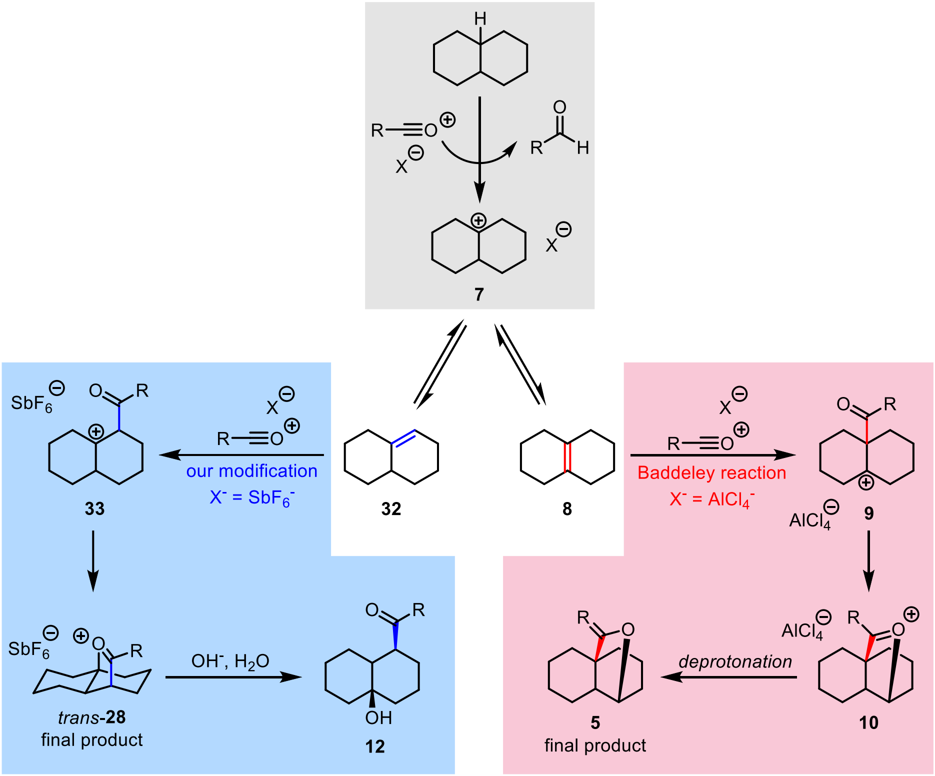

Scheme S5. Proposed reaction mechanism. The initial step (gray) is identical to the Baddeley reaction; the key difference is the regioselectivity of the electrophilic addition of acylium ion to octalin (blue versus red pathway).

The following evidence supports this proposal (for experimental details, see the sections specified at the beginning of each paragraph):

1. (Section **4.3a**) *trans*-**28** was observed by ^{1}H and ^{13}C NMR spectroscopy as the main reaction product and was stable over a period of 64 hours (no change observed in the ^{1}H NMR spectrum). This indicates that, under our conditions, the reaction terminates at the oxocarbenium stage, with no side-reactions (such as deprotonation) taking place.

2. (Sections **4.3b** and **4.3e**) The same regioisomer is observed when the reaction is conducted with $\Delta^{9,10}$-octalin (**8**) as the starting material, which proves that an isomerization process between the two octalin isomers is operative, with $\Delta^{1,9}$-octalin (**32**) acting as the preferential nucleophile under our conditions. The observation of regioisomeric mixtures with less sterically demanding acyl chlorides (R = Me, Et) suggests that the selectivity of this process is likely at least partially influenced by steric factors. The octalin isomerization step seems to be acid catalyzed, since in presence of a stoichiometric amount of 2,6-di-*tert*-butylpyridine no reaction occurred between $\Delta^{9,10}$-octalin and benozyl chloride + $AgSbF_6$. Furthermore, a detrimental impact of a substoichiometric amount of 2,6-di-*tert*-butylpyridine on the yield of the standard reaction (*cis*-decalin + benzoyl chloride + $AgSbF_6$) was observed.

3. (Section **4.3c**) The anticipated aldehyde by-product, together with the product of Henry addition, was observed (see Scheme **4c**). However, in the majority of instances, the cumulative yield of these by-products was below 10%. Similar results were obtained with non-volatile aldehydes, eliminating aldehyde volatility as the primary causative factor. Previous research by Lewis *et al.* demonstrated that in the Baddeley reaction with acetyl chloride, acetaldehyde participates in the formation of 1-chloroethyl acetate, a product resulting from reaction of the aldehyde with an acylium ion and $AlCl_4^-$.[36] Notably, we did not observe the generation of such halogenated esters in our reaction or in a control experiment conducted

in the absence of decalin (Scheme **S6**). This observation leads us to posit that the aldehyde undergoes non-specific decomposition reactions, such as polymerization (yielding insoluble compounds) or the formation of water-soluble products that persist in the aqueous phase.

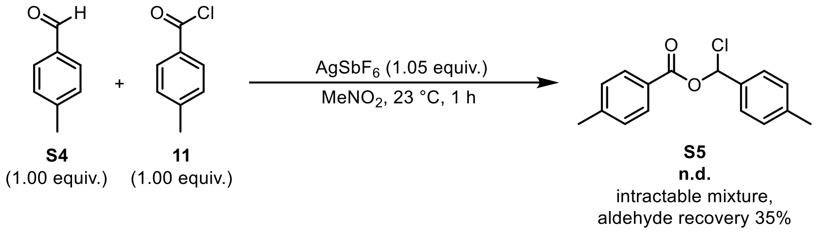

Scheme S6: Chlorinated ester **S5** was not detected in the crude reaction product (^{1}H and ^{13}C NMR analysis). Conditions: **S4** (0.1 mmol), **11** (1.0 equiv.), $MeNO_2$ (1.0 mL), 23 °C, 1 h. The reaction was conducted as described in **General Procedure A** (see section **4.4**), with *cis*-decalin being replaced by **S4**. Aldehyde recovery was quantified by ^{1}H NMR analysis of the crude reaction product using mesitylene as internal standard.

4. (Section **4.3d**) Some of our results suggested that the reaction might operate under thermodynamic control, with the conditions (highly polar solvent, weakly coordinating counterion) allowing a more efficient equilibration, in contrast to the "normal" Baddeley reaction with $AlCl_3$. This could explain why, in the case of aromatic acyl chlorides the reaction is fully selective (better stabilization of the aromatic acylium ion), while in the case of aliphatic acyl chlorides the reaction affords a mixture of regioisomers (**25** and **26**), with a higher *r.r.* at elevated temperature. As kindly suggested by one of our reviewers, we tested this hypothesis with a crossover experiment (Scheme **S7**).

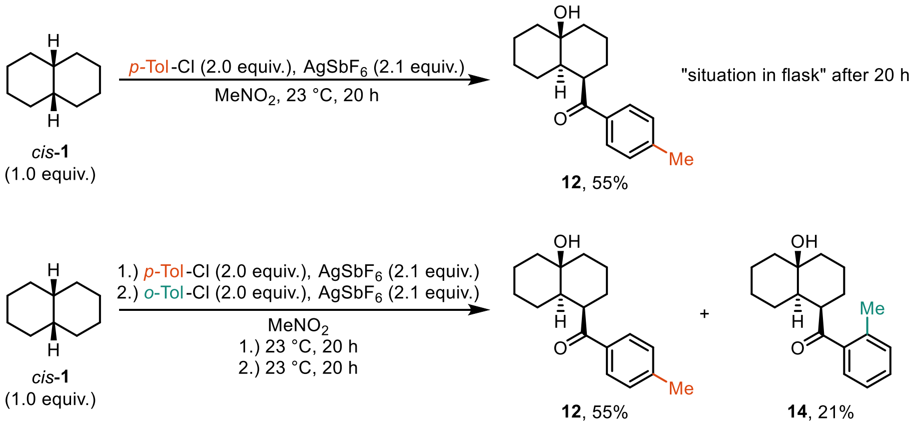

Scheme S7: Crossover experiment disproving reversibility of the acylium addition step. For experimental details see Section **4.3d**. *p*-Tol-Cl = *para*-toluoyl chloride; *o*-Tol-Cl = *ortho*-toluoyl chloride.

The obtained results, however, do not indicate reversibility, as the yield of the *para*-toluoyl product **12** remained constant after the addition of a second acylium ion. Of note, under equilibrating conditions, the addition of *ortho*-toluoyl acylium ion should have led to a higher yield of **12** (while the stoichiometry of acylium ions appears to be 2 equiv.:2 equiv., the sacrificial equivalent of *para*-toluoyl acylium in the first step has to be considered, effectively leading to an excess of *ortho*-toluoyl acylium). Additionally, the overall yield was found to increase (with a constant yield of **12**), hinting towards increased conversion rather than equilibration.

4. Experimental Section

4.1 General Information

Unless otherwise stated, all glassware was flame-dried before use and all reactions were performed under an atmosphere of argon. The given reaction temperature refers to the temperature of the surrounding (cooling/heating) medium, and not to an internally measured temperature. Anhydrous solvents and reagents were used as received from commercial suppliers unless otherwise stated. Reaction progress was monitored by thin layer chromatography (TLC) performed on aluminium plates coated with silica gel F254 with 0.2 mm thickness. Compounds were visualized by fluorescence quenching with UV light at 254 nm or by staining using potassium permanganate or phosphomolybdic acid, followed by heating. Flash column chromatography was performed using silica gel 60 (0.04 – 0.063 mm), unless otherwise stated. Neat infrared spectra were recorded using a Perkin-Elmer Spectrum 100 FT-IR spectrometer. Wavenumbers (v_{max}) are reported in cm^{-1}. Mass spectra were obtained using a Bruker maXis UHR-TOF (QQ-TOF) spectrometer, using electrospray ionization (ESI) or an Agilent 7200B GC/Q-TOF spectrometer, using electron ionization (EI). All ^{1}H NMR and ^{13}C NMR spectra were recorded using Bruker AV-400, AV-600 or AV-700 spectrometers at 298 K, unless stated otherwise. Chemical shifts (δ) are given in parts per million (ppm) and referenced to the given solvent peak as described in literature.[37] Coupling constants (*J*) are reported in Hz and the splitting patterns are designated as singlet (s), doublet (d), triplet (t) and quartet (q) as they appeared in the spectrum. If the appearance of a signal differs from the expected splitting pattern, the observed pattern is designated as apparent (app). Splitting patterns that could not be interpreted or easily visualized are designated as multiplet (m) or broad (br). DEPTQ ^{13}C NMR spectra are phased such that CH_2/C signals are negative, and CH/CH_3 signals are positive. Unless otherwise stated, the compounds were isolated as racemic mixtures and the stereochemistry drawn illustrates only their relative configuration.

4.2 Nitromethane purification

During our optimization studies, we noticed an influence of nitromethane quality on the reaction performance (see Table **S1**, Entries 12–13). Nitromethane obtained from commercial suppliers typically contains water as an impurity, which was observed to have a detrimental impact on reaction yield. We attempted to dry the solvent using 4 Å molecular sieves (pre-activated in oven at 120 °C, followed by drying in high vacuum at 150 °C for 18 hours), which increased the yield from 3% to 32%. However, best results were obtained by drying $MeNO_2$ over calcium hydride, followed by a distillation:

A flame-dried 250 mL round bottom flask equipped with a large stirring bar was charged with 5.0 g of powdered CaH_2. An air condenser with a vacuum inlet was connected to the flask and the apparatus was evacuated/backfilled with argon three times. Approximately 75 mL $MeNO_2$ were added under stirring at 23 °C (the air condenser was shortly removed under positive Ar pressure, and the solvent was poured directly into the flask; effervescent reaction occurs). The resulting suspension was refluxed for 3 hours (oil bath 150 °C, air condenser connected to argon line with a constant flow of Ar), after which the contents were allowed to cool to room temperature. The air condenser was then replaced with a Claisen adapter attached to a Liebig condenser with a collecting flask and an argon inlet (this part was evacuated/backfilled with argon three times beforehand) and the solvent was distilled at 140 to 160 °C (oil bath temperature). The first 5 mL of distillate were discarded, after which the collecting flask was replaced with a large flame-dried Schlenk flask charged with pre-activated (dried in high vacuum at 150 °C over 18 hours) 4 Å molecular sieves (approx. 10% of total flask volume). The distillation was continued until approx. 90% of total solvent volume evaporated, after which heating was stopped, the Schlenk flask was removed under positive Ar pressure and immediately sealed with a septum. The septum was wrapped with parafilm, and the so-obtained purified nitromethane was used as reaction solvent (standard Schlenk techniques).

After several days, yellow coloration of the solvent was observed, which did not have any impact on reaction performance. We did not observe any decrease in yields after a storage period of 6 months (stored over 4 Å pre-activated molecular sieves, at 23 °C, in a Schlenk flask sealed with septum and parafilm).

Deuterated nitromethane ($MeNO_2$-d_3) was used directly as received from commercial suppliers, without further purification.

4.3 Mechanistic Experiments

Synthesis of Δ^{9,10}-octalin (8)

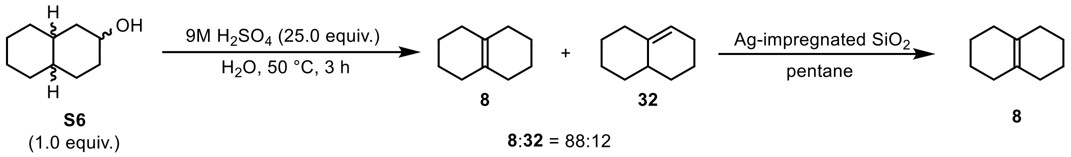

Decahydro-2-naphthol (500 mg, 3.24 mmol, 1.00 equiv.) was weighed into a Schlenk flask and 9 M H_2SO_4 (8.0 mL, 72.00 mmol, 25.00 equiv.) was added at ambient temperature (23 °C). The suspension was heated to 50 °C for 3 h, resulting in formation of a dark oil on the surface of the aqueous phase. After cooling to ambient temperature (23 °C), water (30 mL) was slowly added, and the mixture was transferred into a separation funnel (the reaction flask was washed with pentane, 2 × 5 mL). The aqueous layer was extracted with pentane (3 × 10 mL), the combined organic layers were washed with brine (25 mL), dried over anhydrous sodium sulfate, the dried solution was filtered, and the filtrate was concentrated under reduced pressure (min. 100 mbar, water bath 50 °C). The crude residue was purified using column chromatography (pentane) to afford a mixture of **8** and **32** in an 88:12 ratio (333 mg).

Silver impregnated SiO_2 and TLC plates were prepared according to Li *et al.*[38]

The mixture of olefins was separated using 20 g of Ag-impregnated SiO_2 (0-10% Et_2O in pentane, fractions analyzed with Ag-impregnated TLC plates, $KMnO_4$ stain), affording pure **8** as a colorless oil (120 mg, 27%). The trisubstituted olefin (**32**) elutes first, together with additional impurities which could not be identified.

The product is volatile and slowly evaporates already at room temperature under atmospheric pressure, therefore caution is to be taken during solvent removal (min. 100 mbar, water bath 50 °C).

All analytical data were in good accordance with data reported in the literature.[39]

^{1}H NMR (400 MHz, $CDCl_3$) δ 1.84 (app br, 8H), 1.64 – 1.54 (m, 8H).

^{13}C NMR (101 MHz, $CDCl_3$) δ 128.1 (2C), 30.6 ($4CH_2$), 23.4 ($4CH_2$).

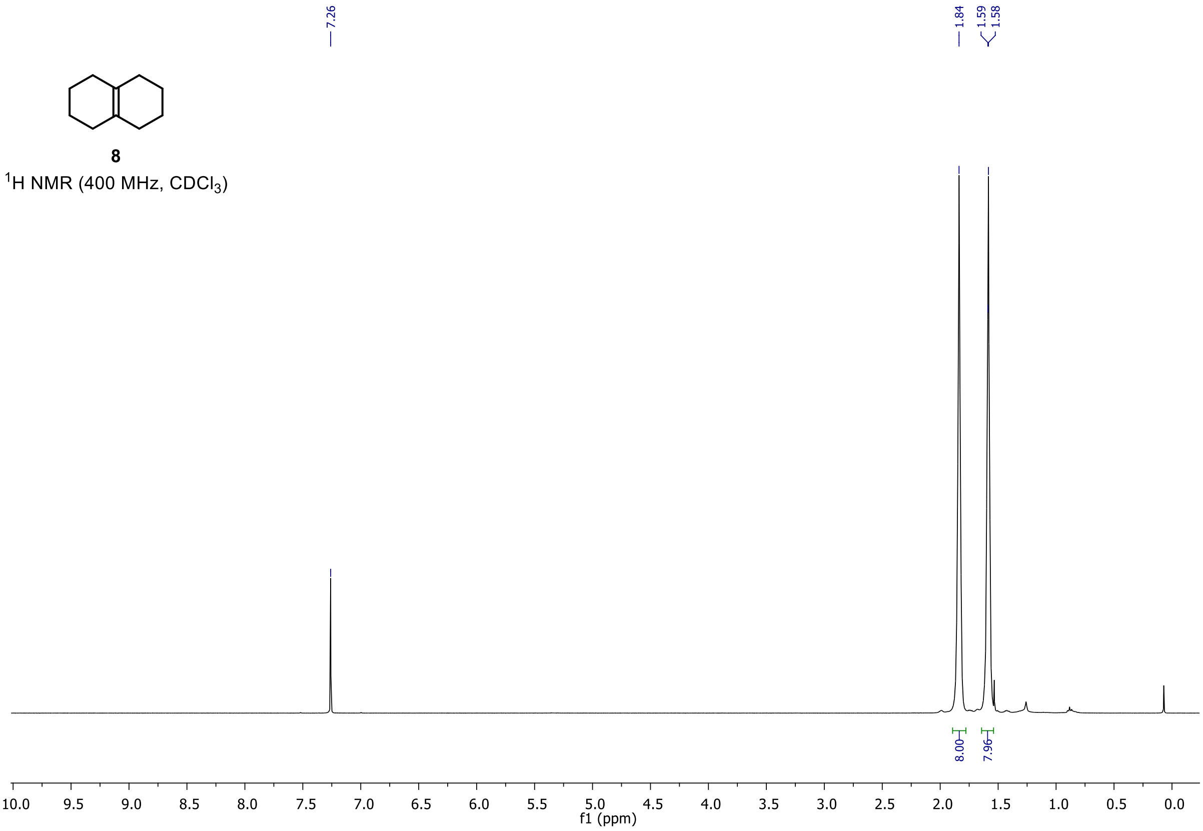

8

^{1}H NMR (400 MHz, $CDCl_3$)

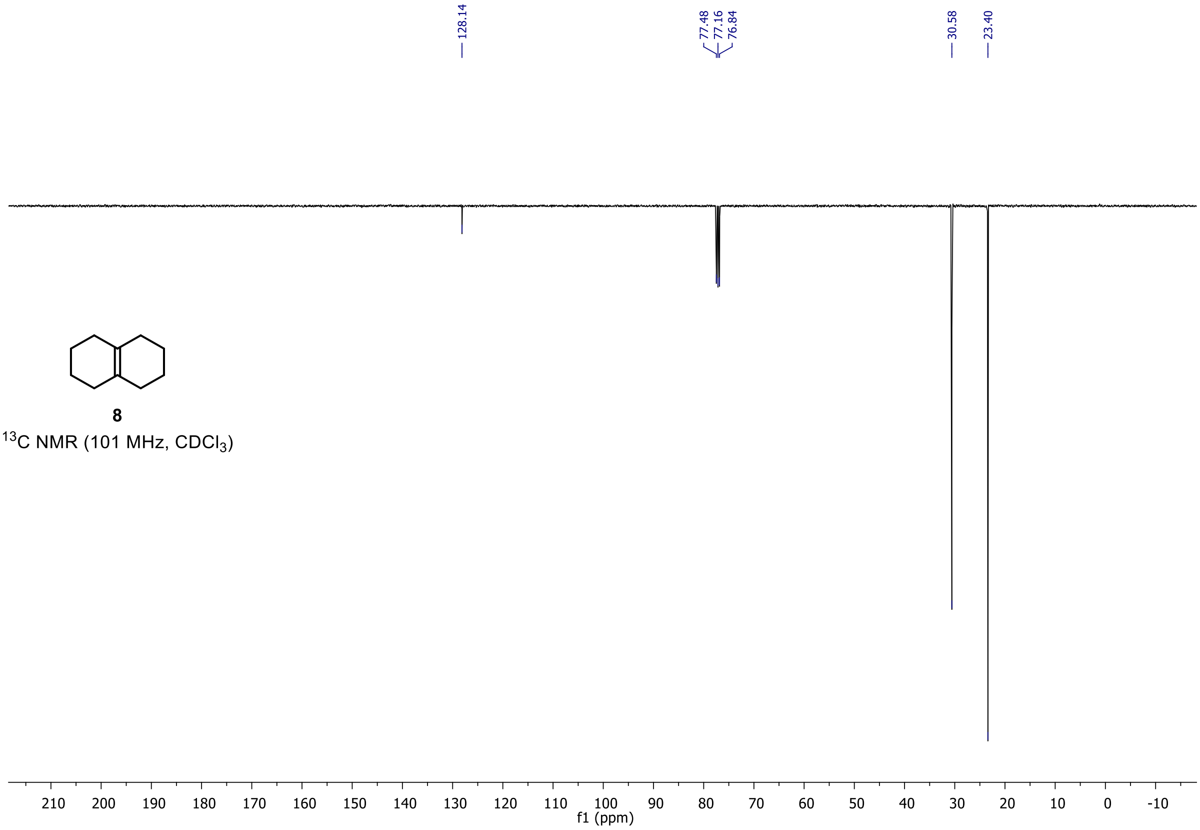

8

^{13}C NMR (101 MHz, $CDCl_3$)

a) Decalin Experiment (*trans*-**28**)

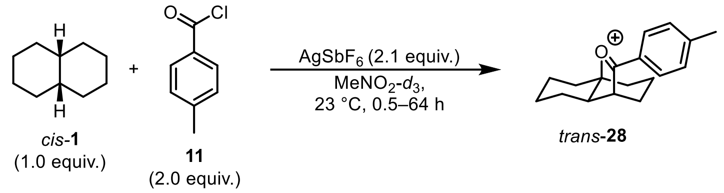

In a flame-dried vial (3 mL max. volume) with a rubber septum cap, $AgSbF_6$ (72 mg, 0.21 mmol, 2.10 equiv., weighed in glovebox) was dissolved in 0.7 mL $MeNO_2$-d_3 at ambient temperature (23 °C). *p*-Toluoyl chloride (31 mg, 0.20 mmol, 2.00 equiv.) was added under stirring at ambient temperature (23 °C), followed by *cis*-decalin (14 mg, 0.10 mmol, 1.00 equiv.). The resulting suspension was then stirred at ambient temperature (23 °C) for 15 minutes, after which the solution was transferred using an argon-purged syringe into an argon-flushed NMR tube. The NMR tube was immediately sealed with a silicone cap and parafilm, and NMR spectra (^{1}H NMR, ^{13}C NMR) were recorded in 30-minute increments over 18 hours at 298 K (first measurement exactly 30 minutes after reaction start). The NMR tube was then stored at 23 °C in the absence of light for 64 hours, after which a final measurement (^{1}H NMR and ^{13}C NMR) was conducted.

Since no change was observed after 2 hours of reaction time ($MeNO_2$ signal used as integration reference), the experiment was repeated and a full 1D and 2D NMR set was recorded after 2 hours (^{1}H NMR, ^{13}C NMR, COSY, HSQC, HMBC, NOESY, TOCSY, H2BC).

The structure of *trans*-**28** was initially proposed by retrospective analysis from the structure of product **12** (formed after aqueous workup and proven by SC-XRD). Furthermore, analysis of the known mechanism of the Baddeley reaction[20] led us to a putative conclusion that oxocarbenium *trans*-**28** should be the final product in our reaction, preceding the aqueous workup. This theory was confirmed by a more detailed NMR analysis (see below).

NMR data of *trans*-**28**:

^{1}H NMR (600 MHz, Nitromethane-d3) δ 8.44 (d, *J* = 8.3 Hz, 2H), 7.71 (d, *J* = 8.5 Hz, 2H), 4.39 – 4.36 (m, 1H), 2.65 (s, 3H), 2.53 (dd, *J* = 11.9, 6.6 Hz, 1H), 2.35 – 2.28 (m, 1H), 2.14 – 2.06 (m, 2H), 2.02 – 1.97 (m, 3H), 1.85 – 1.80 (m, 1H), 1.73 – 1.68 (m, 1H), 1.58 – 1.48 (m, 2H), 1.43 – 1.26 (m, 3H), 0.98 – 0.89 (m, 1H).

^{13}C NMR (151 MHz, Nitromethane-d3) δ 220.1 (C), 161.1 (C), 133.8 (2 CH), 132.4 (2CH), 124.1 (C), 115.1 (C), 57.1 (CH), 48.9 (CH), 33.0 (CH_2), 32.6 (CH_2), 29.2 (CH_2), 28.0 (CH_2), 23.6 (CH_2), 23.5 (CH_3), 21.6 (CH_2), 19.8 (CH_2).

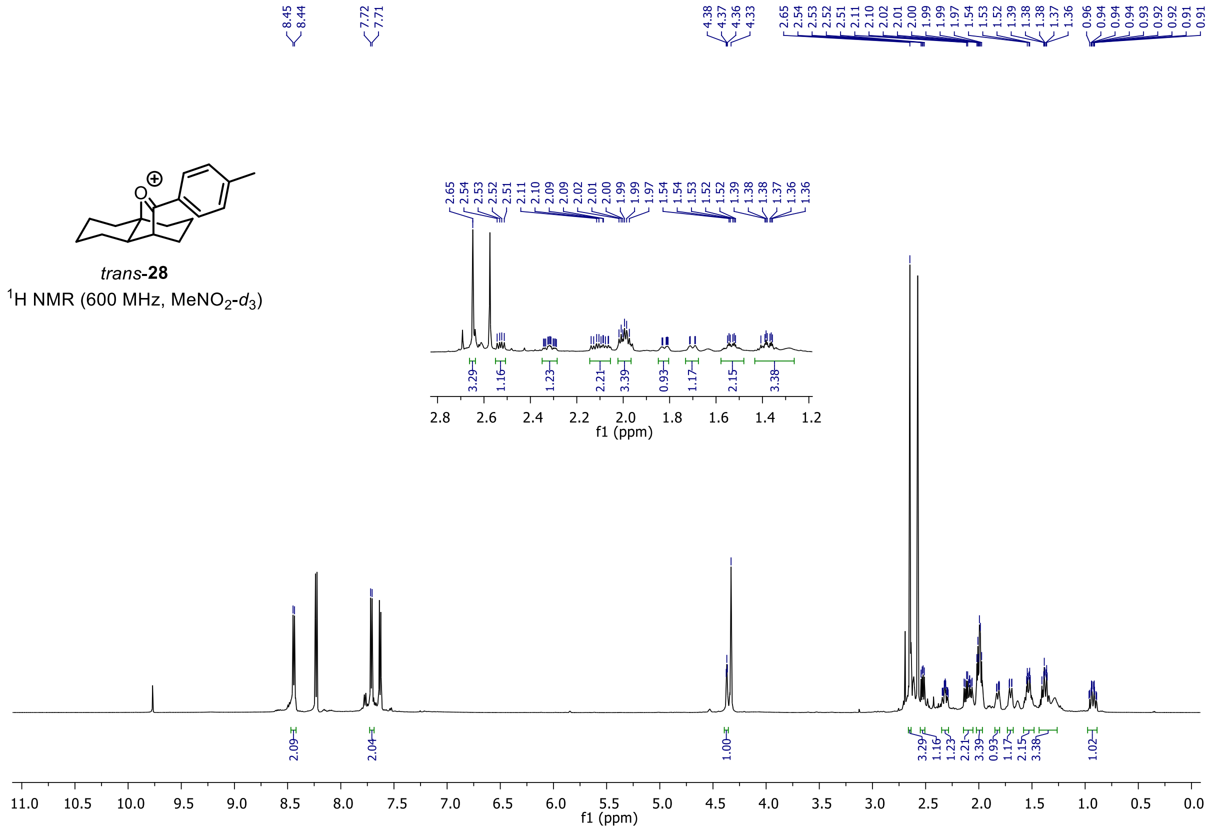

*^{1}H NMR spectrum of the reaction mixture in $MeNO_2$-d_3 (decalin experiment, 2 hours after reaction start). Integrated & peak-picked are signals arising from the oxocarbenium ion trans-**28**. Solvent signal is at 4.33 ppm. Additional aromatic compounds are present (p-tolualdehyde and acylium ion-derived species).*

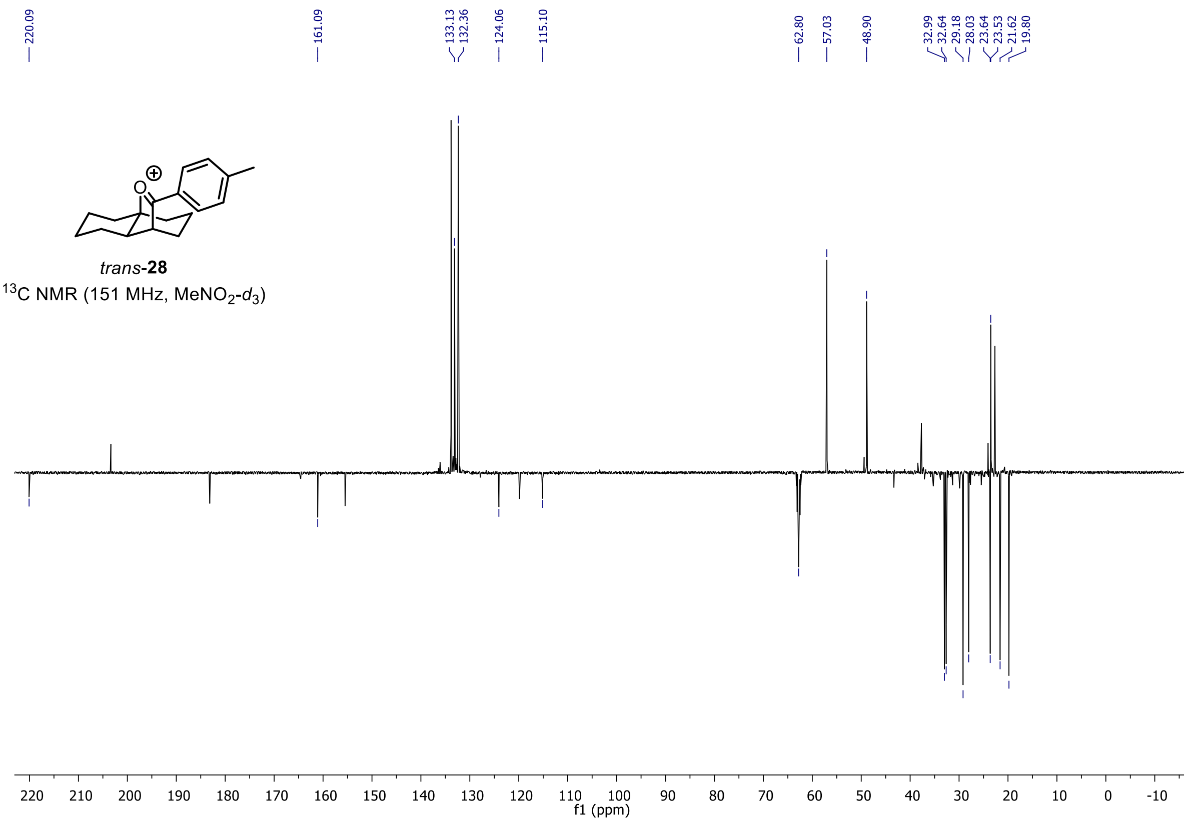

*^{13}C NMR spectrum of the reaction mixture in $MeNO_2$-d_3 (decalin experiment, 2 hours after reaction start). Peak-picked are signals arising from the oxocarbenium ion trans-**28**. Solvent signal is at 62.80 ppm.*

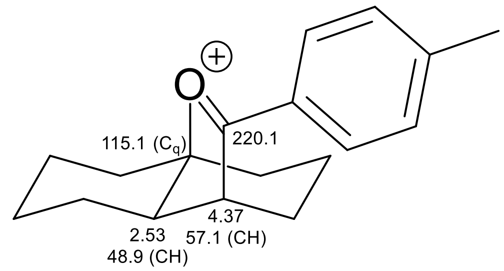

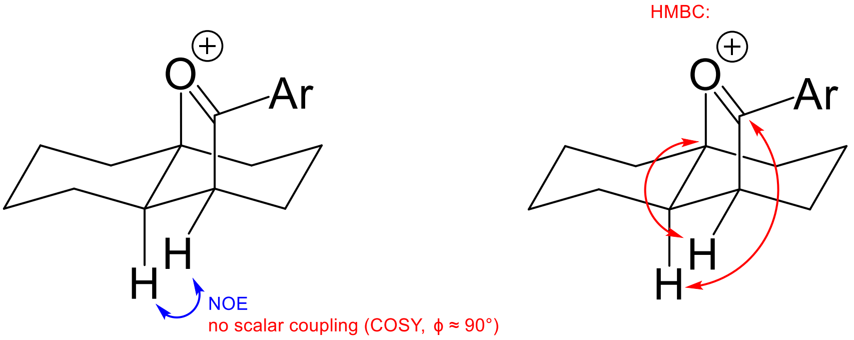

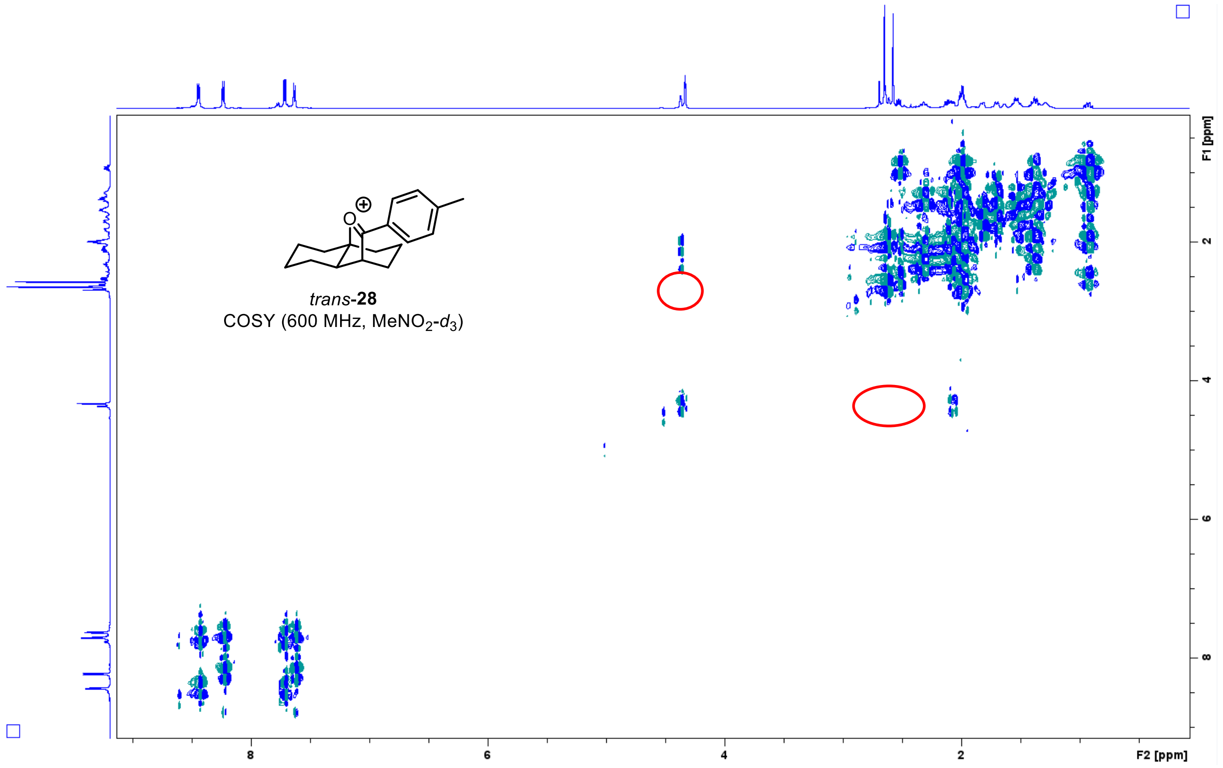

COSY spectrum of the reaction mixture, highlighted is the area where a crosspeak between 4.37 ppm + 2.53 ppm is missing

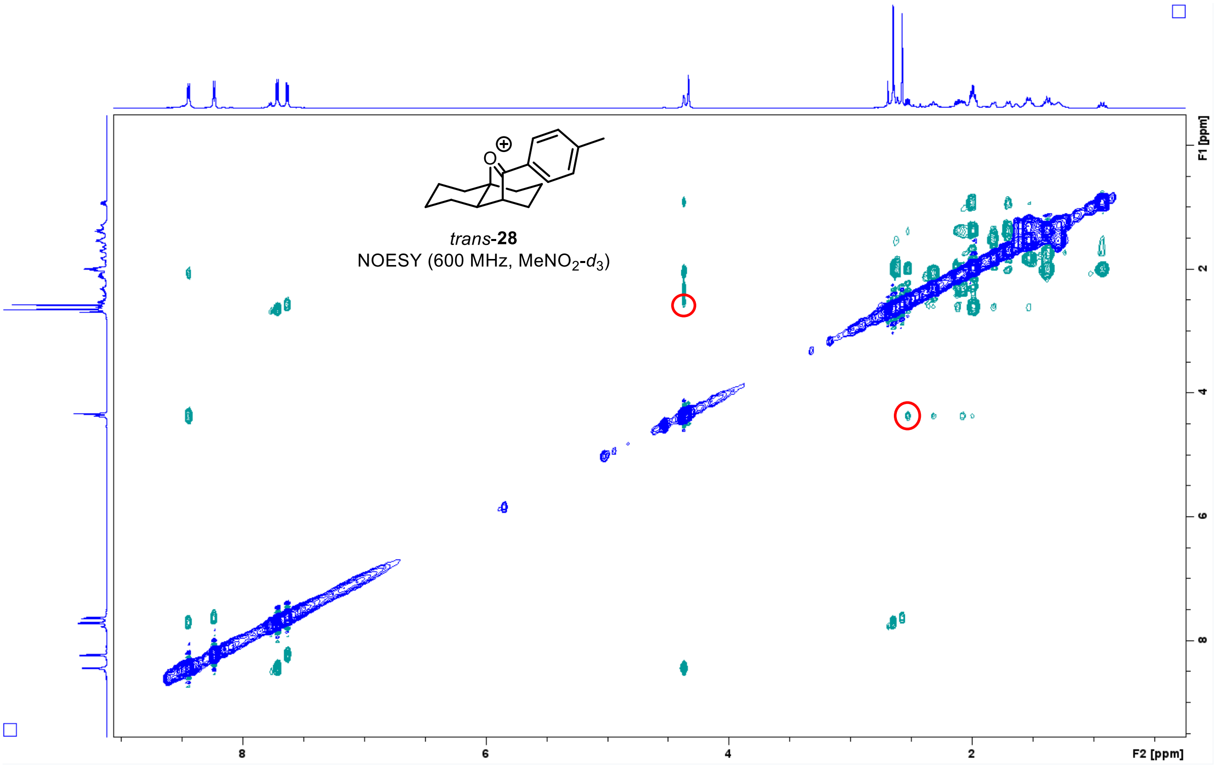

NOESY spectrum of the reaction mixture, highlighted are opposite-phase crosspeaks (= positive NOE) between 4.37 ppm + 2.53 ppm.

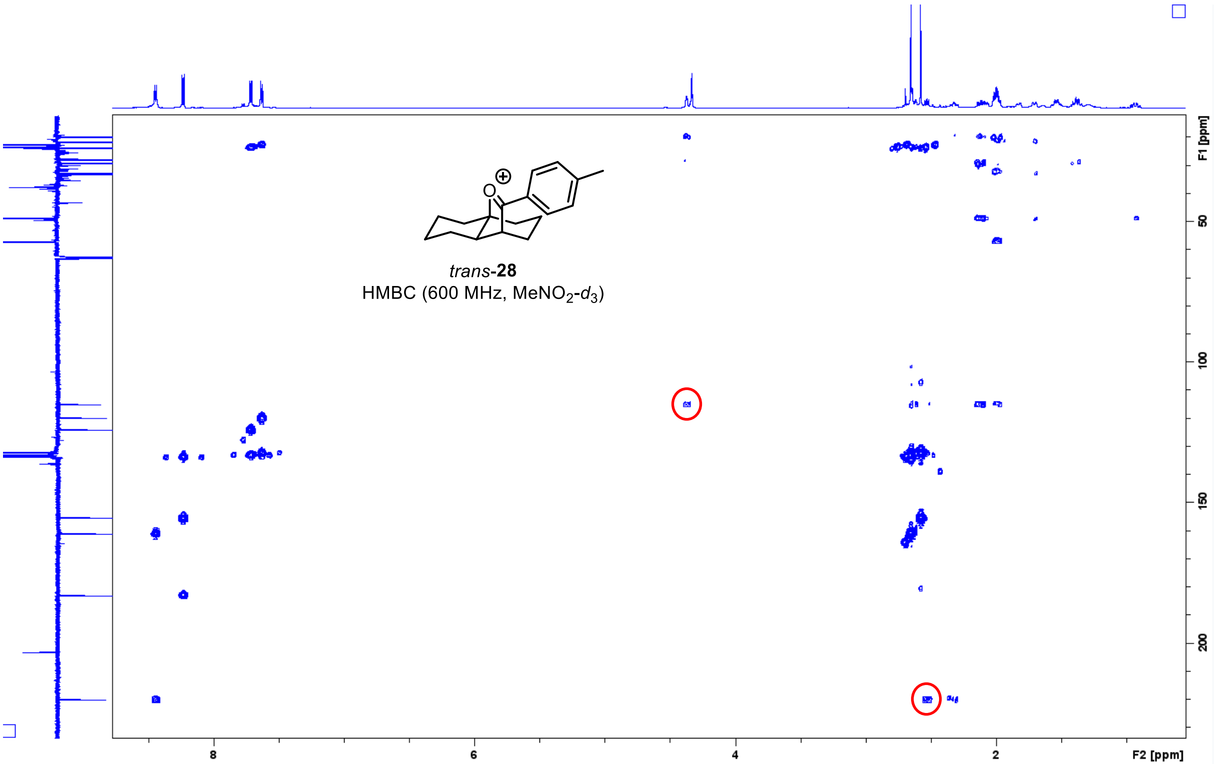

HMBC spectrum of the reaction mixture, highlighted are correlations between 115.1 ppm + 4.37 ppm ($^3J_{C–H}$) and 220.1 ppm + 2.53 ppm ($^3J_{C–H}$).

b) Octalin Experiment #1 (*cis*-**28** and *trans*-**28**)

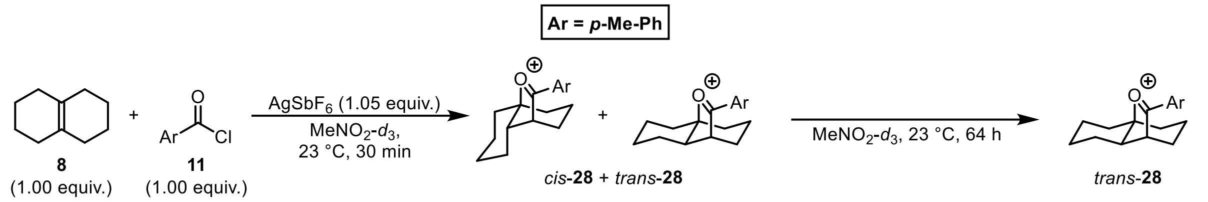

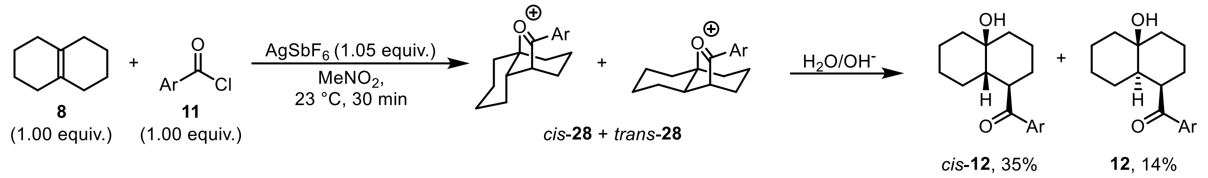

In a flame-dried vial (3 mL max. volume) with a rubber septum cap, $AgSbF_6$ (72 mg, 0.21 mmol, 1.05 equiv., weighed in glovebox) was dissolved in 0.7 mL $MeNO_2$-d_3 at ambient temperature (23 °C). *p*-Toluoyl chloride (31 mg, 0.20 mmol, 1.00 equiv.) was added under stirring at ambient temperature (23 °C), followed by $\Delta^{9,10}$-octalin (27 mg, 0.20 mmol, 1.00 equiv.). The resulting suspension was then stirred at ambient temperature (23 °C) for 15 minutes, after which the solution was transferred using an argon-purged syringe into an argon-flushed NMR tube. The NMR tube was immediately sealed with a silicone cap and parafilm, and NMR spectra (^{1}H NMR, ^{13}C NMR, COSY, HSQC, HMBC, NOESY, TOCSY, H2BC) were recorded at 298 K (first measurement exactly 30 minutes after reaction start). The NMR tube was then stored at 23 °C in the absence of light for 64 hours, after which a final measurement (^{1}H NMR and ^{13}C NMR) was conducted.

To obtain additional information on the structure of the second product (*cis*-**28**), the experiment was repeated under identical conditions, but with non-deuterated $MeNO_2$ and the reaction was stopped after 30 minutes by the addition of a 10% aqueous K_2CO_3 solution (see **General Procedure A** for the exact workup procedure). The two keto-alcohol isomers *cis*-**12** and **12** were separated by flash column chromatography (0–40% Et_2O in pentane).

The structure of *cis*-**28** was initially proposed based on similarities in ^{1}H and ^{13}C NMR spectra with *trans*-**28** (see below). Both ^{1}H and ^{13}C NMR spectra of *cis*-**12** (after workup) displayed broad signals at 298 K, a common phenomenon observed with *cis*-decalin scaffolds due to ring inversion. Measuring the spectra at 238 K resulted in line sharpening and appearance of missing decalin signals (two sets for two conformers), further hinting at the presence of a *cis*-decalin core. The presence of key NOEs, COSY crosspeaks and HMBC signals (see below) both in *cis*-**28** and *cis*-**12** confirmed this theory.

^{1}H and ^{13}C NMR spectra of *trans*-**28** were identical to those described in Section **4.3a**.

^{1}H and ^{13}C NMR spectra of **12** were identical to those described in Section **4.4**.

Due to numerous overlaps with *trans*-**28**, only the key (easily distinguishable) ^{1}H signals of *cis*-**28** were assigned (see below).

^{13}C NMR data for *cis*-**28**:

^{13}C NMR (151 MHz, Nitromethane-d3) δ 220.9 (C), 161.0 (C), 133.3 (2CH), 132.0 (2CH), 124.0 (C), 114.0 (C), 51.2 (CH), 48.7 (CH), 36.3 (CH_2), 25.8 (CH_2), 24.8 (CH_2), 24.7 (CH_2), 23.58 (CH_2), 23.52 (CH_3), 22.2 (CH_2), 18.9 (CH_2).

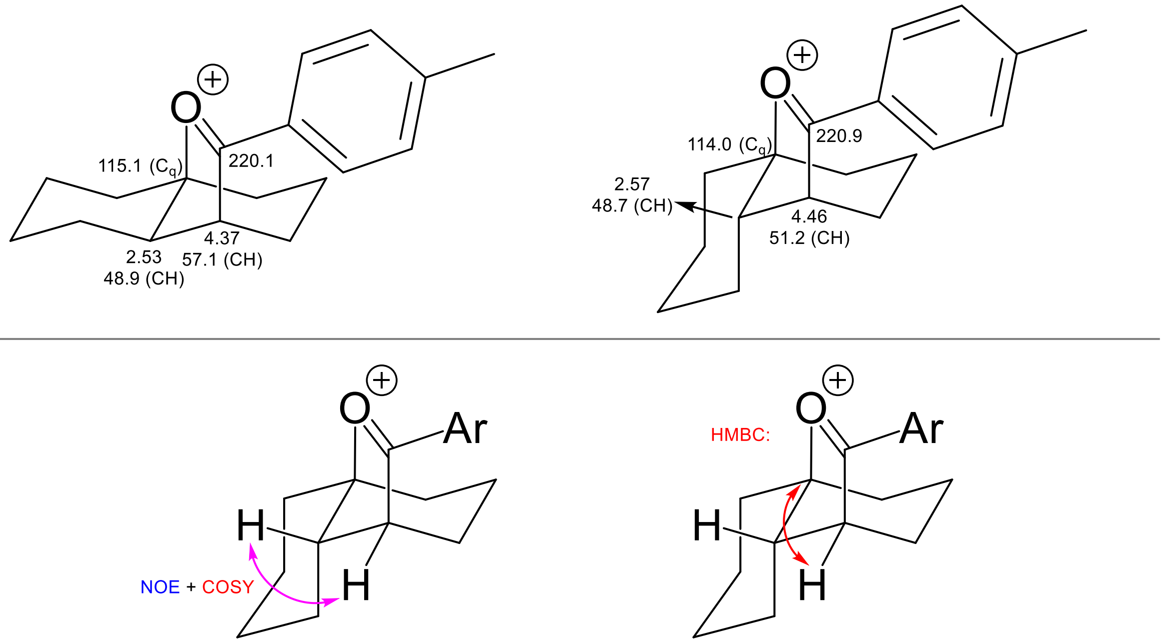

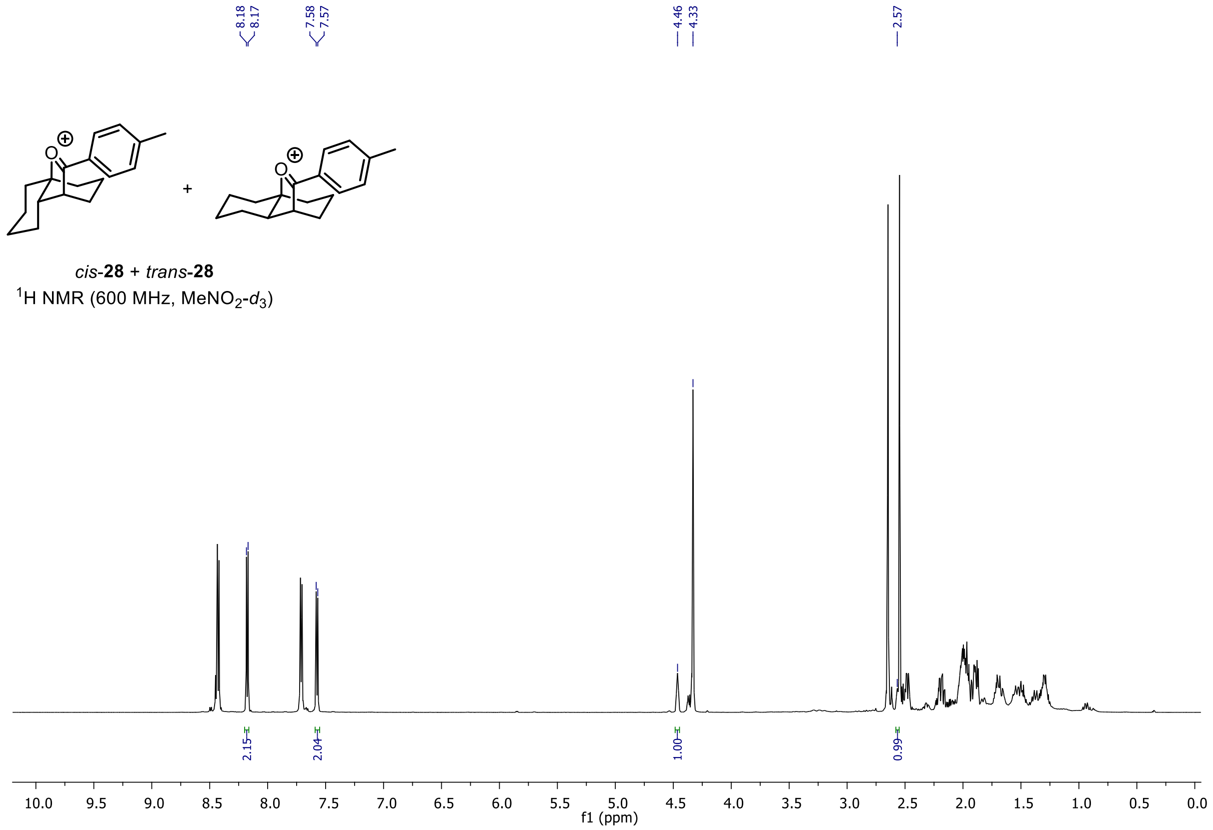

*^{1}H NMR spectrum of the reaction mixture, 30 minutes after reaction start. Integrated & peak-picked are key signals arising from the oxocarbenium ion cis-**28**. The signals of trans-**28** are overlapping with other aromatic species present inside the reaction mixture. Solvent signal is at 4.33 ppm.*

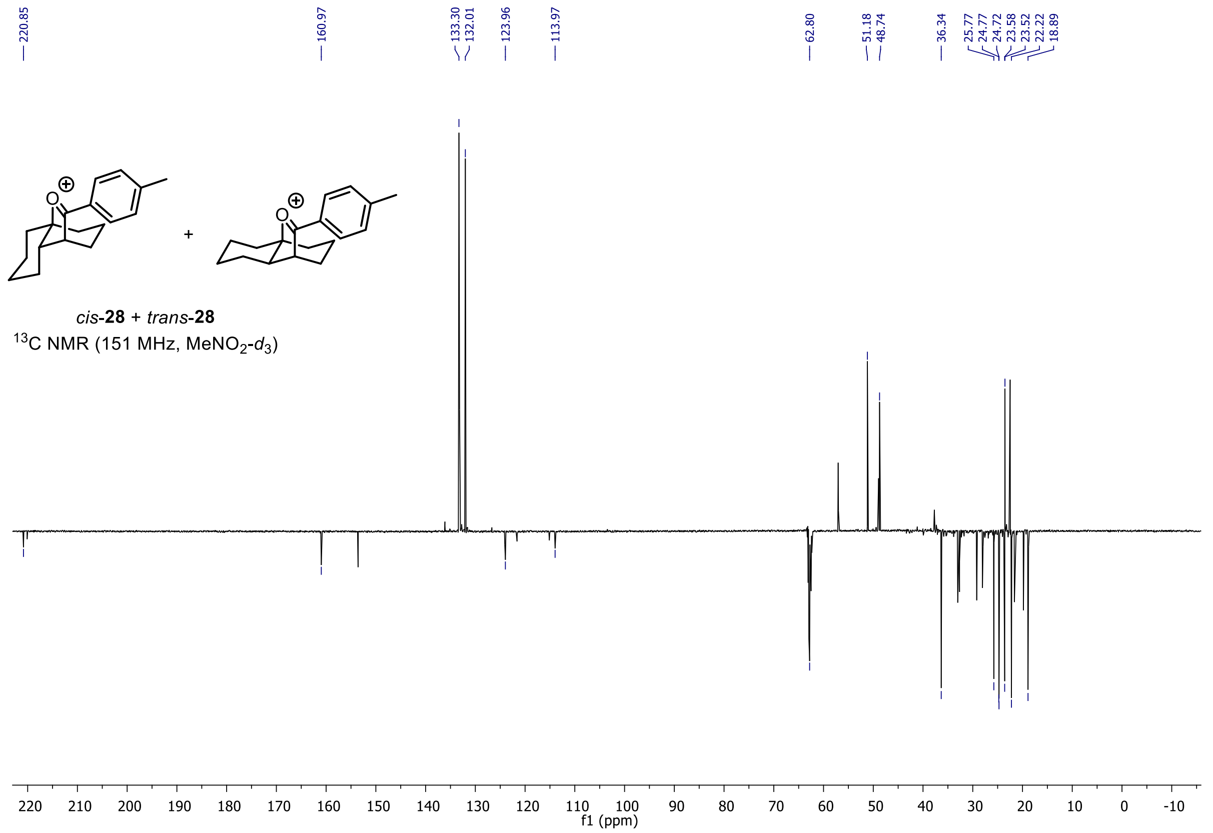

*^{13}C NMR spectrum of the reaction mixture, 30 minutes after reaction start. Peak-picked are signals arising from the oxocarbenium ion cis-**28**. Solvent signal is at 62.80 ppm.*

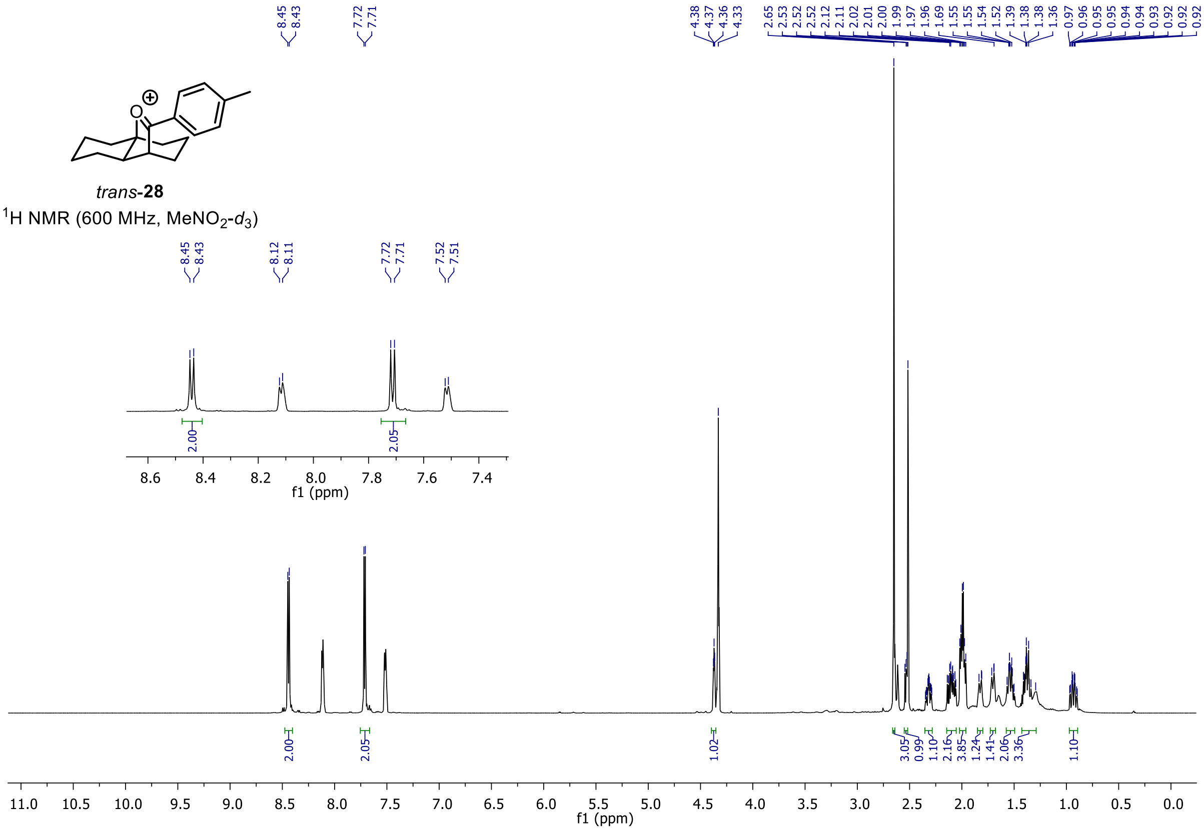

*^{1}H NMR spectrum of the reaction mixture, 64 hours after reaction start. Integrated & peak-picked are signals arising from the oxocarbenium ion trans-**28**. Additional aromatic compound is present (presumably from decomposition of unreacted acylium ion), note that this is not the cis isomer. Solvent signal is at 4.33 ppm.*

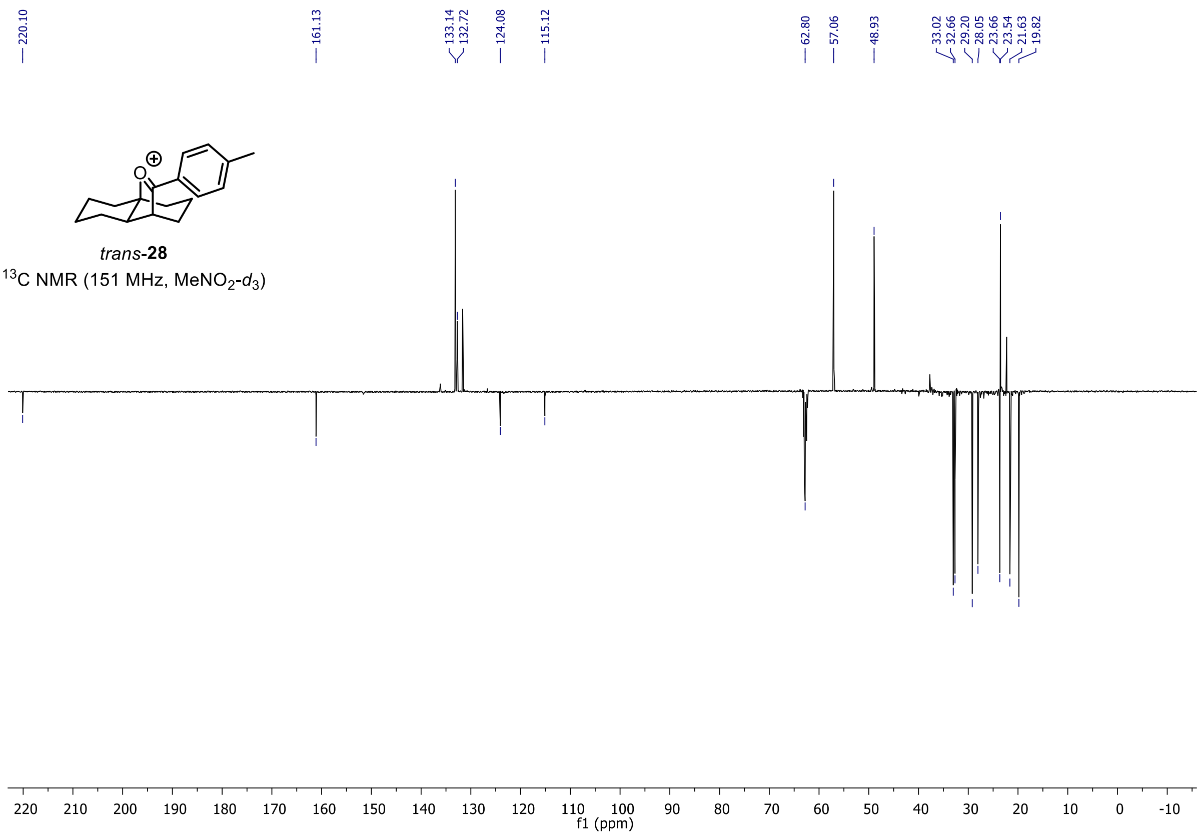

*^{13}C NMR spectrum of the reaction mixture, 64 hours after reaction start. Integrated & peak-picked are signals arising from the oxocarbenium ion trans-**28**. Additional aromatic compound is present (presumably from decomposition of unreacted acylium ion), note that this is not the cis isomer. Solvent signal is at 62.80 ppm.*

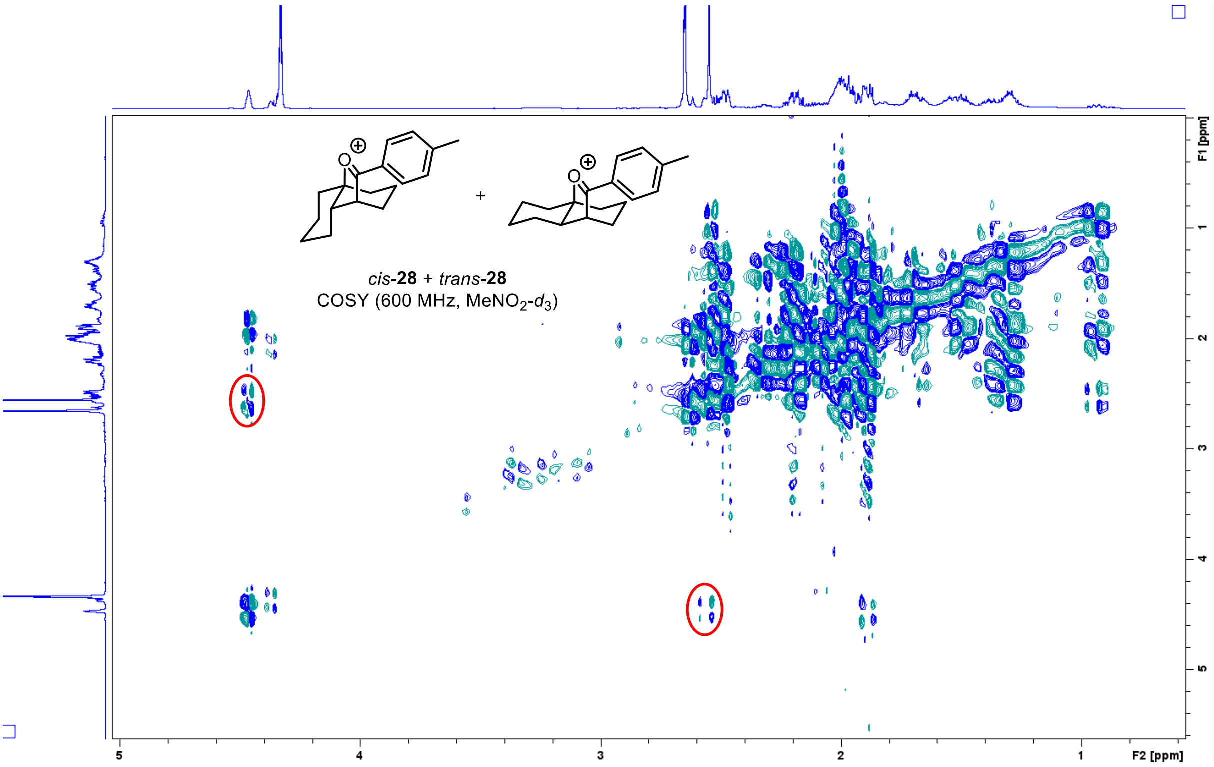

COSY spectrum of the reaction mixture, 30 minutes after reaction start. Highlighted are crosspeaks between 4.46 ppm + 2.57 ppm.

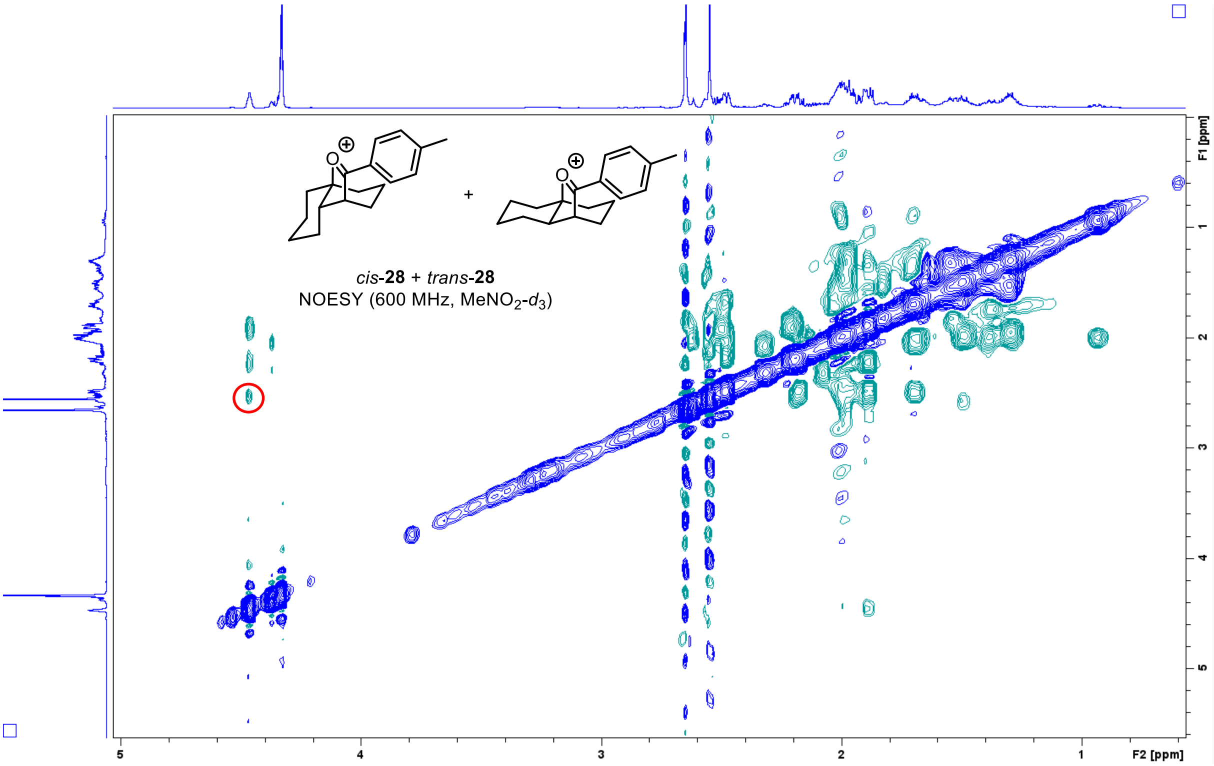

NOESY spectrum of the reaction mixture, 30 minutes after reaction start. Highlighted is the opposite-phase crosspeak (= positive NOE) between 4.46 ppm + 2.57 ppm.

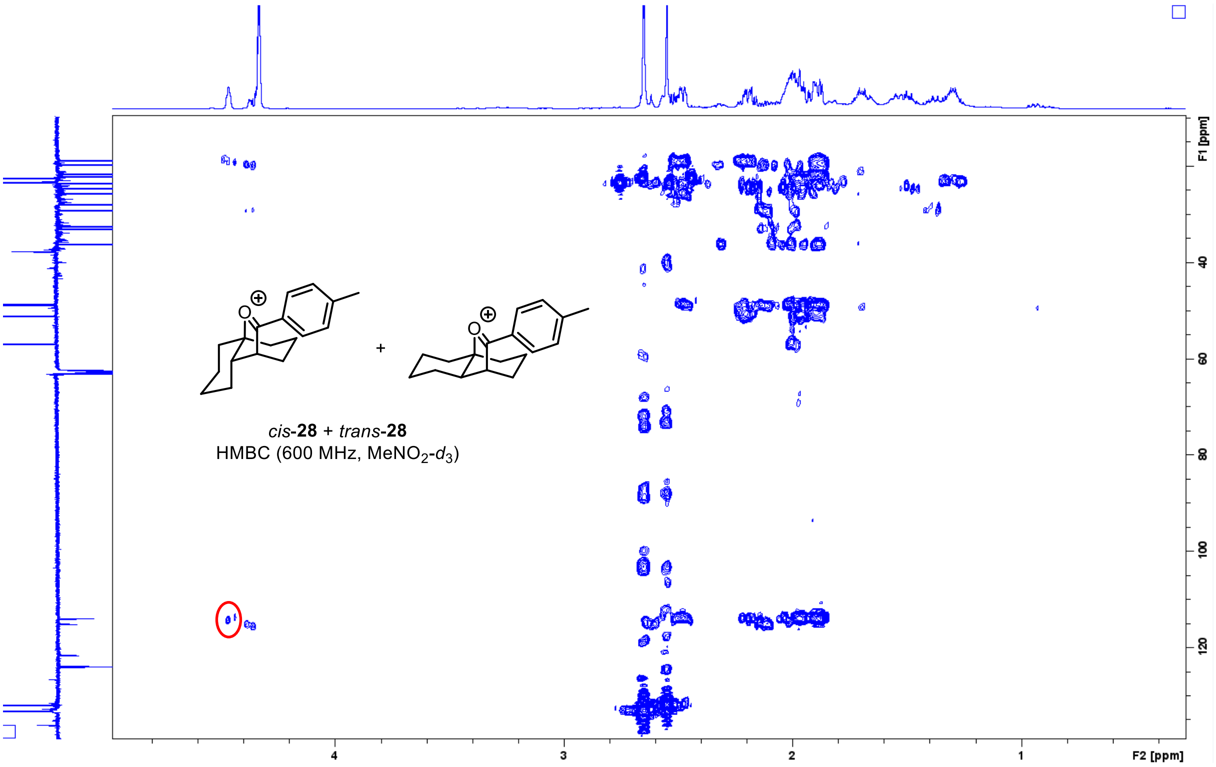

HMBC spectrum of the reaction mixture, 30 minutes after reaction start. Highlighted is the correlation between 114.0 ppm + 4.46 ppm ($^{3}J_{C\text{–}H}$).

NMR data of *cis*-**12** (^{a} and ^{b} denote signals arising from the 2 conformers *cis*-**12a** and *trans*-**12b** respectively, undesignated signals arise from both)**:**

^{1}H NMR (700 MHz, $CDCl_3$, 238 K) δ 7.91 (d, *J* = 8.1 Hz, 1.30H^{b}), 7.84 (d, *J* = 7.9 Hz, 0.70H^{a}), 7.32 – 7.24 (m, 2H), 5.98 (d, *J* = 4.5 Hz, 0.33H^{a}), 3.73 (td, *J* = 11.9, 3.2 Hz, 0.65H^{b}), 3.47 (d, *J* = 6.7 Hz, 0.35H^{a}), 2.41 (d, *J* = 3.6 Hz, 3H), 2.14 (br, 0.27H$^{b,\ \text{acidic OH}}$), 2.11 – 2.05 (m, 0.65H^{b}), 1.96 – 1.44 (m, 11.36H), 1.36 (d, *J* = 12.9 Hz, 2H), 1.29 – 1.15 (m, 2.50H).

^{13}C NMR (176 MHz, $CDCl_3$, 238 K) δ 206.6 (C^{a}), 203.4 (C^{b}), 144.5 (C^{a}), 144.4 (C^{b}), 134.1 (C^{b}), 132.6 (C^{a}), 129.54 (2CH^{b}), 129.49 (2CH^{a}), 129.0 (2CH^{a}), 128.4 (2CH^{b}), 71.5 (C^{b}), 69.5 (C^{a}), 47.9 (CH^{a}), 44.0 (CH^{b}), 43.5 (CH^{b}), 41.8 (CH^{a}), 41.4 (CH_2^{b}), 41.1 (CH_2^{a}), 31.8 (CH_2^{a}), 31.3 (CH_2^{b}), 31.2 (CH_2^{b}), 31.0 (CH_2^{a}), 26.2 (CH_2^{a}), 23.9 (CH_2^{a}), 23.7 (CH_2^{b}), 23.4 (CH_2^{b}), 23.3 (CH_2^{a}), 21.9 (CH_3), 21.3 (CH_2^{b}), 20.4 (CH_2^{b}), 17.9 (CH_2^{a}).

core decalin signals are broad (^{1}H NMR) and broad/missing (^{13}C NMR) at 298 K
the signals reported here were obtained at 238 K

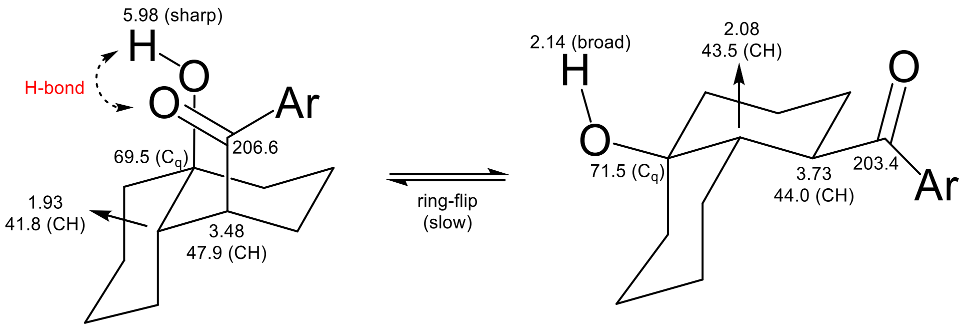

cis-**12a**, **conformer 1** *cis*-**12b**, **conformer 2**

ratio **a**:**b** at 238 K = 1:1.9

---

same-phase NOESY crosspeaks (EXSY)

highlighted are easily distinguishable protons

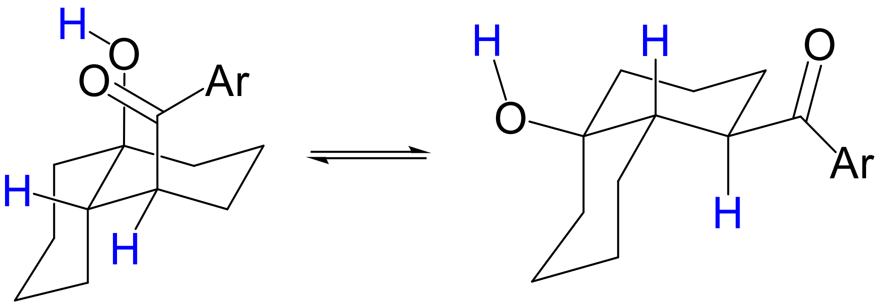

---

opposite-phase NOESY crosspeaks (positive NOEs)

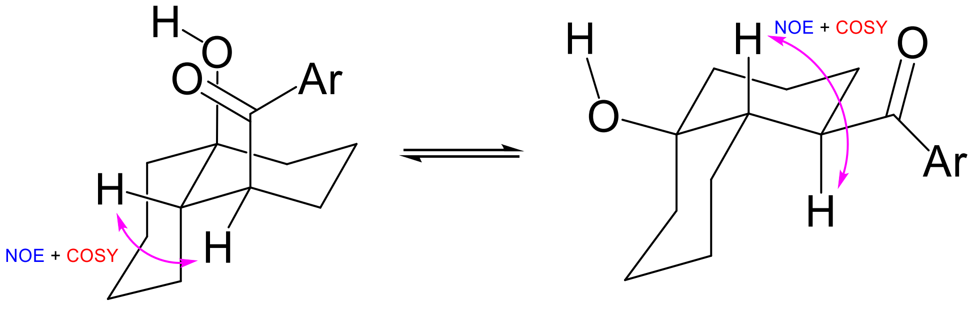

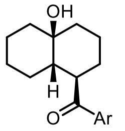

cis-**12**

^{1}H NMR (700 MHz, $CDCl_3$, **298 K**)

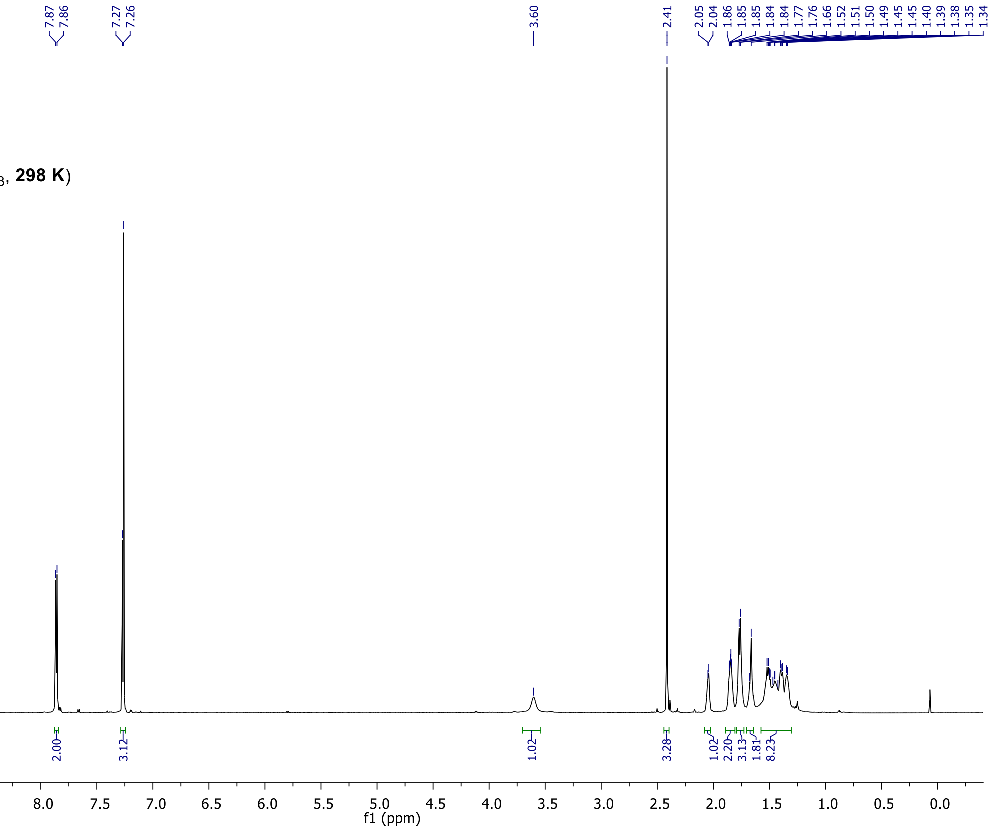

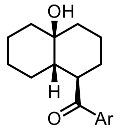

cis-**12**, 2 conformers

^{1}H NMR (700 MHz, $CDCl_3$, **238 K**)

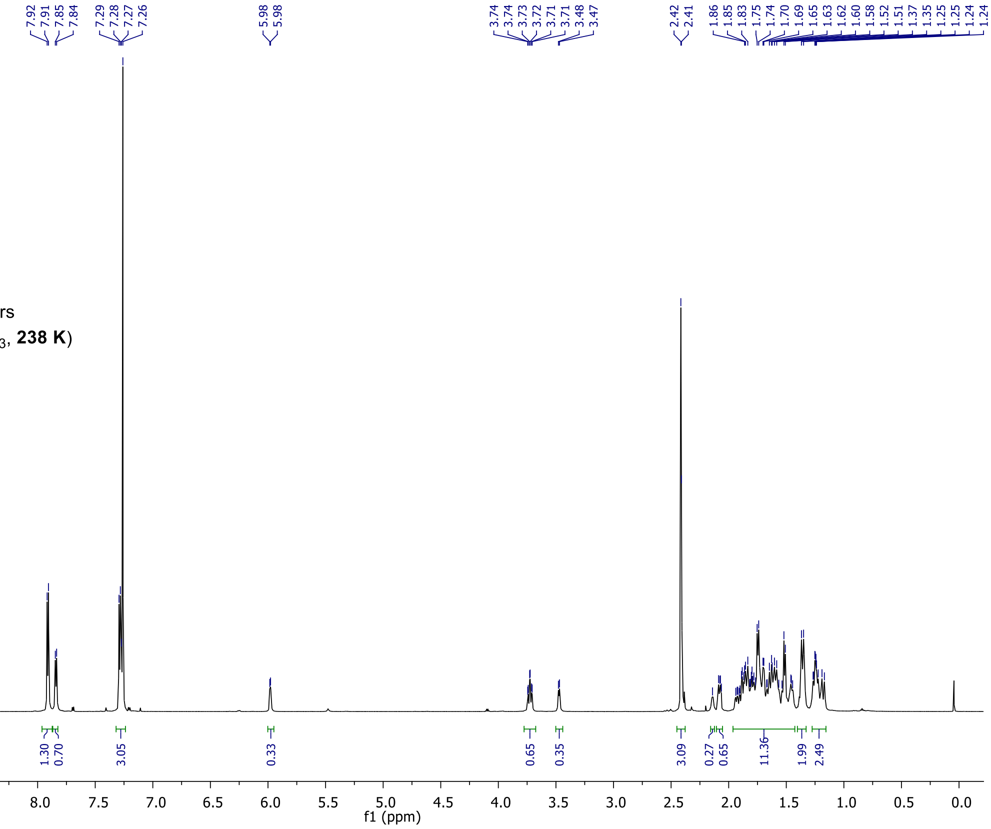

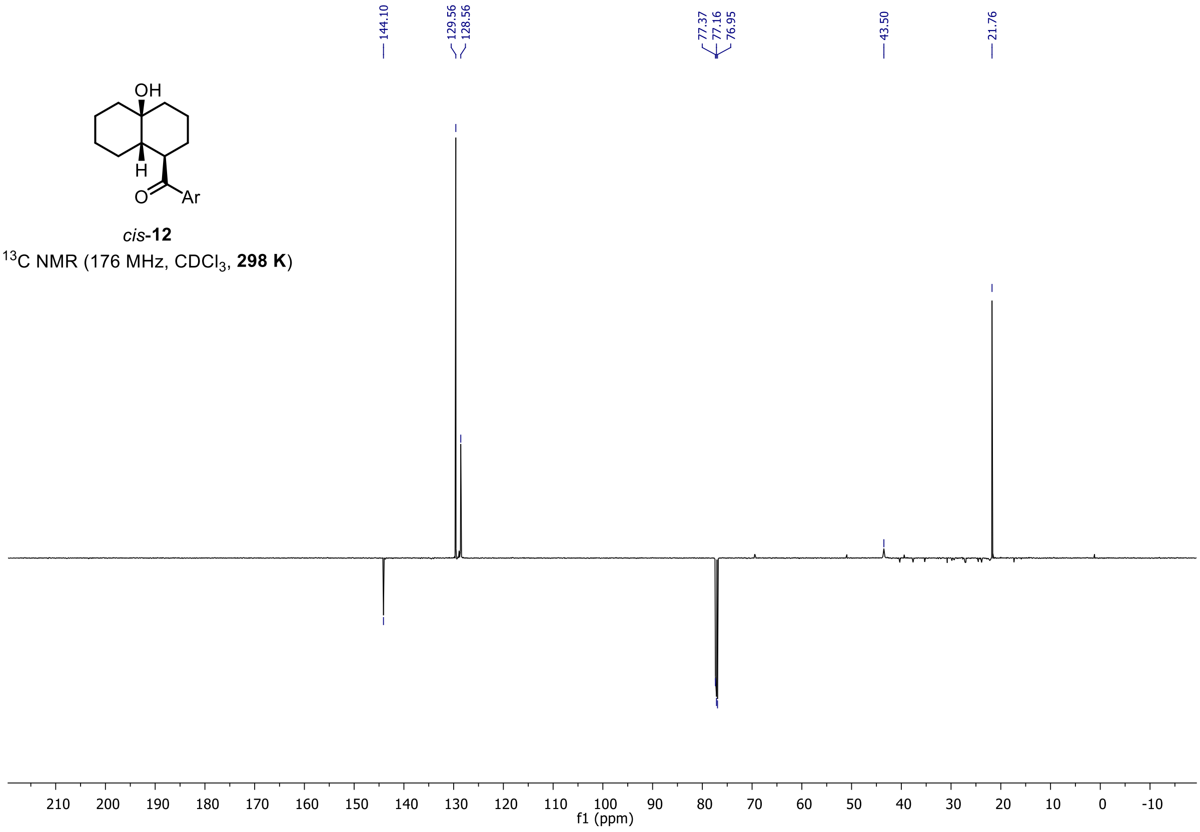

cis-**12**

^{13}C NMR (176 MHz, $CDCl_3$, **298 K**)

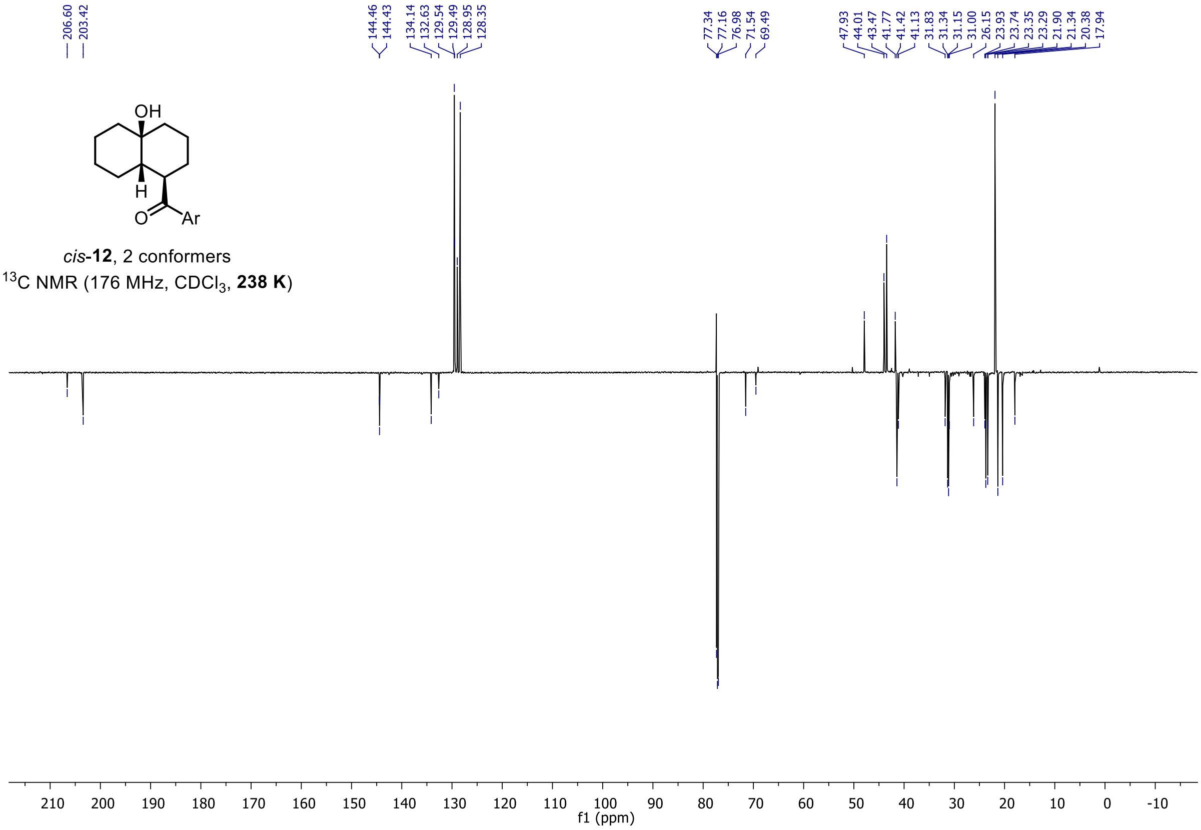

cis-**12**, 2 conformers

^{13}C NMR (176 MHz, $CDCl_3$, **238 K**)

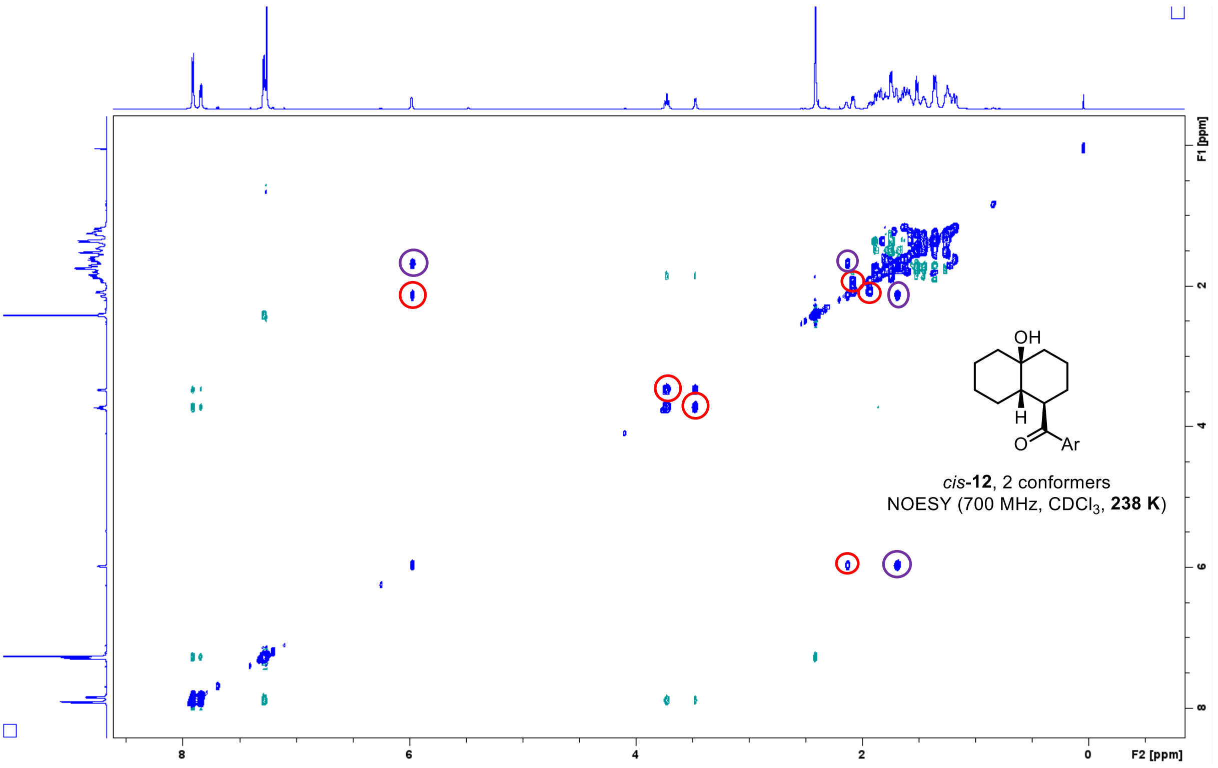

Highlighted in: *Red: same-phase crosspeaks between 5.98 ppm + 2.14 ppm (OH), 2.08 ppm + 1.93 ppm (CH) and 3.73 + 3.48 ppm ($CH_{\alpha\text{-}CO}$). These are indicative of an exchange process between the two species. Purple: same-phase crosspeaks between water and the acidic OH group at 5.98 ppm or 2.14 ppm.*

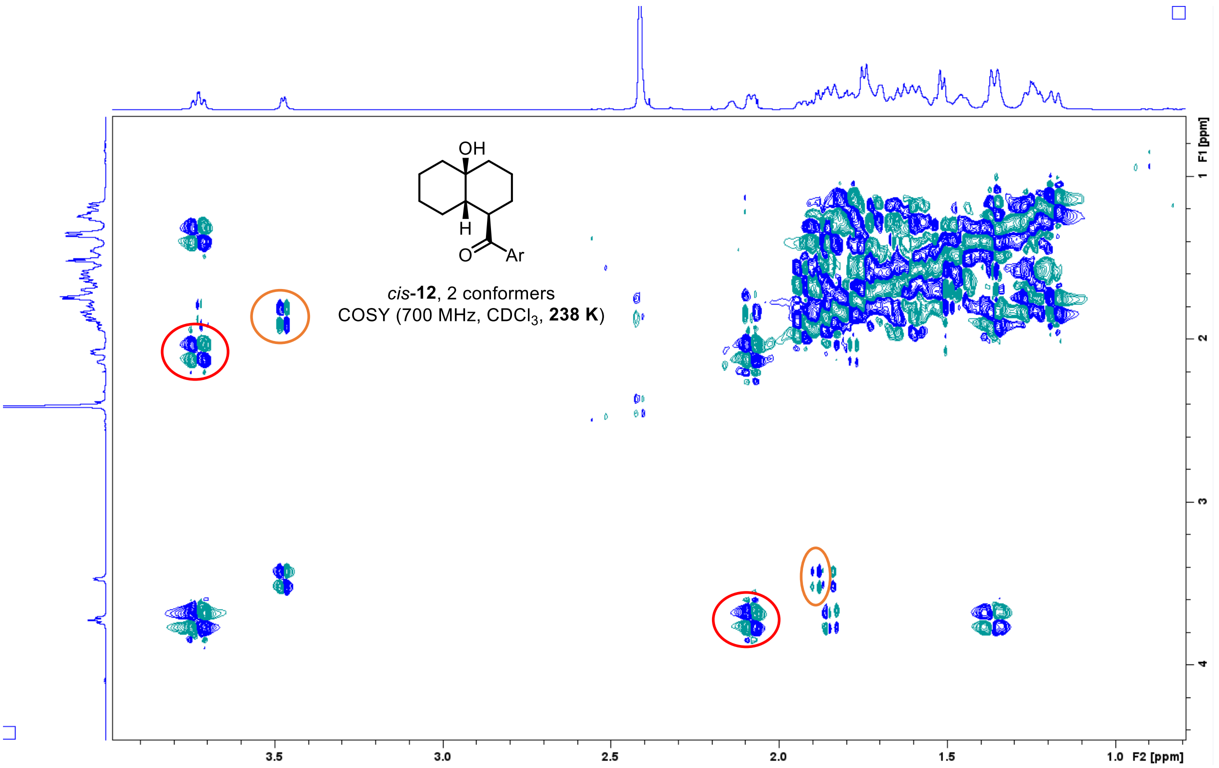

Highlighted in: *Red: crosspeaks between 3.48 ppm + 1.93 ppm. Orange: 3.73 + 2.08 ppm.*

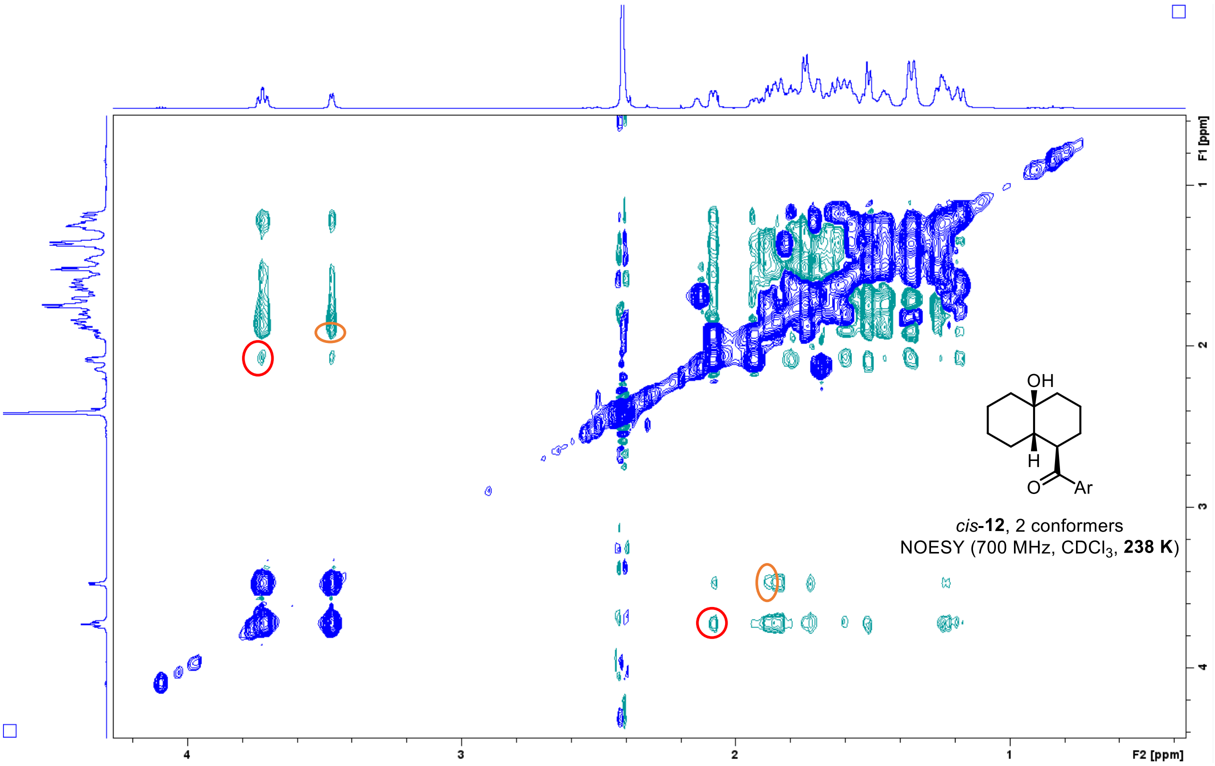

Highlighted in: *Red: opposite-phase crosspeaks (= positive NOE) between 3.73 + 2.08 ppm. Orange: opposite-phase crosspeaks between 3.48 ppm + 1.93 ppm.*

b) Octalin experiment #2

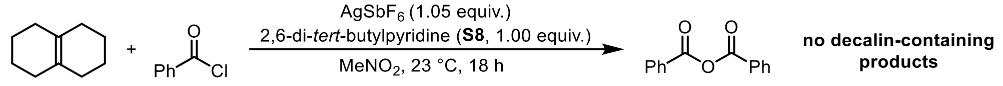

8 (1.00 equiv.) **S7** (1.00 equiv.) **S9**, ~40%

In a flame-dried vial (3 mL max. volume) with a rubber septum cap, $AgSbF_6$ (72 mg, 0.21 mmol, 1.05 equiv., weighed in glovebox) was dissolved in 1.0 mL $MeNO_2$ at ambient temperature (23 °C). Benzoyl chloride (28 mg, 0.20 mmol, 1.00 equiv.) was added under stirring at ambient temperature (23 °C), followed by 2,6-di-*tert*-butylpyridine (38 mg, 0.20 mmol, 1.00 equiv.) and $\Delta^{9,10}$-octalin (27 mg, 0.20 mmol, 1.00 equiv.). The resulting suspension was then stirred at ambient temperature (23 °C) for 18 h. Work up and extraction were performed in an identical way as outlined in **General Procedure A**. An analysis of the crude reaction product by ^{1}H NMR and ^{13}C NMR revealed the presence of anhydride **S9** (quantified using mesitylene as internal standard), with no decalin products. Traces of 2,6-di-*tert*-butylpyridine (**S8**) were detected as well (majority being likely removed upon drying in high vacuum).

The characteristic ^{1}H and ^{13}C NMR signals of **S8** and **S9** were in good accordance with those reported in literature.[40,41]

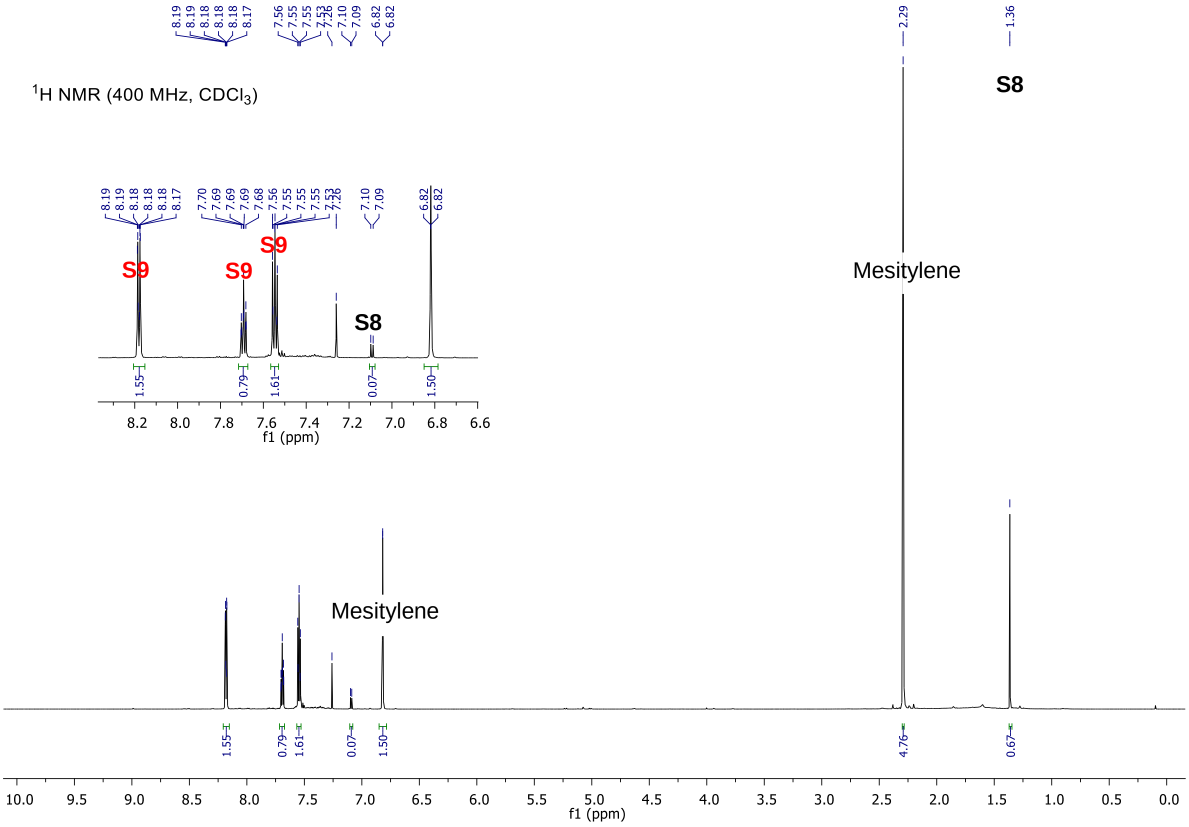

*Spectra of the crude reaction product with 0.1 mmol mesitylene as internal standard. Integrated & peak-picked are signals arising from mesitylene, anhydride **S9** (~40%) and 2,6-di-tert-butylpyridine **S8** (~3.5%).*

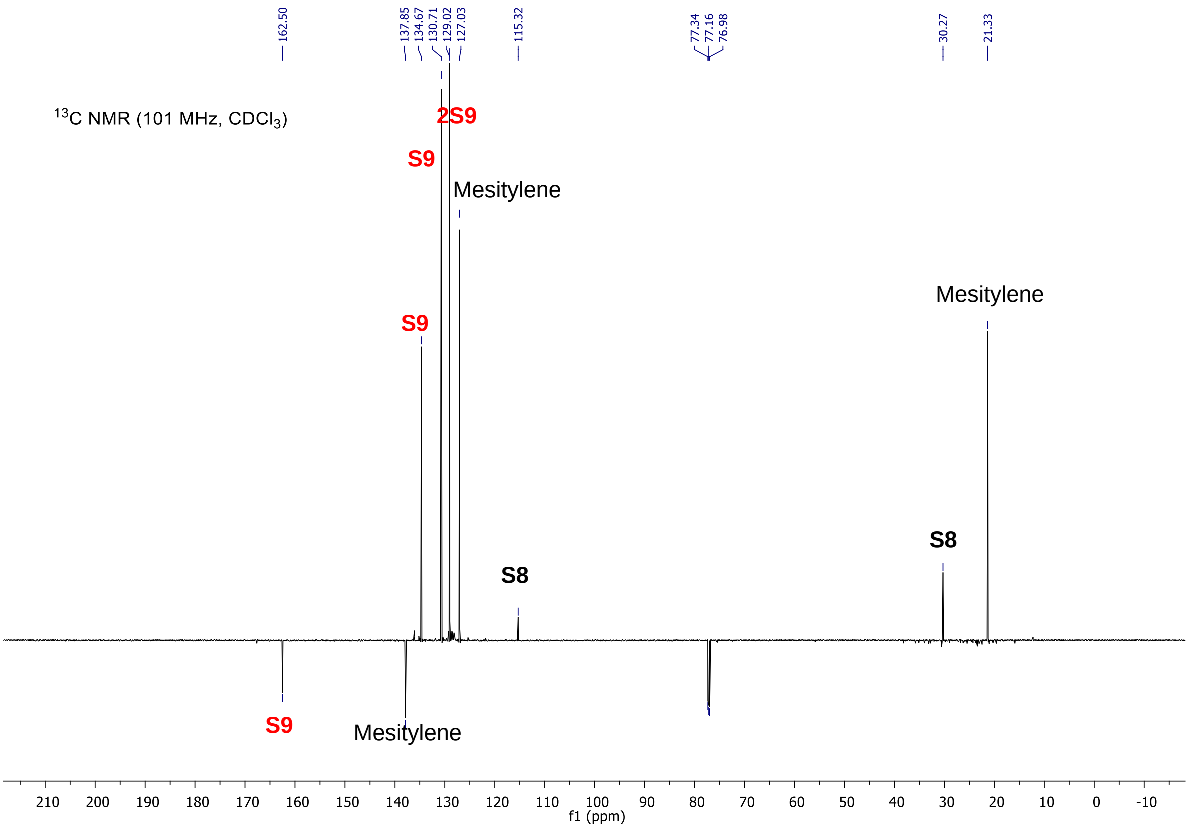

c) Aldehyde Presence

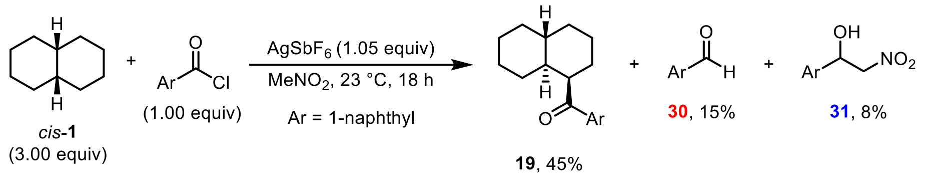

The reaction was performed following **General Procedure A** using 1-naphthoyl chloride (38 mg, 0.20 mmol, 1.00 equiv.), *cis*-decalin (93 µL, 0.60 mmol, 3.00 equiv.) and $AgSbF_6$ (72 mg, 0.21 mmol, 1.05 equiv.). An analysis of the crude reaction product by ^{1}H NMR and ^{13}C NMR showed the presence of **30** and **31** (key signals in good accordance with those reported in literature[42,43]), which were quantified using mesitylene as internal standard (^{1}H NMR).

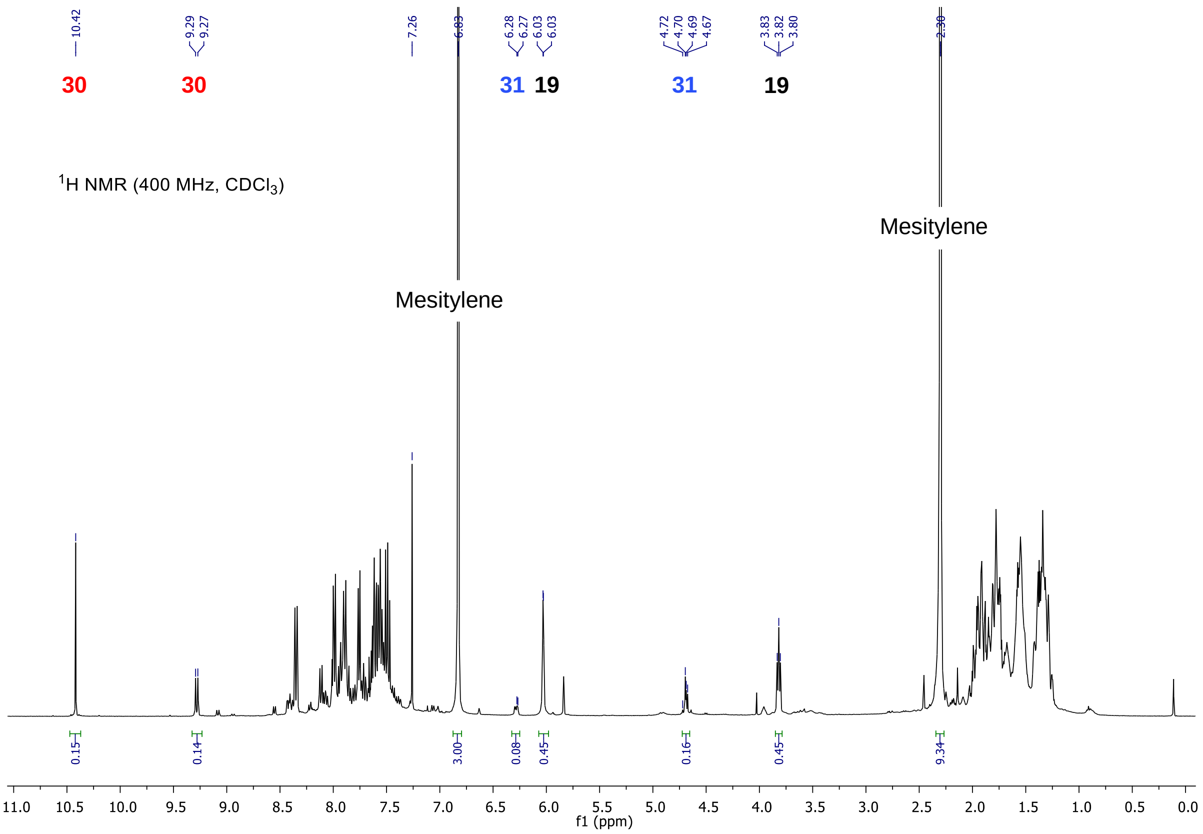

*^{1}H NMR spectrum of the crude reaction product with 0.1 mmol mesitylene as internal standard. Integrated are signals arising from mesitylene and the key signals arising from aldehyde **30** (15%), Henry product **31** (8%) and the keto alcohol **19** (45%).*

d) Crossover experiment

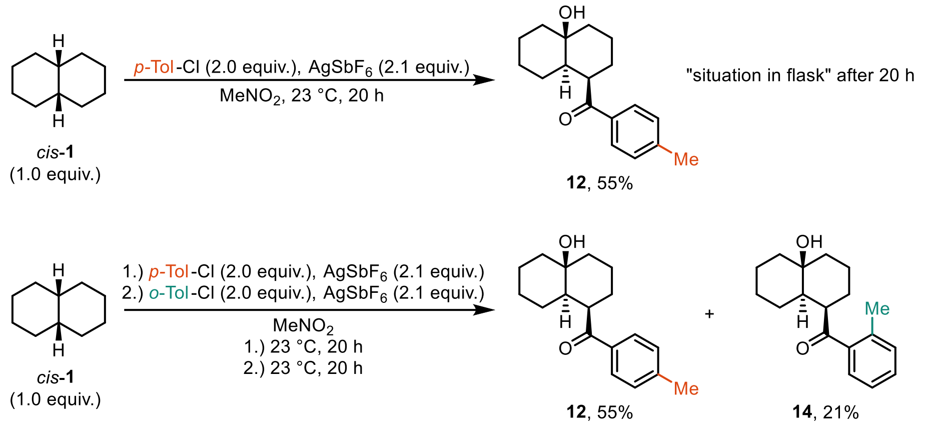

The first reaction was performed following **General Procedure A** using *p*-toluoyl chloride (26 µL, 0.20 mmol, 2.00 equiv.), *cis*-decalin (15 µL, 0.10 mmol, 1.00 equiv.) and $AgSbF_6$ (72 mg, 0.21 mmol, 2.10 equiv.). Yield of **12** was determined by ^{1}H NMR analysis of the crude reaction product using mesitylene as internal standard.

The second reaction was set up in parallel and performed as follows:

In a small flame-dried Schlenk tube with a rubber septum, $AgSbF_6$ (72 mg, 0.21 mmol, 2.10 equiv., weighed in glovebox) was dissolved in 0.5 mL $MeNO_2$ at ambient temperature (23 °C). *p*-Toluoyl chloride (26 µL, 0.20 mmol, 2.00 equiv.) was added under stirring at ambient temperature (23 °C), followed by *cis*-decalin (15 µL, 0.10 mmol, 1.00 equiv.). The resulting suspension was then stirred at ambient temperature (23 °C) for 20 hours, after which the flask was opened under positive Ar pressure and fresh $AgSbF_6$ (72 mg, 0.21 mmol, 2.10 equiv., weighed in glovebox) was added in one portion. The flask was immediately sealed with a septum and *o*-toluoyl chloride (26 µL, 0.20 mmol, 2.00 equiv.) was added. The suspension was then stirred at ambient temperature (23 °C) for 20 hours. Work up and extraction were performed in an identical way as outlined in **General Procedure A**. Yields of **12** and **14** were determined by ^{1}H NMR analysis of the crude reaction product using mesitylene as internal standard.

The characteristic ^{1}H NMR signals of **12** and **14** were in good accordance with those reported in Section **4.4** (isolated pure products).

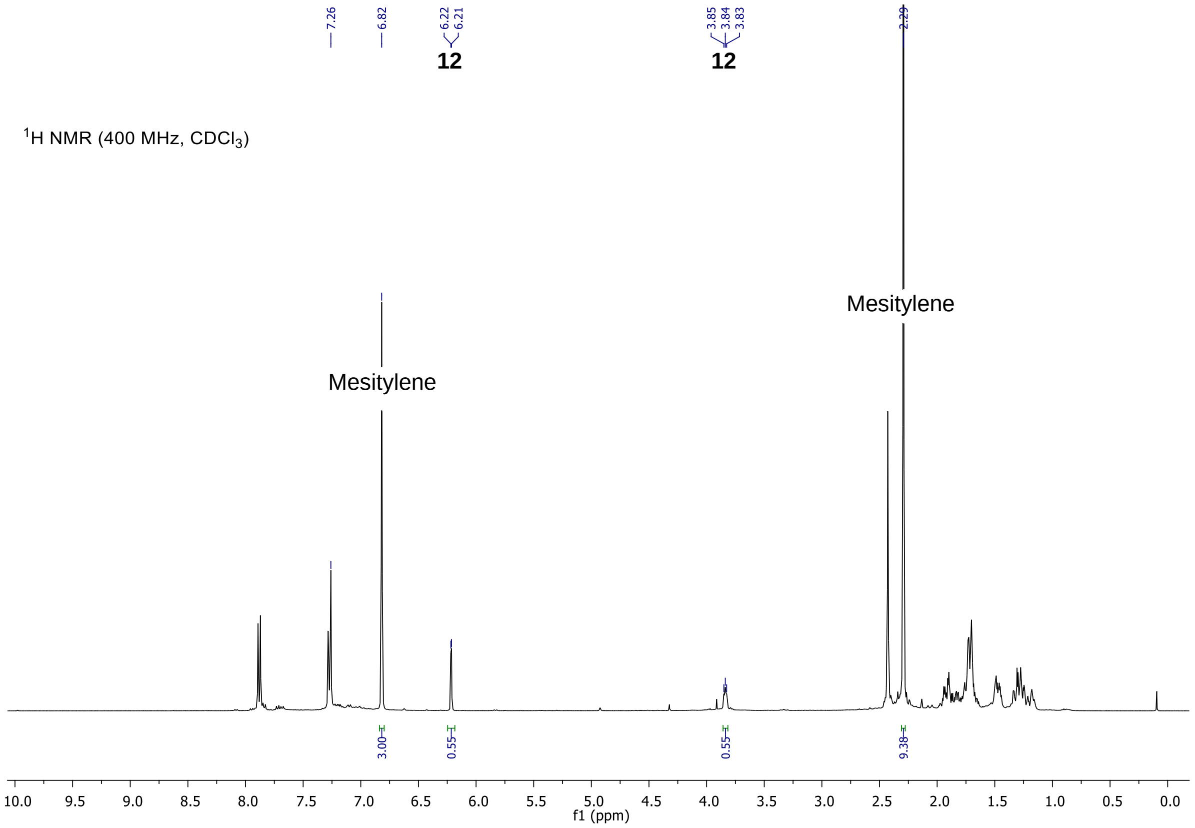

*^{1}H NMR spectrum of the crude reaction product from the <u>first reaction</u> with 0.1 mmol mesitylene as internal standard. Integrated are signals arising from mesitylene and the key signals arising from keto alcohol **12** (55%).*

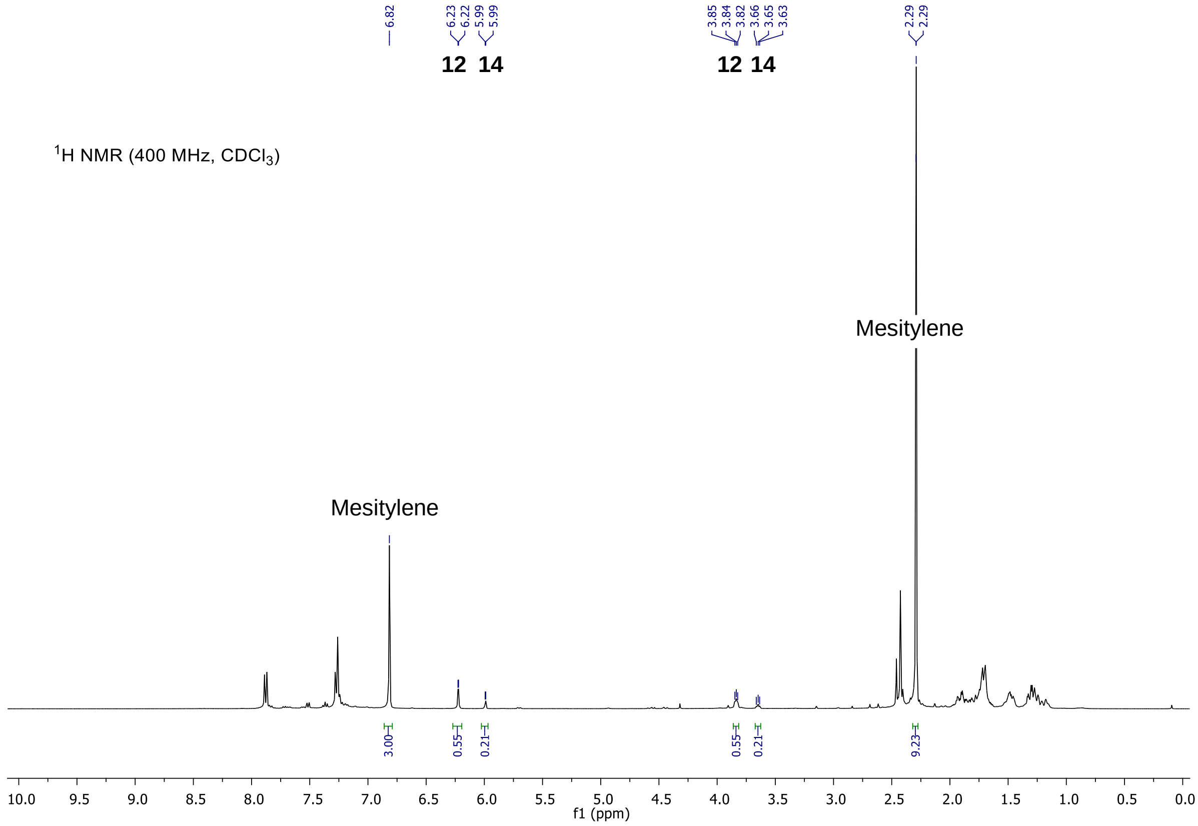

*^{1}H NMR spectrum of the crude reaction product from the <u>second reaction</u> with 0.1 mmol mesitylene as internal standard. Integrated are signals arising from mesitylene and the key signals arising from keto alcohols **12** (55%) and **14** (21%).*

e) Influence of 2,6-di-*tert*-butylpyridine on reaction with decalin

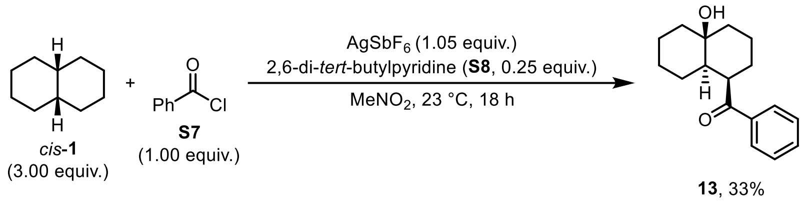

In a flame-dried vial (3 mL max. volume) with a rubber septum cap, $AgSbF_6$ (72 mg, 0.21 mmol, 1.05 equiv., weighed in glovebox) was dissolved in 0.5 mL $MeNO_2$ at ambient temperature (23 °C). Benzoyl chloride (28 mg, 0.20 mmol, 1.00 equiv.) was added under stirring at ambient temperature (23 °C), followed by 2,6-di-*tert*-butylpyridine (10 mg, 0.05 mmol, 0.25 equiv.) and *cis*-decalin (93 µL, 0.60 mmol, 3.00 equiv.). The resulting suspension was then stirred at ambient temperature (23 °C) for 18 h. Work up and extraction were performed in an identical way as outlined in **General Procedure A**. Yield of **13** was determined by ^{1}H NMR analysis of the crude reaction product using mesitylene as internal standard. Traces of 2,6-di-*tert*-butylpyridine (**S8**) were detected as well (majority being likely removed upon drying in high vacuum).

The characteristic ^{1}H NMR signals of **13** were in good accordance with those reported in Section **4.4** (isolated pure product). The characteristic ^{1}H NMR signals of **S8** were in good accordance with those reported in literature.[40]

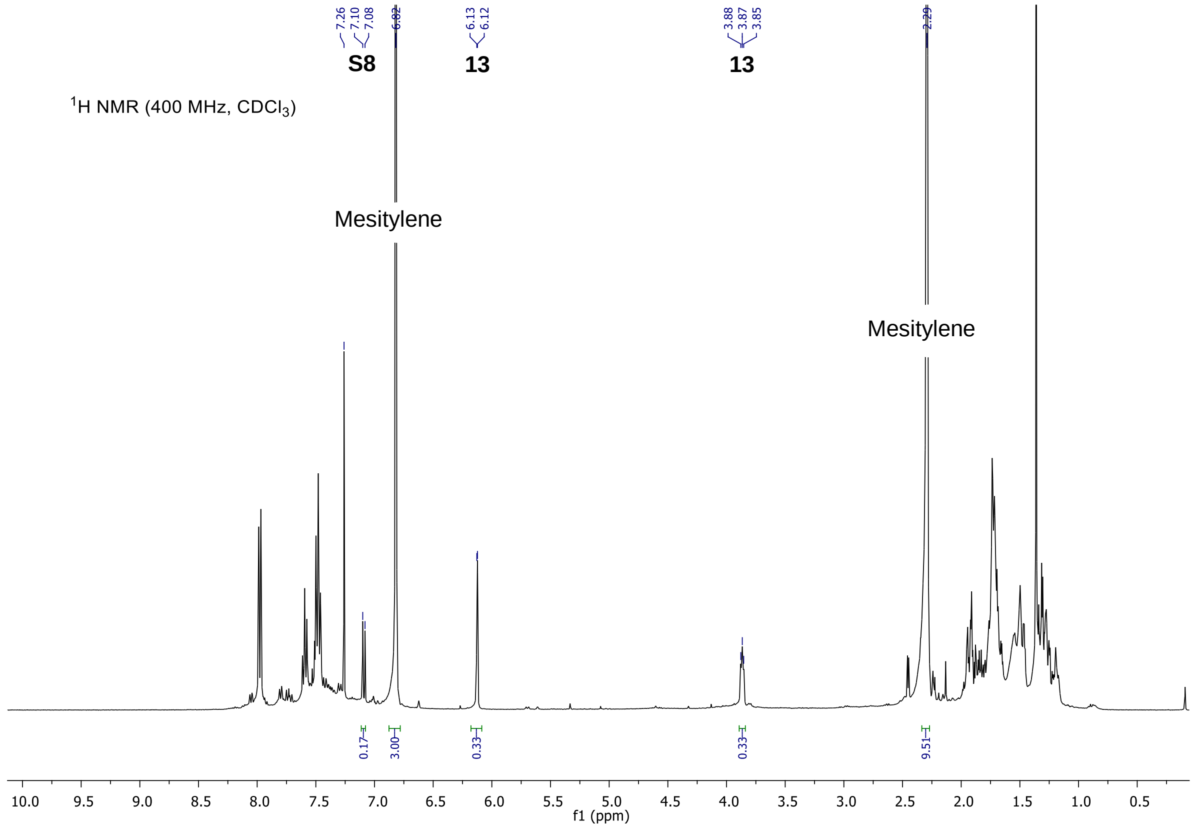

*^{1}H NMR spectrum of the crude reaction product with 0.1 mmol mesitylene as internal standard. Integrated are signals arising from mesitylene and the key signals arising from keto alcohol **13** (33%) and 2,6-di-tert-butylpyridine **S8** (~9%).*

4.4 Modified Baddeley reaction

General Procedure A: Modified Baddeley reaction for electron rich & electron neutral acyl chlorides

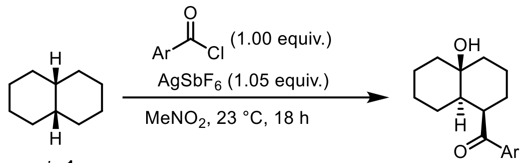

cis-**1**
(3.00 equiv.)

In a small flame-dried vial with a rubber septum cap, $AgSbF_6$ (72 mg, 0.21 mmol, 1.05 equiv., weighed in glovebox) was dissolved in 0.5 mL $MeNO_2$ at ambient temperature (23 °C). Acyl chloride (0.20 mmol, 1.00 equiv.) was added under stirring at ambient temperature (23 °C), followed by *cis*-decalin (0.60 mmol, 3.00 equiv.). The cap was sealed with multiple layers of parafilm[a] and the resulting suspension was then stirred at ambient temperature (23 °C) for 18 hours. The reaction was stopped by adding 10% aqueous K_2CO_3 solution (2 mL) and stirring for 1 h at ambient temperature (23 °C). The mixture was transferred to a separatory funnel, diluted with more 10% aqueous K_2CO_3 solution (5 mL) and the aqueous layer was extracted with DCM (3 × 5 mL). The combined organic layers were dried over anhydrous sodium sulfate, the dried solution was filtered, and the filtrate was concentrated under reduced pressure (final drying for >30 min at <1 mbar at 23 °C to remove unreacted decalin and $MeNO_2$). The crude residue was purified by flash column chromatography to afford the desired compound.

[a]corrosion of rubber septum caps was sometimes observed.

(4-hydroxydecahydronaphthalen-1-yl)(p-tolyl)methanone (12)

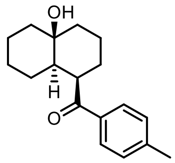

12

The title compound was prepared following **General Procedure A** using *p*-toluoyl chloride (26 µL, 0.20 mmol, 1.00 equiv.), *cis*-decalin (93 µL, 0.60 mmol, 3.00 equiv.) and $AgSbF_6$ (72 mg, 0.21 mmol, 1.05 equiv.). Purification by flash column chromatography (0 – 40% Et_2O in pentane) gave **12** as a yellow crystalline solid (18.9 mg, 70%).

A crystal of sufficient quality for X-ray diffractometry was obtained by slow evaporation of a solution in $CHCl_3$ (18 mg dissolved in approx. 2.0 mL $CHCl_3$ at 23 °C, vial loosely capped and stored at 23 °C until complete solvent evaporation).

R_f = 0.48 in 20% EtOAc/heptanes

m.p. (+5 °C/min) = 91.4 – 93.9 °C

^{1}H NMR (600 MHz, DMSO) δ 7.91 (d, *J* = 8.3 Hz, 2H), 7.34 (d, *J* = 8.0 Hz, 2H), 5.73 (d, *J* = 2.0 Hz, 1H), 3.98 (t, *J* = 5.6 Hz, 1H), 2.38 (s, 3H), 1.85 – 1.67 (m, 4H), 1.62 (br d, *J* = 11.5 Hz, 1H), 1.52 – 1.34 (m, 6H), 1.31 – 1.13 (m, 5H).

^{13}C NMR (151 MHz, DMSO) δ 206.4 (C), 144.4 (C), 133.7 (C), 129.5 (2CH), 128.9 (2CH), 67.5 (C), 44.70 (CH), 44.68 (CH), 40.2 (CH_2), 39.5 (CH_2), 29.2 (CH_2), 26.6 (CH_2), 25.9 (CH_2), 21.3 (CH_2), 21.2 (CH_3), 17.1 (CH_2).

IR (neat) ν_{max}: 3382, 2928, 2855, 1658, 1606, 1447, 1225, 1207, 960, 738.

HRMS (ESI$^+$): exact mass calculated for $[M+Na]^+$ ($C_{18}H_{24}O_2Na$) requires *m*/*z* 295.1674, found *m*/*z* 295.1668.

SC-XRD: See Section **5**.

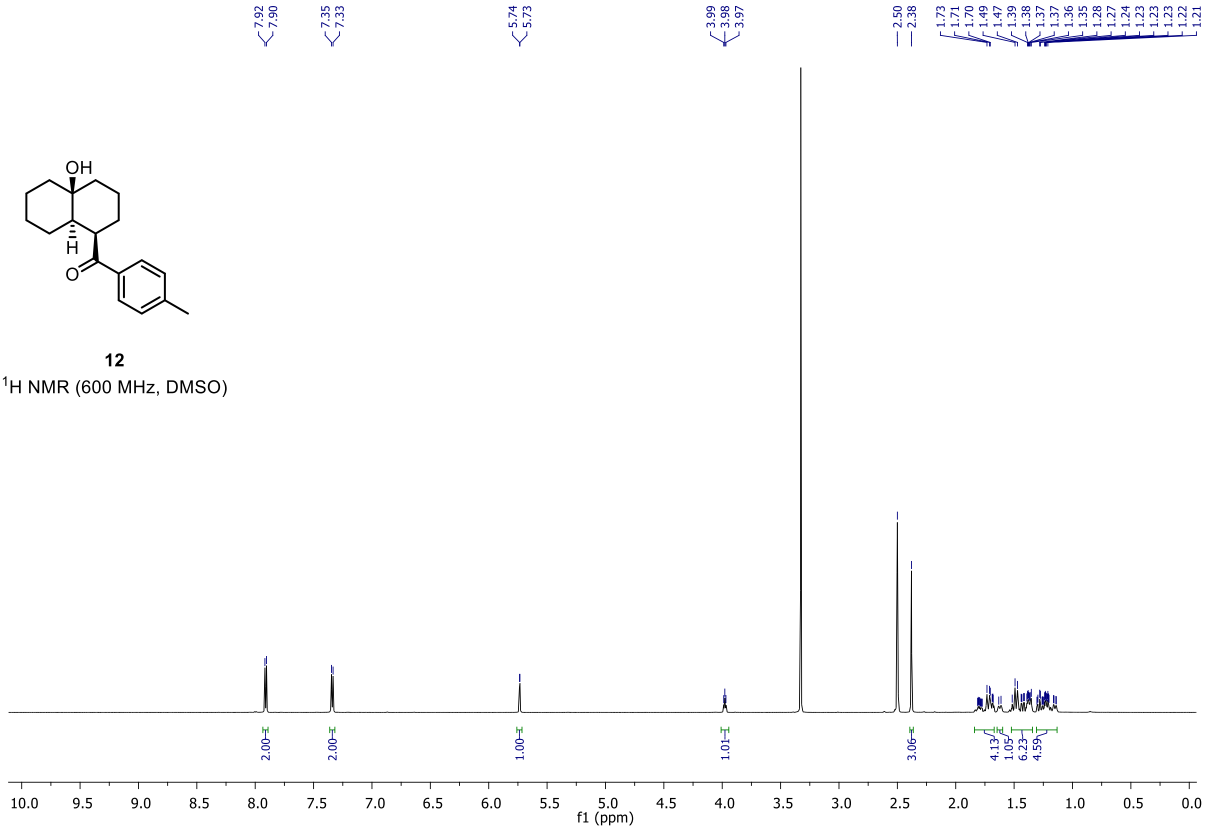

12

^{1}H NMR (600 MHz, DMSO)

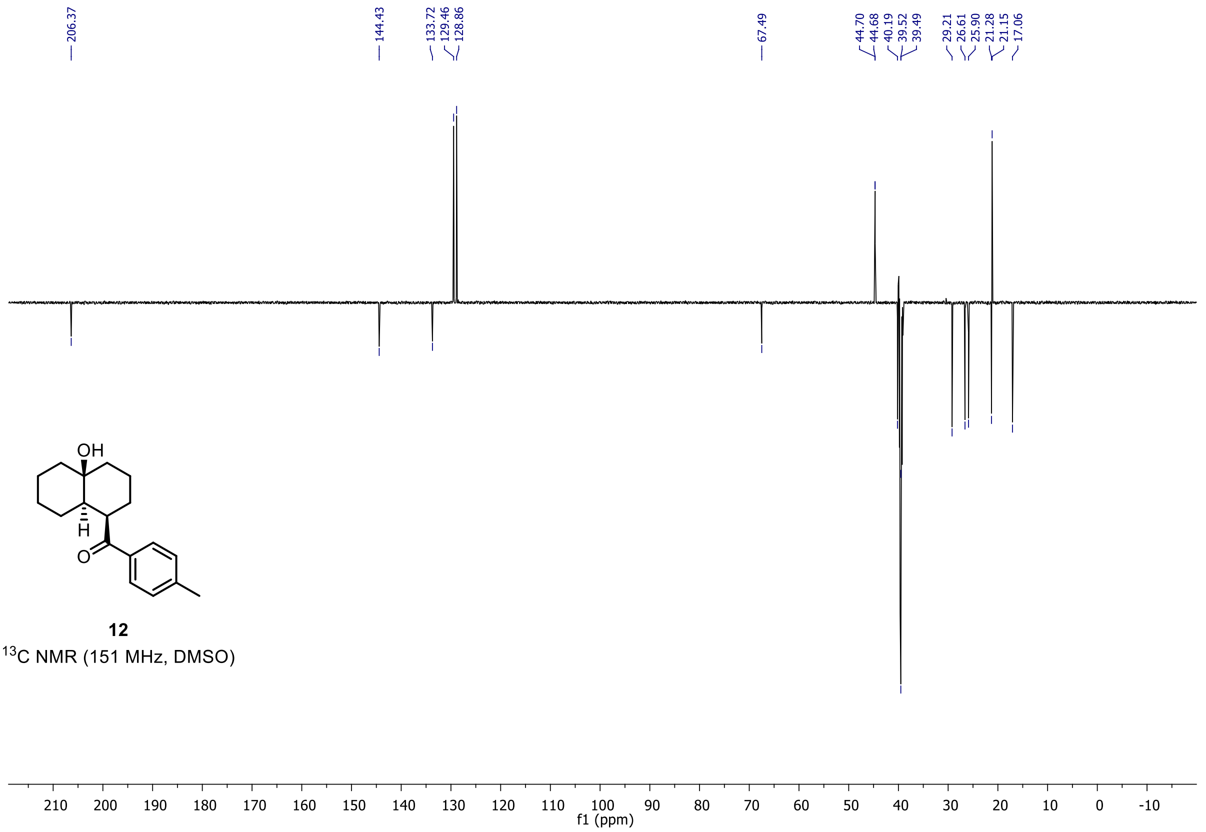

12

^{13}C NMR (151 MHz, DMSO)

(4-hydroxydecahydronaphthalen-1-yl)(phenyl)methanone (13)

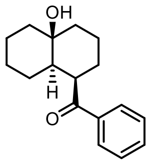

13

The title compound was prepared following **General Procedure A** using benzoyl chloride (23 µL, 0.20 mmol, 1.00 equiv.), *cis*-decalin (93 µL, 0.60 mmol, 3.00 equiv.) and $AgSbF_6$ (72 mg, 0.21 mmol, 1.05 equiv.). Purification by flash column chromatography (0 – 30% Et_2O in pentane) gave **13** as a light-yellow crystalline solid (12.9 mg, 50%).

R_f = 0.46 in 20% EtOAc/heptanes

m.p. (+5 °C/min) = 68.5 – 71.6 °C

^{1}H NMR (400 MHz, $CDCl_3$) δ 7.96 (dd, *J* = 5.2, 3.3 Hz, 2H), 7.62 – 7.55 (m, 1H), 7.52 – 7.43 (m, 2H), 6.09 (d, *J* = 2.3 Hz, 1H), 3.88 – 3.82 (m, 1H), 1.98 – 1.65 (m, 9H), 1.46 (ddd, *J* = 9.9, 6.2, 2.9 Hz, 2H), 1.33 – 1.15 (m, 4H).

^{13}C NMR (101 MHz, $CDCl_3$) δ 206.1 (C), 136.7 (C), 133.6 (2CH), 128.9 (3CH), 68.2 (C), 46.1 (CH), 45.8 (CH), 40.8 (CH_2), 40.2 (CH_2), 29.9 (CH_2), 27.4 (CH_2), 26.6 (CH_2), 21.8 (CH_2), 17.5 (CH_2).

IR (neat) v_{max}: 3388, 2926, 2854, 1659, 1447, 1217, 1102, 987, 959, 711.

HRMS (ESI$^+$): exact mass calculated for $[M+Na]^+$ ($C_{17}H_{22}O_2Na$) requires *m*/*z* 281.1512, found *m*/*z* 281.1508.

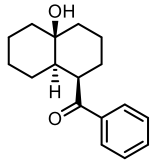

13

^1H NMR (400 MHz, $CDCl_3$)

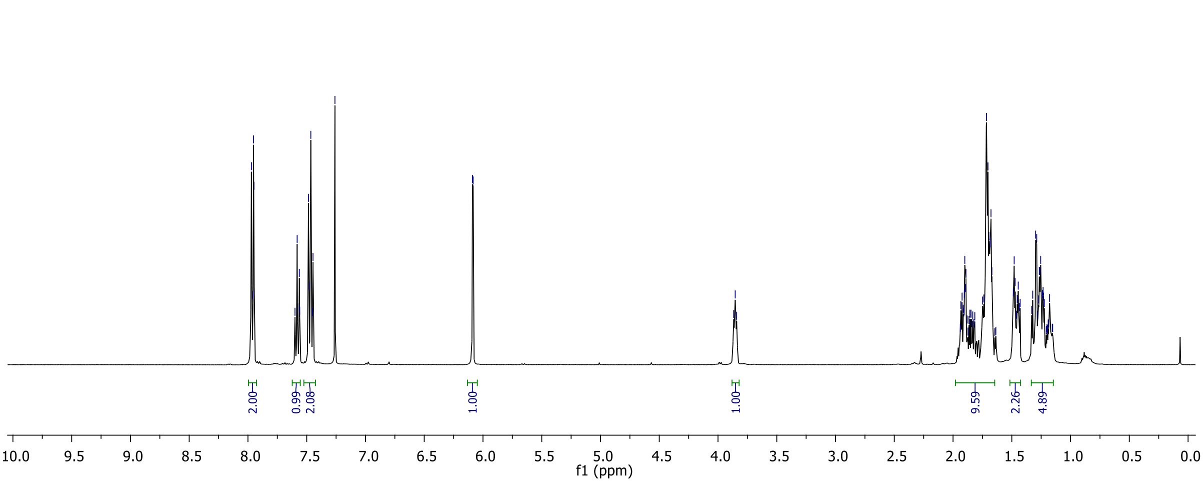

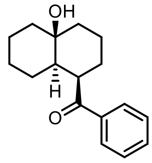

13

^{13}C NMR (101 MHz, $CDCl_3$)

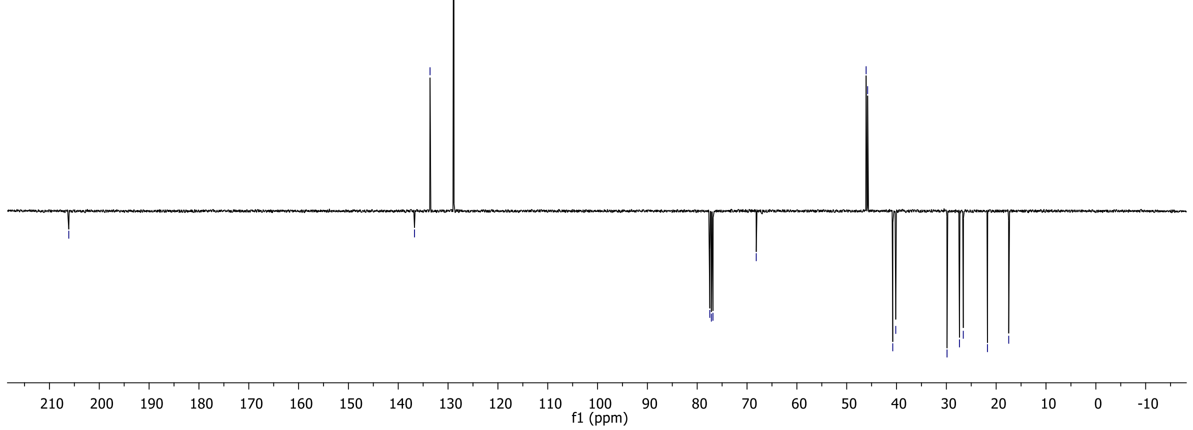

(4-hydroxydecahydronaphthalen-1-yl)(o-tolyl)methanone (14)

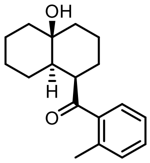

14

The title compound was prepared following **General Procedure A** using *o*-toluoyl chloride (26 µL, 0.20 mmol, 1.00 equiv.), *cis*-decalin (93 µL, 0.60 mmol, 3.00 equiv.) and $AgSbF_6$ (72 mg, 0.21 mmol, 1.05 equiv.). Purification by flash column chromatography (0 – 20% Et_2O in pentane) gave **14** as a yellow oil (17.0 mg, 62%).

R_f = 0.47 in 20% EtOAc/heptanes

^{1}H NMR (400 MHz, $CDCl_3$) δ 7.50 (d, *J* = 7.8 Hz, 1H), 7.40 – 7.33 (m, 1H), 7.23 (t, *J* = 8.1 Hz, 2H), 5.95 (d, *J* = 2.2 Hz, 1H), 3.64 (dd, *J* = 8.5, 3.3 Hz, 1H), 2.44 (s, 3H), 1.97 – 1.64 (m, 9H), 1.54 – 1.44 (m, 2H), 1.35 – 1.18 (m, 4H).

^{13}C NMR (101 MHz, $CDCl_3$) δ 211.2 (C), 139.0 (C), 138.1 (C), 134.0 (CH), 131.3 (CH), 128.1 (CH), 125.6 (CH), 68.3 (C), 50.0 (CH), 46.0 (CH), 40.7 (CH_2), 40.1 (CH_2), 28.9 (CH_2), 27.4 (CH_2), 26.7 (CH_2), 21.7 (CH_2), 21.0 (CH_3), 17.5 (CH_2).

IR (neat) v_{max}: 3394, 2926, 2854, 1663, 1447, 1218, 1102, 986, 958, 736.

HRMS (ESI$^+$): exact mass calculated for $[M+Na]^+$ ($C_{18}H_{24}O_2Na$) requires *m*/*z* 295.1669, found *m*/*z* 295.1671.

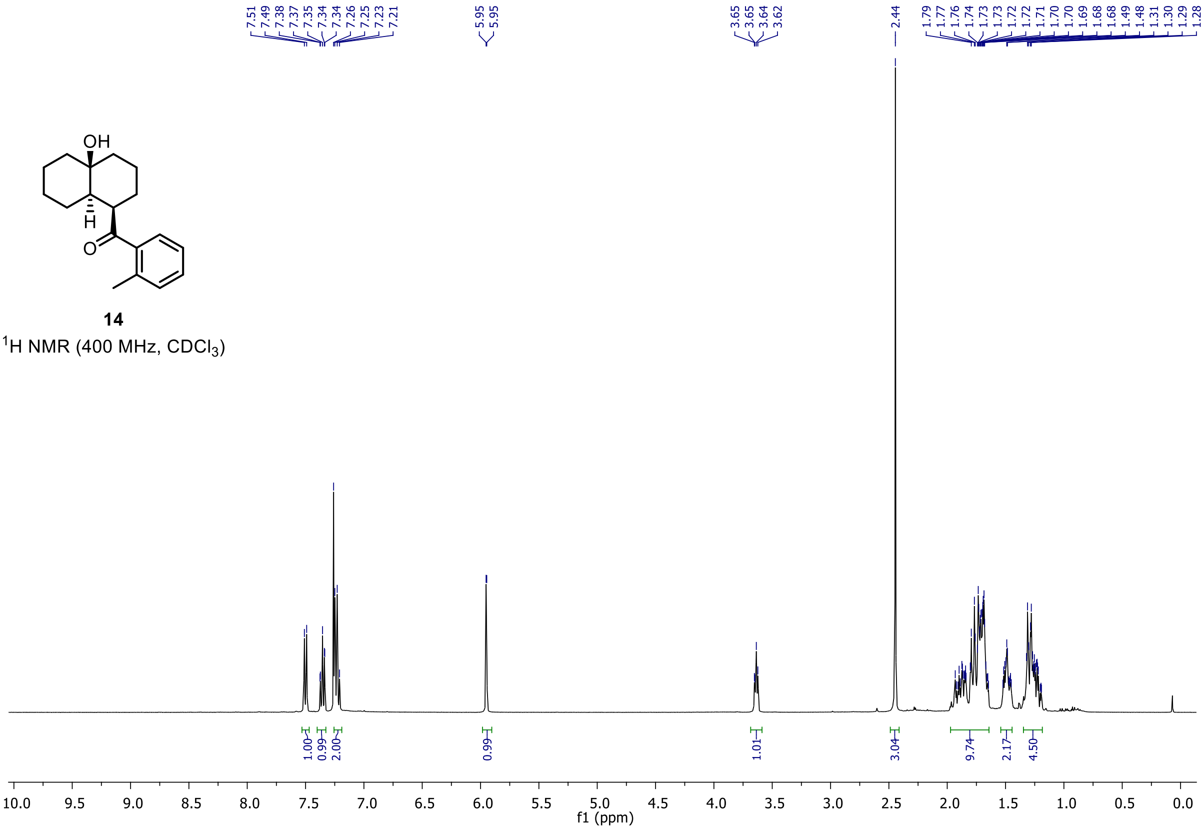
7.51 7.49 7.38 7.37 7.35 7.34 7.34 7.26 7.25 7.23 7.21
5.95 5.95
3.65 3.65 3.64 3.62
2.44
1.79 1.77 1.76 1.74 1.73 1.73 1.72 1.72 1.71 1.70 1.70 1.69 1.68 1.68 1.49 1.48 1.31 1.30 1.29 1.28
OH
H
O
14
^{1}H NMR (400 MHz, CDCl$_3$)
1.00 0.99 2.00 0.99 1.01 3.04 9.74 2.17 4.50
f1 (ppm)

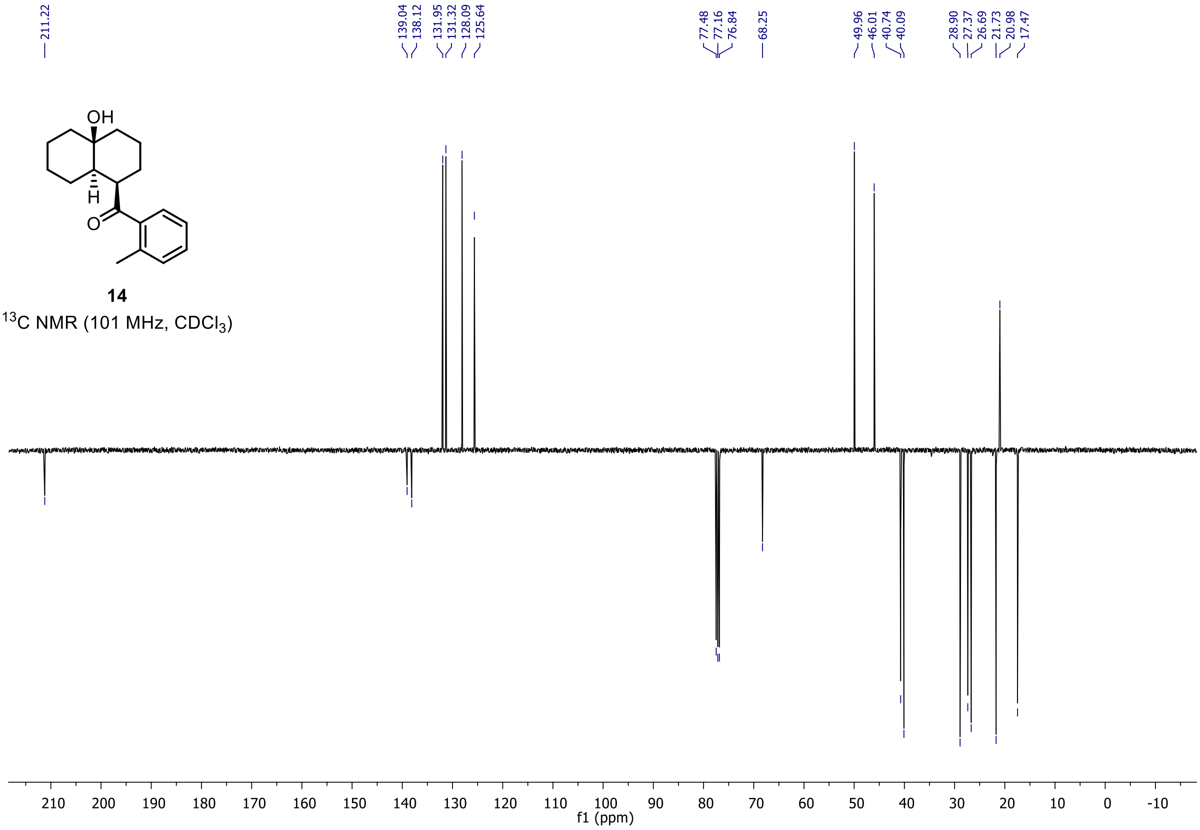
211.22
139.04 138.12 131.95 131.32 128.09 125.64
77.48 77.16 76.84 68.25
49.96 46.01 40.74 40.09
28.90 27.37 26.69 21.73 20.98 17.47
OH
H
O
14
^{13}C NMR (101 MHz, CDCl$_3$)
f1 (ppm)

(4-hydroxydecahydronaphthalen-1-yl)(naphthalen-2-yl)methanone (15)

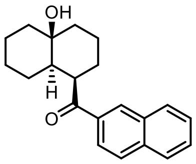

15

The title compound was prepared following **General Procedure A** using 2-naphthoyl chloride (38 mg, 0.20 mmol, 1.00 equiv.), *cis*-decalin (93 µL, 0.60 mmol, 3.00 equiv.) and $AgSbF_6$ (72 mg, 0.21 mmol, 1.05 equiv.). Purification by flash column chromatography (0 – 30% Et_2O in pentane) gave **15** as a yellow crystalline solid (11.1 mg, 36%).

R_f = 0.24 in 20% Et_2O/heptanes

m.p. (+5 °C/min) = 119.1 – 122.3 °C

^{1}H NMR (400 MHz, $CDCl_3$) δ 8.49 (s, 1H), 8.03 (dd, *J* = 8.7, 1.8 Hz, 1H), 7.97 (d, *J* = 8.1 Hz, 1H), 7.89 (t, *J* = 8.5 Hz, 2H), 7.65 – 7.53 (m, 2H), 6.15 (d, *J* = 2.2 Hz, 1H), 4.03 (t, *J* = 5.1 Hz, 1H), 2.04 – 1.85 (m, 3H), 1.82 – 1.67 (m, 6H), 1.55 – 1.45 (m, 2H), 1.37 – 1.19 (m, 4H).

^{13}C NMR (101 MHz, $CDCl_3$) δ 206.1 (C), 135.8 (C), 134.1 (C), 132.6 (C), 130.7 (CH), 129.8 (CH), 128.9 (CH), 128.8 (CH), 127.9 (CH), 127.1 (CH), 124.5 (CH), 68.2 (C), 46.1 (CH), 45.9 (CH), 40.8 (CH_2), 40.2 (CH_2), 30.1 (CH_2), 27.4 (CH_2), 26.7 (CH_2), 21.8 (CH_2), 17.6 (CH_2).

IR (neat) v_{max}: 3383, 2926, 2854, 1655, 1447, 1278, 1215, 1188, 1125, 959, 757.

HRMS (ESI$^+$): exact mass calculated for $[M+Na]^+$ ($C_{21}H_{24}O_2Na$) requires *m*/*z* 331.1669, found *m*/*z* 331.1674.

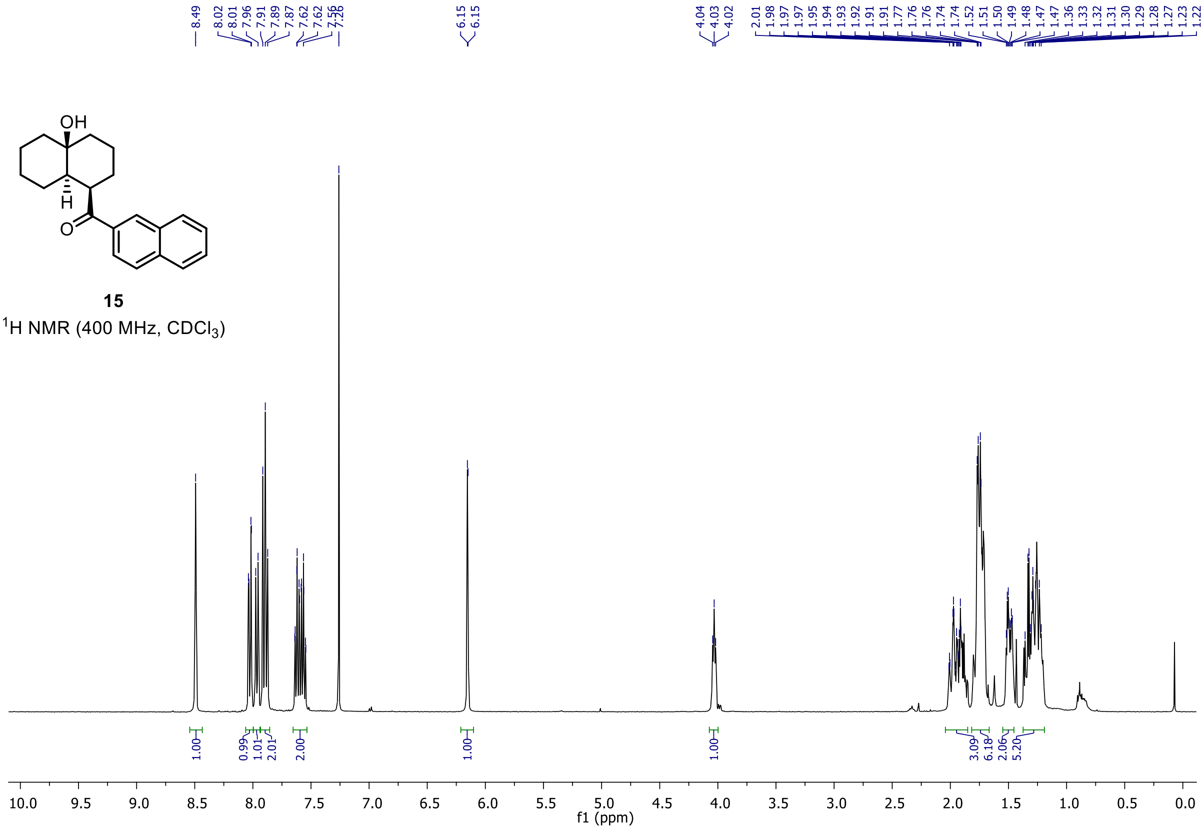

15

^{1}H NMR (400 MHz, CDCl$_3$)

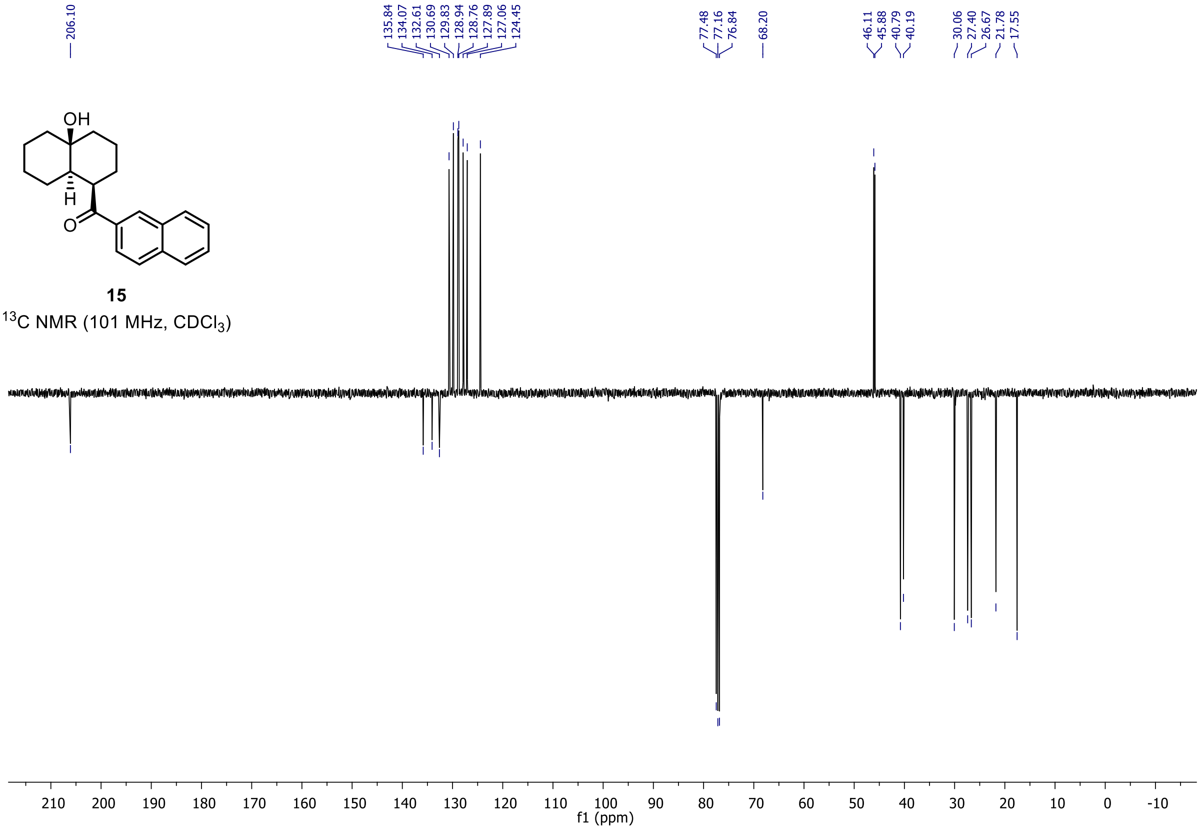

15

^{13}C NMR (101 MHz, CDCl$_3$)

(4-fluorophenyl)-4-hydroxydecahydronaphthalen-1-yl)methanone (16)

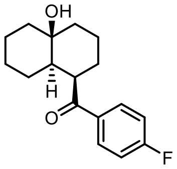

16

The title compound was prepared following **General Procedure A** using 4-fluorobenzoyl chloride (24 µL, 0.20 mmol, 1.00 equiv.), *cis*-decalin (93 µL, 0.60 mmol, 3.00 equiv.) and $AgSbF_6$ (72 mg, 0.21 mmol, 1.05 equiv.). Purification by flash column chromatography (0 – 20% Et_2O in pentane) gave **16** as a colorless oil (14.4 mg, 52%).

R_f = 0.40 in 20% EtOAc/heptanes

^{1}H NMR (400 MHz, $CDCl_3$) δ 7.99 (m, 2H), 7.13 (m, 2H), 6.01 (d, *J* = 2.2 Hz, 1H), 3.80 (m, 1H), 1.92-1.78 (m, 3H), 1.74-1.65 (m, 6H), 1.49-1.43 (m, 2H), 1.33-1.18 (m, 4H).

^{13}C NMR (101 MHz, $CDCl_3$) δ 204.5 (C), 166.2 (d, *J* = 255.8 Hz, C), 133.1 (C), 131.6 (d, *J* = 9.6 Hz, 2CH), 116.1 (d, *J* = 21.9 Hz, 2CH), 68.1 (C), 46.0 (CH), 45.8 (CH), 40.7 (CH_2), 40.1 (CH_2), 29.9 (CH_2), 27.3 (CH_2), 26.6 (CH_2), 21.7 (CH_2), 17.5 (CH_2).

^{19}F NMR (377 MHz, $CDCl_3$) δ -104.43.

IR (neat) v_{max}: 3388, 2927, 2855, 1660, 1594, 1506, 1447, 1412, 1215, 1155, 959, 846.

HRMS (ESI$^+$): exact mass calculated for $[M+Na]^+$ ($C_{17}H_{21}FO_2Na$) requires *m*/*z* 299.1418, found *m*/*z* 299.1414.

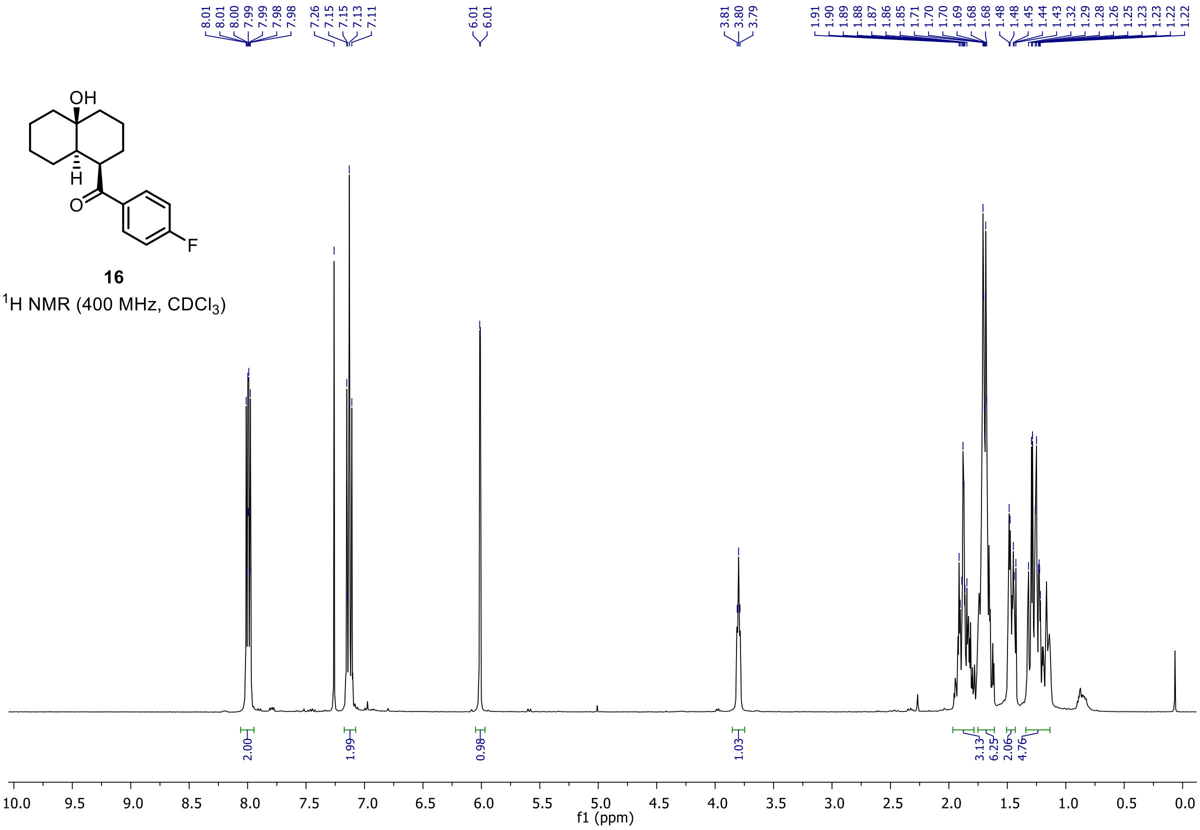

16

^{1}H NMR (400 MHz, $CDCl_3$)

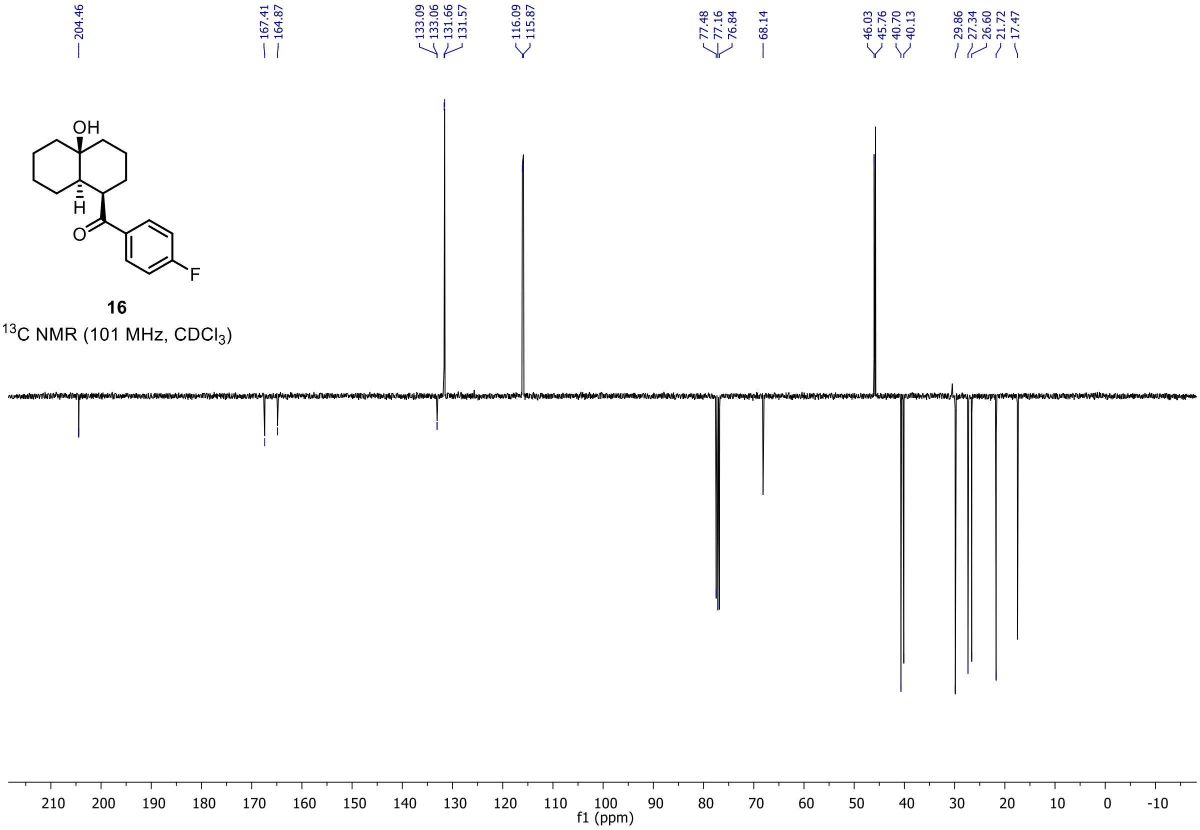

16

^{13}C NMR (101 MHz, $CDCl_3$)

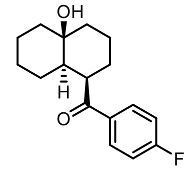

16

^{19}F NMR (377 MHz, $CDCl_3$)

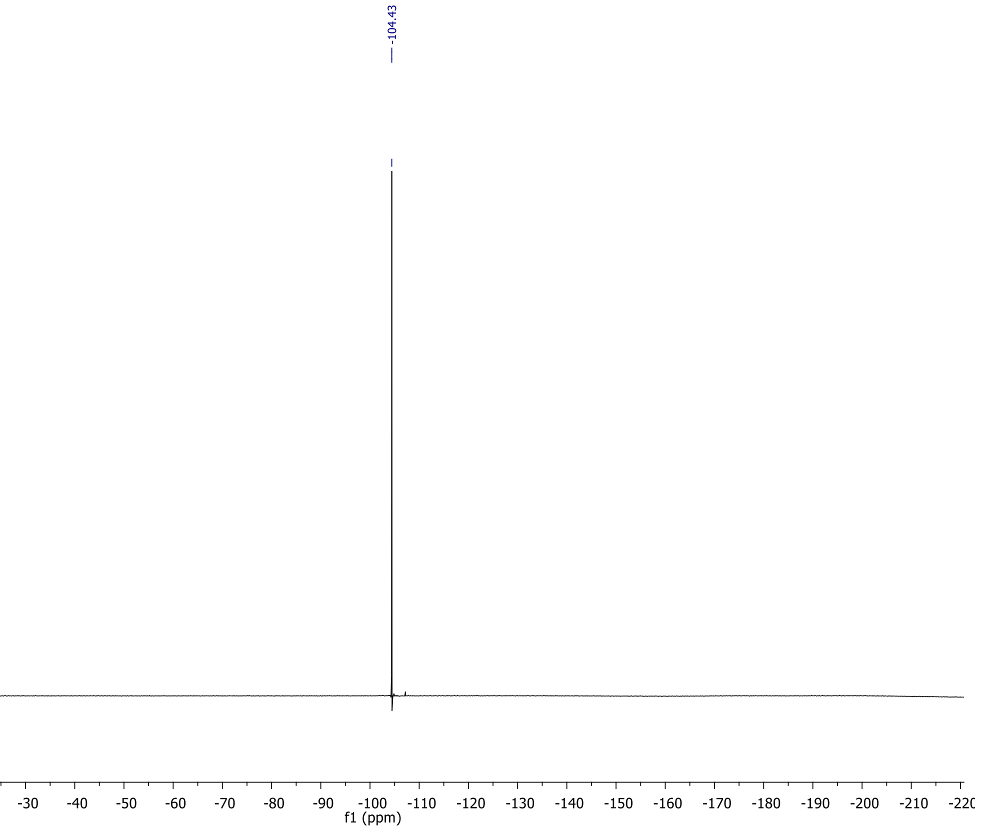

(4-hydroxydecahydronaphthalen-1-yl)(4-methoxyphenyl)methanone (17)

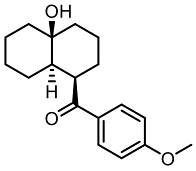

17

The title compound was prepared following **General Procedure A** using 4-methoxybenzoyl chloride (34 µL, 0.20 mmol, 1.00 equiv.), *cis*-decalin (93 µL, 0.60 mmol, 3.00 equiv.) and $AgSbF_6$ (72 mg, 0.21 mmol, 1.05 equiv.). Purification by flash column chromatography (0 – 40% Et_2O in pentane) gave **17** as a colorless oil (16.6 mg, 58%).

R_f = 0.33 in 20% EtOAc/heptanes

^{1}H NMR (400 MHz, $CDCl_3$) δ 8.00 – 7.93 (m, 2H), 6.96 – 6.89 (m, 2H), 6.27 (d, *J* = 2.2 Hz, 1H), 3.87 (s, 3H), 3.83 – 3.77 (m, 1H), 1.95 – 1.65 (m, 9H), 1.50 – 1.41 (m, 2H), 1.35 – 1.18 (m, 4H).

^{13}C NMR (101 MHz, $CDCl_3$) δ 204.5 (C), 164.0 (C), 131.3 (2CH), 129.6 (C), 114.0 (2CH), 68.1 (C), 55.7 (CH_3), 45.8 (CH), 45.6 (CH), 40.9 (CH_2), 40.2 (CH_2), 30.1 (CH_2), 27.4 (CH_2), 26.6 (CH_2), 21.8 (CH_2), 17.6 (CH_2).

IR (neat) v_{max}: 3359, 2928, 2854, 1651, 1596, 1251, 1222, 1170, 960.

HRMS (ESI$^+$): exact mass calculated for $[M+Na]^+$ ($C_{18}H_{24}O_3Na$) requires *m*/*z* 311.1618, found *m*/*z* 311.1619.

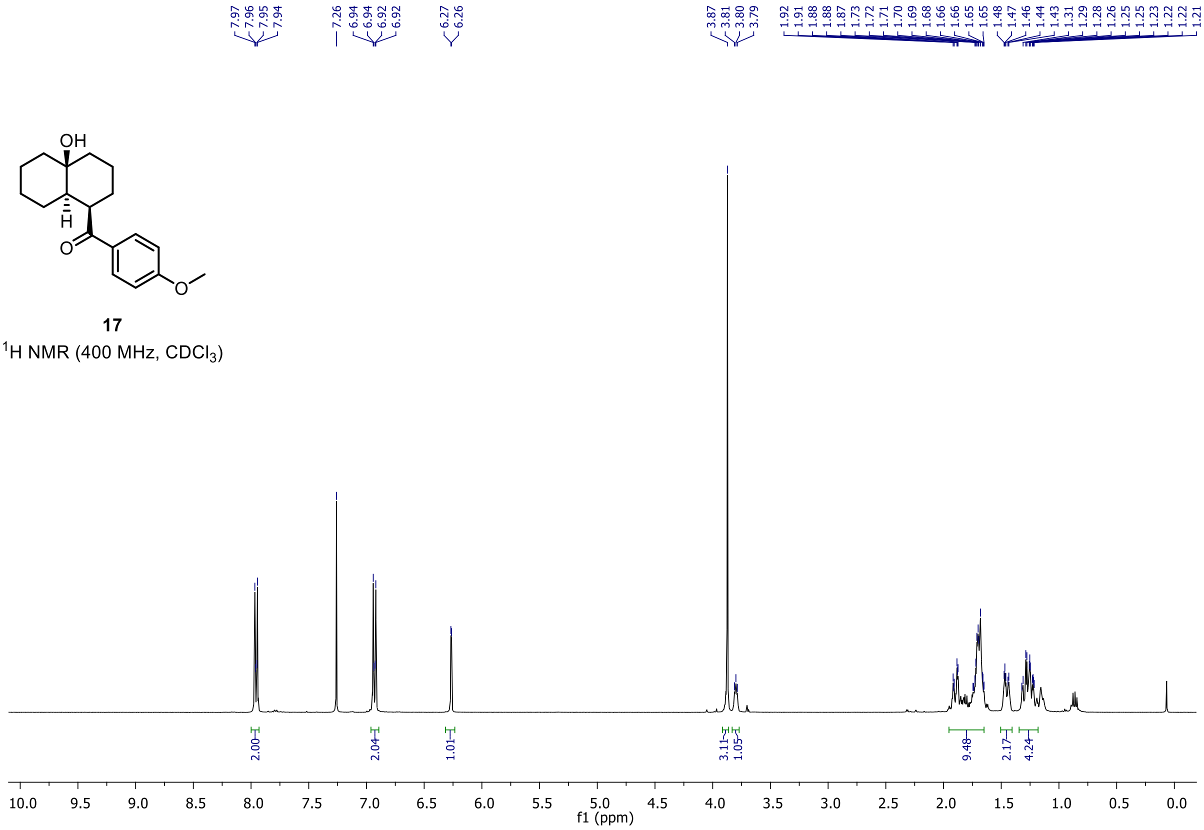

17

^{1}H NMR (400 MHz, $CDCl_3$)

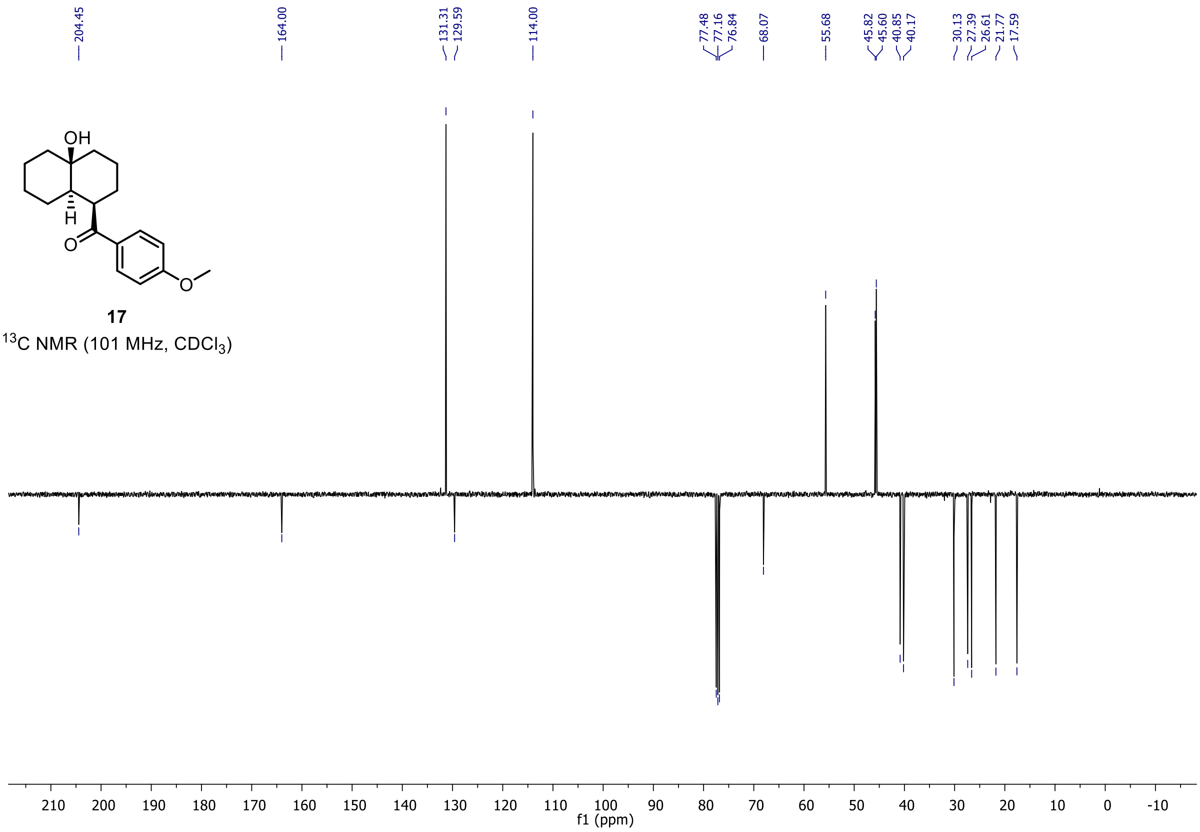

17

^{13}C NMR (101 MHz, $CDCl_3$)

(4-(tert-butyl)phenyl)-4-hydroxydecahydronaphthalen-1-yl)methanone (18)

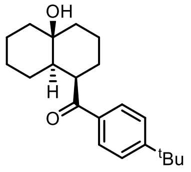

18

The title compound was prepared following **General Procedure A** using 4-*tert*-butylbenzoyl chloride (39 µL, 0.20 mmol, 1.00 equiv.), *cis*-decalin (93 µL, 0.60 mmol, 3.00 equiv.) and $AgSbF_6$ (72 mg, 0.21 mmol, 1.05 equiv.). Purification by flash column chromatography (5 – 25% EtOAc in heptanes) gave **18** as a colorless oil (19.7 mg, 63%).

R_f = 0.52 in 20% EtOAc/heptanes

^{1}H NMR (400 MHz, $CDCl_3$) δ 7.94 – 7.89 (m, 2H), 7.50 – 7.44 (m, 2H), 6.20 (d, *J* = 2.2 Hz, 1H), 3.95 – 3.73 (m, 1H), 1.97 – 1.61 (m, 9H), 1.51 – 1.39 (m, 2H), 1.34 (s, 9H), 1.32 – 1.12 (m, 4H).

^{13}C NMR (101 MHz, $CDCl_3$) δ 205.6 (C), 157.5 (C), 134.0 (C), 128.9 (2C), 125.8 (2C), 68.1 (C), 45.9 (CH), 45.8 (CH), 40.8 (CH_2), 40.2 (CH_2), 35.3 (C), 31.2 ($3CH_3$), 30.0 (CH_2), 27.4 (CH_2), 26.6 (CH_2), 21.8 (CH_2), 17.5 (CH_2).

IR (neat) v_{max}: 3391, 2921, 2849, 1661, 1440, 1211, 1156, 978, 943, 731.

HRMS (ESI$^+$): exact mass calculated for $[M+Na]^+$ ($C_{21}H_{30}O_2$) requires *m/z* 337.2138, found *m/z* 337.2138.

18

^{1}H NMR (400 MHz, $CDCl_3$)

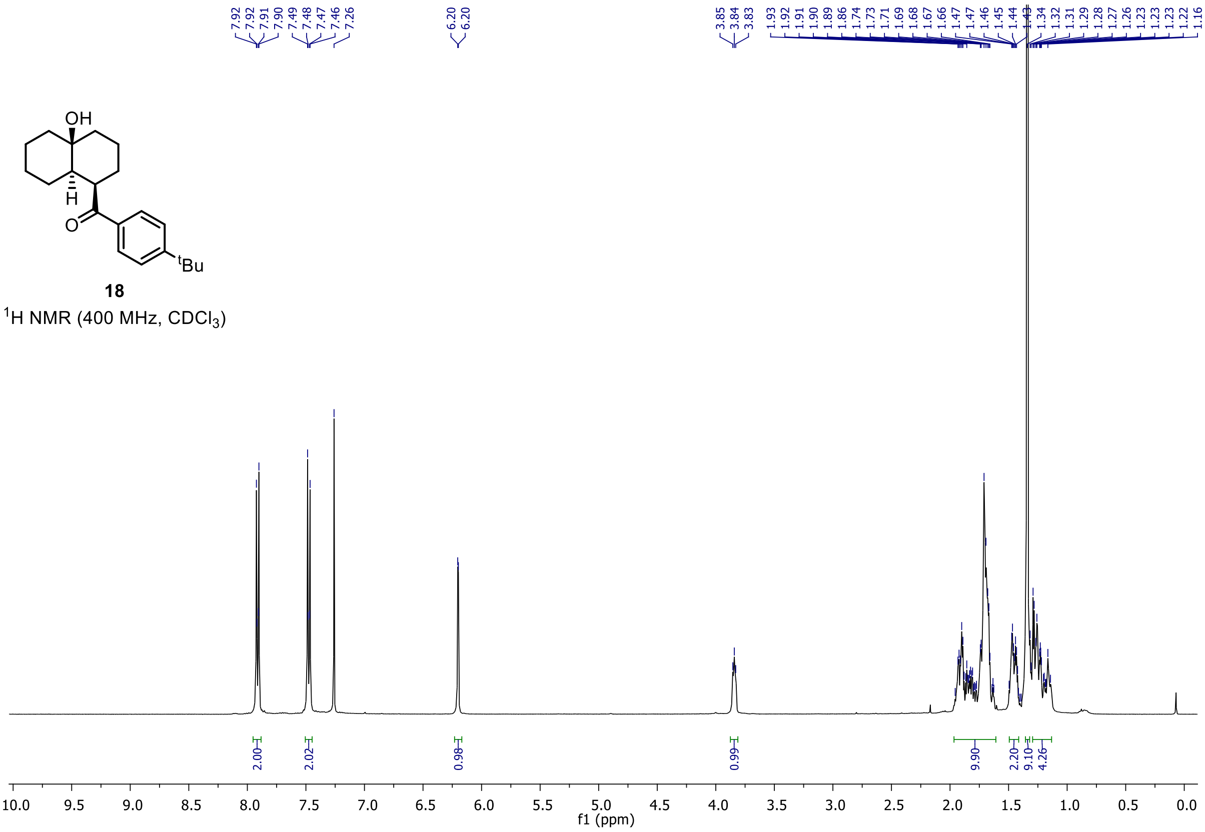

18

^{13}C NMR (101 MHz, $CDCl_3$)

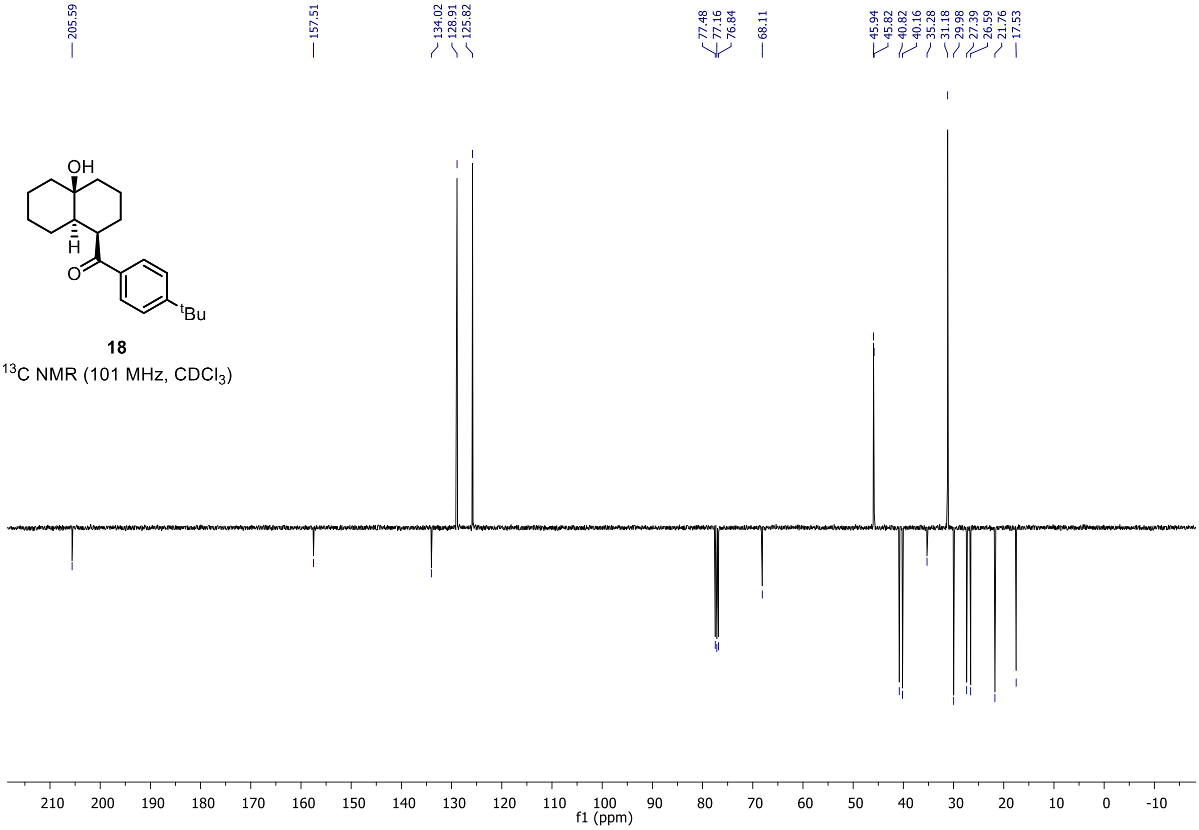

(4-hydroxydecahydronaphthalen-1-yl)(naphthalen-1-yl)methanone (19)

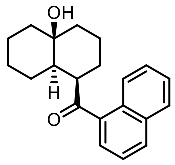

19

The title compound was prepared following **General Procedure A** using 1-naphthoyl chloride (38 mg, 0.20 mmol, 1.00 equiv.), *cis*-decalin (93 µL, 0.60 mmol, 3.00 equiv.) and $AgSbF_6$ (72 mg, 0.21 mmol, 1.05 equiv.). Purification by flash column chromatography (0 – 25% Et_2O in pentane) gave **19** as a yellow crystalline solid (13.5 mg, 44%).

A crystal of sufficient quality for X-ray diffractometry was obtained by slow evaporation of a solution in Et_2O/pentane (10 mg dissolved in approx. 2 mL 1:3 Et_2O/pentane at 23 °C, vial loosely capped and stored at 23 °C until complete solvent evaporation).

R_f = 0.22 in 10% EtOAc/heptanes

m.p. (+5 °C/min) = 136.6 – 141.9 °C

^{1}H NMR (600 MHz, $CDCl_3$) δ 8.33 (d, *J* = 8.5 Hz, 1H), 7.99 (d, *J* = 8.0 Hz, 1H), 7.89 (d, *J* = 8.0 Hz, 1H), 7.75 (dd, *J* = 7.1 Hz, *J* = 0.8 Hz, 1H), 7.60 (m, 1H), 7.55 (m, 1H), 7.48 (t, *J* = 7.7 Hz, 1H), 5.96 (d, *J* = 2.2 Hz, 1H), 3.81 (t, *J* = 8.5 Hz, 1H), 1.85-1.97 (m, 4H), 1.72-1.82 (m, 5H), 1.49-1.56 (m, 2H), 1.28-1.40 (m, 4H).

^{13}C NMR (151 MHz, $CDCl_3$) δ 211.1 (C), 137.4 (C), 134.0 (C), 132.6 (CH), 130.6 (C), 128.6 (CH), 128.1 (CH), 127.0 (CH), 126.8 (CH), 125.6 (CH), 124.3 (CH), 68.4 (C), 50.8 (CH), 46.1 (CH), 40.7 (CH_2), 40.2 (CH_2), 29.0 (CH_2), 27.4 (CH_2), 26.8 (CH_2), 21.8 (CH_2), 17.5 (CH_2).

IR (neat) ν_{max}: 3398, 2925, 2853, 1660, 1446, 1214, 957, 788, 775, 753.

HRMS (ESI$^+$): exact mass calculated for $[M+Na]^+$ ($C_{21}H_{24}O_2Na$) requires *m*/*z* 331.1669, found *m*/*z* 331.1665.

SC-XRD: See Section **5**.

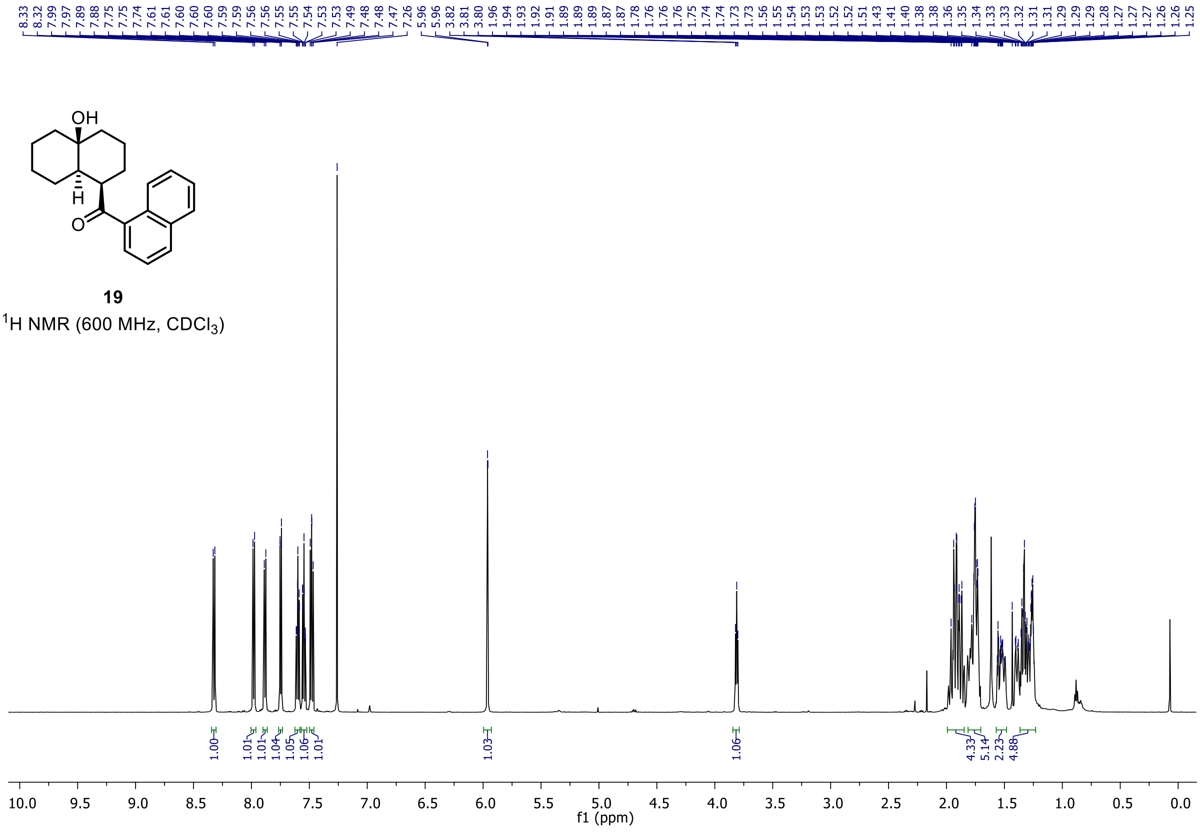
8.33 8.32 7.99 7.97 7.89 7.88 7.75 7.75 7.74 7.61 7.61 7.60 7.60 7.60 7.59 7.59 7.56 7.56 7.55 7.55 7.54 7.53 7.53 7.49 7.48 7.47 7.26 5.96 5.96 3.82 3.81 3.80 1.96 1.94 1.93 1.92 1.91 1.89 1.89 1.89 1.87 1.87 1.78 1.76 1.76 1.76 1.75 1.74 1.74 1.73 1.73 1.56 1.55 1.54 1.53 1.53 1.52 1.52 1.51 1.43 1.41 1.40 1.38 1.38 1.36 1.35 1.34 1.33 1.33 1.32 1.31 1.31 1.29 1.29 1.28 1.27 1.27 1.27 1.26 1.25
OH
H
O
19
1H NMR (600 MHz, CDCl3)
1.00 1.01 1.01 1.04 1.05 1.06 1.01 1.03 1.06 4.33 5.14 2.23 4.88
10.0 9.5 9.0 8.5 8.0 7.5 7.0 6.5 6.0 5.5 5.0 4.5 4.0 3.5 3.0 2.5 2.0 1.5 1.0 0.5 0.0
f1 (ppm)

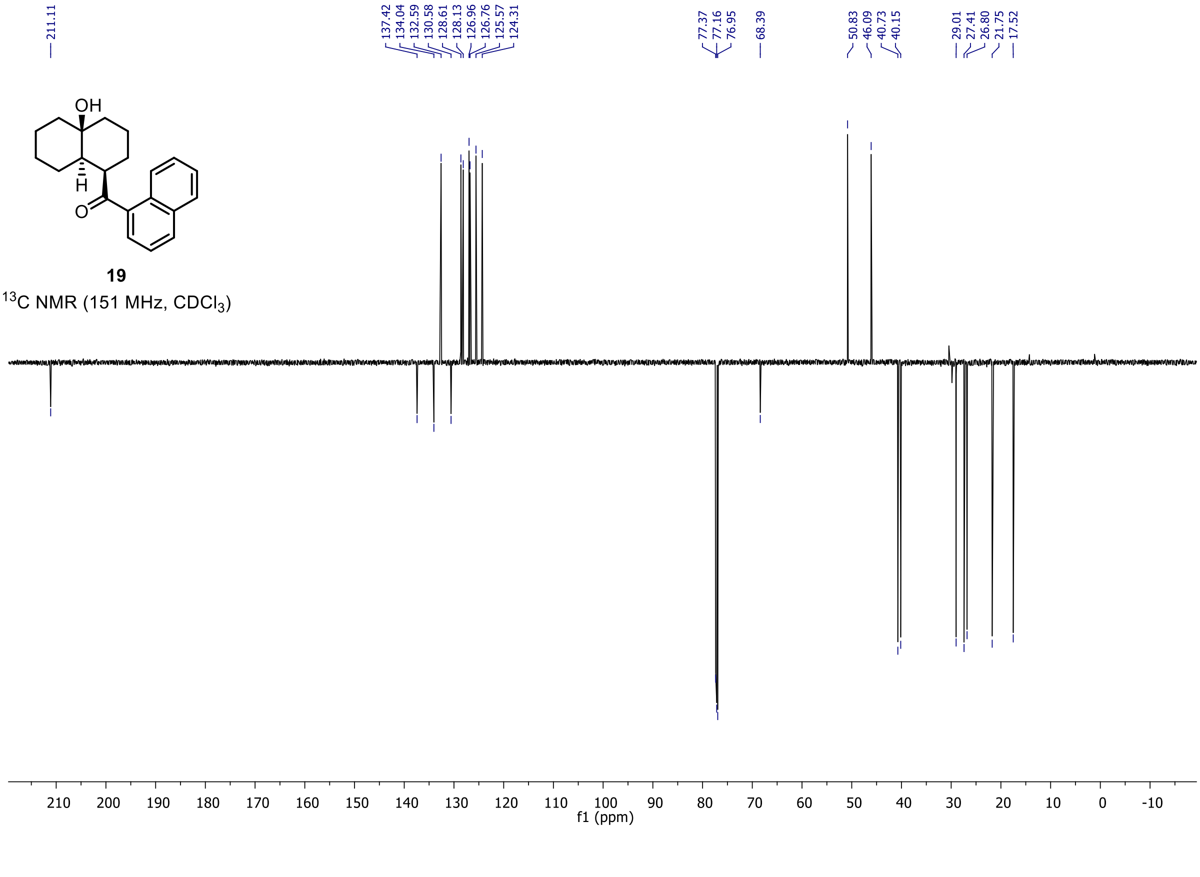
211.11
137.42 134.04 132.59 130.58 128.61 128.13 126.96 126.76 125.57 124.31
77.37 77.16 76.95 68.39
50.83 46.09 40.73 40.15
29.01 27.41 26.80 21.75 17.52
OH
H
O
19
13C NMR (151 MHz, CDCl3)
210 200 190 180 170 160 150 140 130 120 110 100 90 80 70 60 50 40 30 20 10 0 -10
f1 (ppm)

(4-hydroxydecahydronaphthalen-1-yl)(2-iodophenyl)methanone (20)

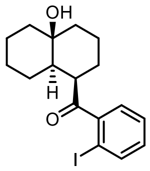

20

The title compound was prepared following **General Procedure A** using 2-iodobenzoyl chloride (28 µL, 0.20 mmol, 1.00 equiv.), *cis*-decalin (93 µL, 0.60 mmol, 3.00 equiv.) and $AgSbF_6$ (72 mg, 0.21 mmol, 1.05 equiv.). Purification by flash column chromatography (0 – 20% Et_2O in pentane) gave **20** as a yellow crystalline solid (21.1 mg, 55%).

A crystal of sufficient quality for X-ray diffractometry was obtained by slow evaporation of a solution in $CHCl_3$ (20 mg dissolved in approx. 2.0 mL $CHCl_3$ at 23 °C, vial loosely capped and stored at 23 °C until complete solvent evaporation).

$\mathbf{R_f}$ = 0.46 in 40% Et_2O/heptanes

m.p. (+5 °C/min) = 144.4 – 146.8 °C

^{1}H NMR (400 MHz, $CDCl_3$) δ 7.91 (dd, *J* = 8.0, 0.7 Hz, 1H), 7.39 (td, *J* = 7.6, 1.0 Hz, 1H), 7.33 (dd, *J* = 7.7, 1.8 Hz, 1H), 7.15 – 7.09 (m, 1H), 5.52 (d, *J* = 2.2 Hz, 1H), 3.59 (t, *J* = 5.5 Hz, 1H), 2.00 – 1.64 (m, 9H), 1.55 – 1.42 (m, 2H), 1.35 – 1.17 (m, 4H).

^{13}C NMR (101 MHz, $CDCl_3$) δ 210.3 (C), 144.9 (C), 141.0 (CH), 131.9 (CH), 128.4 (CH), 127.9 (CH), 92.1 (C), 68.4 (C), 50.8 (CH), 46.2 (CH), 40.7 (CH_2), 40.1 (CH_2), 28.4 (CH_2), 27.4 (CH_2), 26.7 (CH_2), 21.7 (CH_2), 17.6 (CH_2).

IR (neat) ν_{max}: 3416, 2926, 2853, 1678, 1446, 1213, 958, 770, 739.

HRMS (ESI$^+$): exact mass calculated for $[M+Na]^+$ ($C_{17}H_{21}IO_2Na$) requires *m*/*z* 407.0478, found *m*/*z* 407.0484.

SC-XRD: See Section **5**.

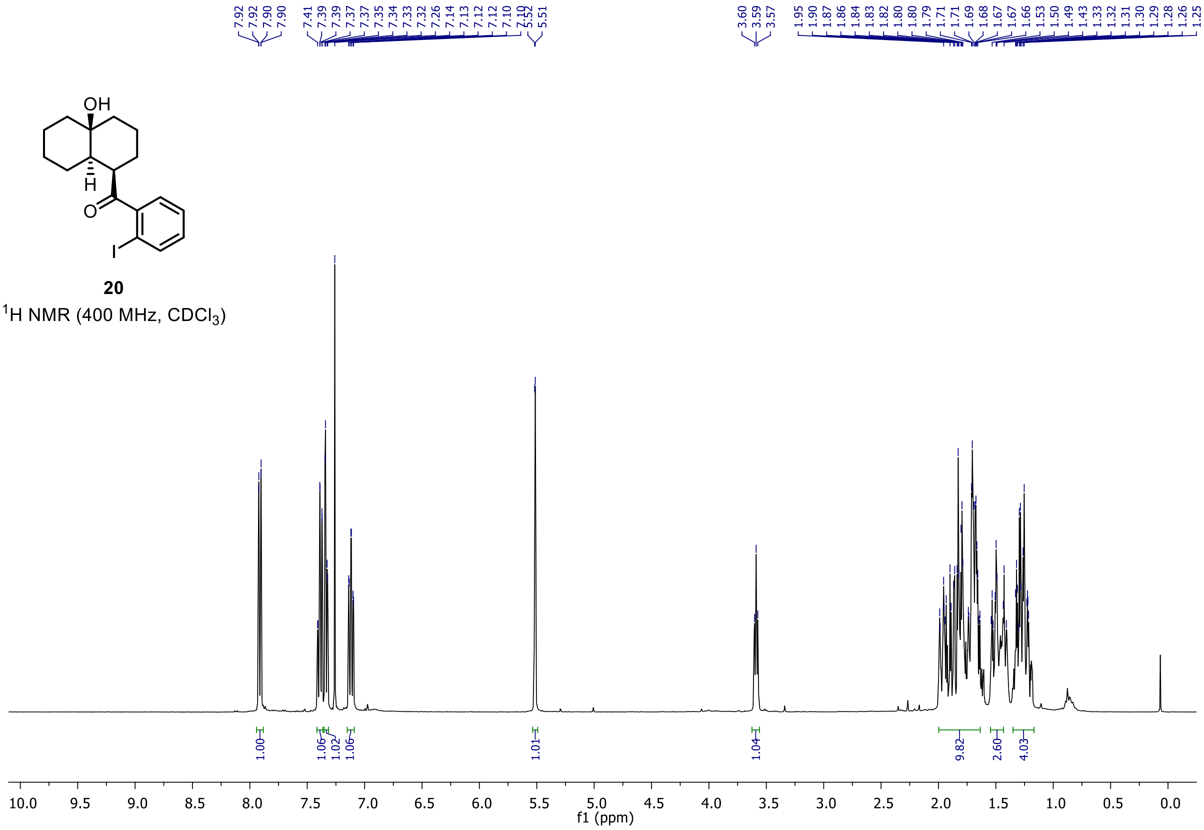

20

^{1}H NMR (400 MHz, $CDCl_3$)

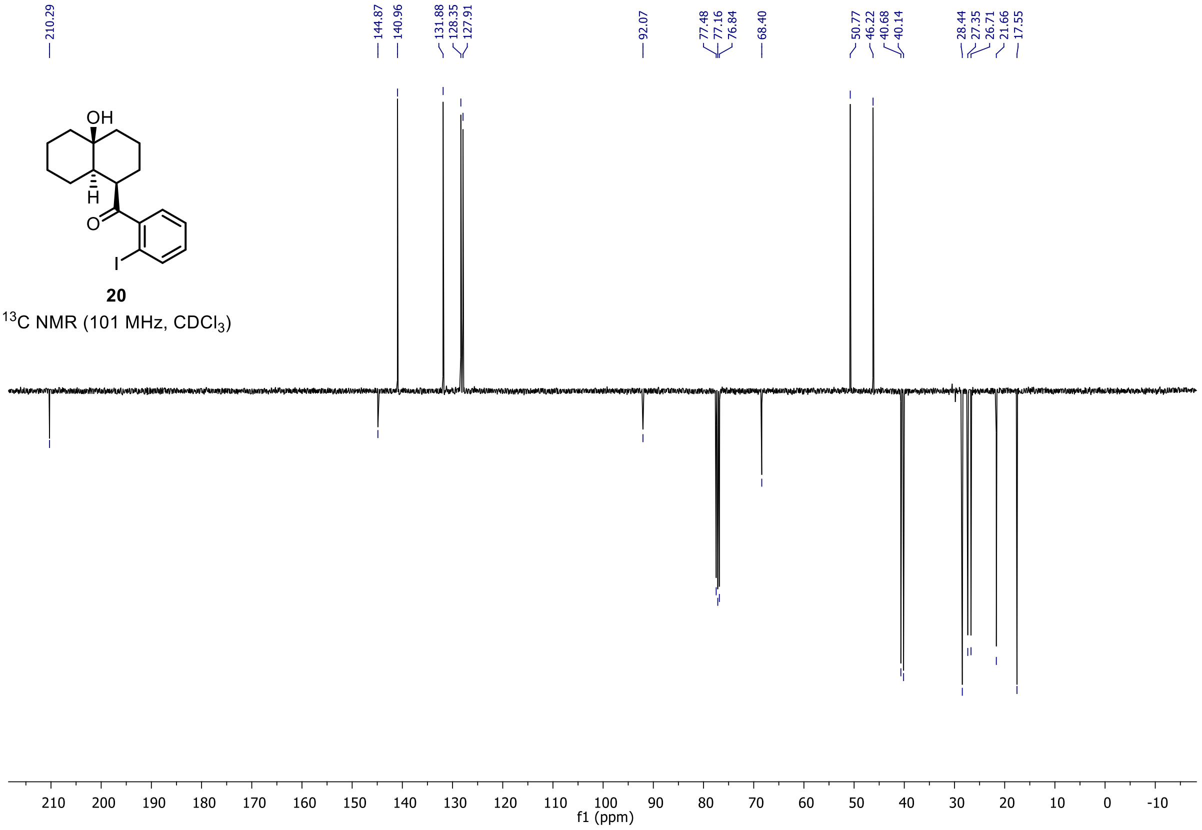

20

^{13}C NMR (101 MHz, $CDCl_3$)

General Procedure B: Modified Baddeley reaction for electron poor acyl chlorides

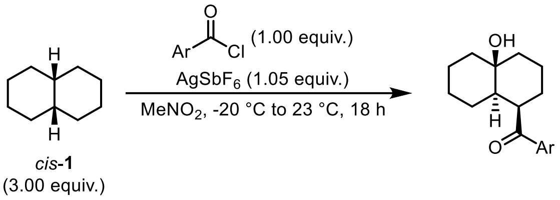

In a small flame-dried vial with a rubber septum cap, $AgSbF_6$ (72 mg, 0.21 mmol, 1.05 equiv., weighed in glovebox) was dissolved in 0.5 mL $MeNO_2$ at ambient temperature (23 °C) and then cooled to –20 °C (acetone bath cooled with dry ice). Acyl chloride (0.20 mmol, 2.00 equiv.) was added under stirring at –20 °C, followed by *cis*-decalin (93 µL, 0.60 mmol, 3.00 equiv.). The cap was sealed with multiple layers of parafilm[a] and the resulting suspension was then stirred for 18 hours while slowly warmed to ambient temperature (23 °C, cooling bath left to warm up overnight). The reaction was stopped by adding 10% aqueous K_2CO_3 solution (2 mL) and stirring for 1 h at ambient temperature (23 °C). The mixture was transferred to a separatory funnel, diluted with more 10% aqueous K_2CO_3 solution (5 mL) and the aqueous layer was extracted with DCM (3 × 5 mL). The combined organic layers were dried over anhydrous sodium sulfate, the dried solution was filtered, and the filtrate was concentrated under reduced pressure (final drying for >30 min at <1 mbar at 23 °C to remove unreacted decalin and $MeNO_2$). The crude residue was purified by flash column chromatography to afford the desired compound.

[a]Corrosion of rubber septum caps was sometimes observed.

(4-hydroxydecahydronaphthalen-1-yl)(4-(trifluoromethyl)phenyl)methanone (21)

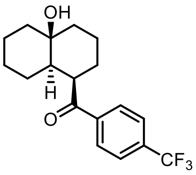

21

The title compound was prepared following **General Procedure B** using 4-(trifluoromethyl)benzoyl chloride (31 µL, 0.20 mmol, 1.00 equiv.), *cis*-decalin (93 µL, 0.60 mmol, 3.00 equiv.) and $AgSbF_6$ (72 mg, 0.21 mmol, 1.05 equiv.). Purification by flash column chromatography (0 – 20% Et_2O in pentane) gave **21** as a colorless oil (17.0 mg, 52%).

R_f = 0.47 in 20% EtOAc/heptanes

^{1}H NMR (400 MHz, $CDCl_3$) δ 8.06 (d, *J* = 8.2 Hz, 2H), 7.73 (d, *J* = 8.3 Hz, 2H), 5.78 (d, *J* = 2.3 Hz, 1H), 3.87 – 3.80 (m, 1H), 1.97 – 1.81 (m, 3H), 1.78 – 1.61 (m, 6H), 1.53 – 1.43 (m, 2H), 1.36 – 1.17 (m, 4H).

^{13}C NMR (101 MHz, $CDCl_3$) δ 205.3 (C), 139.5 (C), 134.9 (q, *J* = 33.7 Hz, C), 129.2 (2CH), 126.0 (q, *J* = 3.6 Hz, 2CH), 123.7 (q, *J* = 272.5 Hz, C), 68.3 (C), 46.6 (CH), 45.8 (CH), 40.6 (CH_2), 40.1 (CH_2), 29.6 (CH_2), 27.3 (CH_2), 26.6 (CH_2), 21.7 (CH_2), 17.4 (CH_2).

^{19}F NMR (376 MHz, $CDCl_3$) δ -63.21.

IR (neat) v_{max}: 3414, 2929, 2856, 1669, 1448, 1323, 1168, 1128, 1066, 959.

HRMS (ESI$^+$): exact mass calculated for $[M+Na]^+$ ($C_{18}H_{21}F_3O_2Na$) requires *m*/*z* 349.1386, found *m*/*z* 349.1390.

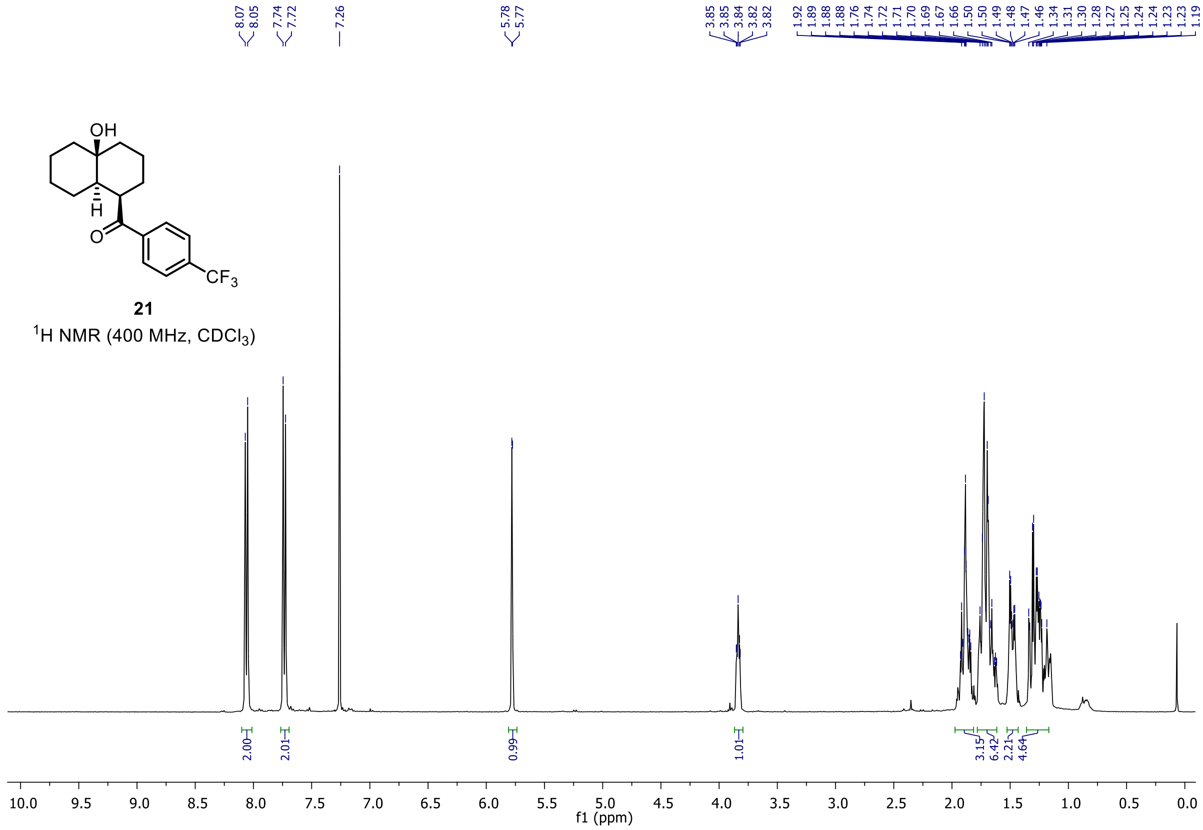
8.07
8.05
7.74
7.72
7.26
5.78
5.77
3.85
3.85
3.84
3.82
3.82
1.92
1.89
1.88
1.88
1.76
1.74
1.72
1.71
1.70
1.69
1.67
1.66
1.50
1.50
1.49
1.48
1.47
1.46
1.34
1.31
1.30
1.28
1.27
1.25
1.24
1.24
1.23
1.23
1.19
OH
H
O
CF3
21
1H NMR (400 MHz, CDCl3)
2.00
2.01
0.99
1.01
3.15
6.42
2.21
4.64
f1 (ppm)

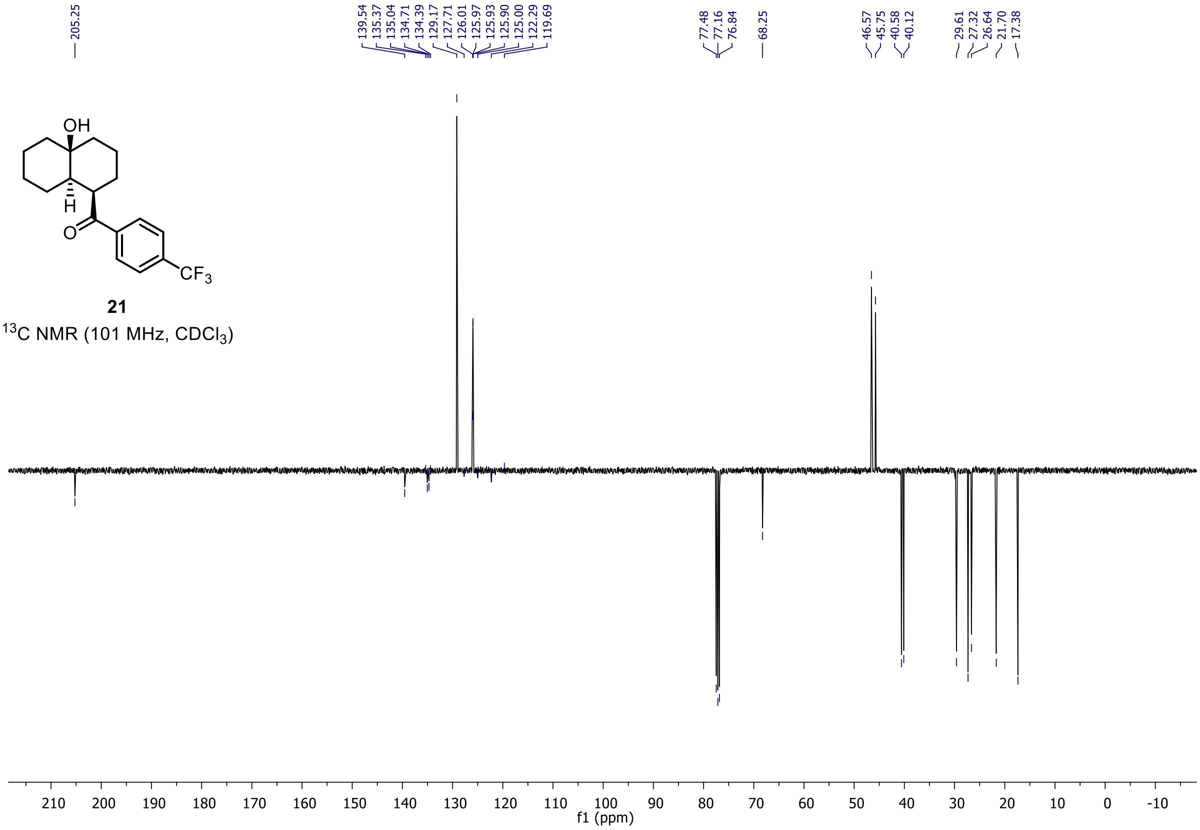
205.25
139.54
135.37
135.04
134.71
134.39
129.17
127.71
126.01
125.97
125.93
125.90
125.00
122.29
119.69
77.48
77.16
76.84
68.25
46.57
45.75
40.58
40.12
29.61
27.32
26.64
21.70
17.38
OH
H
O
CF3
21
13C NMR (101 MHz, CDCl3)
f1 (ppm)

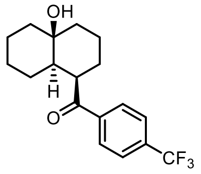

21

^{19}F NMR (377 MHz, $CDCl_3$)

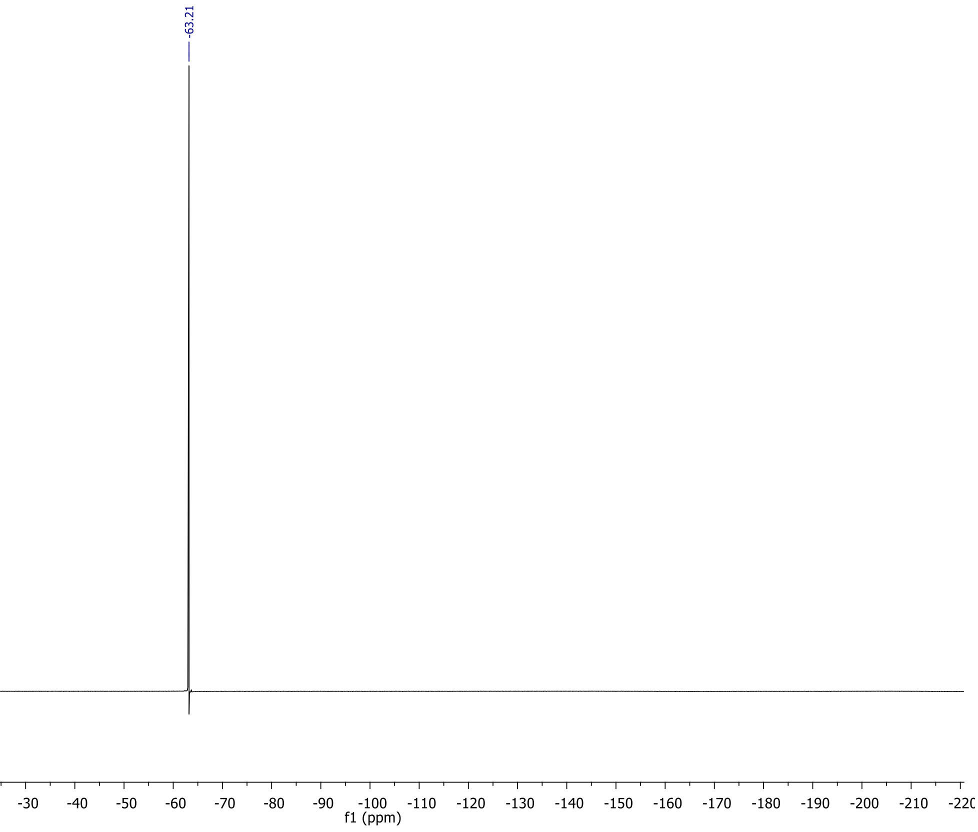

(3-chlorophenyl)(4-hydroxydecahydronaphthalen-1-yl)methanone (22)

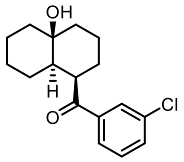

22

The title compound was prepared following **General Procedure B** using 3-chlorobenzoyl chloride (26 µL, 0.20 mmol, 1.00 equiv.), *cis*-decalin (93 µL, 0.60 mmol, 3.00 equiv.) and $AgSbF_6$ (72 mg, 0.21 mmol, 1.05 equiv.). Purification by flash column chromatography (0 – 25% Et_2O in pentane) gave **22** as a light-yellow oil (14.1 mg, 48%).

$\mathbf{R_f}$ = 0.23 in 10% EtOAc/heptanes

^{1}H NMR (400 MHz, $CDCl_3$) δ 7.92 (t, *J* = 1.9 Hz, 1H), 7.86 – 7.79 (m, 1H), 7.55 (ddd, *J* = 8.0, 2.1, 1.0 Hz, 1H), 7.41 (t, *J* = 8.0 Hz, 1H), 5.85 (d, *J* = 2.3 Hz, 1H), 3.81 – 3.74 (m, 1H), 1.96 – 1.79 (m, 3H), 1.76 – 1.60 (m, 6H), 1.52 – 1.42 (m, 2H), 1.34 – 1.14 (m, 4H).

^{13}C NMR (101 MHz, $CDCl_3$) δ 204.9 (C), 138.4 (C), 135.2 (C), 133.5 (CH), 130.2 (CH), 129.0 (CH), 126.9 (CH), 68.2 (C), 46.3 (CH), 45.7 (CH), 40.6 (CH_2), 40.1 (CH_2), 29.7 (CH_2), 27.3 (CH_2), 26.6 (CH_2), 21.7 (CH_2), 17.4 (CH_2).

IR (neat) ν_{max}: 3405, 2931, 2856, 1667, 1447, 1212, 959, 758, 738.

HRMS (ESI$^+$): exact mass calculated for $[M+Na]^+$ ($C_{17}H_{21}ClO_2Na$) requires *m*/*z* 315.1122, found *m*/*z* 315.1115.

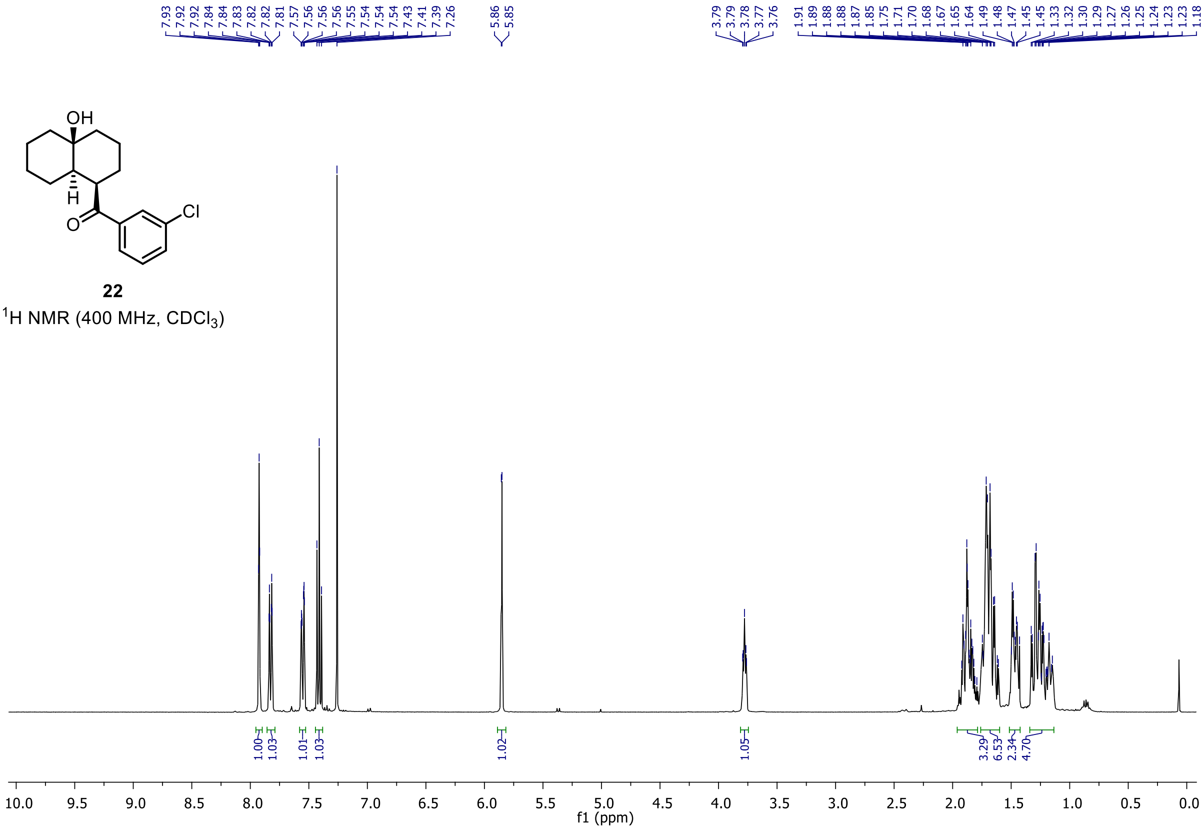

22

^{1}H NMR (400 MHz, $CDCl_3$)

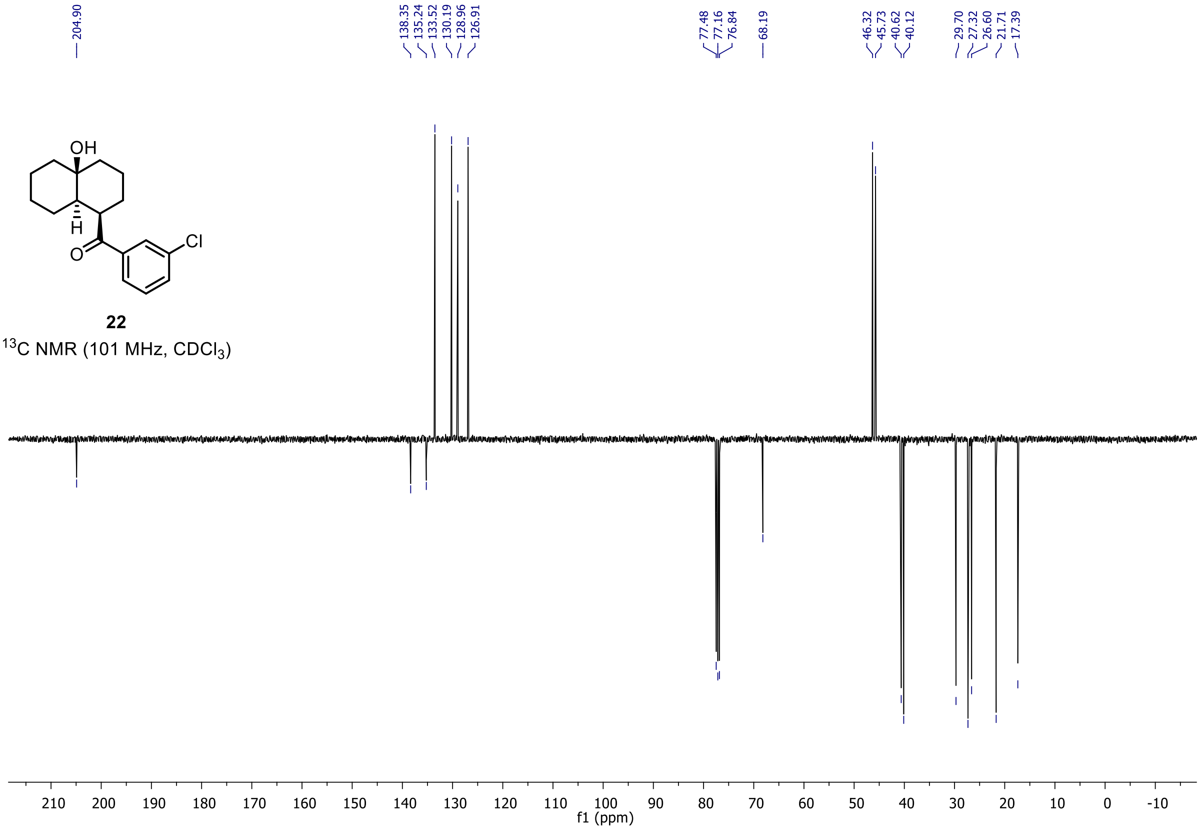

22

^{13}C NMR (101 MHz, $CDCl_3$)

methyl 4-(4-hydroxydecahydronaphthalene-1-cbonyl)benzoate (23)

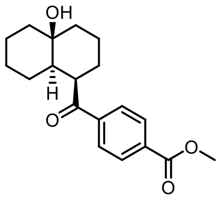

23

The title compound was prepared following **General Procedure B** using 4-nitrobenzoyl chloride (37 mg, 0.20 mmol, 1.00 equiv.), *cis*-decalin (93 µL, 0.60 mmol, 3.00 equiv.) and $AgSbF_6$ (72 mg, 0.21 mmol, 1.05 equiv.). Purification by flash column chromatography (0 – 40% Et_2O in pentane) gave **23** as a yellow crystalline solid (15.0 mg, 47%).

R_f = 0.34 in 20% EtOAc/heptanes

m.p. (+5 °C/min) = 115.3 – 117.9 °C

^{1}H NMR (400 MHz, $CDCl_3$) δ 8.14 – 8.09 (m, 2H), 8.02 – 7.97 (m, 2H), 5.84 (d, *J* = 2.2 Hz, 1H), 3.95 (s, 3H), 3.88 – 3.82 (m, 1H), 1.94 – 1.80 (m, 3H), 1.77 – 1.62 (m, 6H), 1.52 – 1.42 (m, 2H), 1.34 – 1.17 (m, 4H).

^{13}C NMR (101 MHz, $CDCl_3$) δ 205.8 (C), 166.2 (C), 140.1 (C), 134.3 (C), 130.1 (2CH), 128.7 (2CH), 68.2 (C), 52.7 (CH_3), 46.6 (CH), 45.8 (CH), 40.6 (CH_2), 40.1 (CH_2), 29.7 (CH_2), 27.3 (CH_2), 26.6 (CH_2), 21.7 (CH_2), 17.4 (CH_2).

IR (neat) v_{max}: 3408, 2928, 2854, 1724, 1665, 1274, 1213, 1106, 959.

HRMS (ESI$^+$): exact mass calculated for $[M+Na]^+$ ($C_{19}H_{24}O_4Na$) requires *m*/*z* 339.1567, found *m*/*z* 339.1573.

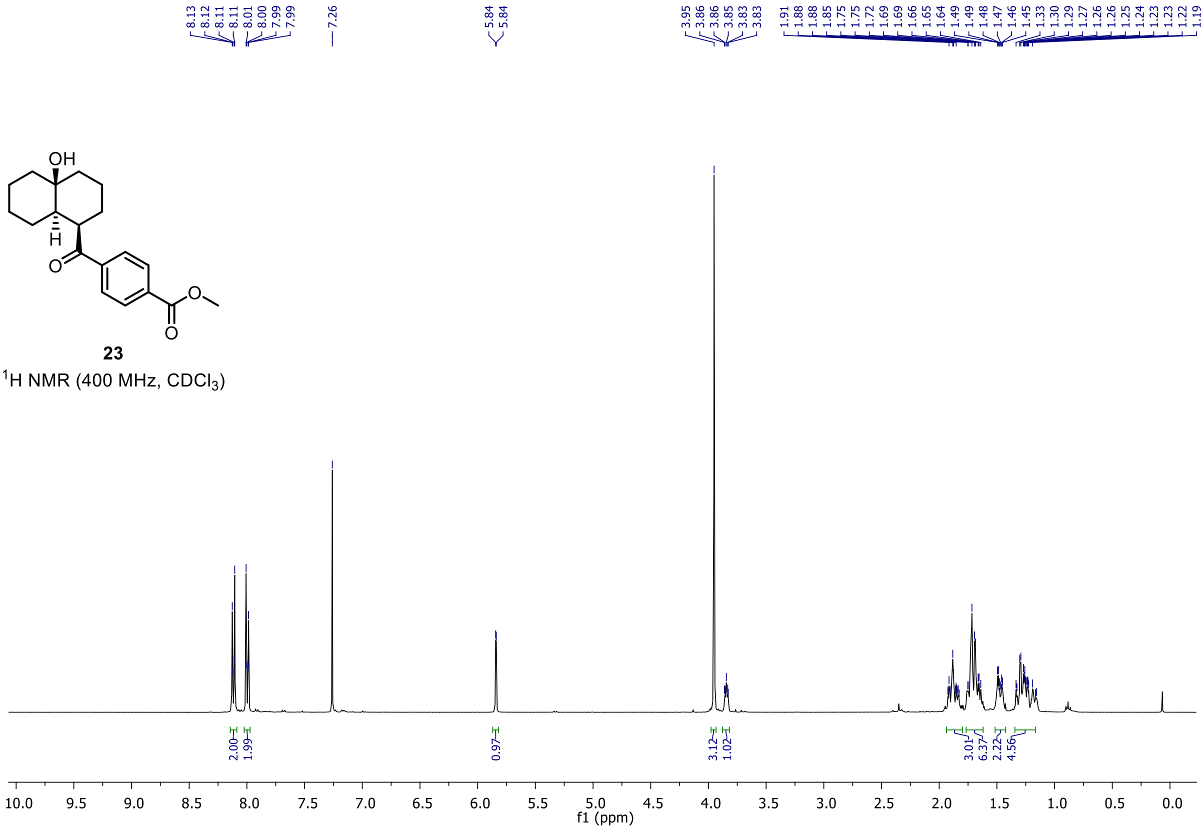

23

^{1}H NMR (400 MHz, $CDCl_3$)

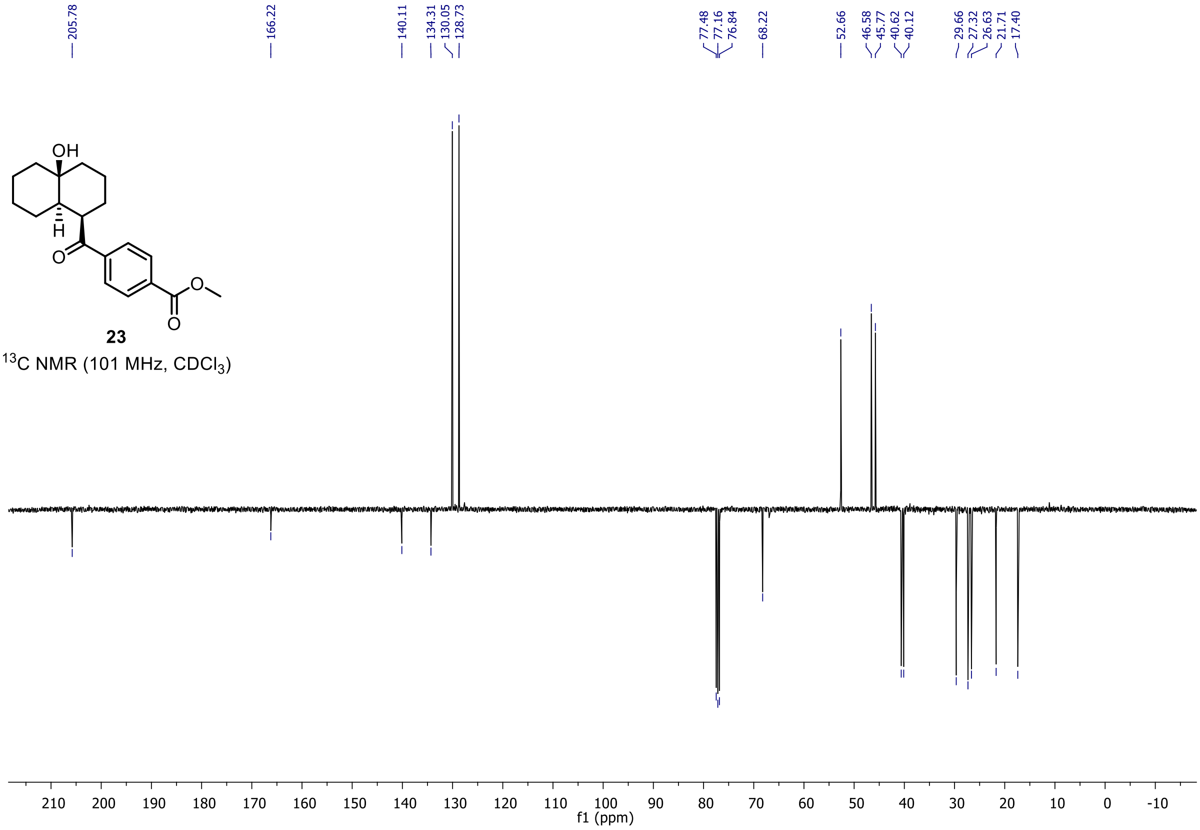

23

^{13}C NMR (101 MHz, $CDCl_3$)

(4-hydroxydecahydronaphthalen-1-yl)(4-nitrophenyl)methanone (24)

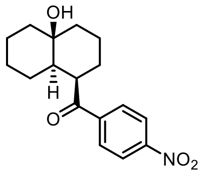

24

The title compound was prepared following **General Procedure B** using 4-nitrobenzoyl chloride (37 mg, 0.20 mmol, 1.00 equiv.), *cis*-decalin (93 µL, 0.60 mmol, 3.00 equiv.) and $AgSbF_6$ (72 mg, 0.21 mmol, 1.05 equiv.). Purification by flash column chromatography (0 – 30% Et_2O in pentane) gave **24** as a colorless solid (11.2 mg, 37%).

R_f = 0.31 in 20% EtOAc/heptanes

m.p. (+5 °C/min) = 164.7 – 167.6 °C

^{1}H NMR (400 MHz, $CDCl_3$) δ 8.31 (d, *J* = 8.9 Hz, 2H), 8.10 (d, *J* = 8.9 Hz, 2H), 5.58 (d, *J* = 2.3 Hz, 1H), 3.87 – 3.79 (m, 1H), 1.95 – 1.83 (m, 3H), 1.82 – 1.64 (m, 6H), 1.54 – 1.46 (m, 2H), 1.36 – 1.23 (m, 4H).

^{13}C NMR (101 MHz, $CDCl_3$) δ 204.8 (C), 150.6 (C), 141.5 (C), 129.9 (2CH), 124.1 (2CH), 68.3 (C), 46.9 (CH), 45.7 (CH), 40.5 (CH_2), 40.1 (CH_2), 29.5 (CH_2), 27.3 (CH_2), 26.7 (CH_2), 21.7 (CH_2), 17.3 (CH_2).

IR (neat) v_{max}: 3393, 2932, 2855, 1667, 1520, 1444, 1348, 1213, 958, 853, 713.

HRMS (ESI$^+$): exact mass calculated for $[M+Na]^+$ ($C_{17}H_{21}NO_4Na$) requires *m*/*z* 326.1363, found *m*/*z* 326.1370.

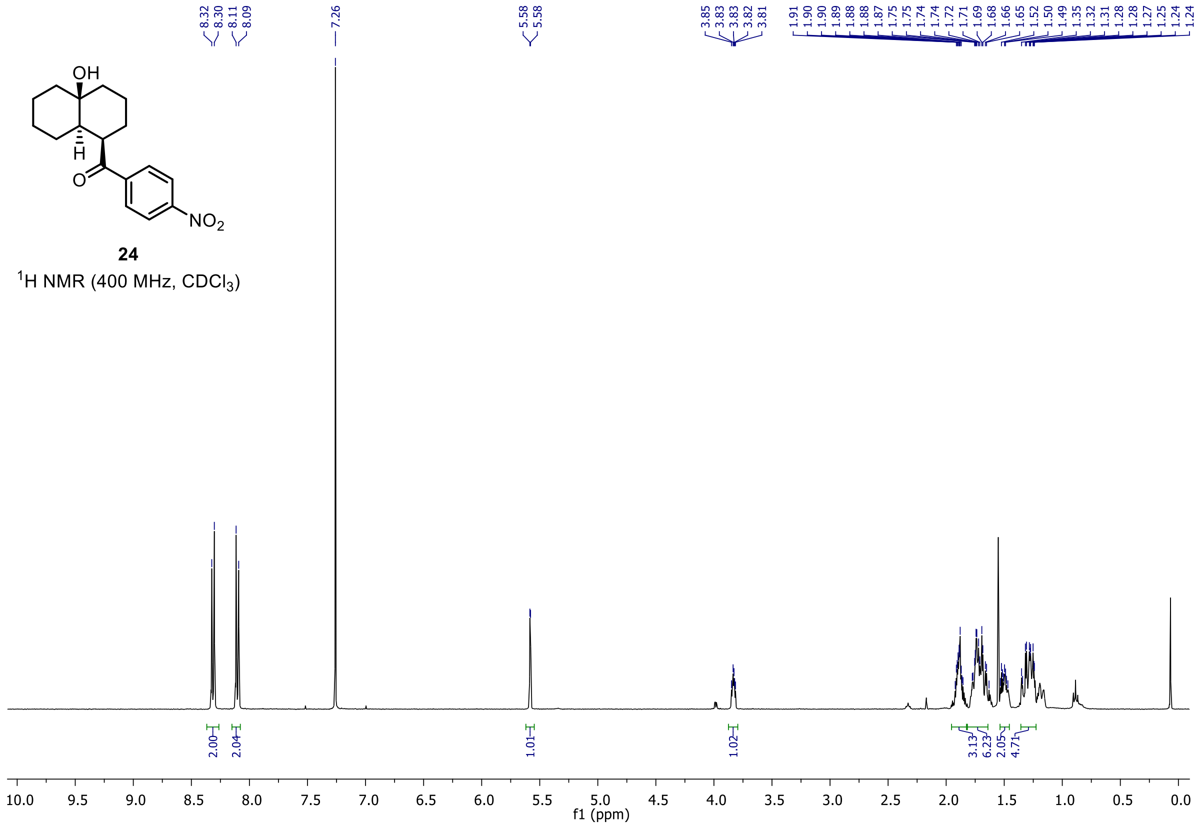

24

^1H NMR (400 MHz, $CDCl_3$)

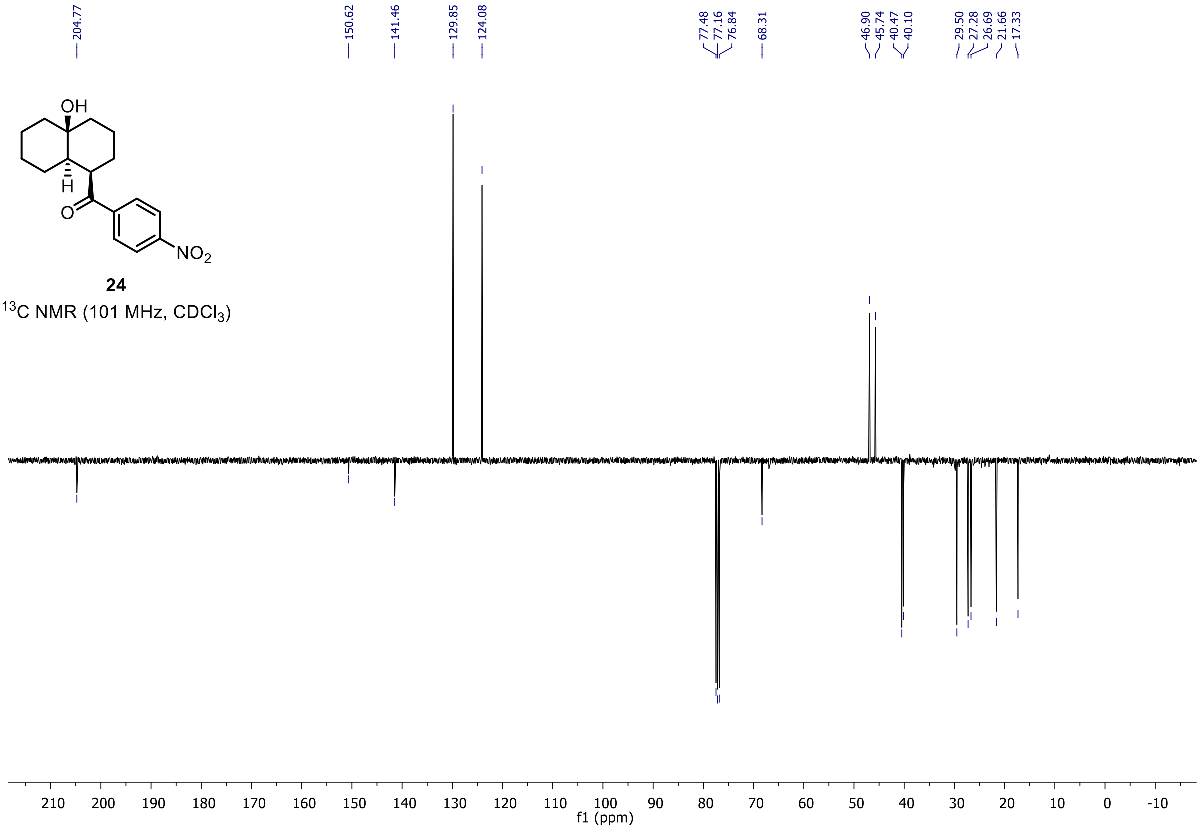

24

^{13}C NMR (101 MHz, $CDCl_3$)

General Procedure C: Modified Baddeley reaction for aliphatic acyl chlorides

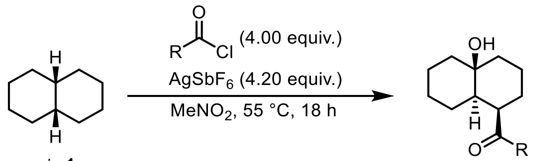

cis-**1**
(1.00 equiv.)

In a small flame-dried vial with a rubber septum cap, $AgSbF_6$ (144 mg, 0.42 mmol, 4.20 equiv., weighed in glovebox) was dissolved in 0.5 mL $MeNO_2$ at ambient temperature (23 °C) and then heated to 55 °C. Acyl chloride (0.40 mmol, 4.00 equiv.) was added under stirring at 55 °C, followed immediately by *cis*-decalin (15 µL, 0.10 mmol, 1.00 equiv.). The cap was sealed with multiple layers of parafilm[a] and the resulting suspension was then stirred for 18 hours at 55 °C. After cooling down to ambient temperature (23 °C), the reaction was stopped by adding 10% aqueous K_2CO_3 solution (2 mL) and stirring for 1 h at ambient temperature (23 °C). The mixture was transferred to a separatory funnel, diluted with more 10% aqueous K_2CO_3 solution (5 mL) and the aqueous layer was extracted with DCM (3 × 5 mL). The combined organic layers were dried over anhydrous sodium sulfate, the dried solution was filtered, and the filtrate was concentrated under reduced pressure (final drying for >30 min at <1 mbar at 23 °C to remove unreacted decalin and $MeNO_2$). The crude residue was purified by flash column chromatography to afford the desired compound.

[a]Corrosion of rubber septum caps was sometimes observed.

1-(4a-hydroxydecahydronaphthalen-1-yl)ethan-1-one (25a)

1-(1-hydroxyoctahydronaphthalen-4a(2H)-yl)ethan-1-one (25b)

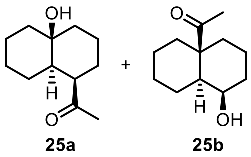

a:b = 10:1

The title compounds were prepared following **General Procedure C** using acetyl chloride (31 mg, 0.40 mmol, 4.00 equiv.), *cis*-decalin (14 mg, 0.10 mmol, 1.00 equiv.) and $AgSbF_6$ (144 mg, 0.42 mmol, 4.20 equiv.). Purification by flash column chromatography (0 – 25% EtOAc in heptanes) gave **25a** (7.4 mg, 38%) and **25b** (0.6 mg, 3%) as colorless oils. The ratio **a**:**b** was determined by ^{1}H NMR analysis of the crude reaction product using mesitylene as internal standard.

25a:

R_f = 0.39 in 20% EtOAc/heptanes

^{1}H NMR (400 MHz, $CDCl_3$) δ 5.57 (d, *J* = 2.1 Hz, 1H), 2.96 (t, *J* = 5.4 Hz, 1H), 2.21 (d, *J* = 0.5 Hz, 3H), 1.93 – 1.79 (m, 2H), 1.77 – 1.41 (m, 9H), 1.29 – 1.12 (m, 4H).

^{13}C NMR (101 MHz, $CDCl_3$) δ 215.9 (C), 68.2 (C), 52.3 (CH), 45.7 (CH), 40.7 (CH_2), 40.0 (CH_2), 31.0 (CH_3), 28.6 (CH_2), 27.3 (CH_2), 26.5 (CH_2), 21.7 (CH_2), 17.5 (CH_2).

IR (neat) v_{max}: 3391, 2927, 2855, 1691, 1447, 1416, 1354, 1185, 1166, 1142, 968, 952.

HRMS (ESI$^+$): exact mass calculated for $[M+Na]^+$ ($C_{12}H_{20}O_2Na$) requires *m*/*z* 219.1356, found *m*/*z* 219.1355.

25b:

All analytical data were in good accordance with the literature.[21]

R_f = 0.20 in 20% EtOAc/heptanes

^{1}H NMR (700 MHz, $CDCl_3$) δ 5.44 (d, *J* = 8.5 Hz, 1H), 3.71 (dd, *J* = 8.4, 3.1 Hz, 1H), 2.19 – 2.14 (m, 4H), 1.96 – 1.93 (m, 1H), 1.87 – 1.83 (m, 1H), 1.83 – 1.79 (m, 1H), 1.76 (dd, *J* = 13.3, 4.1 Hz, 1H), 1.70 – 1.67 (m, 1H), 1.51 – 1.47 (m, 1H), 1.44 – 1.40 (m, 3H), 1.30 – 1.25 (m, 4H), 1.12 – 1.04 (m, 1H).

^{13}C NMR (176 MHz, $CDCl_3$) δ 217.7 (C), 69.2 (CH), 56.3 (C), 49.1 (CH), 39.7 (CH_2), 37.2 (CH_2), 35.3 (CH_2), 27.2 (CH_2), 26.7 (CH_2), 25.9 (CH_3), 24.2 (CH_2), 17.4 (CH_2).

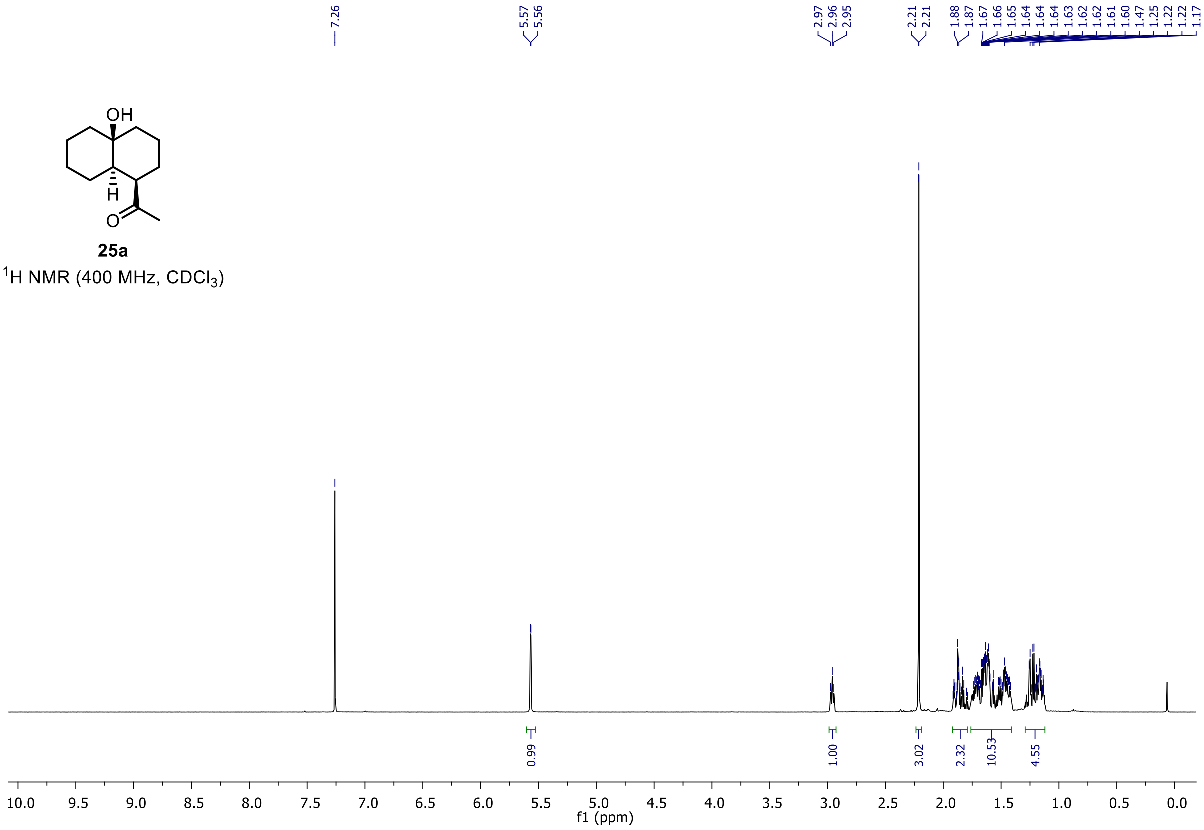

25a

^{1}H NMR (400 MHz, $CDCl_3$)

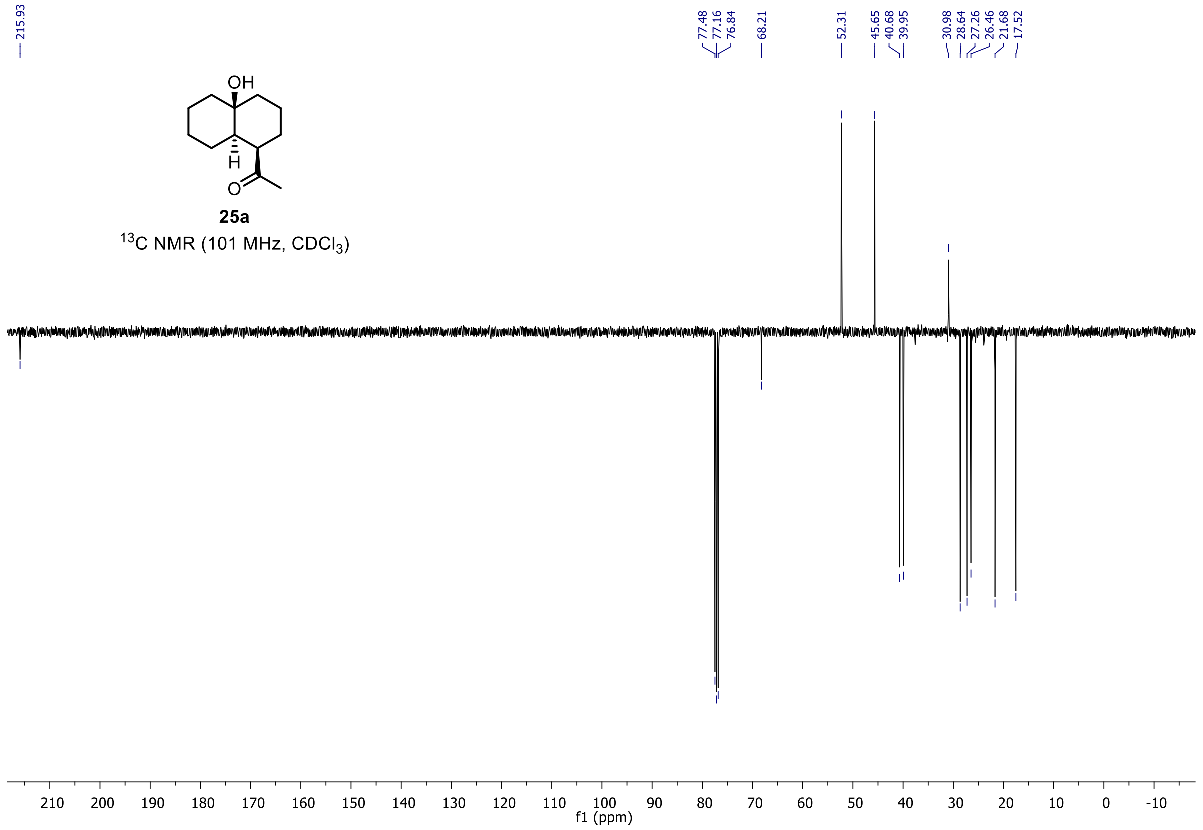

25a

^{13}C NMR (101 MHz, $CDCl_3$)

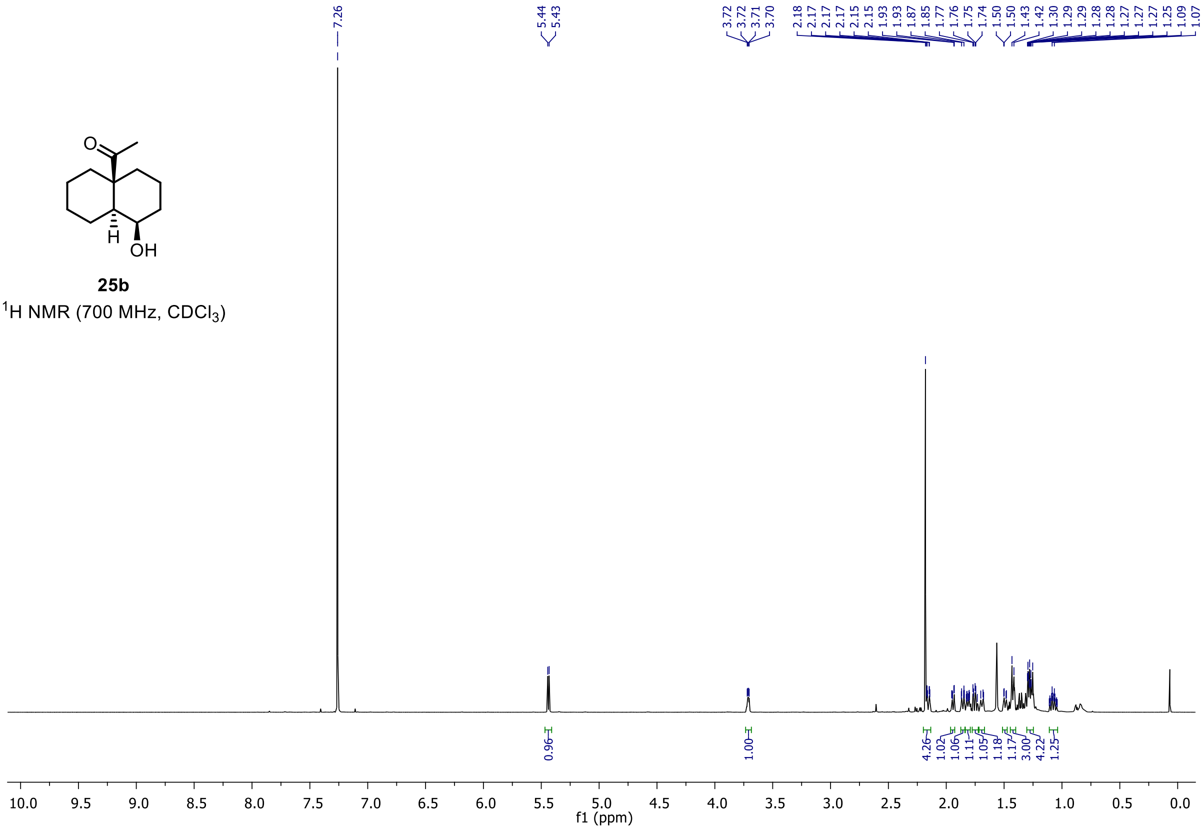
25b
^{1}H NMR (700 MHz, CDCl$_3$)
f1 (ppm)

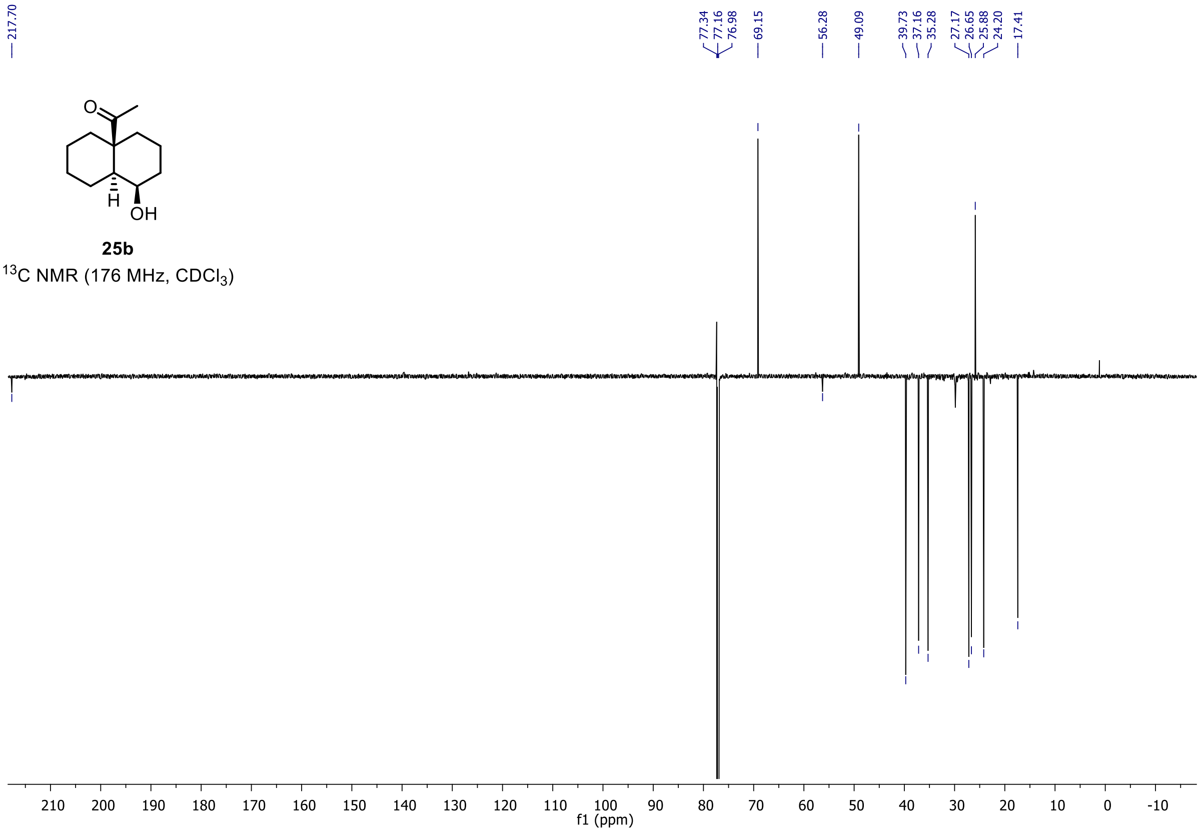
25b
^{13}C NMR (176 MHz, CDCl$_3$)
f1 (ppm)

1-(4a-hydroxydecahydronaphthalen-1-yl)propan-1-one (26a)

1-(1-hydroxyoctahydronaphthalen-4a(2H)-yl)propan-1-one (26b)

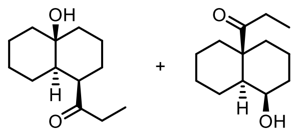

26a **26b**

a:b = 11:1

The title compounds were prepared following **General Procedure C** using propionyl chloride (37 mg, 0.40 mmol, 4.00 equiv.), *cis*-decalin (14 mg, 0.10 mmol, 1.00 equiv.) and $AgSbF_6$ (144 mg, 0.42 mmol, 4.20 equiv.). Purification by flash column chromatography (0 – 20% EtOAc in heptanes) gave **26a** (11 mg, 52%) as a colorless oil. The ratio **a**:**b** was determined by ^{1}H NMR analysis of the crude reaction product using mesitylene as internal standard. **26b** could not be isolated, its presence was proven by ^{1}H NMR analysis of the crude reaction product.

26a:

R_f = 0.38 in 20% EtOAc/heptanes

^{1}H NMR (400 MHz, $CDCl_3$) δ 5.70 (d, *J* = 2.4 Hz, 1H), 2.94 (dd, *J* = 8.0, 3.2 Hz, 1H), 2.64 (dq, *J* = 17.8, 7.2 Hz, 1H), 2.37 (dq, *J* = 17.8, 7.3 Hz, 1H), 1.91 – 1.78 (m, 2H), 1.76 – 1.30 (m, 10H), 1.23 – 1.12 (m, 3H), 1.04 (t, *J* = 7.3 Hz, 3H).

^{13}C NMR (101 MHz, $CDCl_3$) δ 218.4 (C), 68.1 (C), 51.2 (CH), 45.4 (CH), 40.6 (CH_2), 39.9 (CH_2), 36.9 (CH_2), 28.7 (CH_2), 27.2 (CH_2), 26.5 (CH_2), 21.7 (CH_2), 17.5 (CH_2), 8.1 (CH_3).

IR (neat) v_{max}: 3397, 2927, 2857, 2833, 1776, 1708, 1447, 1169, 1104, 930.

HRMS (ESI$^+$): exact mass calculated for $[M+Na]^+$ ($C_{13}H_{22}O_2Na$) requires *m*/*z* 233.1512, found *m*/*z* 233.1510.

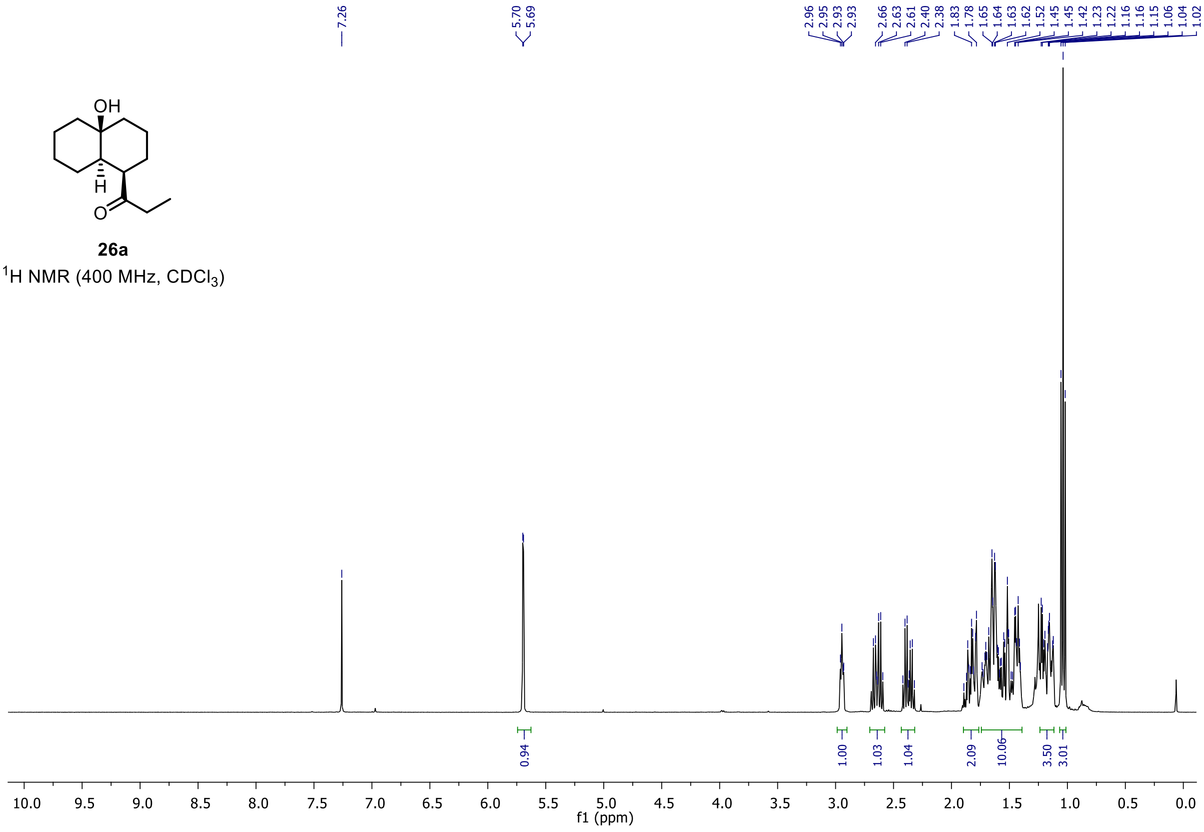

26a

^{1}H NMR (400 MHz, $CDCl_3$)

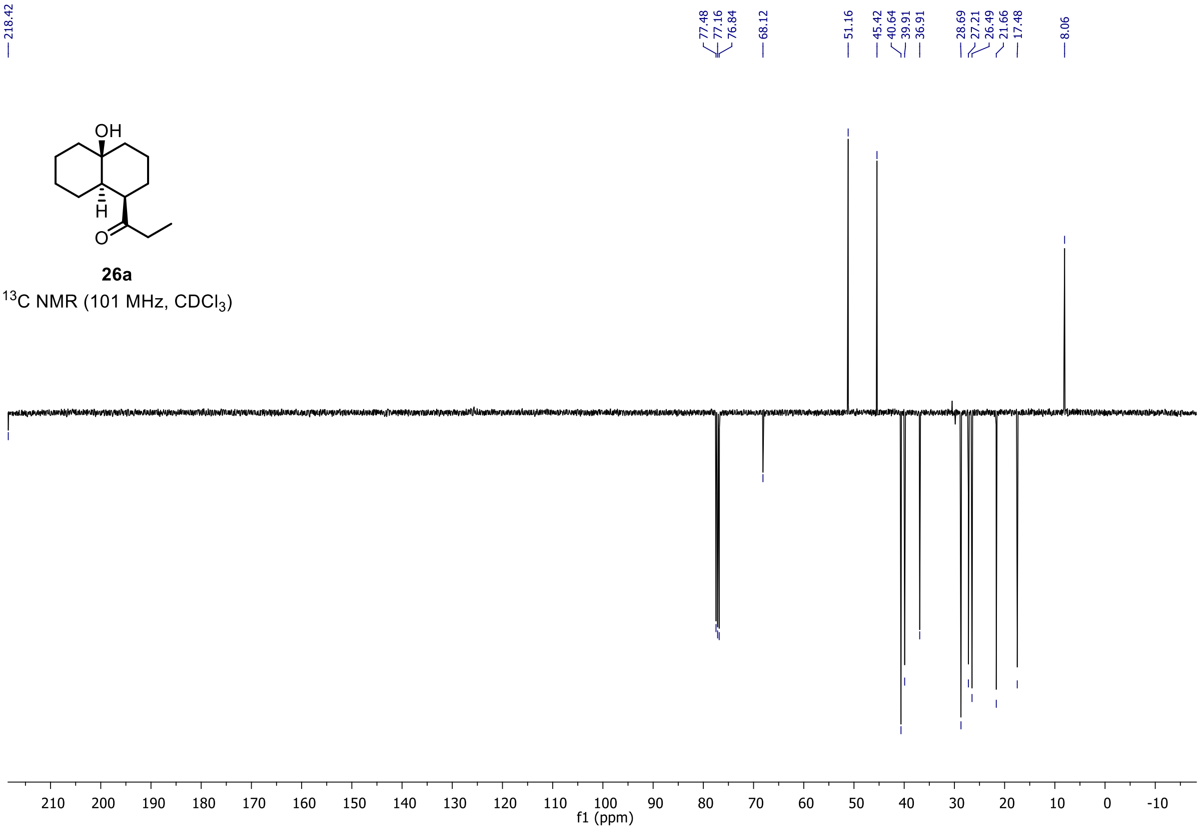

26a

^{13}C NMR (101 MHz, $CDCl_3$)

1-(-hydroxydecahydronaphthalen-1-yl)dodecan-1-one (27)

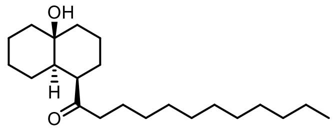

27

The title compounds were prepared following **General Procedure C** using lauroyl chloride (88 mg, 0.40 mmol, 4.00 equiv.), *cis*-decalin (14 mg, 0.10 mmol, 1.00 equiv.) and $AgSbF_6$ (144 mg, 0.42 mmol, 4.20 equiv.). Purification by flash column chromatography (0 – 15% EtOAc in heptanes) gave **27** (15 mg, 45%) as a colorless oil.

$\mathbf{R_f}$ = 0.57 in 20% EtOAc/heptanes

^{1}H NMR (400 MHz, $CDCl_3$) δ 5.74 (d, *J* = 2.3 Hz, 1H), 2.93 (dd, *J* = 8.2, 3.2 Hz, 1H), 2.60 – 2.50 (m, 1H), 2.37 (ddd, *J* = 17.1, 8.0, 6.3 Hz, 1H), 1.85 (ddd, *J* = 17.3, 13.3, 9.5 Hz, 2H), 1.76 – 1.40 (m, 12H), 1.33 – 1.10 (m, 19H), 0.87 (t, *J* = 6.8 Hz, 3H).

^{13}C NMR (101 MHz, $CDCl_3$) δ 218.1 (C), 68.1 (C), 51.5 (CH), 45.5 (CH), 43.6 (CH_2), 40.7 (CH_2), 39.9 (CH_2), 32.1 (CH_2), 29.7 ($2CH_2$), 29.6 (CH_2), 29.53 (CH_2), 29.47 (CH_2), 29.3 (CH_2), 28.6 (CH_2), 27.2 (CH_2), 26.5 (CH_2), 24.0 (CH_2), 22.8 (CH_2), 21.7 (CH_2), 17.5 (CH_2), 14.3 (CH_3)

IR (neat) ν_{max}: 3396, 2921, 2852, 1694, 1448, 1143, 1107, 960.

HRMS (ESI$^+$): exact mass calculated for $[M+Na]^+$ ($C_{22}H_{40}O_2Na$) requires *m*/*z* 359.2921, found *m*/*z* 359.2919.

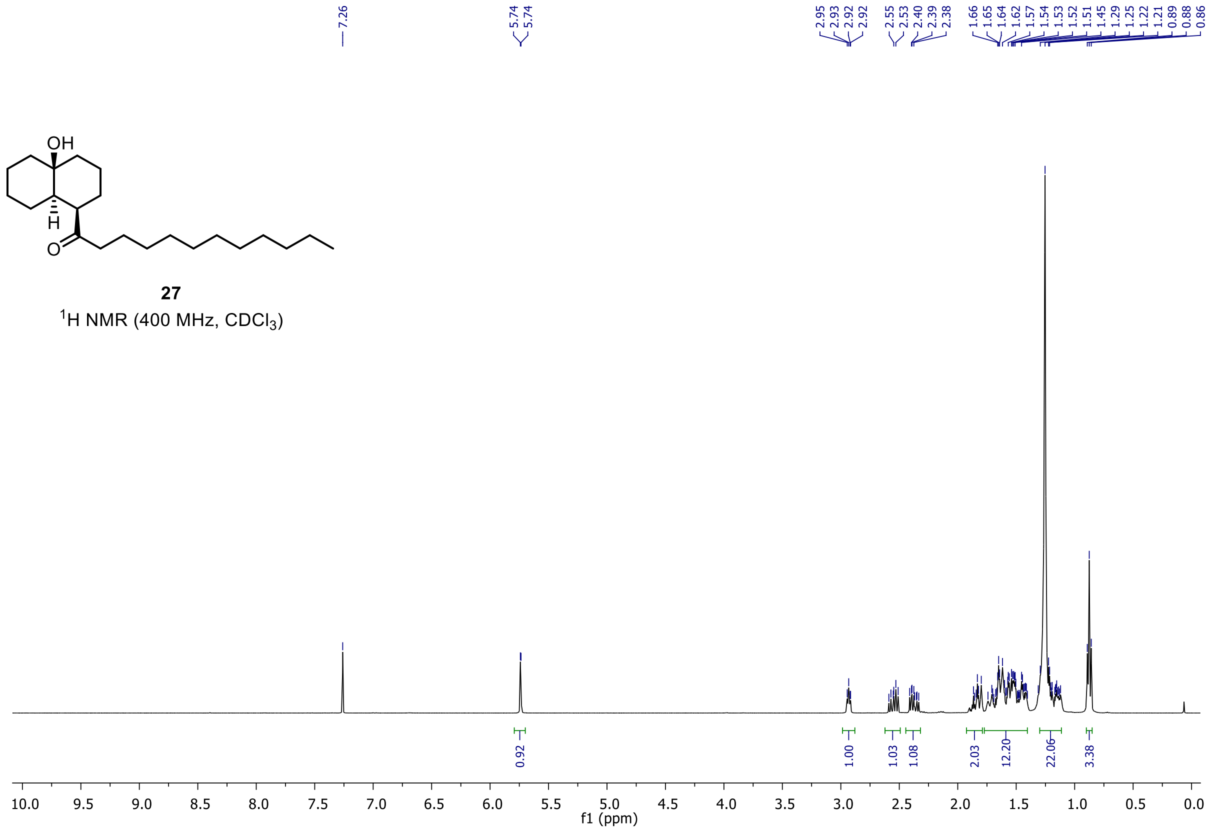
7.26
5.74
5.74
2.95
2.93
2.92
2.92
2.55
2.53
2.40
2.39
2.38
1.66
1.65
1.64
1.62
1.57
1.54
1.53
1.52
1.51
1.45
1.29
1.25
1.22
1.21
0.89
0.88
0.86
OH
H
O
27
^{1}H NMR (400 MHz, $CDCl_3$)
0.92
1.00
1.03
1.08
2.03
12.20
22.06
3.38
10.0 9.5 9.0 8.5 8.0 7.5 7.0 6.5 6.0 5.5 5.0 4.5 4.0 3.5 3.0 2.5 2.0 1.5 1.0 0.5 0.0
f1 (ppm)

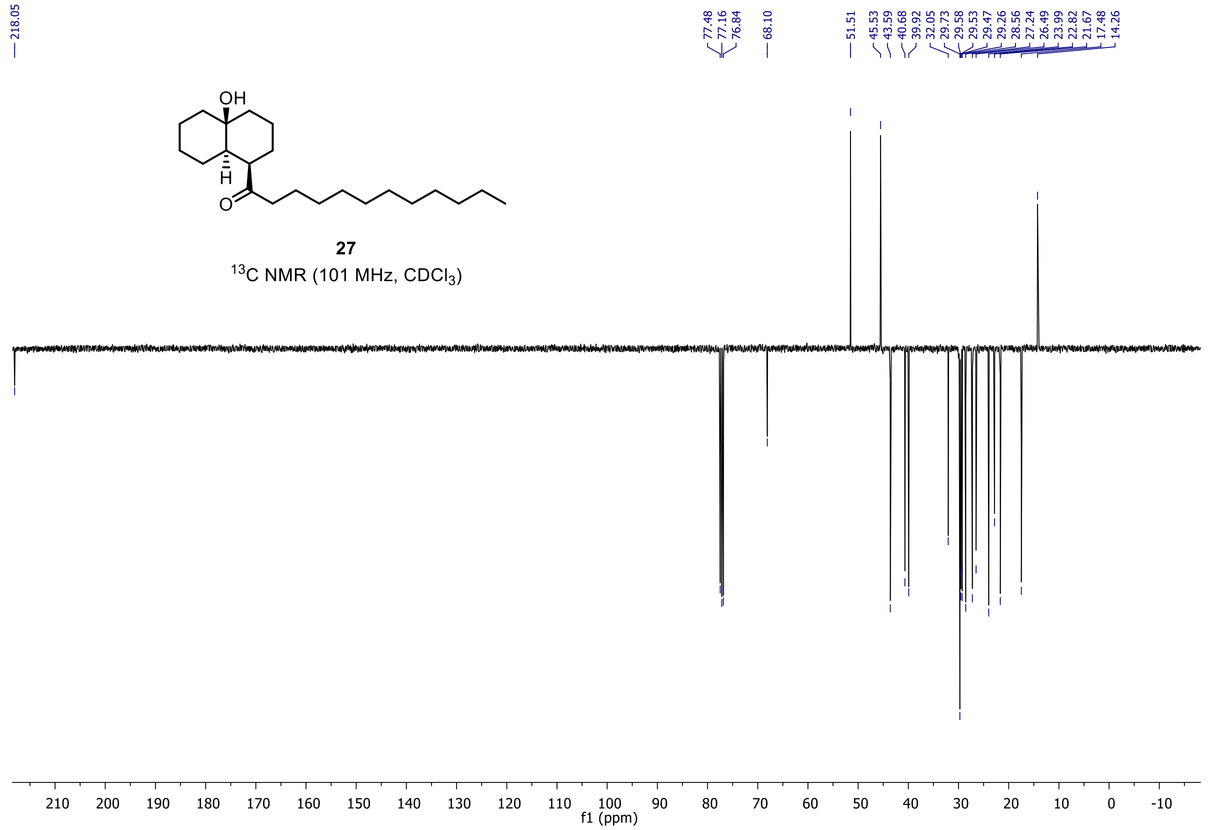
218.05
77.48
77.16
76.84
68.10
51.51
45.53
43.59
40.68
39.92
32.05
29.73
29.58
29.53
29.47
29.26
28.56
27.24
26.49
23.99
22.82
21.67
17.48
14.26
OH
H
O
27
^{13}C NMR (101 MHz, $CDCl_3$)
210 200 190 180 170 160 150 140 130 120 110 100 90 80 70 60 50 40 30 20 10 0 -10
f1 (ppm)

4.5 Large scale preparation of 12

(4-hydroxydecahydronaphthalen-1-yl)(p-tolyl)methanone (12)

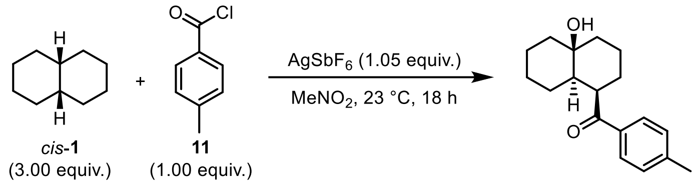

12

In a flame-dried Schlenk flask equipped with a stirring bar and a septum, $AgSbF_6$ (1.17 g, 3.40 mmol, 1.05 equiv., weighed in glovebox) was dissolved in 7 mL $MeNO_2$ at ambient temperature (23 °C). Acyl chloride (0.50 g, 3.23 mmol, 1.00 equiv.) was added slowly over 5 minutes under stirring at ambient temperature (23 °C), followed by *cis*-decalin (1.50 mL, 9.70 mmol, 3.00 equiv.). The resulting suspension was then stirred vigorously (700 rpm) at ambient temperature (23 °C) over 18 hours. The mixture was then cooled to 0 °C, the reaction was stopped by adding 10% aqueous K_2CO_3 solution (10 mL) and the black suspension was stirred for 1 h at ambient temperature (23 °C). The mixture was transferred to a separatory funnel, diluted with more 10% aqueous K_2CO_3 solution (30 mL) and the aqueous layer was extracted with DCM (3 × 30 mL). The combined organic layers were dried over anhydrous potassium carbonate, the dried solution was filtered, and the filtrate was concentrated under reduced pressure (final drying for >5 h at <1 mbar at 23 °C to remove unreacted decalin and $MeNO_2$). Purification by flash column chromatography (0 – 20% Et_2O in pentane) afforded **12** as a light-yellow crystalline solid (310 mg, 70%).

All analytical data were identical to those reported in Section **4.4** (compound **12**).

4.6 Modified Baddeley reaction with different alkane substrates

(3-hydroxy-2-methylcyclohexyl)(p-tolyl)methanone (35)

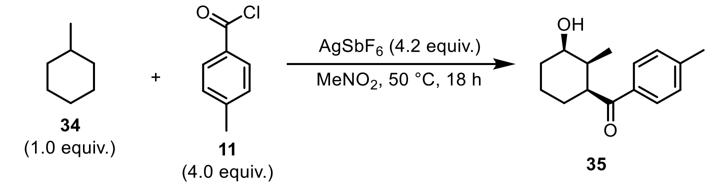

In a small flame-dried Schlenk flask equipped with a stirring bar and a septum, $AgSbF_6$ (433 mg, 1.26 mmol, 4.20 equiv., weighed in glovebox) was dissolved in 1.5 mL $MeNO_2$ at ambient temperature (23 °C). *p*-Toluoyl chloride (186 mg, 1.20 mmol, 4.00 equiv.) was added under stirring at ambient temperature, followed immediately by methylcyclohexane (38 µL, 0.30 mmol, 1.00 equiv.). The resulting suspension was placed into a heating block (pre-heated to 50 °C) and stirred for 18 hours at 50 °C. After cooling down to ambient temperature (23 °C), the reaction was stopped by adding 10% aqueous K_2CO_3 solution (5 mL) and stirring for 1 h at ambient temperature (23 °C). The mixture was transferred to a separatory funnel, diluted with more 10% aqueous K_2CO_3 solution (5 mL) and the aqueous layer was extracted with DCM (3 × 20 mL). The combined organic layers were dried over anhydrous sodium sulfate, the dried solution was filtered, and the filtrate was concentrated under reduced pressure (final drying for >30 min at <1 mbar at 23 °C to remove unreacted decalin and $MeNO_2$). The crude residue was purified by flash column chromatography (10 – 60% EtOAc in heptanes) to afford **35** contaminated with a minor amount of an unknown impurity. Second purification by preparative TLC (60% Et_2O in pentane) afforded pure **35** as a colorless oil (23 mg, 33%).

Stereochemical assignment of this compound was done by comparison of the spectroscopic data with those described in literature for a highly similar compound (aromatic rest being Ph instead of *p*-Me-Ph).[26] This was further confirmed by a 2D NMR (NOESY and COSY) analysis.

R_f = 0.45 in 50% EtOAc/heptanes; 0.24 in 50% Et_2O/pentane

The acidic OH proton was observed as a broad singlet in ^1H NMR, partially overlapping with the 3.67 – 3.61 multiplet.

^1H NMR (600 MHz, $CDCl_3$) δ 7.84 (d, *J* = 8.2 Hz, 2H), 7.26 (d, *J* = 8.2 Hz, 2H), 3.86 (dt, *J* = 7.5, 3.9 Hz, 1H), 3.67 – 3.61 (m, 1H), 3.59 (br s, 1H), 2.41 (s, 3H), 2.26 – 2.18 (m, 1H), 1.87 – 1.79 (m, 1H), 1.73 – 1.63 (m, 3H), 1.62 – 1.56 (m, 1H), 1.41 – 1.34 (m, 1H), 0.87 (d, *J* = 7.2 Hz, 3H).

Due to conformational changes, some ^{13}C NMR resonances were observed as broad and weak. One signal for the cyclohexyl-bound CH_3 was completely absent (detected only in HSQC). Signal broadening was more pronounced at 151 MHz than 101 MHz.

^{13}C NMR (101 MHz, $CDCl_3$) δ 203.3 (C), 144.2 (C), 133.9 (C), 129.5 (2CH), 128.8 (2CH), 70.9 (CH), 47.5 (CH), 37.3 (CH), 31.7 (CH_2), 24.8 (CH_2), 21.8 (CH_3), 19.3 (CH_2), 11.0 (CH_3, visible only in HSQC).

IR (neat) v_{max}: 3399, 2931, 2867, 1731, 1671, 1605, 1264, 1229, 1181, 1013, 788, 768.

HRMS (ESI$^+$): exact mass calculated for $[M+Na]^+$ ($C_{15}H_{20}O_2Na$) requires *m*/*z* 255.1356, found *m*/*z* 255.1353.

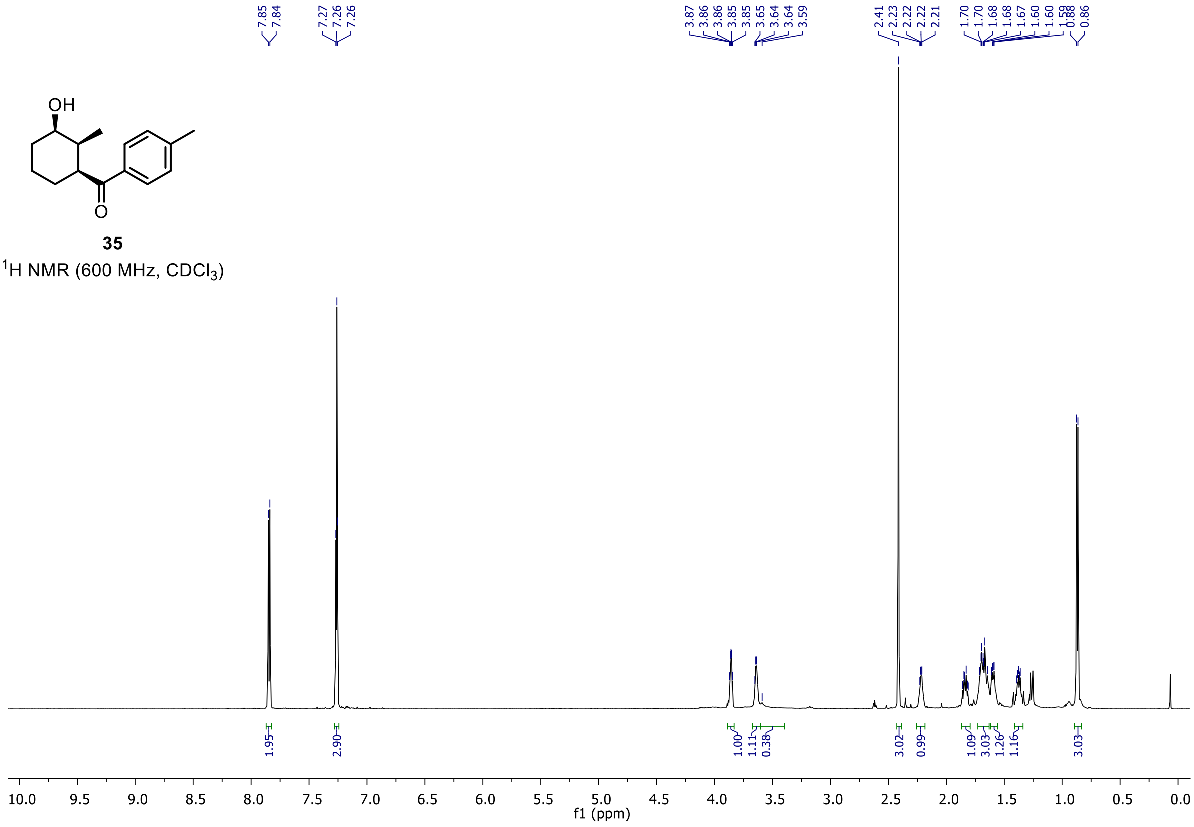

35

^{1}H NMR (600 MHz, CDCl$_3$)

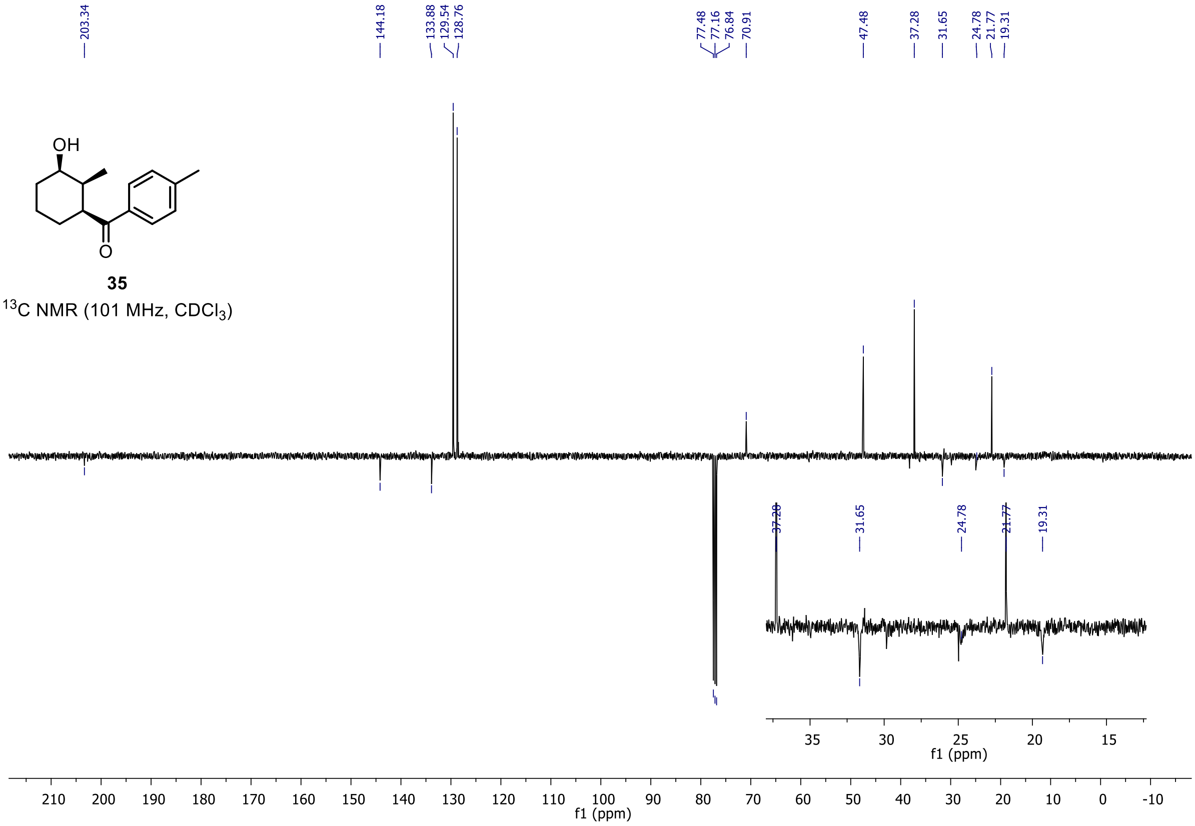

35

^{13}C NMR (101 MHz, CDCl$_3$)

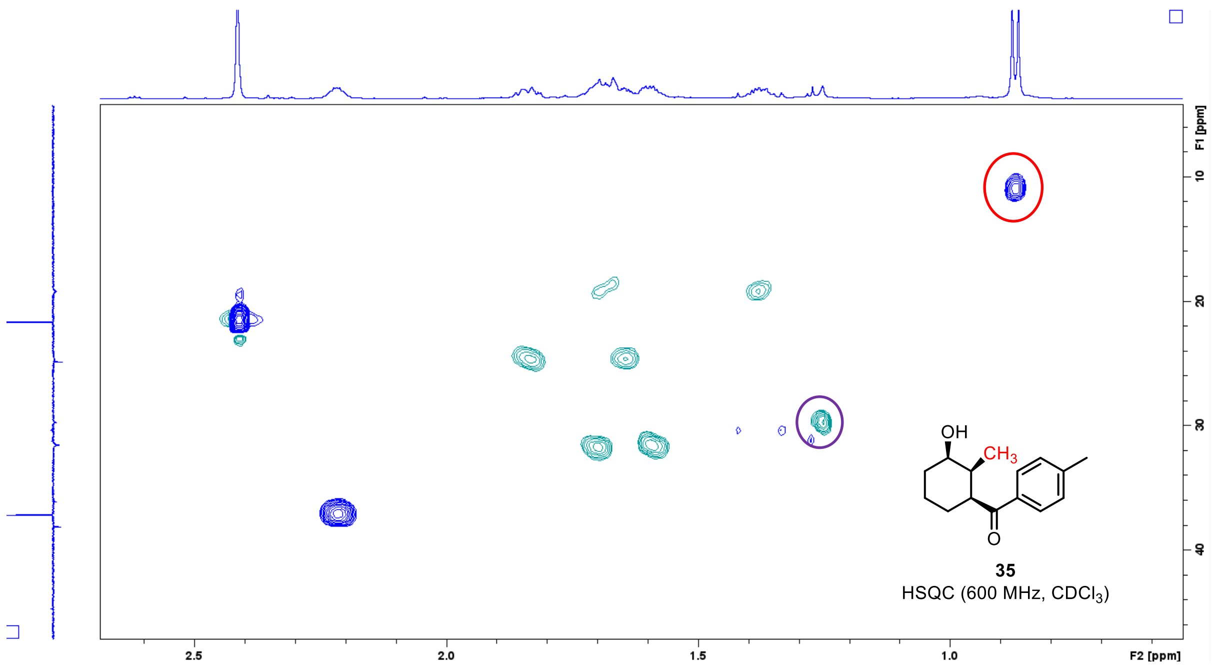

Multiplicity-edited HSQC (blue = CH/CH_3, green = CH_2). ***Highlighted in:*** *Red: CH_3 carbon signal, missing in ^{13}C NMR due to conformational changes. Purple: signal of grease.[37]*

(3-hydroxy-2,3-dimethylcyclohexyl)(p-tolyl)methanone (37)

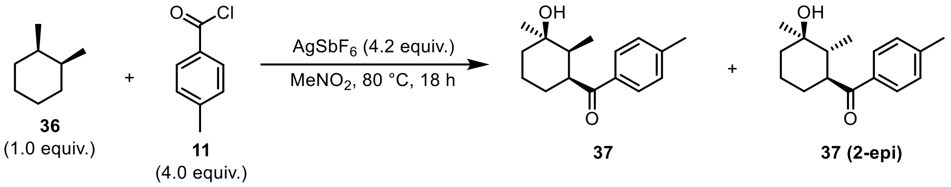

In a small flame-dried Schlenk flask equipped with a stirring bar and a septum, $AgSbF_6$ (433 mg, 1.26 mmol, 4.20 equiv., weighed in glovebox) was dissolved in 1.5 mL $MeNO_2$ at ambient temperature (23 °C). *p*-Toluoyl chloride (186 mg, 1.20 mmol, 4.00 equiv.) was added under stirring at ambient temperature, followed immediately by *cis*-1,2-dimethylcyclohexane (42 µL, 0.30 mmol, 1.00 equiv.). The resulting suspension was placed into a heating block (pre-heated to 80 °C) and stirred for 18 hours at 80 °C. After cooling down to ambient temperature (23 °C), the reaction was stopped by adding 10% aqueous K_2CO_3 solution (5 mL) and stirring for 1 h at ambient temperature (23 °C). The mixture was transferred to a separatory funnel, diluted with more 10% aqueous K_2CO_3 solution (5 mL) and the aqueous layer was extracted with DCM (3 × 20 mL). The combined organic layers were dried over anhydrous sodium sulfate, the dried solution was filtered, and the filtrate was concentrated under reduced pressure (final drying for >30 min at <1 mbar at 23 °C to remove unreacted decalin and $MeNO_2$). *dr* was determined by ^{1}H NMR analysis of the crude reaction product using mesitylene as internal standard (**37**:**37 (2-epi**) = 2.8:1). Purification by flash column chromatography (10 – 60% EtOAc in heptanes) afforded a mixture of **37** and **37 (2-epi)** (31 mg, 42%, **37:37 (2-epi)** = 2.7:1). Separation of diastereomers was achieved by subjecting an analytical sample (15 mg) to preparative TLC (30% Et_2O in pentane), affording pure **37** and **37 (2-epi)** as colorless oils.

R_f = 0.63 (**37**) and 0.61 (**37 (2-epi)**) in 50% EtOAc/heptanes

Data for **37**:

^{1}H NMR (700 MHz, CDCl$_3$) δ 7.86 (d, *J* = 8.2 Hz, 2H), 7.26 (d, *J* = 8.2 Hz, 3H), 5.10 (br s, 1H), 3.83 (dd, *J* = 10.1, 5.1 Hz, 1H), 2.41 (s, 3H), 1.91 – 1.83 (m, 2H), 1.80 – 1.76 (m, 1H), 1.75 – 1.70 (m, 1H), 1.66 – 1.59 (m, 1H), 1.45 – 1.39 (m, 2H), 1.29 (s, 3H), 0.97 (d, *J* = 7.2 Hz, 3H).

^{13}C NMR (176 MHz, CDCl$_3$) δ 204.8 (C), 144.4 (C), 134.1 (C), 129.6 (2CH), 128.9 (2CH), 69.8 (C), 47.2 (CH), 41.5 (CH), 39.8 (CH_2), 28.3 (CH), 27.8 (CH_2), 21.8 (CH_3), 18.6 (CH_2), 12.4 (CH_3).

IR (neat) v_{max}: 3358, 2926, 2855, 1663, 1606, 1459, 1410, 1374, 1226, 1182, 743.

HRMS (ESI$^+$): exact mass calculated for $[M+Na]^+$ ($C_{16}H_{22}O_2Na$) requires *m*/*z* 269.1512, found *m*/*z* 269.1512.

Data for **37 (2-epi)**:

^{1}H NMR (600 MHz, CDCl$_3$) δ 7.85 (d, *J* = 8.2 Hz, 2H), 7.27 (d, *J* = 8.2 Hz, 2H), 3.22 (td, *J* = 11.0, 3.6 Hz, 1H), 2.42 (s, 3H), 2.00 (dq, *J* = 10.5, 6.7 Hz, 1H), 1.84 – 1.78 (m, 3H), 1.53 – 1.46 (m, 3H), 1.20 (s, 3H), 0.87 (d, *J* = 6.7 Hz, 3H).

^{13}C NMR (101 MHz, CDCl$_3$) δ 203.2 (C), 144.1 (C), 135.0 (C), 129.5 (2CH), 128.5 (2CH), 72.9 (C), 49.3 (CH), 43.9 (CH), 41.9 (CH_2), 30.7 (CH_2), 23.5 (CH_2), 21.8 (CH_3), 21.1 (CH), 13.1 (CH_3).

IR (neat) v_{max}: 3458, 2927, 2861, 1670, 1605, 1283, 1180, 1114, 908, 876, 744.

HRMS (ESI$^+$): exact mass calculated for $[M+Na]^+$ ($C_{16}H_{22}O_2Na$) requires *m*/*z* 269.1512, found *m*/*z* 269.1512.

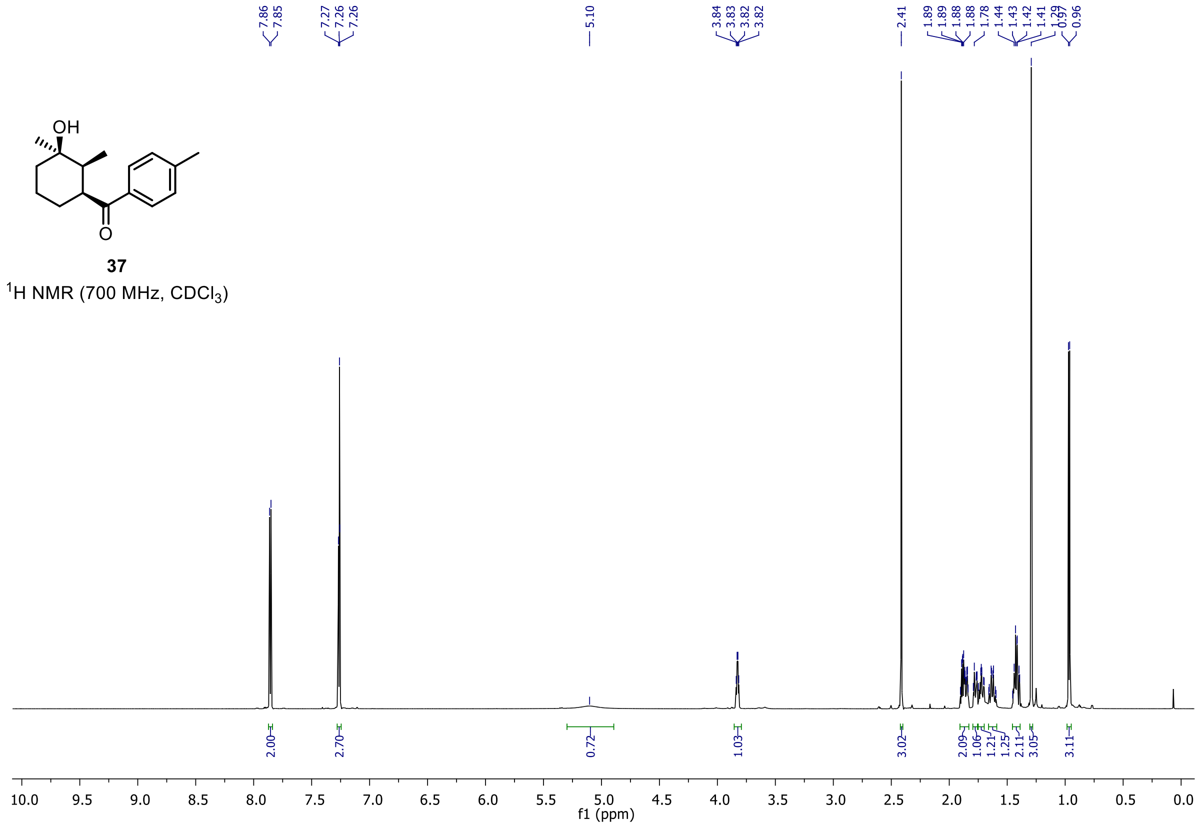
7.86
7.85
7.27
7.26
7.26
5.10
3.84
3.83
3.82
3.82
2.41
1.89
1.89
1.88
1.88
1.78
1.44
1.43
1.42
1.41
1.29
0.97
0.96
37
¹H NMR (700 MHz, CDCl₃)
2.00
2.70
0.72
1.03
3.02
2.09
1.06
1.21
1.25
2.11
3.05
3.11
f1 (ppm)

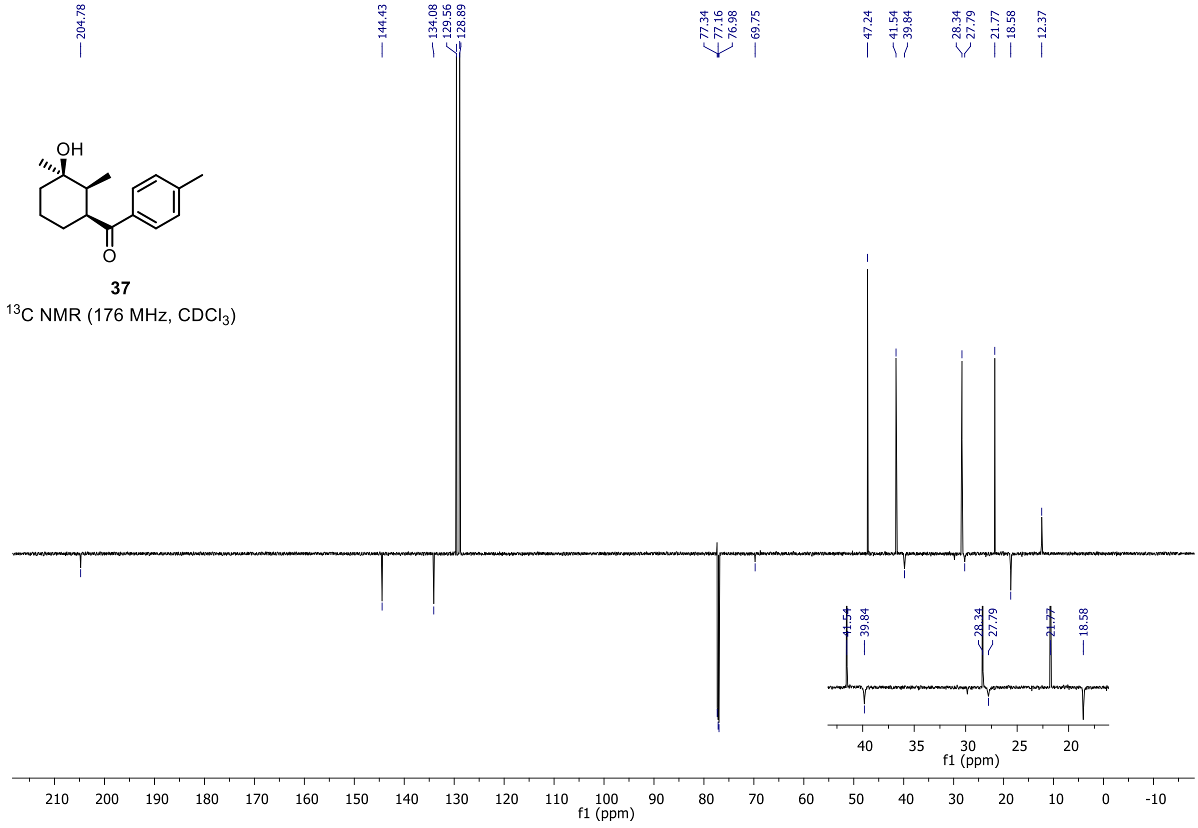
204.78
144.43
134.08
129.56
128.89
77.34
77.16
76.98
69.75
47.24
41.54
39.84
28.34
27.79
21.77
18.58
12.37
37
¹³C NMR (176 MHz, CDCl₃)
f1 (ppm)

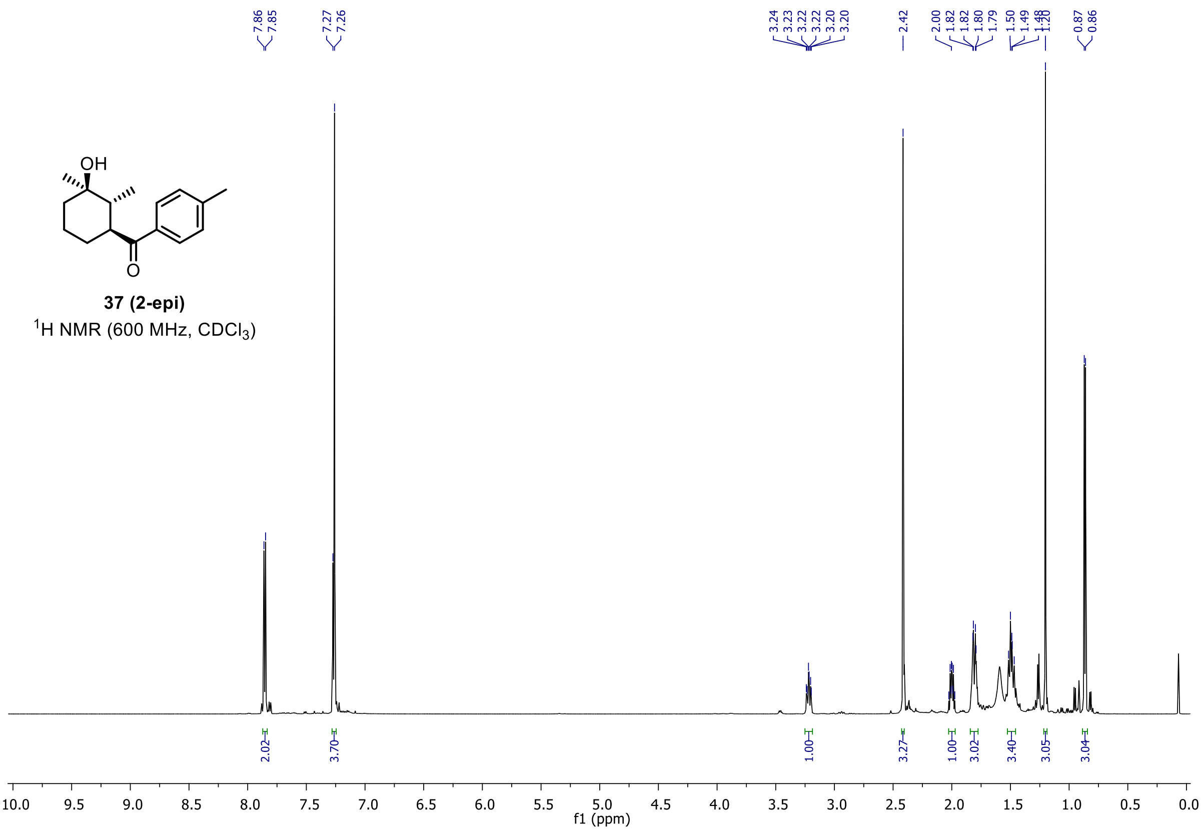

Broad singlet at 1.58 ppm belongs to water. The acidic OH signal could not be observed.

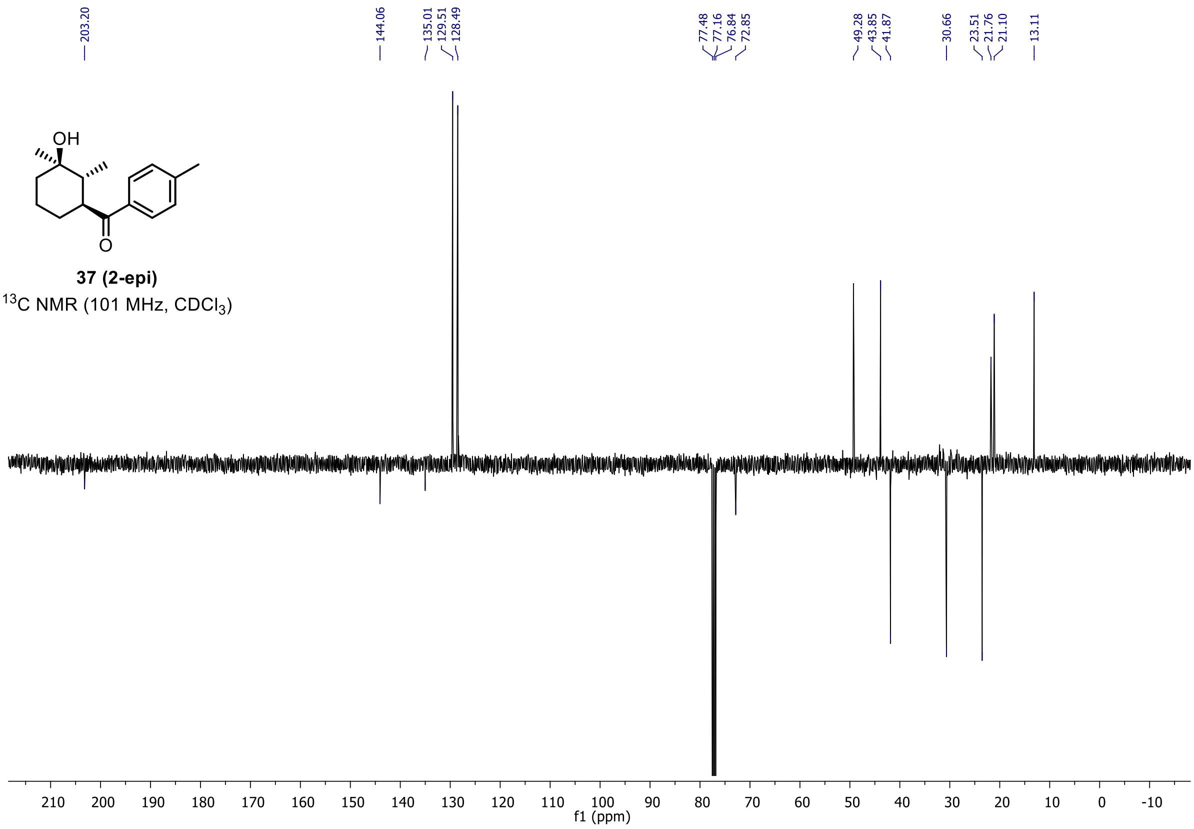

Stereochemical assignment of **37** and **37 (2-epi)**:

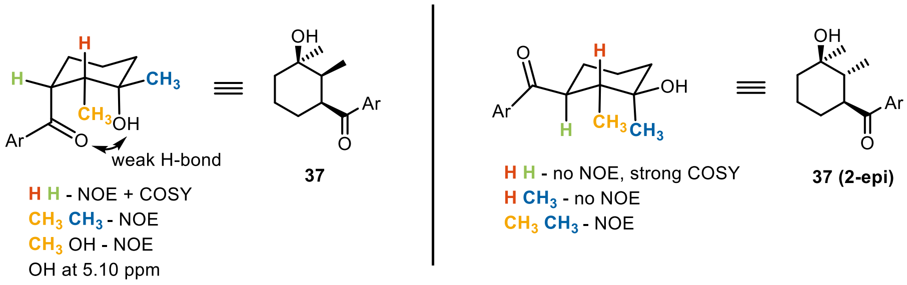

Evidence for **37**:

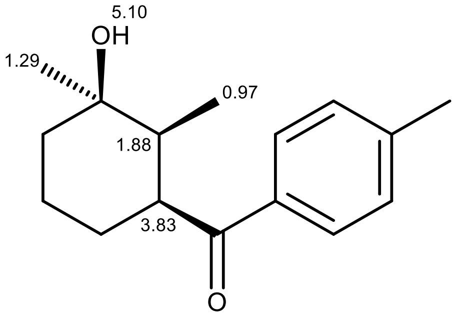

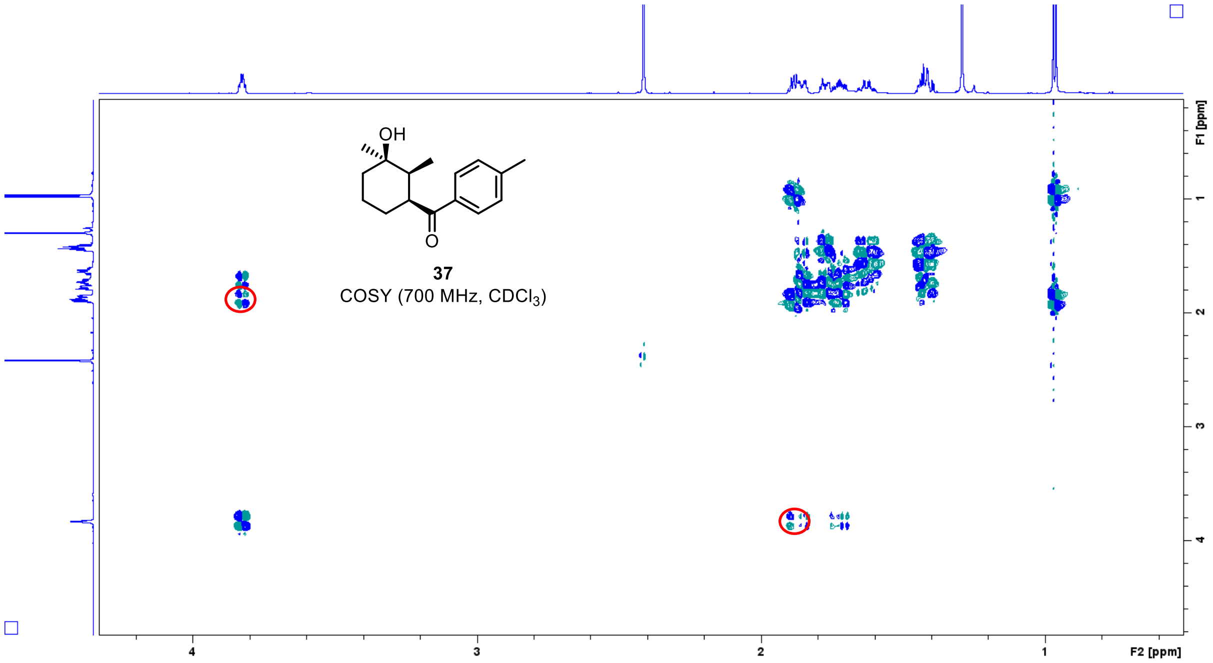

Highlighted are crosspeaks between 3.83 ppm + 1.88 ppm.

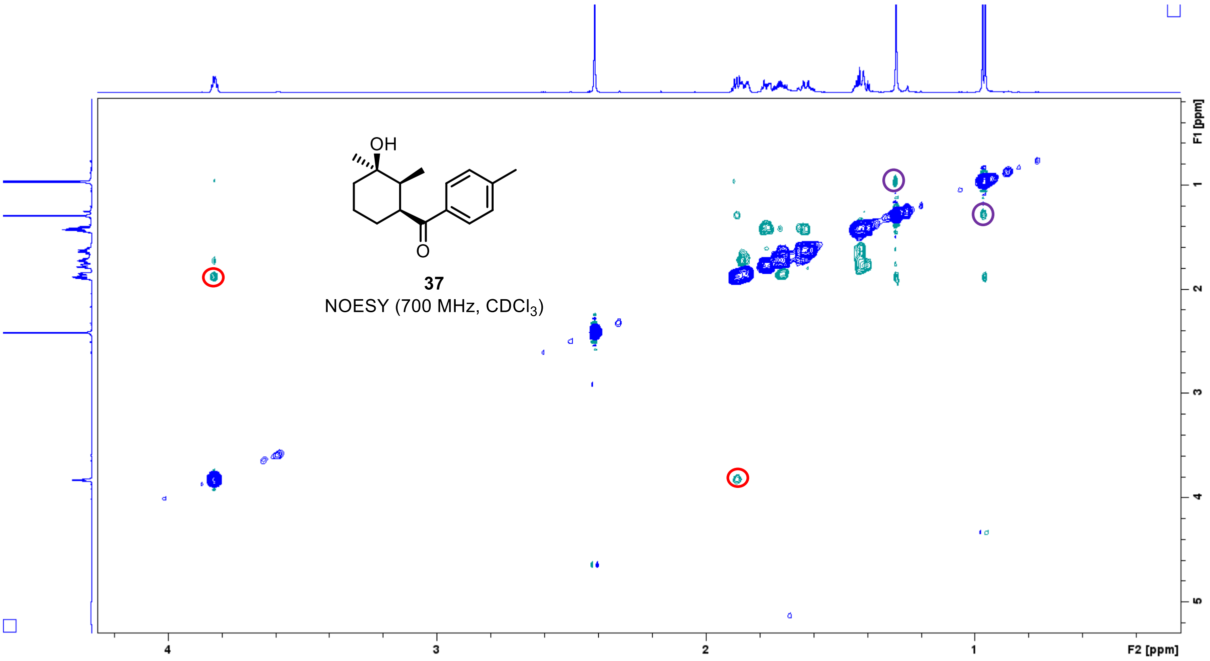

Highlighted in: *Red: opposite-phase crosspeaks (= positive NOE) between 3.83 ppm + 1.88 ppm. Purple: opposite-phase crosspeaks between 1.29 + 0.97 ppm.*

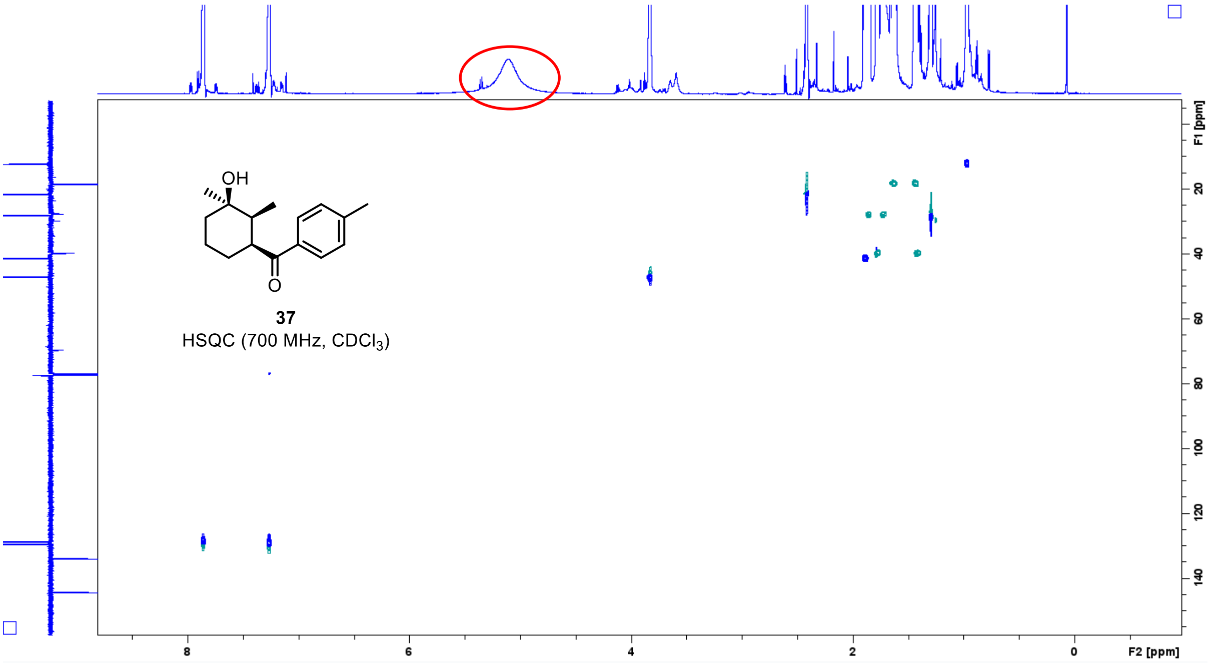

Proof that the signal at 5.10 ppm arises from an OH proton (no signal in HSQC). The unusual downfield shift of this proton is indicative of H-bonding (cf. all our acyl 9-decalinol products, where this was proven by SC-XRD).

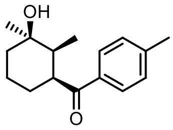

37
NOESY (700 MHz, $CDCl_3$)
Zoom-in on OH proton

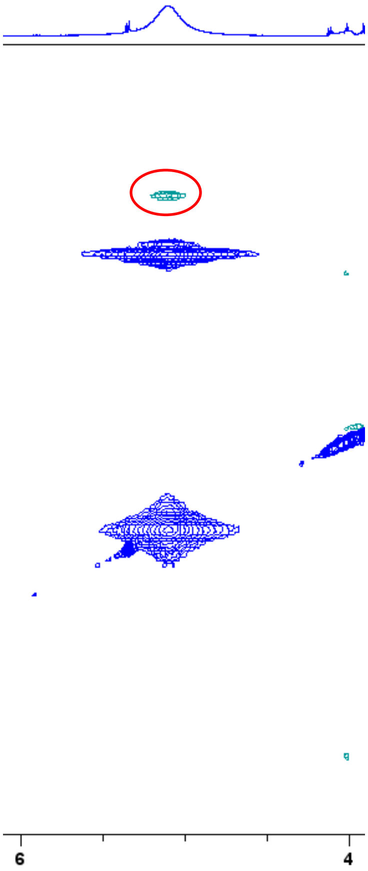

Highlighted is the opposite-phase crosspeak (= positive NOE) between 5.10 ppm + 0.97 ppm.

Evidence for **37 (2-epi)**:

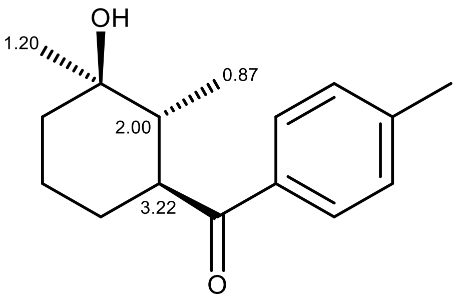

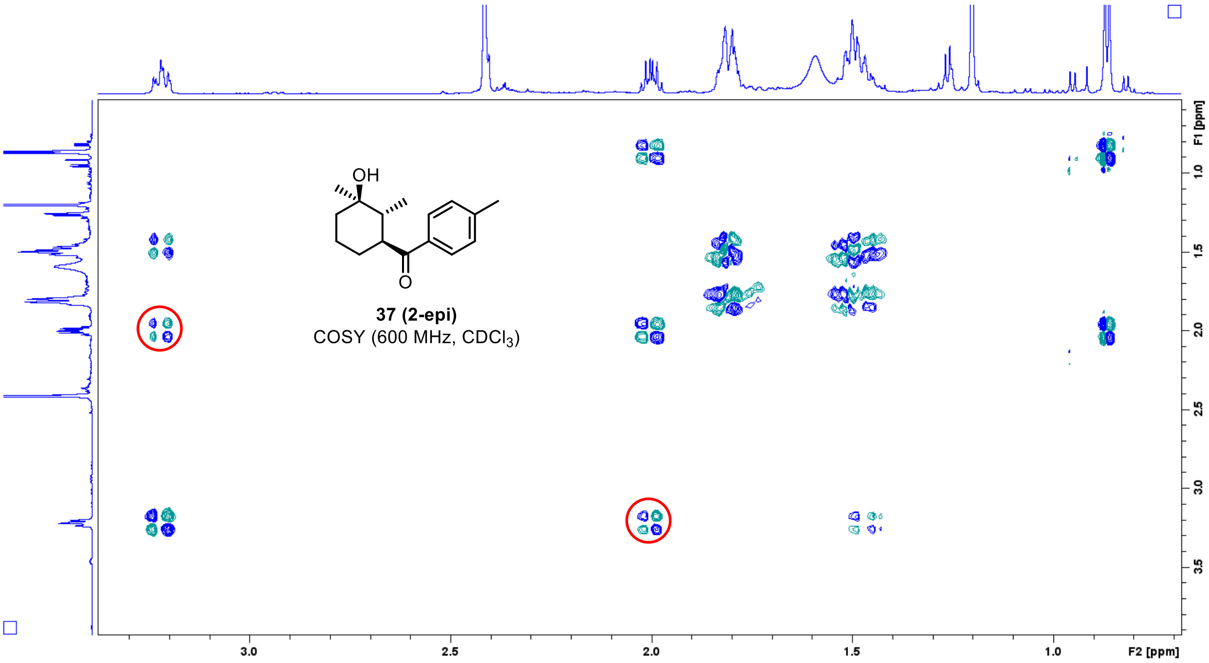

Highlighted are crosspeaks between 3.22 and 2.00 ppm.

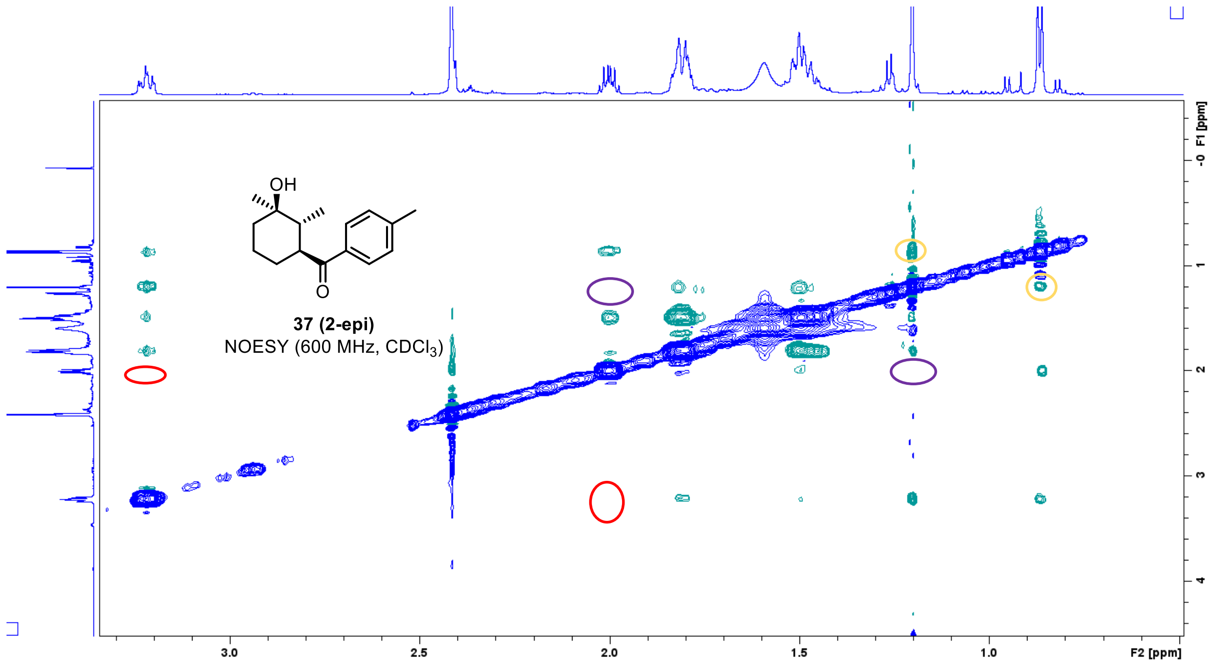

Highlighted in: *Red: missing opposite-phase crosspeaks (= positive NOE) between 3.22 + 2.00 ppm. Purple: missing opposite-phase crosspeaks between 2.00 + 1.20 ppm. Orange: opposite-phase crosspeaks between 1.20 + 0.87 ppm.*

(4-hydroxy-2,4-dimethylcyclohexyl)(p-tolyl)methanone (39)

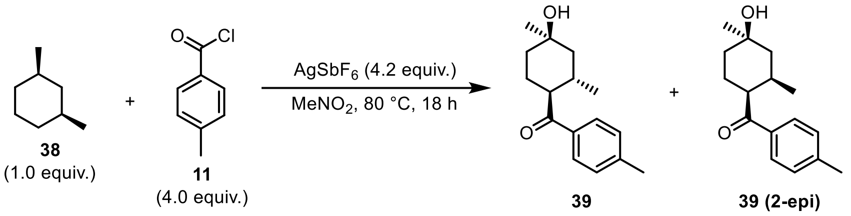

In a small flame-dried Schlenk flask equipped with a stirring bar and a septum, $AgSbF_6$ (433 mg, 1.26 mmol, 4.20 equiv., weighed in glovebox) was dissolved in 1.5 mL $MeNO_2$ at ambient temperature (23 °C). *p*-Toluoyl chloride (186 mg, 1.20 mmol, 4.00 equiv.) was added under stirring at ambient temperature, followed immediately by *cis*-1,3-dimethylcyclohexane (43 µL, 0.30 mmol, 1.00 equiv.). The resulting suspension was placed into a heating block (pre-heated to 80 °C) and stirred for 18 hours at 80 °C. After cooling down to ambient temperature (23 °C), the reaction was stopped by adding 10% aqueous K_2CO_3 solution (5 mL) and stirring for 1 h at ambient temperature (23 °C). The mixture was transferred to a separatory funnel, diluted with more 10% aqueous K_2CO_3 solution (5 mL) and the aqueous layer was extracted with DCM (3 × 20 mL). The combined organic layers were dried over anhydrous sodium sulfate, the dried solution was filtered, and the filtrate was concentrated under reduced pressure (final drying for >30 min at <1 mbar at 23 °C to remove unreacted decalin and $MeNO_2$). *dr* was determined by ^1H NMR analysis of the crude reaction product using mesitylene as internal standard (**39**:**39 (2-epi**) = 2.0:1). Purification by flash column chromatography (5 – 60% EtOAc in heptanes) afforded a mixture of **39** and **39 (2-epi)** (38 mg, 52%, **39:39 (2-epi)** = 1.8:1).

R_f = 0.39 (identical for **39** and **39 (2-epi)**) in 40% EtOAc/heptanes

^{a} denotes signals arising from **39**, ^{b} denotes signals arising from **39 (2-epi)**. Undesignated signals arise from both isomers.

^{1}H NMR (700 MHz, CDCl$_3$) δ 7.87 (d, *J* = 8.2 Hz, 1.28H^a), 7.81 (d, *J* = 8.2 Hz, 0.72H^b), 7.28 – 7.23 (m, 2H), 3.46 (dt, *J* = 7.7, 4.5 Hz, 0.36H^b), 2.96 – 2.91 (m, 0.65H^a), 2.41 (s, 1.98H^a), 2.40 (s, 1.11H^b), 2.33 – 2.27 (m, 0.67H^a), 2.19 – 2.14 (m, 0.38H^b), 2.11 – 2.05 (m, 0.40H^b), 1.91 (dd, *J* = 13.1, 6.7 Hz, 0.37H^b), 1.83 – 1.64 (m, 4H), 1.54 – 1.44 (m, 1.36H), 1.27 (s, 1.95H^a), 1.26 (s, 1.14H^b), 0.95 (d, *J* = 7.2 Hz, 1.07H^b), 0.82 (d, *J* = 6.5 Hz, 1.99H^a).

^{13}C NMR (176 MHz, CDCl$_3$) δ 203.9 (C^a), 202.8 (C^b), 143.9 (C^a), 143.5 (C^b), 135.1 (C^a), 134.8 (C^b), 129.5 (2CH^a), 129.4 (2CH^b), 128.5 (2CH^a), 128.4 (2CH^b), 70.7 (C^b), 69.5 (C^a), 51.5 (CH^a), 47.1 (CH$_2$^{a}), 46.3 (CH^b), 44.7 (CH$_2$^{b}), 38.2 (CH$_2$^{a}), 37.4 (CH$_2$^{b}), 32.0 (CH^b), 31.4 (CH^a), 29.8 (CH^a), 29.6 (CH^b), 26.5 (CH$_2$^{a}), 22.0 (CH$_2$^{b}), 21.74 (CH$_3$^{a}), 21.71 (CH$_3$^{b}), 20.5 (CH^a), 17.5 (CH$_3$^{b}).

IR (neat) ν_{max}: 3458, 2957, 2925, 2870, 1670, 1605, 1219, 1203, 1180, 933, 908, 737.

HRMS (ESI$^+$): exact mass calculated for $[M+Na]^+$ ($C_{16}H_{22}O_2Na$) requires *m/z* 269.1512, found *m/z* 269.1511.

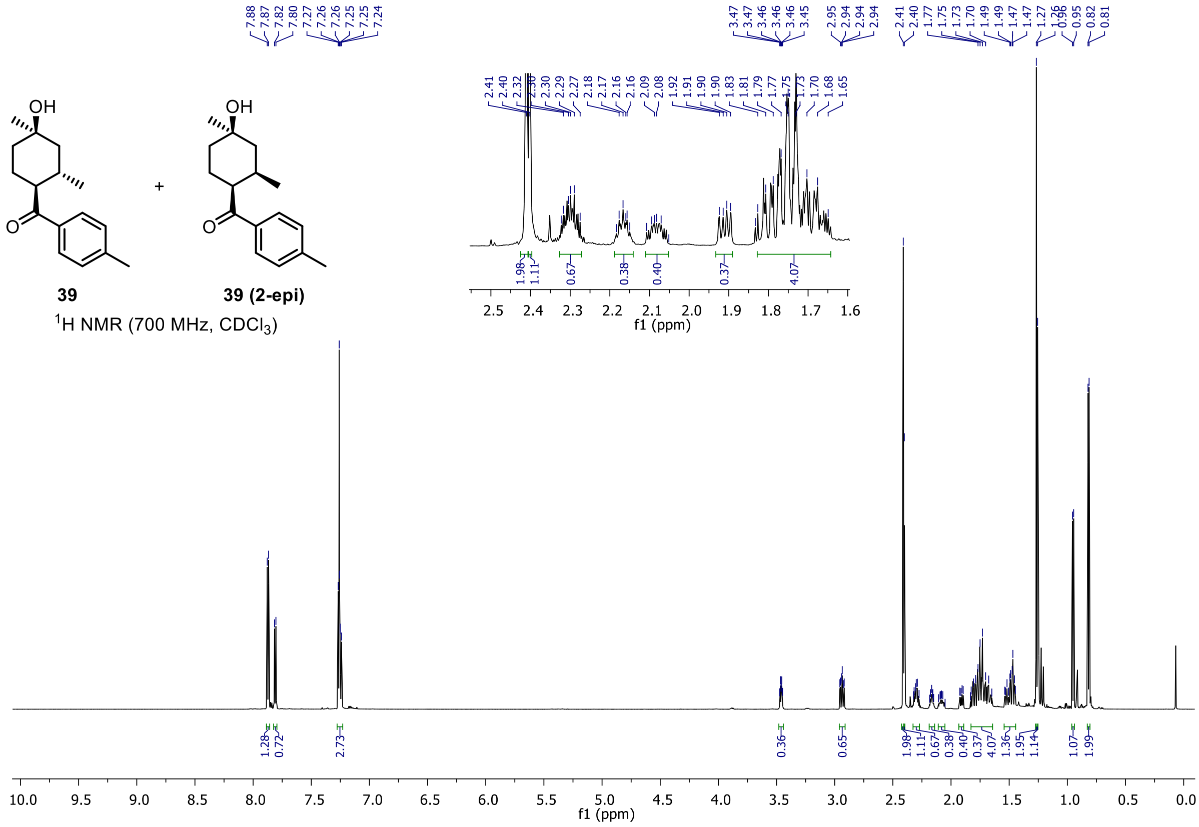

39 + **39 (2-epi)**

^1H NMR (700 MHz, $CDCl_3$)

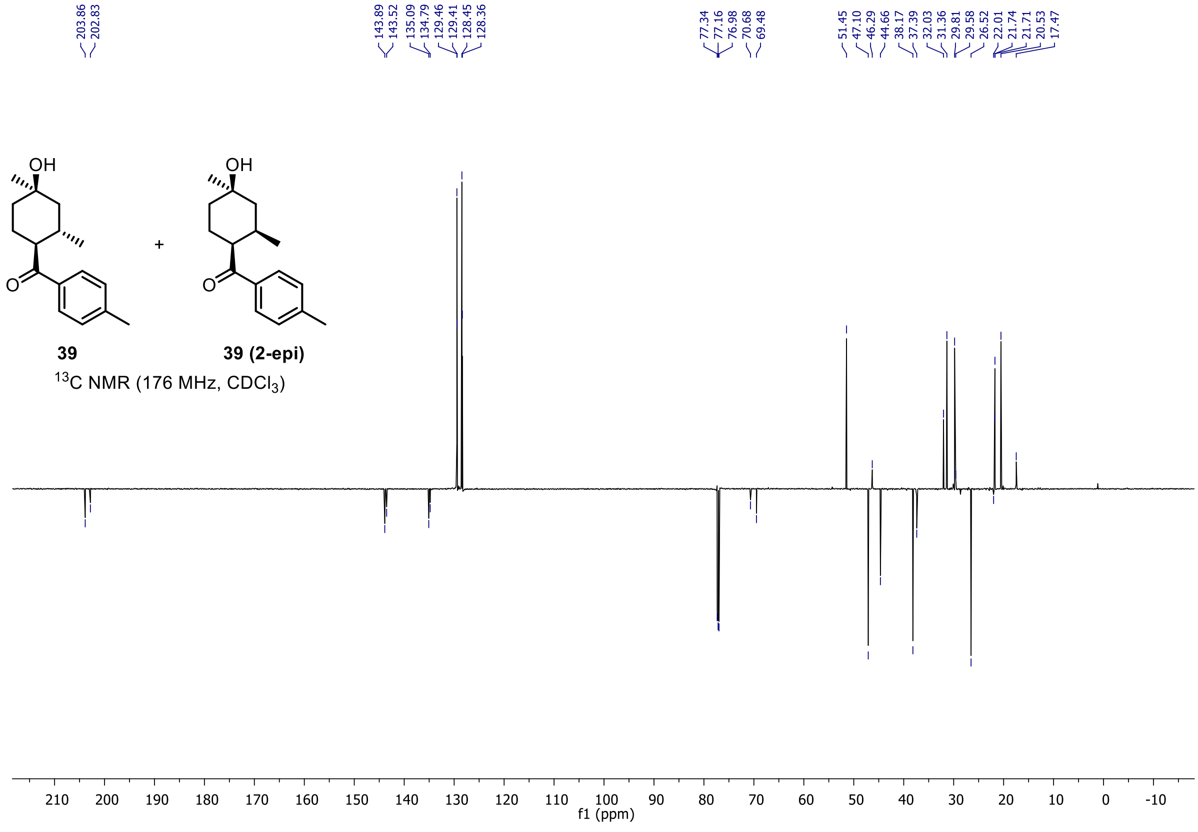

39 + **39 (2-epi)**

^{13}C NMR (176 MHz, $CDCl_3$)

Stereochemical assignment of **39** and **39 (2-epi)**:

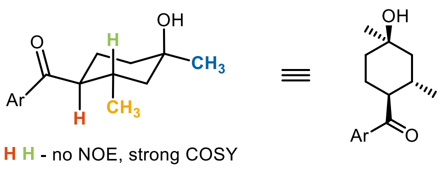

H H - no NOE, strong COSY
CH_3 H - NOE
CH_3 CH_3 - no NOE
H CH_3 - no NOE

39

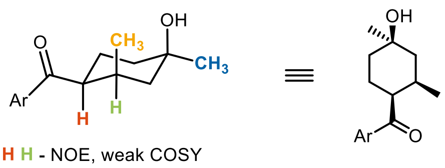

H H - NOE, weak COSY
CH_3 H - no NOE
CH_3 CH_3 - no NOE
H CH_3 - no NOE

39 (2-epi)

Evidence for **39** and **39 (2-epi)**:

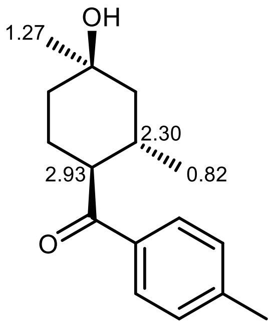

39

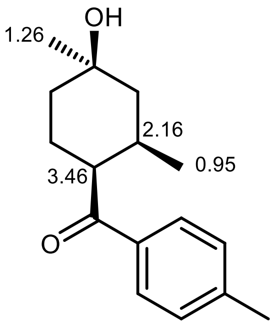

39 (2-epi)

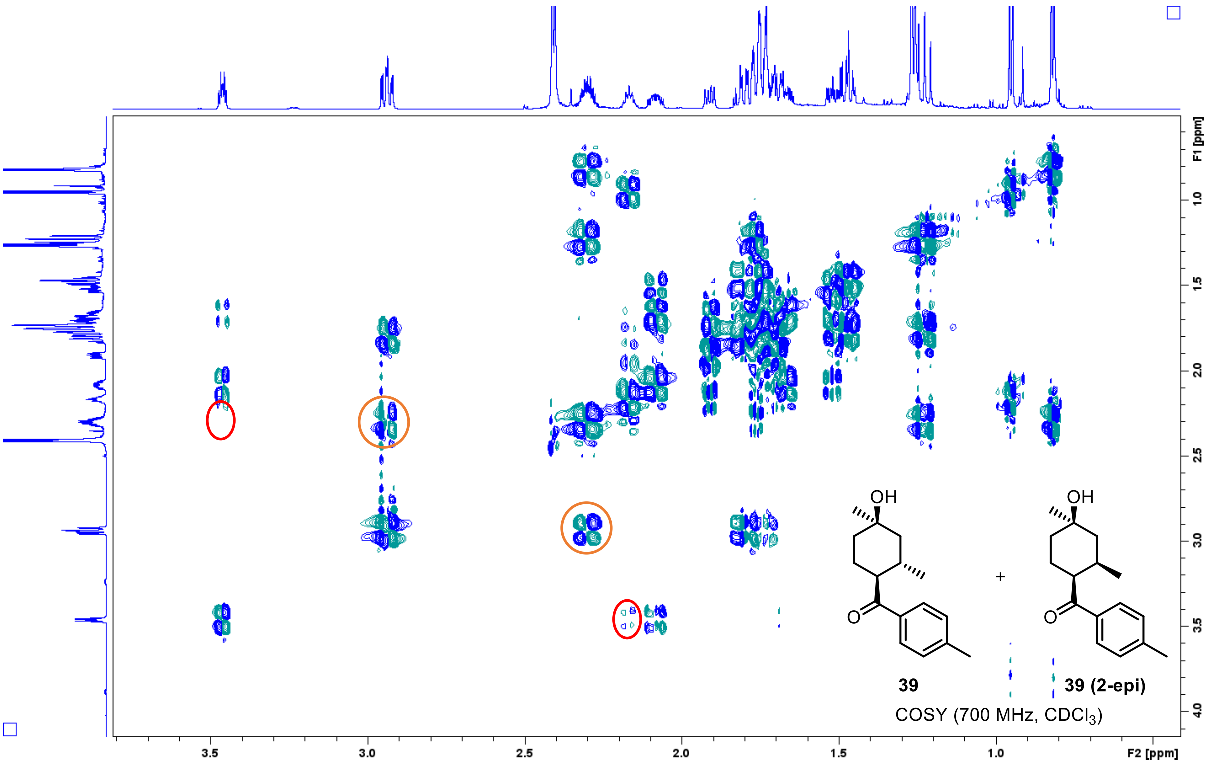

***Highlighted in:** Orange: strong COSY signals between 2.93 + 2.30 ppm. Red: weak COSY signals between 3.46 + 2.16 ppm.*

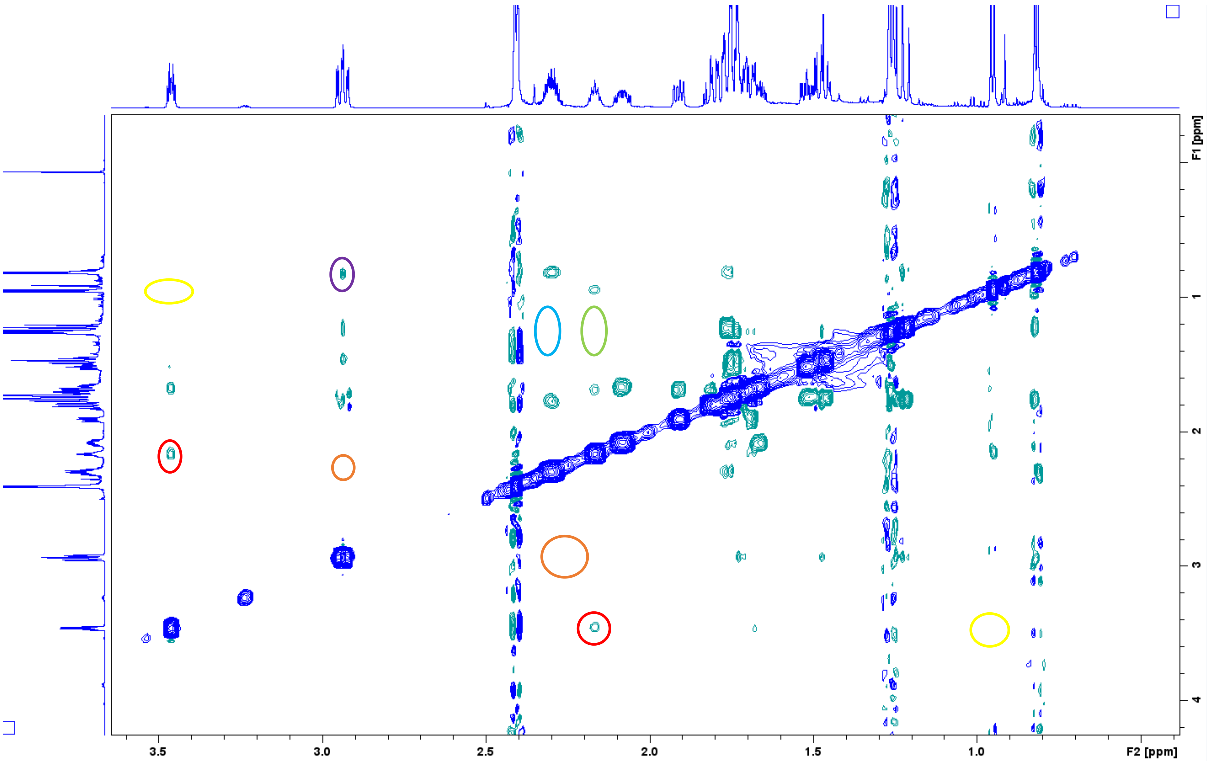

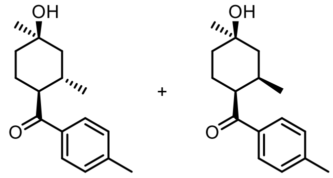

39 **39 (2-epi)**

NOESY (700 MHz, $CDCl_3$)

Highlighted in*: Orange: missing opposite-phase crosspeaks (= positive NOE) between 2.93 + 2.30 ppm. Red: opposite-phase crosspeaks between 3.46 + 2.16 ppm. Purple: opposite-phase crosspeak between 2.93 + 0.82 ppm. Blue: missing opposite-phase crosspeak between 2.30 + 1.27 ppm. Green: missing opposite-phase crosspeak between 2.16 + 1.26 ppm. Yellow: missing opposite-phase crosspeaks between 3.46 + 0.95 ppm.*

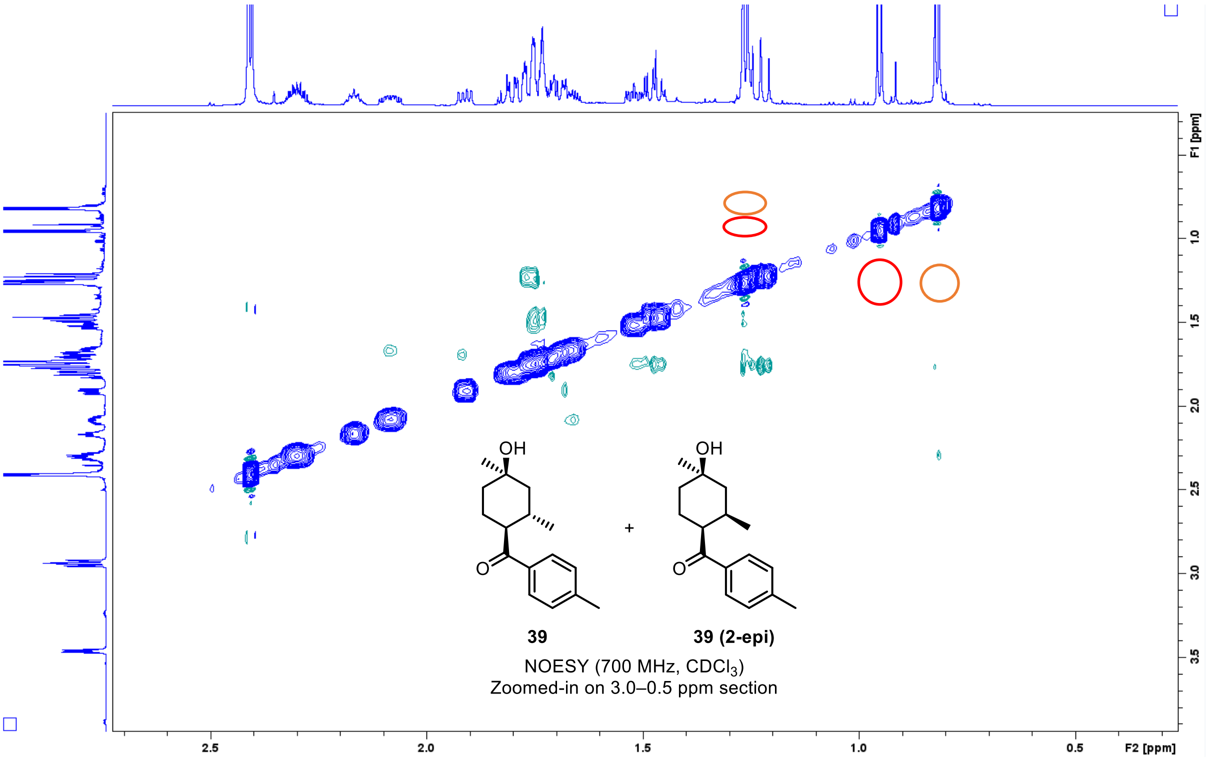

Highlighted in: *Orange: missing opposite-phase crosspeaks (= positive NOE) between 1.27 + 0.82 ppm. Red: missing opposite-phase crosspeaks between 1.26 + 0.95 ppm.*

4.7 Full scope of alkane substrates

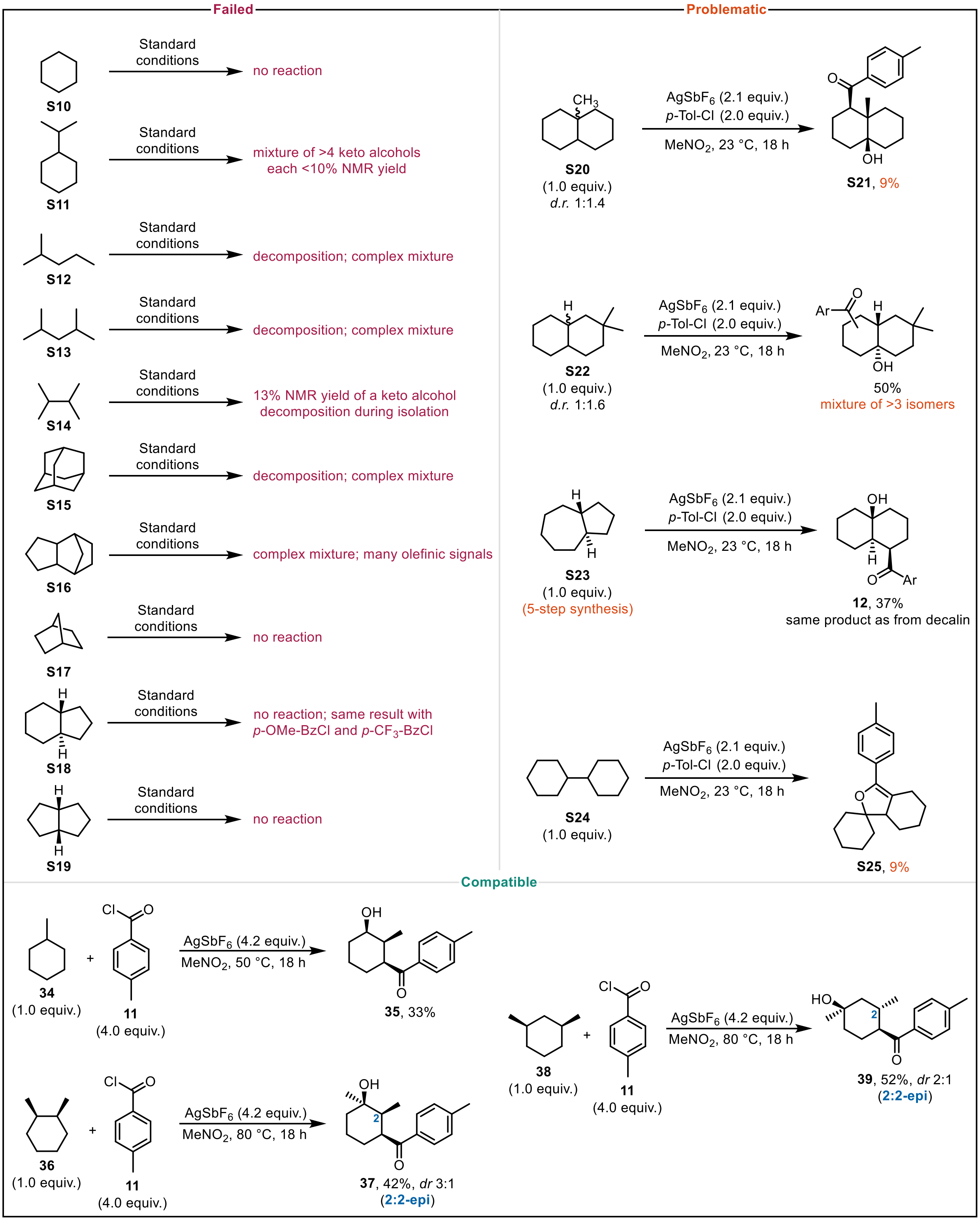

Scheme S8: Full scope of alkane substrates which were tested in our reaction. Standard conditions = alkane (0.1 mmol), *para*-toluoyl chloride (2.0 equiv.), $AgSbF_6$ (2.1 equiv.), $MeNO_2$ (0.5 mL), 23 °C, 18 h (the reactions were conducted as described in **General Procedure A**, see section **4.4**). *p*-Tol-Cl = *para*-toluoyl chloride. For synthesis of starting materials that were not commercially available, see section **4.8**.

Alkanes that do not contain a tertiary C–H bond (such as **S10**) were unreactive, presumably due to the lack of a sufficiently hydridic C–H bond. **S11** afforded a complex mixture of products (regioisomers and diastereomers) that could not be separated by standard purification techniques (column chromatography, preparative TLC). Two open-chain alkanes **S12** and **S13** afforded complex product mixtures, that (based on analysis of the crude reaction products by ^{1}H and ^{13}C NMR) did not contain keto alcohols. Alkane **S14** afforded 13% (NMR yield, determined by ^{1}H NMR analysis of crude reaction product using mesitylene as internal standard) of what seemed to be a keto alcohol, however, this compound partially decomposed during isolation on SiO_2 and then fully decomposed after standing in $CDCl_3$ (before ^{13}C NMR could be measured, ~6 h). Increasing the reaction temperature to 50 °C or introducing an additional 1 M NaOH washing step during extraction did not lead to any significant improvement. In the case of **S15**, the aldehyde and Henry product (see Scheme **4c**) were observed in the crude reaction product in minor quantities, indicating that the hydride abstraction was at least partially successful. However, no ketone signals were detected, hence we believe that the adamantyl cation cannot react in a productive pathway with acylium ions. **S16** afforded a highly complex mixture of products, several of which contained olefinic protons (observed in ^{1}H NMR) and some contained a ketone (observed in ^{13}C NMR, minor quantities). We believe that the hydride abstraction in this case proceeds to some extent, however, the formed olefin is not sufficiently reactive towards acylium ions. The presence of ketone signals and absence of alcohols could be explained if the oxocarbenium ion - rather than being hydrolyzed to a keto alcohol - eliminates a proton, generating an acyl-substituted olefin. An attempt to separate the components of this mixture by column chromatography failed, as the impurities co-eluted and some components further decomposed on SiO_2. No products were formed in the cases of **S17**, **S18**, and **S19**, indicating that the hydride abstraction step did not ensue. No improvement was observed for **S18** when the acylium ion source was changed from *p*-toluoyl chloride to more electron-rich or electron-poor acyl chlorides.

In the case of substituted decalins, the synthetic utility of this reaction was severely impacted by a lack of regioselectivity or low reactivity. 9-Methyldecalin (**S20**) afforded the *cis*-decalin product **S21**, but unfortunately in a low yield of 9%. 2,2-Dimethyldecalin (**S22**) afforded a mixture of three major isomers (and multiple unidentified impurities), which could not be separated by standard purification techniques (column chromatography or preparative TLC). Interestingly, the Decahydroazulene (**S23**) afforded an identical product as decalin, a phenomenon that has already been described in literature (skeletal rearrangement of the intermediary formed carbocation).[36] **S24** afforded the dihydrofuran product **S25**, albeit in a low yield – the mechanism of formation of this product was already described in literature.[36]

Methylcyclohexane (**34**) and dimethylcyclohexanes **36** and **38** afforded the products in good yields, however, a short re-optimization was required (Table **S2**). An increase in temperature (50 °C for **34** and 80 °C for **36** and **38**) led to higher reaction yields and better purity of the crude reaction products. The stoichiometry used for aliphatic acyl chlorides (see Section **4.4**) was found to give highest product yields. The *cis* isomers of **36** and **38** (as shown in Scheme **S8**) were slightly more reactive and provided better product yields.

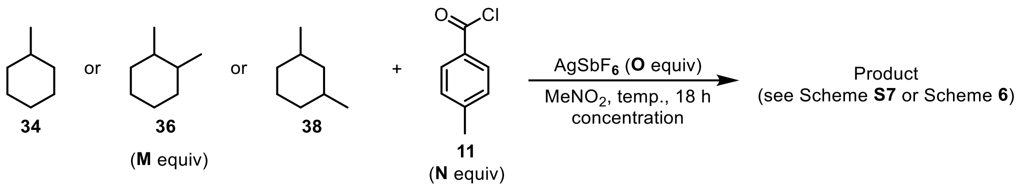

Entry	**Alkane**	**M**	**N**	**O**	temp. (°C)	concentration	Yield, *dr*	Notes
1	36[a]	1.0	2.0	2.1	23	0.2 M	33%, 1:1	
2	36[a]	1.0	2.0	2.1	**50**	0.2 M	34%, 1:1.1	
3	36[a]	1.0	2.0	2.1	**80**	0.2 M	34%, 1:1.8	Highest purity
4	36[a]	1.0	2.0	2.1	**90**	0.2 M	34%, 1:1.8	
5	36[a]	1.0	2.0	2.1	**–20 to 23**	0.2 M	35%, 1:1	
6	36 **(*cis*)**	1.0	**2.0**	**2.1**	23	0.2 M	38%, 1:1	
7	36 **(*trans*)**	1.0	**2.0**	**2.1**	23	0.2 M	23%, 1:1	
8	36[a]	1.0	2.0	2.1	23	**0.1 M**	21%, 1:1	
9	36[a]	**3.0**	**2.0**	**2.1**	23	0.2 M	40%, 1:1	
10	36[a]	**1.0**	**4.0**	**4.2**	23	0.2 M	51%, 1:1.5	Lower purity
12	36 (*cis*)	1.0	4.0	4.2	80	0.2 M	45%, 1:2.8 (42%†)	0.3 mmol scale
13	38[b]	1.0	2.0	2.1	23	0.2 M	30%, 1:1.3	
14	38[b]	1.0	2.0	2.1	**50**	0.2 M	32%, 1:1.5	
15	38[b]	1.0	2.0	2.1	**80**	0.2 M	34%, 1:1.8	Highest purity
16	38[b]	1.0	2.0	2.1	**–20 to 23**	0.2 M	32%, 1:1.2	
17	38 **(*cis*)**	1.0	2.0	2.1	23	0.2 M	35%, 1:1.3	
18	38[b]	**3.0**	**2.0**	**2.1**	23	0.2 M	39%, 1:2.3	
19	38[b]	**1.0**	**4.0**	**4.2**	23	0.2 M	64%, 1:2.4	Lower purity
20	38 (*cis*)	1.0	4.0	4.2	80	0.2 M	54%, 1:1.8 (52%†)	0.3 mmol scale
21	34	1.0	2.0	2.1	23	0.2 M	19%	
22	34	1.0	2.0	2.1	**50**	0.2 M	21%	Highest purity
23	34	1.0	2.0	2.1	**80**	0.2 M	7%	
24	34	1.0	4.0	4.2	50	0.2 M	33% (33%†)	0.3 mmol scale

Table S2. Reaction optimization for alkane substrates. All reactions were done on 0.1 mmol scale, unless otherwise specified. Concentrations are given with respect to the theoretical product yield (0.1 mmol, for entries highlighted in green 0.3 mmol). Conditions: alkane (**M** equiv.), *p*-toluoyl chloride (**N** equiv.), $AgSbF_6$ (**O** equiv.), $MeNO_2$ (0.5–1.5 mL, anhydrous, see Section **4.2** for more details). The reactions were conducted as described in **General Procedure A**, see section **4.4**. Yields and *dr were* determined by ^1H NMR analysis of the crude reaction product using mesitylene as internal standard. [a]Technical mixture of isomers (*cis*:*trans* = 6:1). [b]Technical mixture of isomers (*cis*:*trans* = 2.0:1). †Isolated yield.

(4-hydroxy-8-methyldecahydronaphthalen-1-yl)(p-tolyl)methanone--4-methyldecahydronaphthalene (S21)

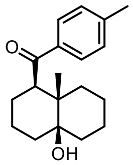

S21

The title compound was prepared following **General Procedure A** using *p*-toluoyl chloride (26 µL, 0.20 mmol, 2.00 equiv.), 9-methyldecalin (**S20**, 15.2 mg, 0.10 mmol, 1.00 equiv., *dr* ~ 1:1.4) and $AgSbF_6$ (72 mg, 0.21 mmol, 2.10 equiv.). Purification by flash column chromatography (0 – 50% Et_2O in pentane) gave **S21** as a colorless oil (2.6 mg, 9%).

R_f = 0.16 in 20% Et_2O/heptanes

The compound undergoes conformational changes (ring-flipping) on NMR time scale, therefore measurement at –35 °C was necessary to reveal all signals and prevent broadening effects.

^{1}H NMR (700 MHz, $CDCl_3$, 238 K) δ 7.88 (d, *J* = 8.2 Hz, 2H), 7.28 (d, *J* = 8.0 Hz, 2H), 7.05 (s, 1H), 3.77 (d, *J* = 6.9 Hz, 1H), 2.43 – 2.38 (m, 4H), 2.10 – 2.05 (m, 1H), 1.92 – 1.87 (m, 1H), 1.71 – 1.46 (m, 11H), 1.13 (s, 3H), 1.09 (dd, *J* = 13.8, 6.7 Hz, 1H).

^{13}C NMR (176 MHz, $CDCl_3$, 238 K) δ 206.3 (C), 144.9 (C), 133.6 (C), 129.6 (2CH), 129.0 (2CH), 71.6 (C), 52.6 (CH), 51.0 (C), 38.0 (CH_2), 37.8 (CH_2), 33.5 (CH_2), 26.9 (CH_2), 26.6 (CH_2), 26.3 (CH_2^l), 25.8 (CH_3), 21.9 (CH_3), 17.0 (CH_2).

IR (neat) v_{max}: 3360, 2929, 2865, 1659, 1605, 1450, 1224, 1181, 750.

HRMS (ESI$^+$): exact mass calculated for $[M+Na]^+$ ($C_{19}H_{26}O_2Na$) requires *m*/*z* 309.1825, found *m*/*z* 309.1827.

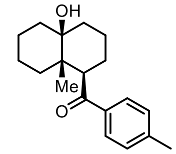

S21
^{1}H NMR (700 MHz, $CDCl_3$, **238 K**)

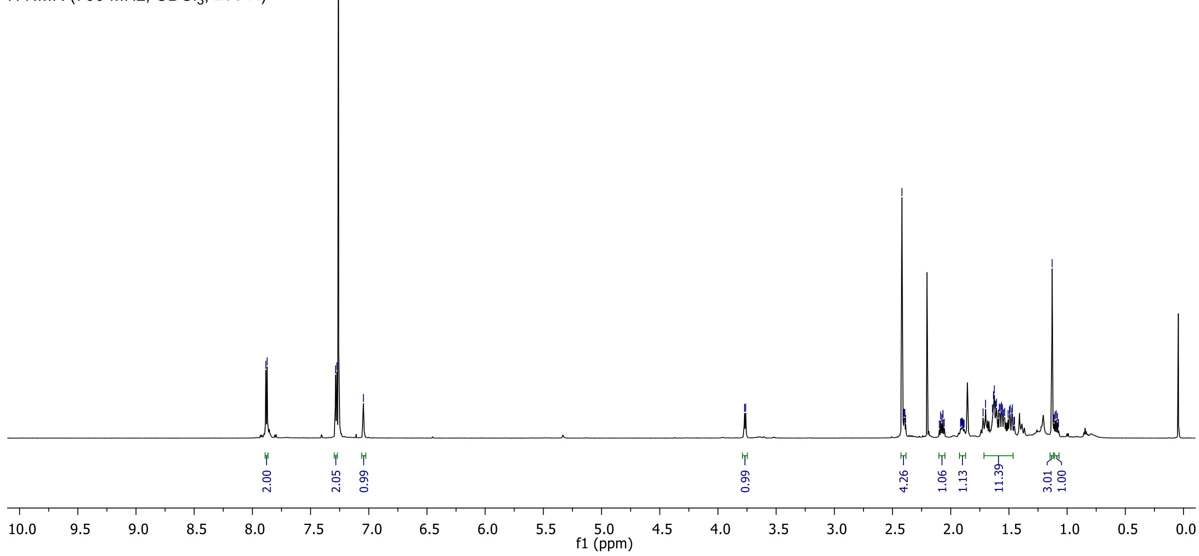

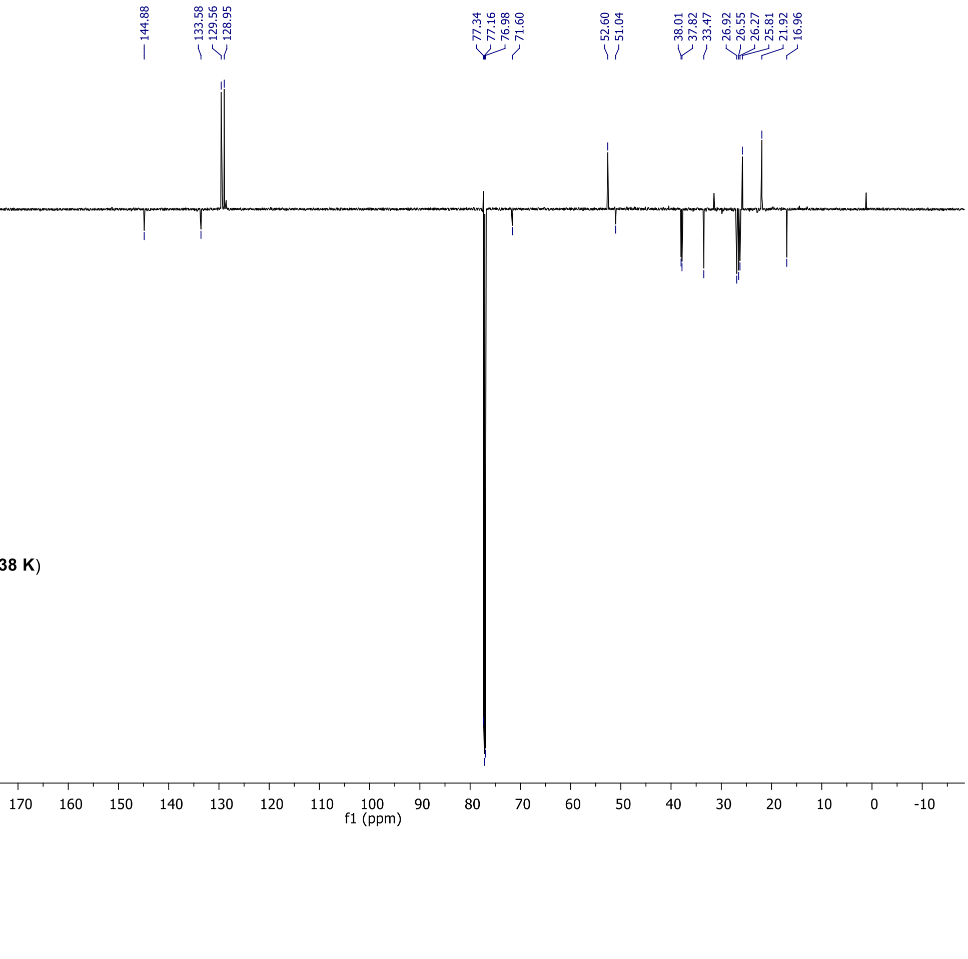

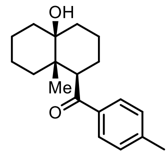

S21
^{13}C NMR (176 MHz, $CDCl_3$, **238 K**)

decahydroazulene (S23) into 12

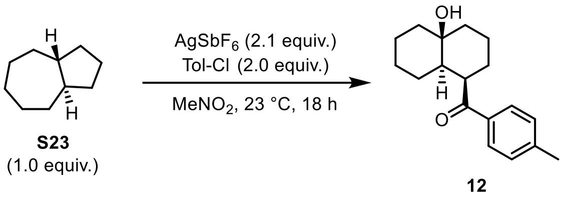

The reaction was carried out following **General Procedure A** using *p*-toluoyl chloride (26 µL, 0.20 mmol, 2.00 equiv.), *trans*-decahydroazulene (**S23**, 13.8 mg, 0.10 mmol, 1.00 equiv.) and $AgSbF_6$ (72 mg, 0.21 mmol, 2.10 equiv.). Purification by flash column chromatography (0 – 40% Et_2O in pentane) gave **12** as a yellow crystalline solid (10.1 mg, 37%).

All analytical data were identical to those reported in Section **4.4** (compound **12**).

3'-(p-tolyl)-5',6',7',7a'-tetrahydro-4'H-spiro[cyclohexane-1,1'-isobenzofuran] (S25)

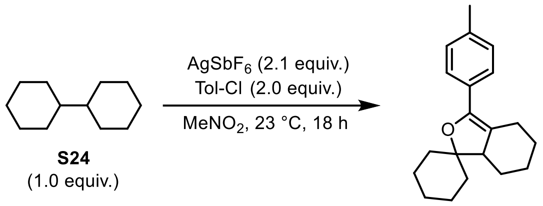

S24
(1.0 equiv.)

S25

The title compound was prepared following **General Procedure A** using *p*-toluoyl chloride (132 µL, 1.00 mmol, 2.00 eq.), bicyclohexyl (**S24**, 97 µL, 0.50 mmol, 1.00 eq.) and $AgSbF_6$ (361 mg, 1.05 mmol, 2.10 eq). Purification by flash column chromatography (0 – 20% Et_2O in pentane) gave **S25** (12.9 mg, 9%) as a colorless oil.

$\mathbf{R_f}$ = 0.63 in 20% Et_2O/heptanes

^{1}H NMR (400 MHz, $CDCl_3$) δ 7.41 (d, *J* = 8.1 Hz, 2H), 7.14 (d, *J* = 8.0 Hz, 2H), 2.82 – 2.74 (m, 1H), 2.53 – 2.45 (m, 1H), 2.34 (s, 3H), 2.18 – 2.06 (m, 1H), 1.89 – 1.71 (m, 7H), 1.68 – 1.42 (m, 4H), 1.40 – 1.16 (m, 5H).

^{13}C NMR (101 MHz, $CDCl_3$) δ 143.7 (C), 137.2 (C), 130.2 (C), 128.9 (2CH), 127.2 (2CH), 110.9 (C), 84.2 (C), 55.2 (CH), 38.4 (CH_2), 31.9 (CH_2), 27.9, 26.8 (CH_2^{l}), 26.0 (CH_2), 25.8 (CH_2), 25.2 (CH_2), 23.1 (CH_2), 22.9 (CH_2), 21.4 (CH_3).

IR (neat) ν_{max}: 2925, 2855, 1706, 1675, 1290, 1272, 1177, 1108, 754.

HRMS (EI$^+$): exact mass calculated for $[M]^{\cdot+}$ $(C_{20}H_{26}O)^{\cdot+}$ requires *m*/*z* 282.1978, found *m*/*z* 282.1980.

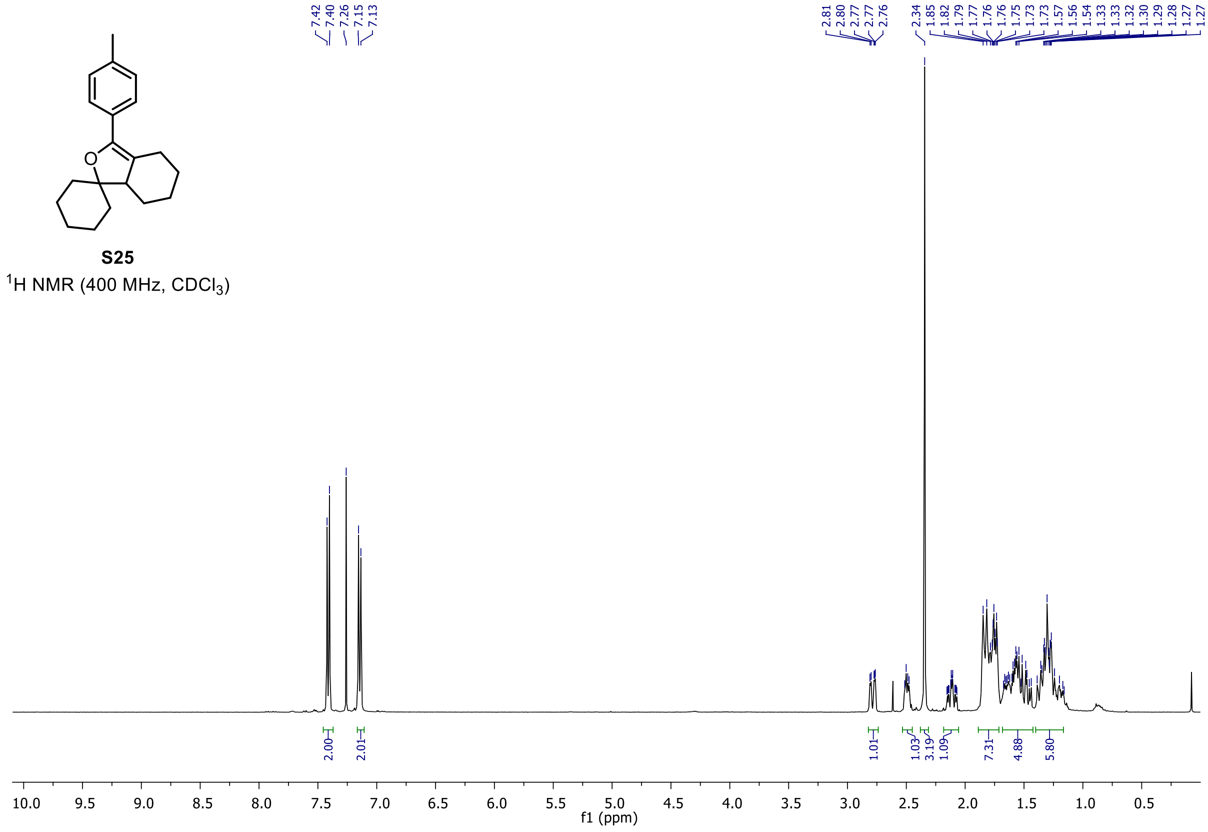

O
S25
^{1}H NMR (400 MHz, CDCl$_3$)
7.42
7.40
7.26
7.15
7.13
2.81
2.80
2.77
2.77
2.76
2.34
1.85
1.82
1.79
1.77
1.76
1.76
1.75
1.73
1.73
1.73
1.57
1.56
1.54
1.33
1.33
1.32
1.30
1.29
1.28
1.27
1.27
2.00
2.01
1.01
1.03
3.19
1.09
7.31
4.88
5.80
f1 (ppm)

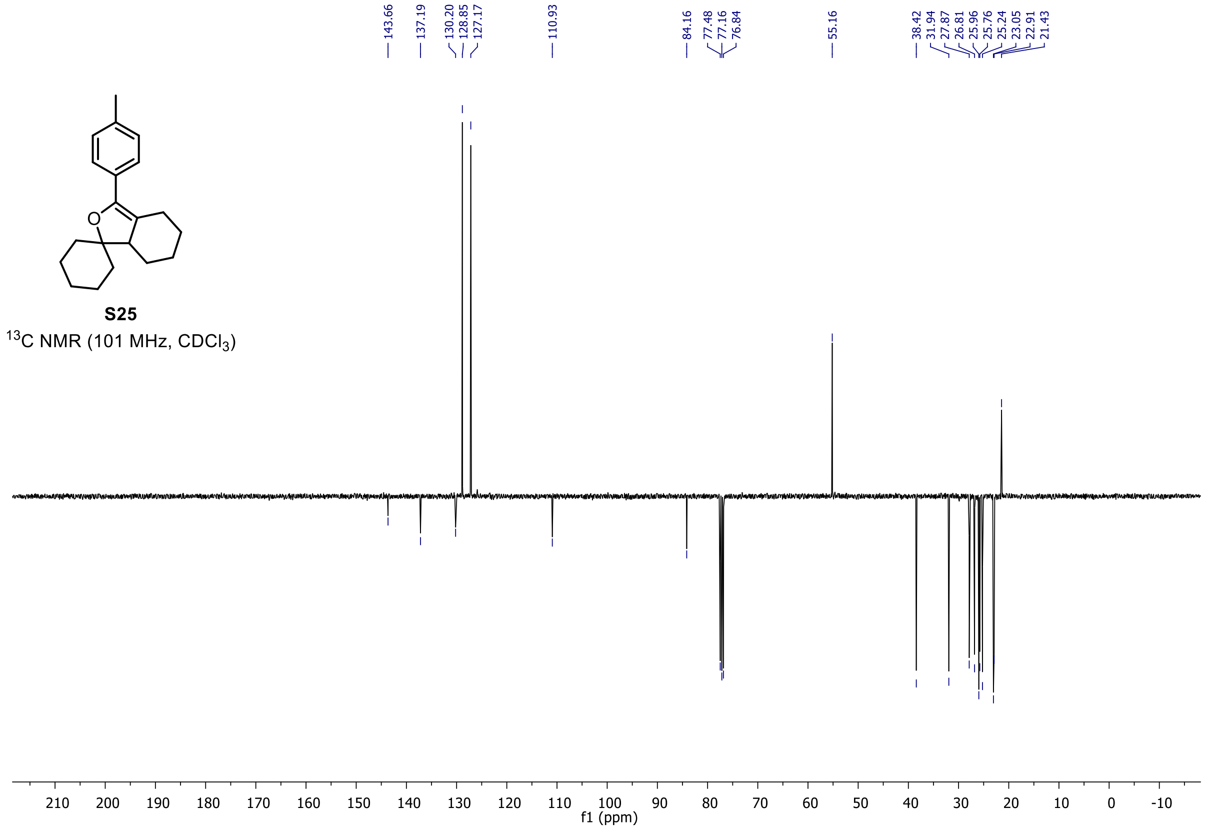

O
S25
^{13}C NMR (101 MHz, CDCl$_3$)
143.66
137.19
130.20
128.85
127.17
110.93
84.16
77.48
77.16
76.84
55.16
38.42
31.94
27.87
26.81
25.96
25.76
25.24
23.05
22.91
21.43
f1 (ppm)

4.8 Synthesis of miscellaneous alkane substrates

***cis*-bicyclo(3.3.0)octan-1-one (S26)**

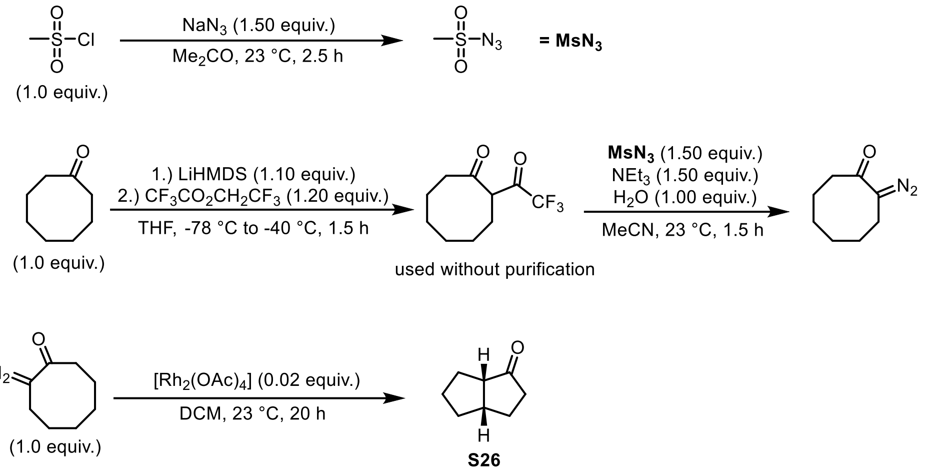

The title compound was prepared following the procedure of Müller *et al.*[44]

Step 1: Mesyl chloride (2.32 mL, 30.0 mmol) –> mesyl azide (2.78 g, 77%).

Step 2: Cyclooctanone (1.26 g, 10.0 mmol) –> α-diazocyclooctanone (1.08 g, 71%).

Step 3: α-diazocyclooctanone (609 mg, 4.00 mmol) –> **S26** (432 mg, 87%).

All analytical data were in good accordance with those reported in literature.[44]

^{1}H NMR (700 MHz, $CDCl_3$) δ 2.79 – 2.73 (m, 1H), 2.54 (td, *J* = 9.5, 4.2 Hz, 1H), 2.30 – 2.21 (m, 2H), 2.14 – 2.07 (m, 1H), 1.88 – 1.78 (m, 3H), 1.63 – 1.49 (m, 3H), 1.41 (td, *J* = 12.5, 6.1 Hz, 1H).

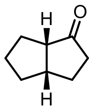

S26

^{1}H NMR (700 MHz, $CDCl_3$)

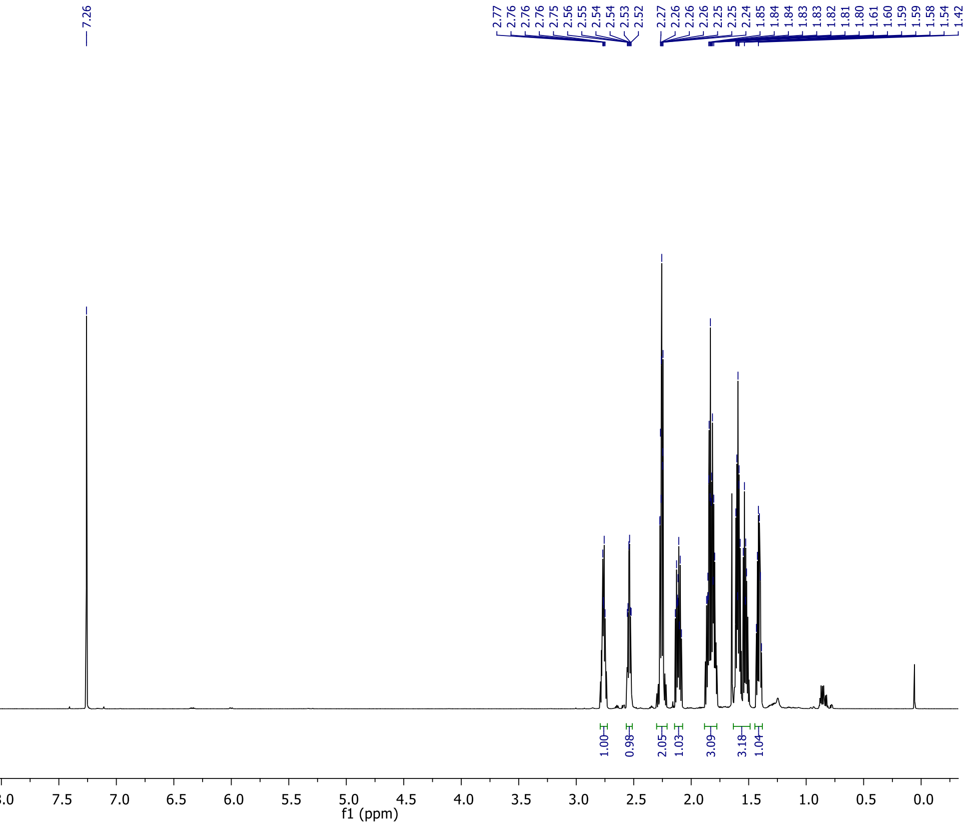

***cis*-bicyclo(3.3.0)octan-1-one (S19)**

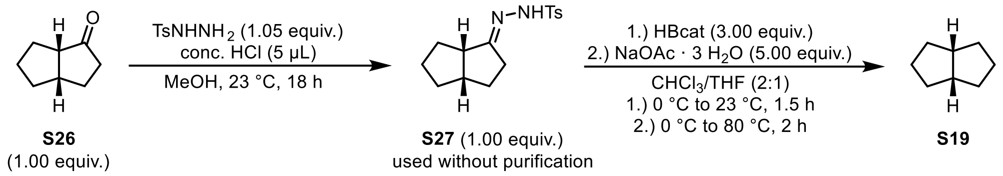

Ketone **S26** (420 mg, 3.38 mmol, 1.00 equiv.) was dissolved in MeOH (2.5 mL) under air at ambient temperature (23 °C). Tosylhydrazine (661 mg, 3.55 mmol, 1.05 equiv.) was added in one portion, followed by 5 µL of concentrated HCl (37% in H_2O). The mixture was stirred at ambient temperature (23 °C) for 18 hours, resulting in the formation of a voluminous colorless precipitate. The suspension was filtered, the solids were washed with pentane (3 × 3 mL) and dried in high vacuum, affording crude hydrazone **S27** (589 mg, ~2.02 mmol), which was used without further purification.

In a flame-dried 50 mL two-neck flask equipped with a septum and a reflux condenser attached to argon line, crude hydrazone **S27** (570 mg, ~1.95 mmol, 1.00 equiv.) was dissolved in a mixture of $CHCl_3$ (12 mL) and THF (6 mL) at ambient temperature (23 °C). The solution was cooled to 0 °C, after which catecholborane (0.62 mL, 5.85 mmol, 3.00 equiv.) was slowly added under stirring. The resulting mixture was stirred at 0 °C for 30 minutes, then warmed to ambient temperature (ice bath replaced for water bath) and stirred for 1 h. Subsequently, the mixture was cooled to 0 °C, and NaOAc·$3H_2O$ (1.33 g, 9.75 mmol, 5.00 equiv.) was added in three portions as a solid. After the addition was complete, the suspension was heated to reflux (90 °C oil bath) for 2 hours. The mixture was diluted with DCM (30 mL), solids were removed by filtration through a short pad of celite and the filtrate was poured on an aqueous saturated NH_4Cl solution (20 mL). The biphasic mixture was separated and the aqueous phase was extracted with DCM (3 × 10 mL). The combined organic phases were washed with brine (30 mL) and dried over anhydrous sodium sulfate, the dried solution was filtered, and the filtrate was concentrated under reduced pressure (min. 200 mbar, 50 °C water bath). The crude residue was purified by flash column chromatography (pentane) to afford **S19** as a colorless oil (64 mg, 30% over 2 steps).

The product is volatile and evaporates already at room temperature under atmospheric pressure, therefore caution is to be taken during solvent evaporation.

All analytical data were in good accordance with data reported in the literature.[45]

R_f = 0.93 in pentane

^{1}H NMR (600 MHz, $CDCl_3$) δ 2.43 – 2.32 (m, 2H), 1.71 – 1.66 (m, 4H), 1.55 – 1.51 (m, 2H), 1.41 – 1.37 (m, 2H), 1.22 – 1.17 (m, 4H).

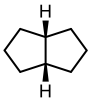

S19

^{1}H NMR (600 MHz, $CDCl_3$)

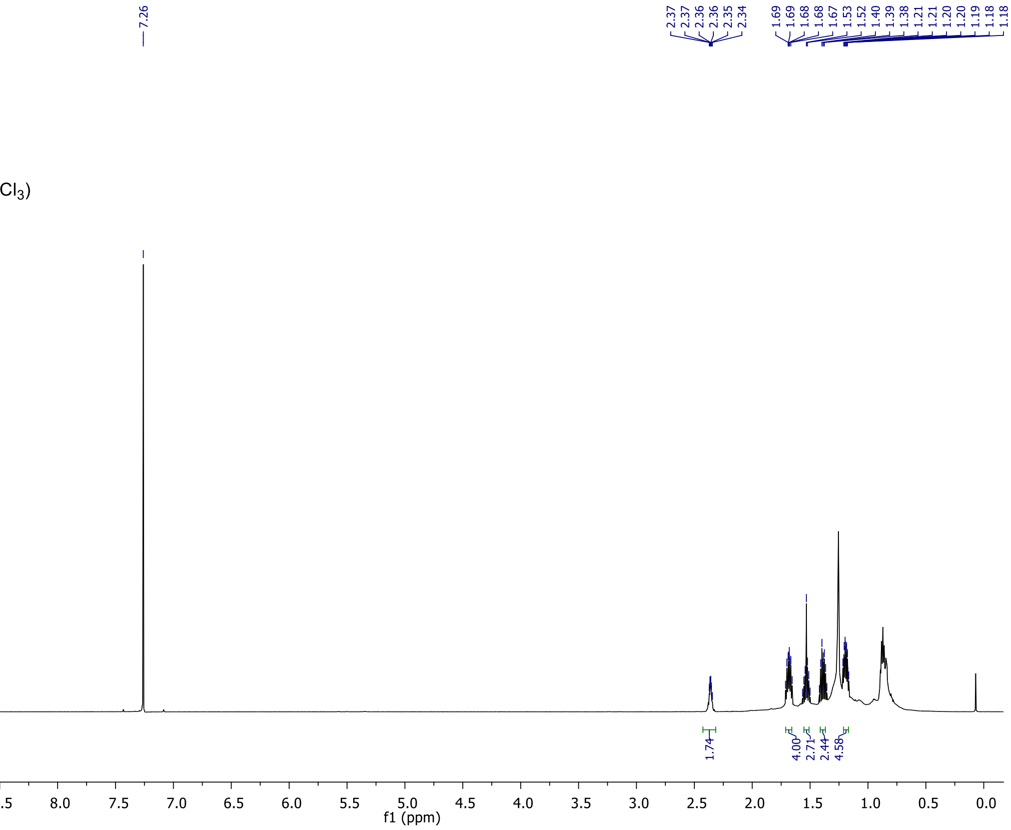

The product contains grease and pentane as minor impurities.[37]

cyclodeca-1,6-dione and *cis*-9-decalinol (S28 and S29)

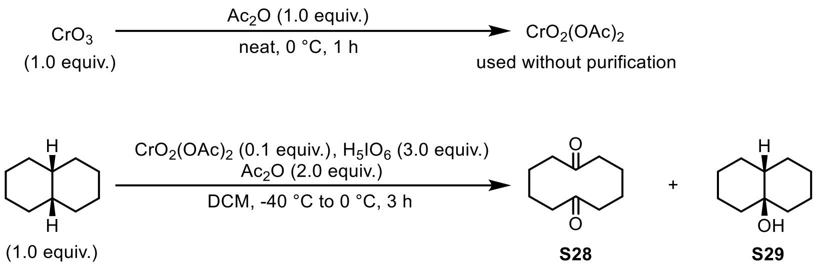

S28 and **S29** were prepared according to the literature procedure by S. Lee and P. L. Fuchs.[46]

cis-decalin (5.00 mL, 32.40 mmol) –> **S28** (1.61 g, 30%) and **S29** (0.35 g, 7%).

All analytical data were in good accordance with data reported in the literature.[47.48]

Data for **S28**:

^{1}H NMR (400 MHz, $CDCl_3$) δ 2.35 (app d, *J* = 3.6 Hz, 8H), 1.84 (app d, *J* = 2.6 Hz, 8H).

^{13}C NMR (101 MHz, $CDCl_3$) δ 214.2 (2C), 42.3 (4CH_2), 23.6 (4CH_2).

Data for **S29**:

^{1}H NMR (400 MHz, $CDCl_3$) δ 1.86 – 1.18 (m, 17H).

^{13}C NMR (101 MHz, $CDCl_3$) δ 71.9 (C), 43.0 (CH).

Due to conformational changes of the *cis*-decalin system, only the ring-junction ^{13}C resonances could be observed.

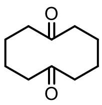

S28

^1H NMR (400 MHz, $CDCl_3$)

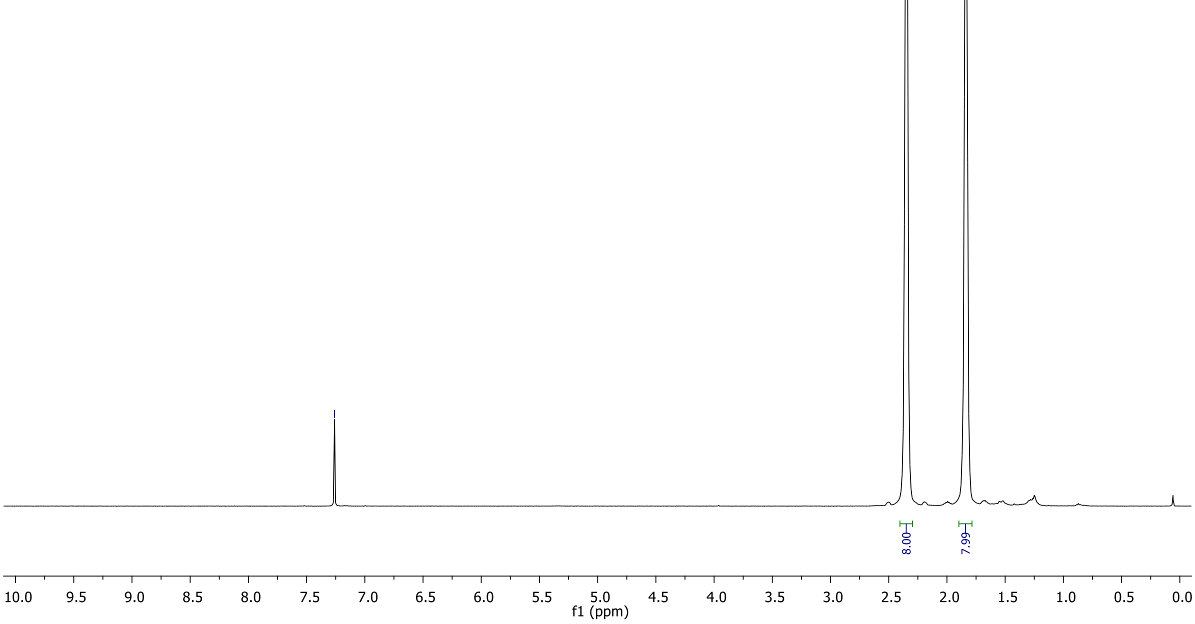

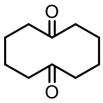

S28

^{13}C NMR (101 MHz, $CDCl_3$)

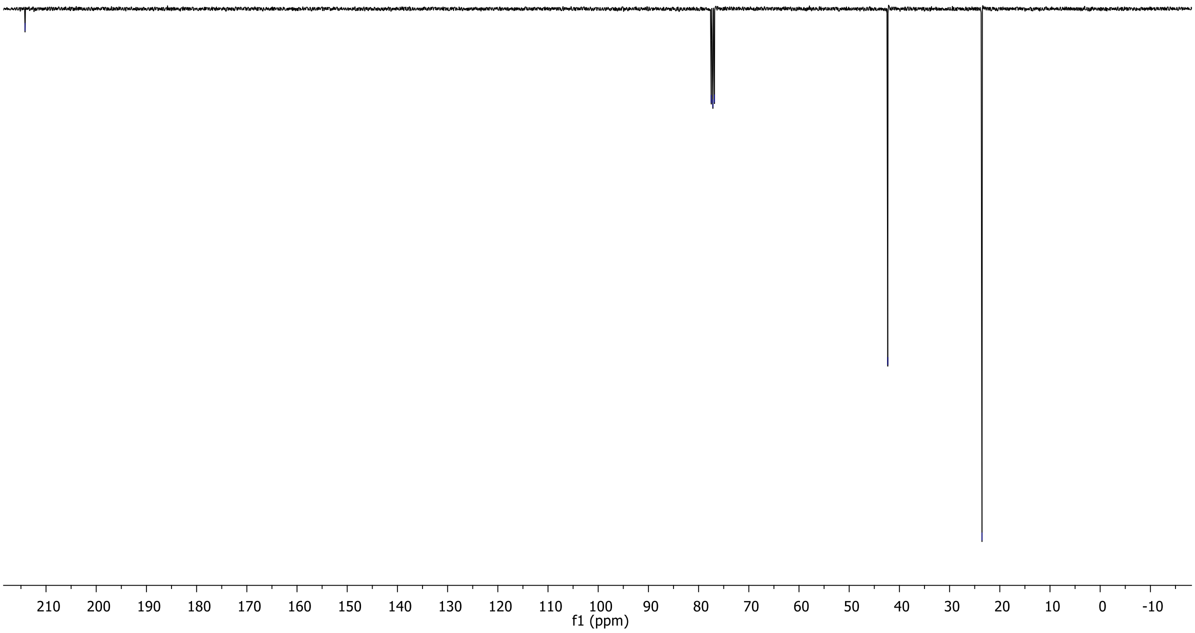

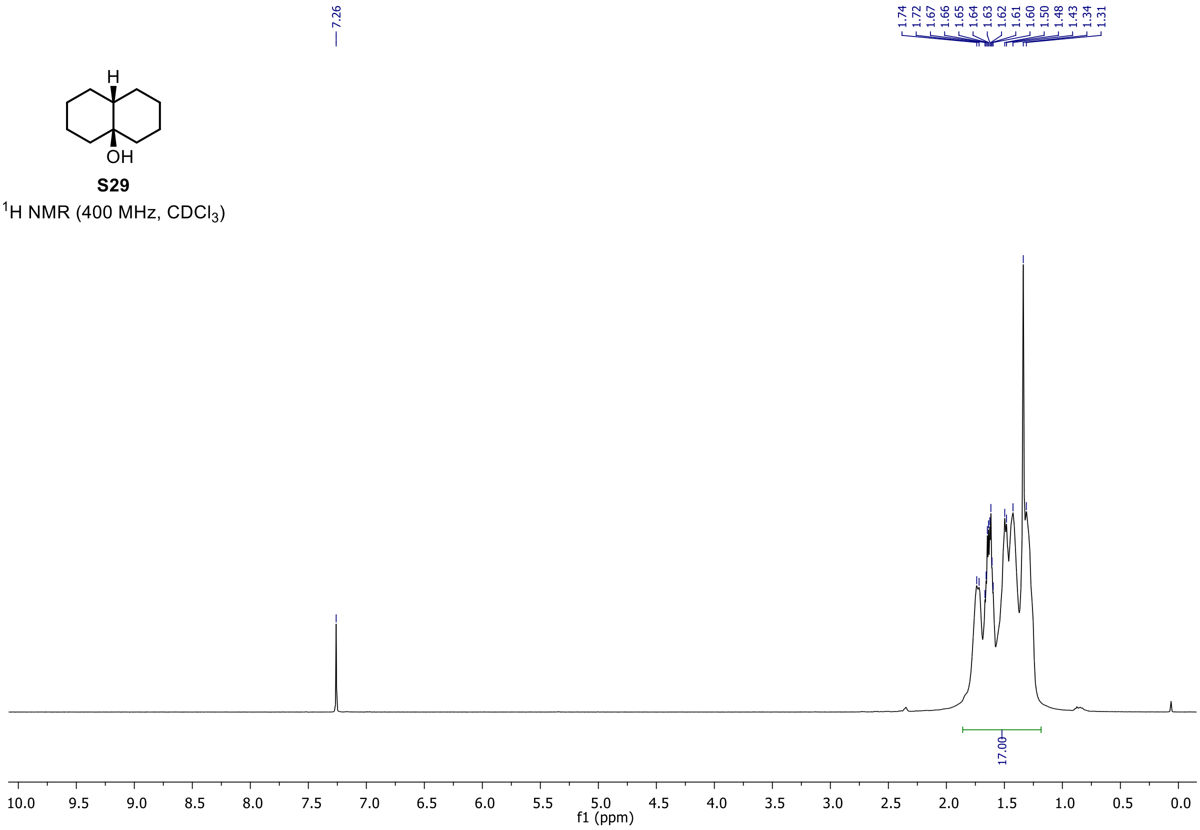

S29
^1H NMR (400 MHz, $CDCl_3$)

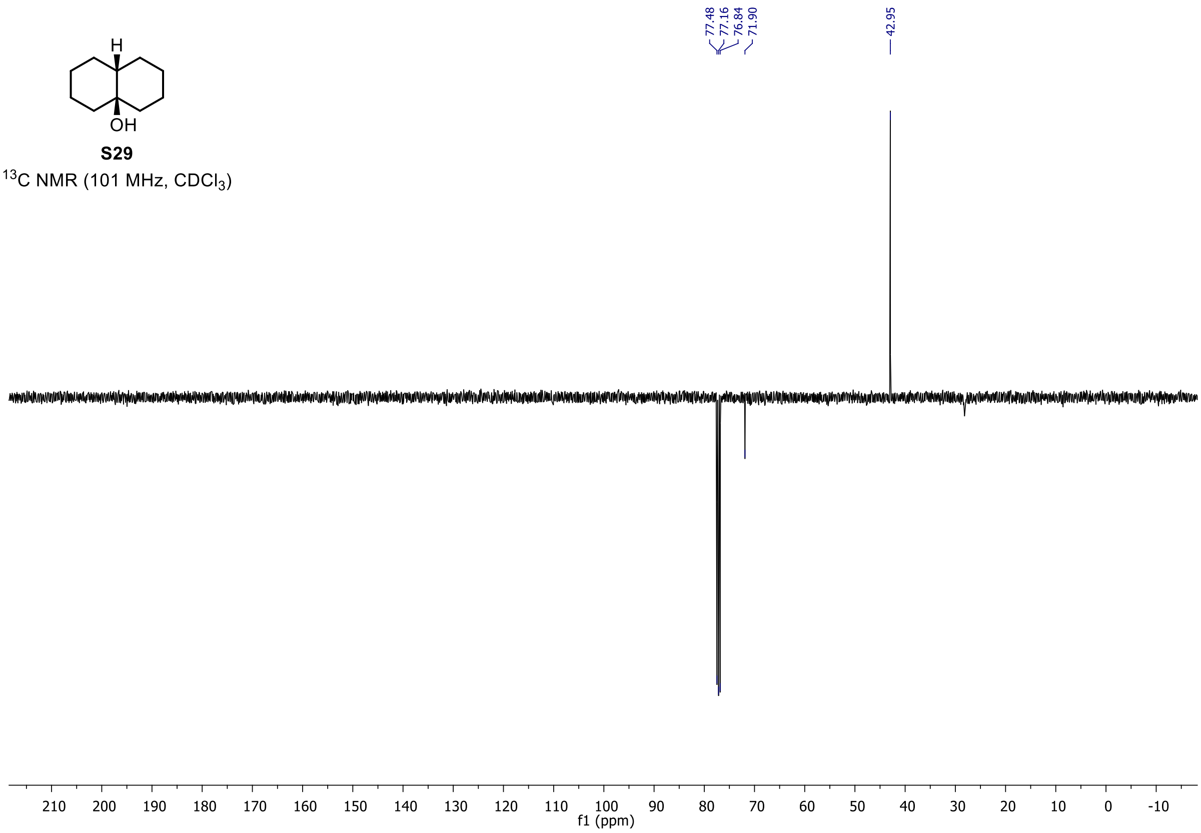

S29
^{13}C NMR (101 MHz, $CDCl_3$)

9-methyldecalin (S20)

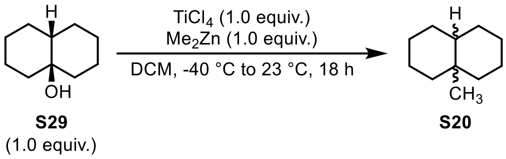

Alcohol **S29** (231 mg, 1.50 mmol, 1.00 equiv.) was dissolved in DCM (6 mL) and the solution was cooled to –40 °C (acetone bath cooled with dry ice). Titanium (IV) chloride (1 M in DCM, 1.50 mL, 1.50 mmol, 1.00 equiv.) was added dropwise, followed by dimethylzinc (2 M in PhMe, 0.75 mL, 1.50 mmol, 1.00 equiv.) at –40 °C. The dark red solution was allowed to slowly warm to ambient temperature (23 °C) and stirred for 18 h. The dark red solution was then diluted with pentane and the excess organometallic reagents were quenched by the addition of aqueous saturated $NaHCO_3$ (10 mL). The biphasic mixture was separated and the aqueous layer was extracted with DCM (3 × 10 mL). The combined organic layers were washed with brine (20 mL), dried over anhydrous sodium sulfate, the dried solution was filtered, and the filtrate was concentrated under reduced pressure (min. 200 mbar, water bath 50 °C). The crude residue was purified by flash column chromatography (pentane) to afford **S20** as a colorless oil and a mixture of diastereomers (141 mg, 62%, *dr* ~ 1:1.4).

The product is volatile and slowly evaporates already at room temperature under atmospheric pressure, therefore caution is to be taken during evaporation.

^{a} and ^{b} denote signals arising from exclusively the major (*trans*) or minor (*cis*) isomer, respectively; undesignated signals arise from both isomers or cannot be assigned to either of them.

The cis-isomer undergoes conformational changes (ring-flipping) on NMR time scale, therefore some signals are missing in ^{13}C NMR spectrum.

^{1}H NMR (400 MHz, $CDCl_3$) δ 1.73 – 1.65 (m, 1.40H^a), 1.56 – 1.43 (m, 5.60H), 1.33 – 1.18 (m, 6.11H), 1.17 – 1.00 (m, 3.40H), 0.96 (s, 1.25H), 0.91 – 0.85 (m, 1H), 0.72 (s, 1.75H).

^{13}C NMR (101 MHz, $CDCl_3$) δ 45.9 (CH^a), 42.2 (CH_2^{a}), 41.4 (CH^b), 29.2 (CH_2), 28.2 (CH_3^{b}), 27.9 (C^a), 27.3 (CH_2), 22.4 (CH_2), 22.1 (CH_2), 15.8 (CH_3^{a}).

IR (neat) ν_{max}: 2973, 2921, 2853, 1464, 1447.

HRMS (EI$^+$): exact mass calculated for $[M]^{\cdot+}$ $(C_{11}H_{20})^{\cdot+}$ requires *m*/*z* 152.1560, found *m*/*z* 152.1558.

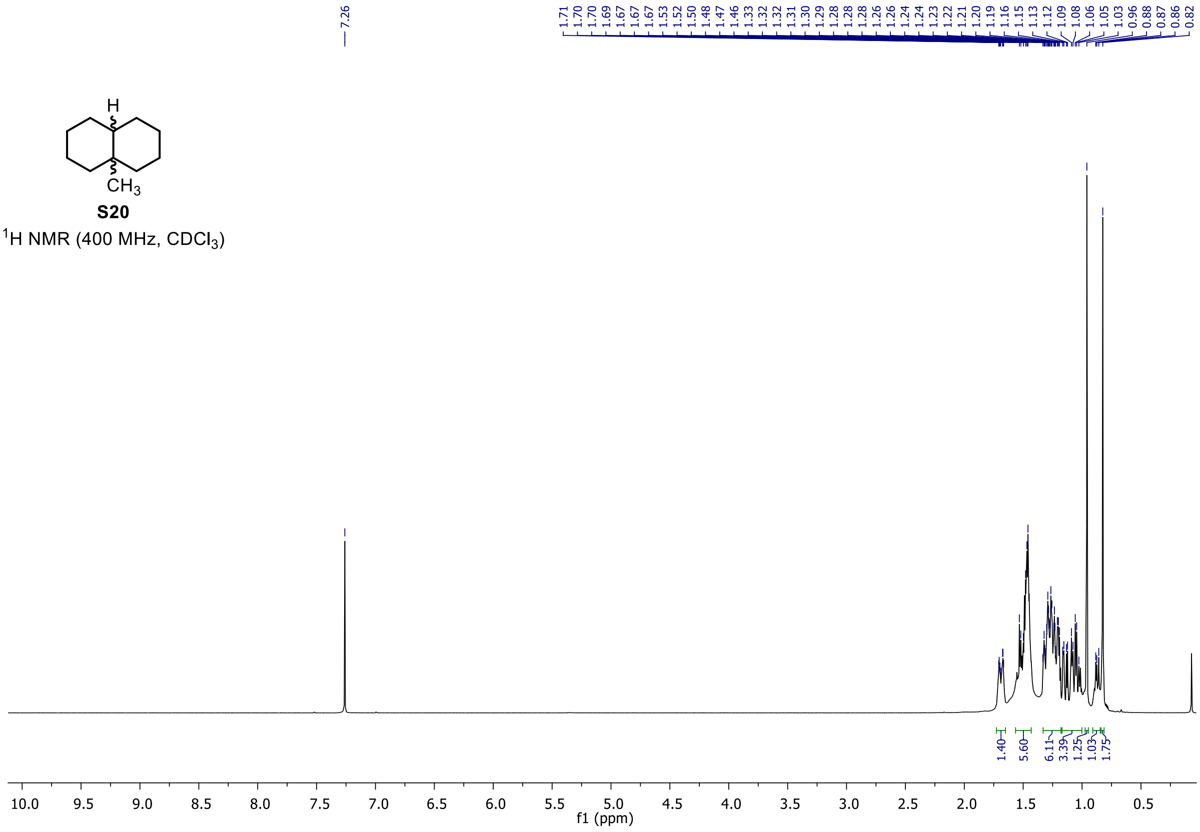

S20

^{1}H NMR (400 MHz, $CDCl_3$)

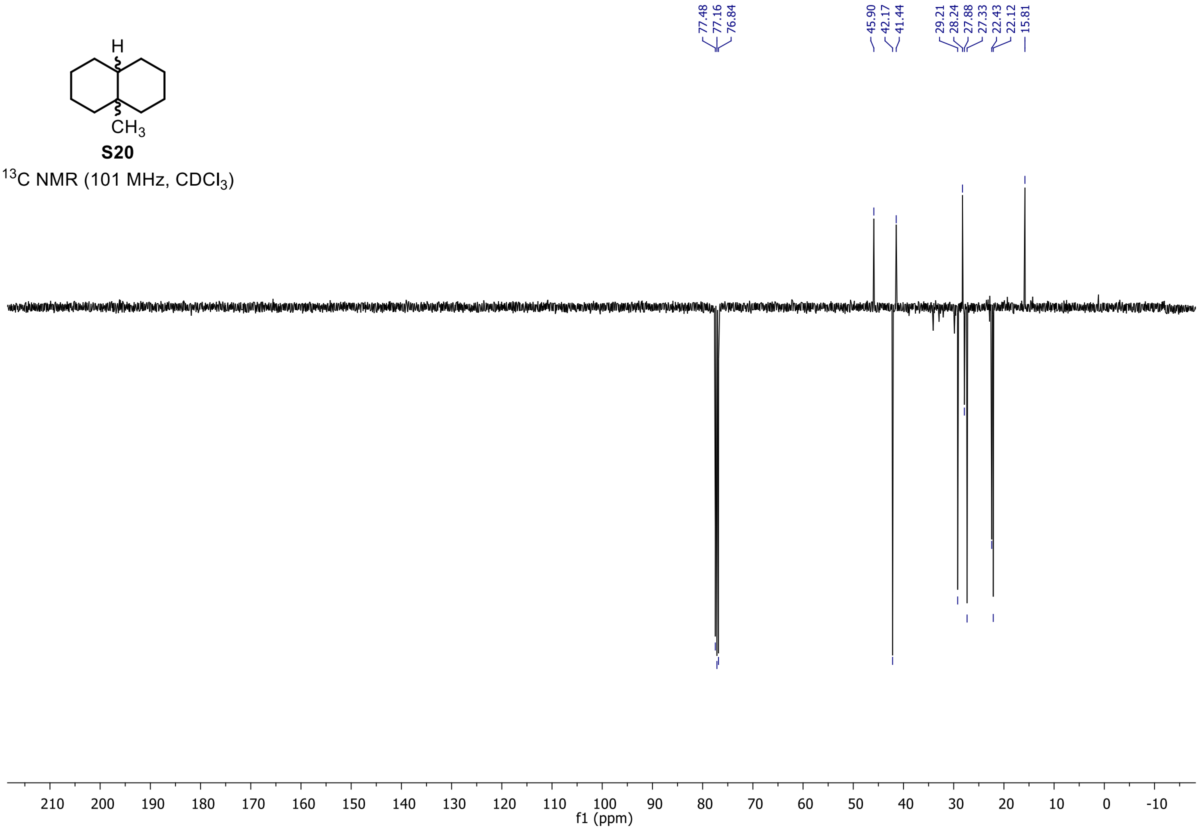

S20

^{13}C NMR (101 MHz, $CDCl_3$)

2,2-dimethyldecalin (S22)

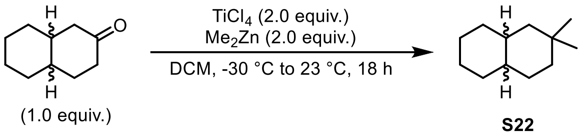

2-decalone (300 mg, 1.97 mmol, 1.00 equiv.) was dissolved in DCM (10 mL) and the solution was cooled to –30 °C (acetone bath cooled with dry ice). Titanium(IV) chloride (1 M in DCM, 3.94 mL, 3.94 mmol, 2.00 equiv.) was added dropwise, followed by dimethylzinc (2 M in PhMe, 1.97 mL, 3.94 mmol, 2.00 equiv.) at –30 °C. The dark red solution was allowed to slowly warm to ambient temperature (23 °C) and stirred for 18 h. The dark red solution was diluted with pentane and the unreacted organometallic reagents were quenched by the addition of aqueous saturated $NaHCO_3$ (15 mL). The biphasic mixture was separated and the aqueous layer was extracted with DCM (3 × 15 mL). The combined organic layers were washed with brine (25 mL), dried over anhydrous sodium sulfate, the dried solution was filtered, and the filtrate was concentrated under reduced pressure (min. 200 mbar, water bath 50 °C). The crude residue was purified by flash column chromatography (pentane) to afford **S22** as a colorless oil and a mixture of diastereomers (211 mg, 64%, *dr* ~ 1:1.6).

^{a} and ^{b} denote signals arising from exclusively the major (*trans*) or minor (*cis*) isomer, respectively; undesignated signals arise from both isomers or cannot be assigned to either of them.

The cis-isomer undergoes conformational changes (ring-flipping) on NMR time scale, therefore some signals appear broad and weak in ^{13}C NMR spectrum.

^{1}H NMR (400 MHz, $CDCl_3$) δ 1.89 – 1.81 (m, 0.75H), 1.72 – 1.46 (m, 6.54H), 1.43 – 1.20 (m, 6.92H), 1.16 – 1.03 (m, 2.17H), 0.90 (s, 2.30H^b), 0.89 (s, 3.70H^a).

^{13}C NMR (101 MHz, $CDCl_3$) δ 47.6 (CH_2^b), 39.7 (CH_2^b), 39.1 (CH_2^a), 35.8 (CH_3^b), 34.5 (CH_2^b), 34.0 (CH_2^a), 33.9 (C^a), 33.7 (CH^a), 33.6 (CH^b), 32.3 (CH_2^a), 31.6 (CH^a), 31.2 (CH_2^b), 30.3 (CH_2^b), 28.5 (CH_2^a), 27.1 (CH_2^a), 27.0 (CH_2^b), 26.9 (CH_2^b), 25.6 (CH_2^a), 25.2 (CH^b), 25.1 (CH^a), 21.2 (CH_2^a).

IR (neat) v_{max}: 2916, 2852, 1448, 737.

HRMS (EI$^+$): exact mass calculated for $[M]^{\cdot+}$ $(C_{12}H_{22})^{\cdot+}$ requires *m/z* 166.1716, found *m/z* 166.1713.

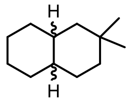

S22

^{1}H NMR (400 MHz, $CDCl_3$)

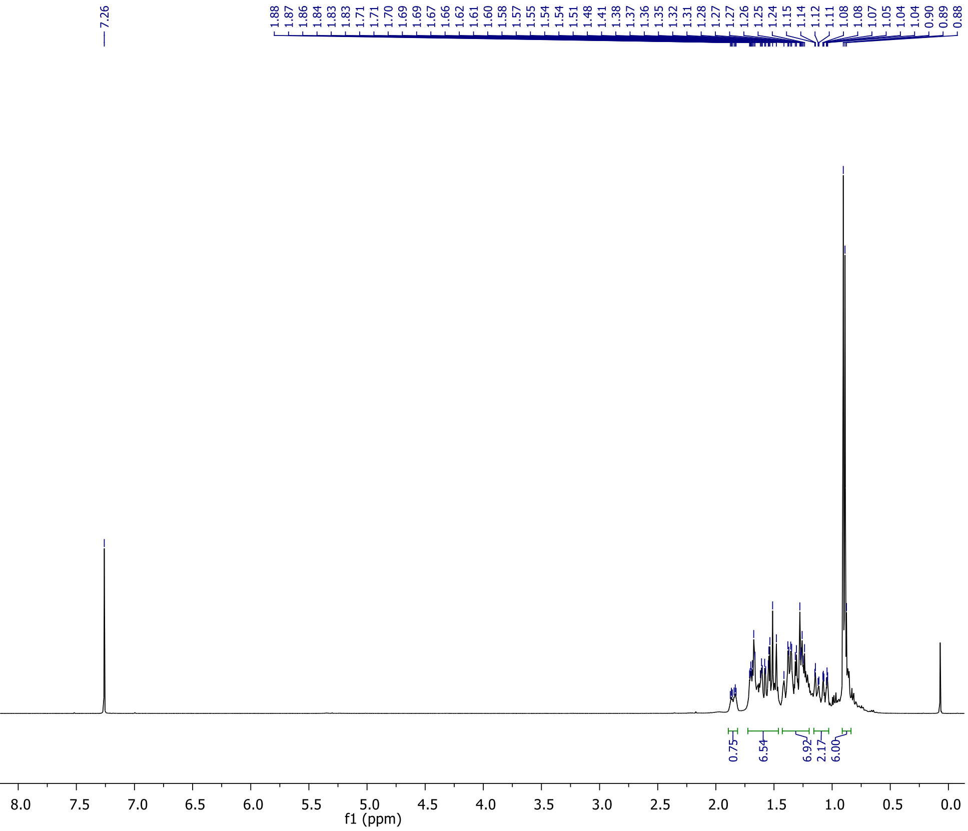

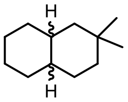

S22

^{13}C NMR (101 MHz, $CDCl_3$)

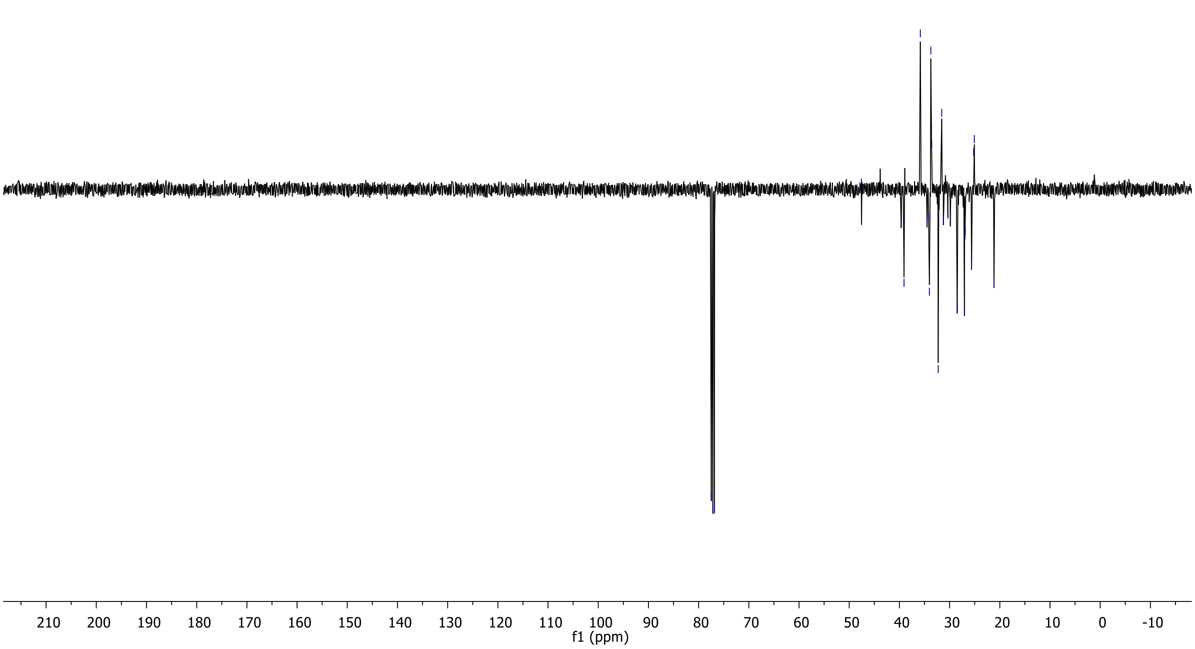

2,3,5,6,7,8-hexahydroazulen-4(1H)-one (S30)

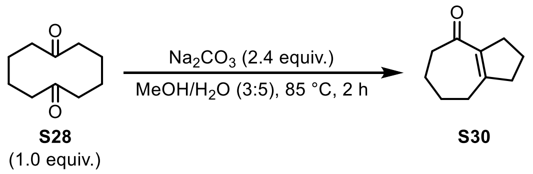

Enone **S30** was prepared following a procedure reported by House *et al*.[47] All analytical data were in accordance with data reported in the literature.[47]

¹H NMR (400 MHz, $CDCl_3$) δ 2.66 – 2.53 (m, 6H), 2.44 (app s, 2H), 1.84 – 1.73 (m, 6H).

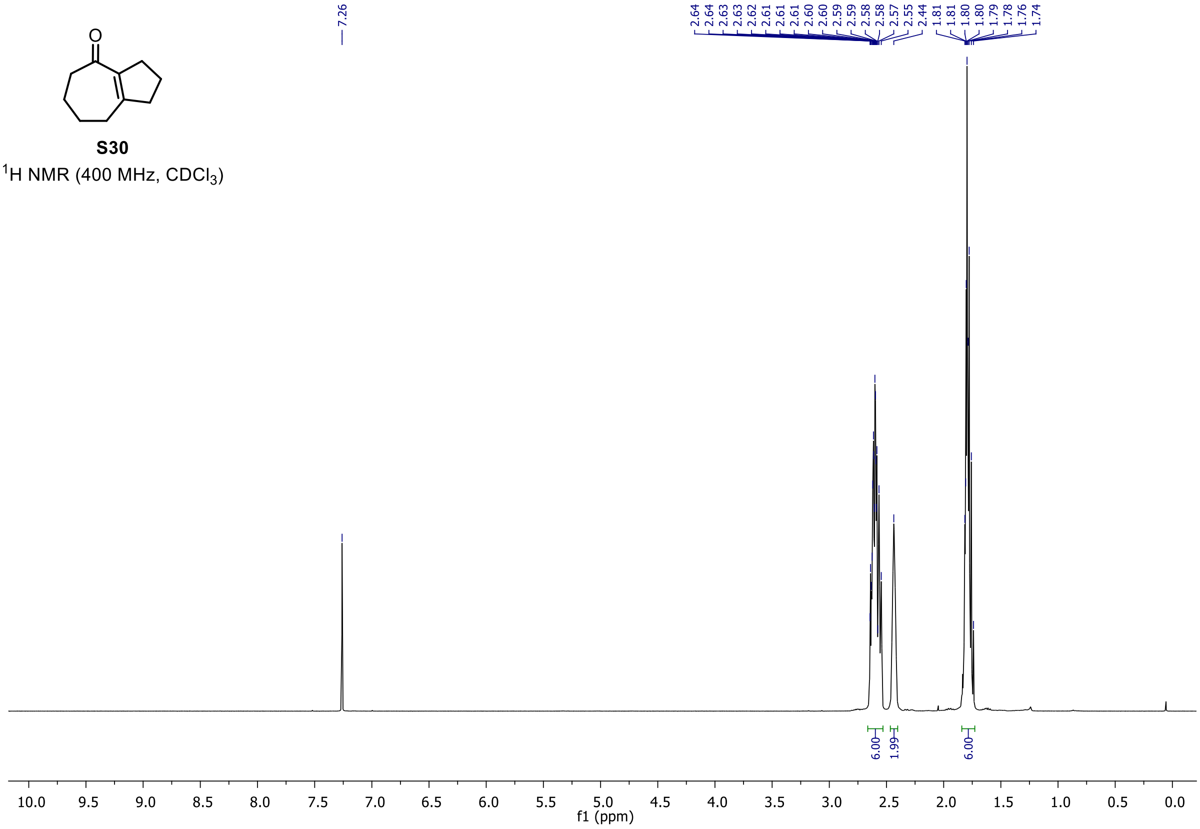

octahydroazulen-4(1H)-one (S31)

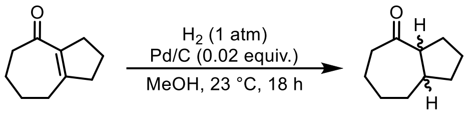

S30
(1.00 equiv.)

S31

Enone **S30** (485 mg, 3.23 mmol, 1.00 equiv.) was dissolved in MeOH (8 mL) and Pd/C (69 mg, 64.60 µmol, 0.02 equiv.) was added at ambient temperature (23 °C). The dark suspension was stirred for 18 h under H_2 atmosphere (balloon pressure, vessel initially flushed 3 times with H_2) at ambient temperature (23 °C) and then filtered through a short pad of Celite which was washed with additional 10 mL MeOH. The solution was concentrated under reduced pressure (min. 150 mbar, water bath 50 °C) and the crude residue was purified by flash column chromatography (0 – 8% Et_2O in pentane) to afford **S31** as colorless oil and mixture of diastereomers (278 mg, 57%, *dr* 3.2:1).

The product is volatile and slowly evaporates already at room temperature under atmospheric pressure, therefore caution is to be taken during evaporation.

^{a} and ^{b} denote signals arising from exclusively the major or minor isomer, respectively; undesignated signals arise from both isomers or cannot be assigned to either of them.

^{1}H NMR (400 MHz, CD_2Cl_2) δ 3.10 (dt, *J* = 10.5, 8.3 Hz, 0.24H^b), 2.77 (dt, *J* = 10.6, 8.2 Hz, 0.76H^a), 2.56 – 2.43 (m, 1.05H), 2.39 – 2.27 (m, 1.26H), 2.16 – 2.05 (m, 1.53H), 2.03 – 1.81 (m, 3.24H), 1.77 – 1.66 (m, 1.74H), 1.64 – 1.23 (m, 6.31H), 1.17 – 1.03 (m, 0.52H^b).

^{13}C NMR (101 MHz, CD_2Cl_2) δ 213.7 (C^b), 213.6 (C^a), 57.3 (CH^a), 54.9 (CH^b), 45.8 (CH^a), 44.2 (CH_2^a), 43.6 (CH_2^b), 40.7 (CH^b), 37.2 (CH_2^a), 36.1 (CH_2^a), 35.6 (CH_2^b), 33.0 (CH_2^b), 29.7 (CH_2^a), 28.2 (CH_2^b), 26.64 (CH_2^a), 26.59 (CH_2^b), 25.8 (CH_2^b), 24.9 (CH_2^b), 24.5 (CH_2^a), 24.0 (CH_2^a).

IR (neat) ν_{max}: 2928, 2859, 1700, 1449, 766.

HRMS (ESI$^+$): exact mass calculated for $[M+Na]^+$ ($C_{10}H_{16}ONa$) requires *m/z* 175.1093, found *m/z* 175.1092.

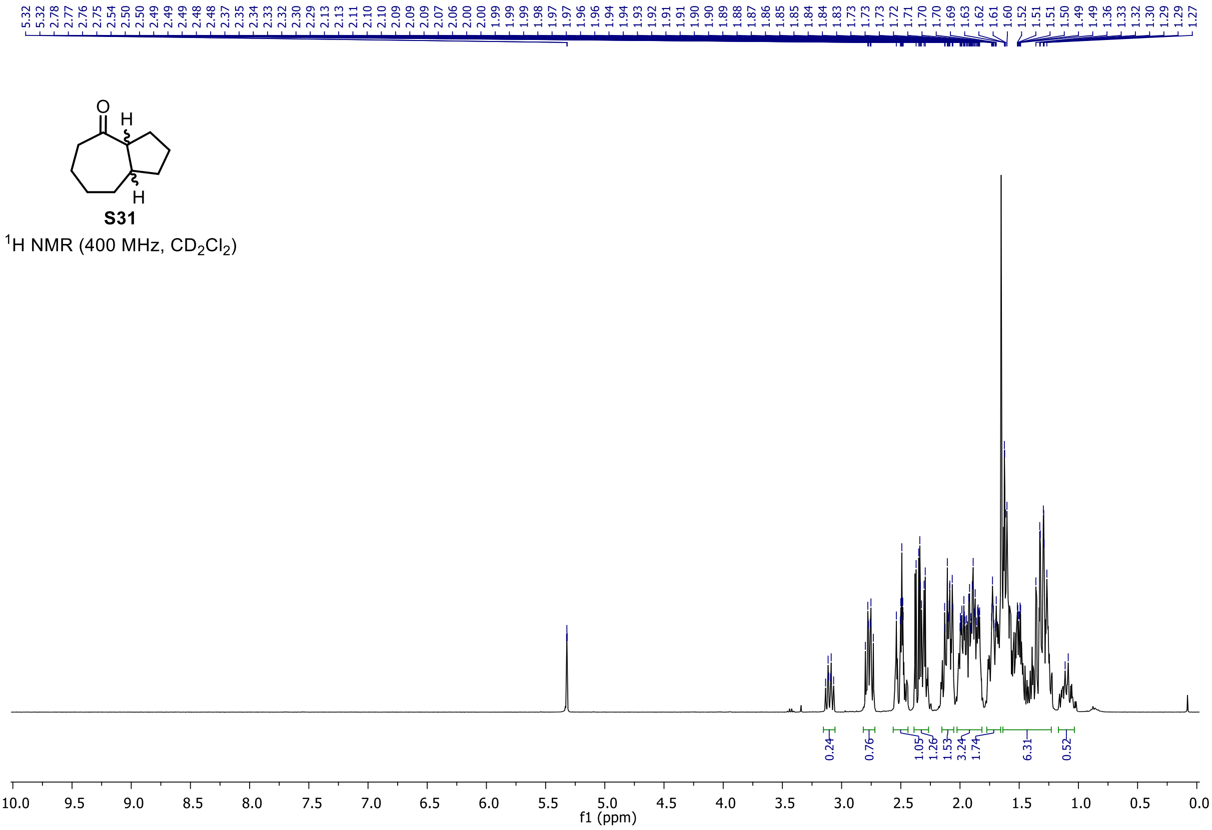
5.32 5.32 2.78 2.77 2.76 2.75 2.54 2.50 2.50 2.49 2.49 2.49 2.48 2.37 2.35 2.34 2.33 2.32 2.30 2.29 2.13 2.13 2.11 2.10 2.10 2.09 2.09 2.09 2.07 2.06 2.00 2.00 1.99 1.99 1.99 1.98 1.97 1.97 1.96 1.96 1.94 1.94 1.93 1.92 1.91 1.91 1.90 1.89 1.88 1.87 1.86 1.85 1.85 1.84 1.84 1.83 1.83 1.73 1.73 1.73 1.72 1.71 1.70 1.70 1.69 1.63 1.62 1.61 1.60 1.52 1.51 1.51 1.50 1.49 1.36 1.33 1.32 1.30 1.29 1.29 1.27
S31
^{1}H NMR (400 MHz, CD_2Cl_2)
0.24 0.76 1.05 1.26 1.53 3.24 1.74 6.31 0.52
10.0 9.5 9.0 8.5 8.0 7.5 7.0 6.5 6.0 5.5 5.0 4.5 4.0 3.5 3.0 2.5 2.0 1.5 1.0 0.5 0.0
f1 (ppm)

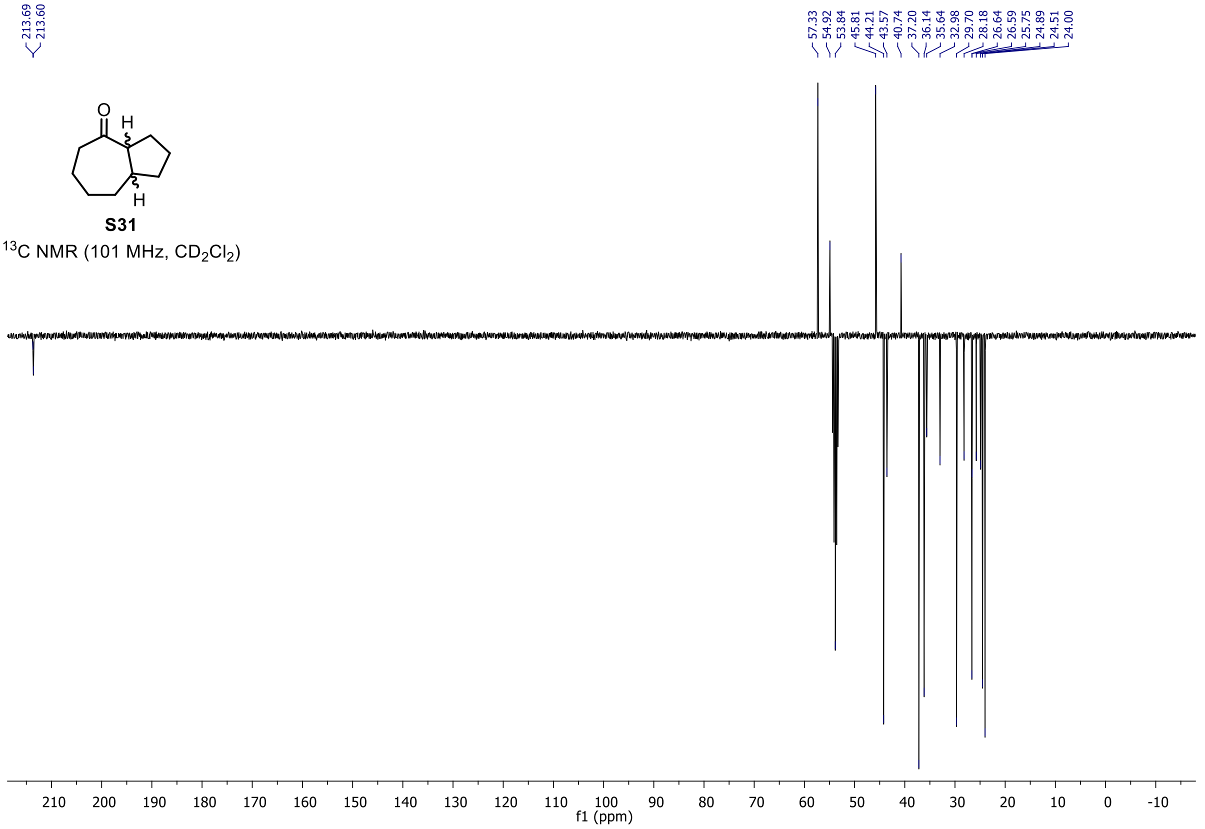
213.69 213.60
57.33 54.92 53.84 45.81 44.21 43.57 40.74 37.20 36.14 35.64 32.98 29.70 28.18 26.64 26.59 25.75 24.89 24.51 24.00
S31
^{13}C NMR (101 MHz, CD_2Cl_2)
210 200 190 180 170 160 150 140 130 120 110 100 90 80 70 60 50 40 30 20 10 0 -10
f1 (ppm)

***trans*-decahydroazulene (S23)**

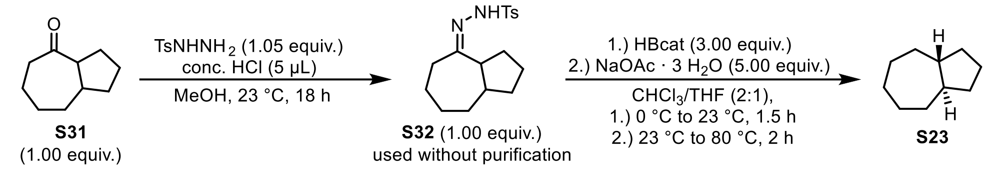

Ketone **S31** (260 mg, 1.71 mmol, 1.00 equiv.) was dissolved in MeOH (1.5 mL) and tosylhydrazine (334 mg, 1.79 mmol, 1.05 equiv.) was added in one portion at ambient temperature. 5 µL of concentrated HCl (37% in H_2O) were added and the resulting solution was stirred 18 h at ambient temperature (23 °C). The colorless precipitate was filtered through a glass frit, washed with 5 mL of pentane and dried at <1 mbar over 3 h at 23 °C to afford hydrazone **S32** as a colorless solid which was used without further purification (301 mg, ~0.94 mmol).

Crude hydrazone **S32** (288 mg, ~0.90 mmol, 1.00 equiv.) was dissolved in a mixture of $CHCl_3$ (6 mL) and THF (3 mL) at ambient temperature (23 °C) and the solution was cooled to 0 °C. Catecholborane (0.29 mL, 2.70 mmol, 1.00 equiv.) was added dropwise at 0 °C and the resulting solution was stirred for 30 min at 0 °C and then 1 h at ambient temperature (23 °C). Sodium acetate trihydrate (611 mg, 4.49 mmol, 5.00 equiv.) was added in one portion at ambient temperature (23 °C) and the resulting suspension was refluxed at 80 °C for 2 h, during which a beige voluminous solid precipitated (stirring stopped after 30 min). The reaction mixture was cooled to ambient temperature (23 °C), followed by the addition of aqueous saturated $NaHCO_3$ (15 mL). The biphasic mixture was separated and the aqueous layer was extracted with DCM (3 × 10 mL). The combined organic layers were washed with an aqueous saturated solution of NH_4Cl (20 mL) and brine (20 mL), dried over anhydrous sodium sulfate, the dried solution was filtered, and the filtrate was concentrated under reduced pressure (min. 150 mbar, water bath 50 °C). The crude residue was purified by flash column chromatography (pentane) to afford **S23** as a colorless oil (87 mg, 39% over 2 steps).

The product is volatile and slowly evaporates already at room temperature under atmospheric pressure, therefore caution is to be taken during evaporation.

All analytical data were in good accordance with data reported in the literature.[49]

^{1}H NMR (700 MHz, CD_2Cl_2) δ 1.90 – 1.78 (m, 4H), 1.67 – 1.60 (m, 2H), 1.57 – 1.44 (m, 8H), 1.23 – 1.12 (m, 4H).

^{13}C NMR (176 MHz, CD_2Cl_2) δ 46.8 (2CH), 35.6 (2CH_2), 34.8 (2CH_2), 28.4 (2CH_2), 27.1 (CH_2), 24.4 (CH_2).

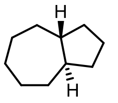

S23

^{1}H NMR (700 MHz, CD_2Cl_2)

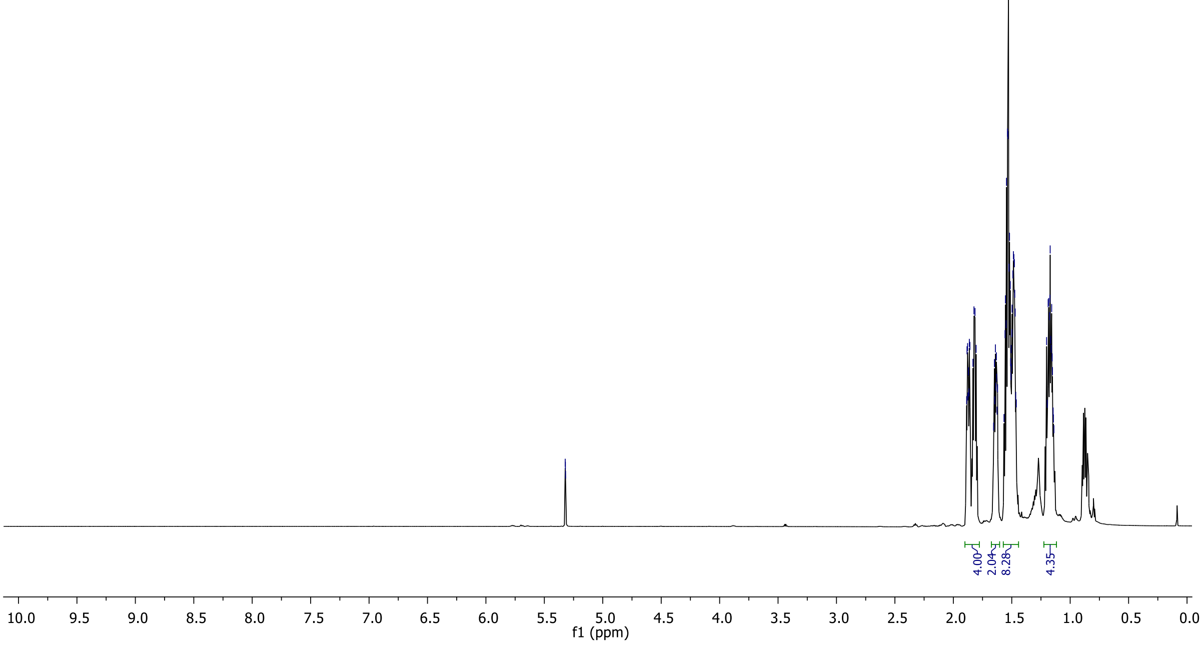

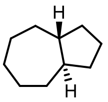

S23

^{13}C NMR (176 MHz, CD_2Cl_2)

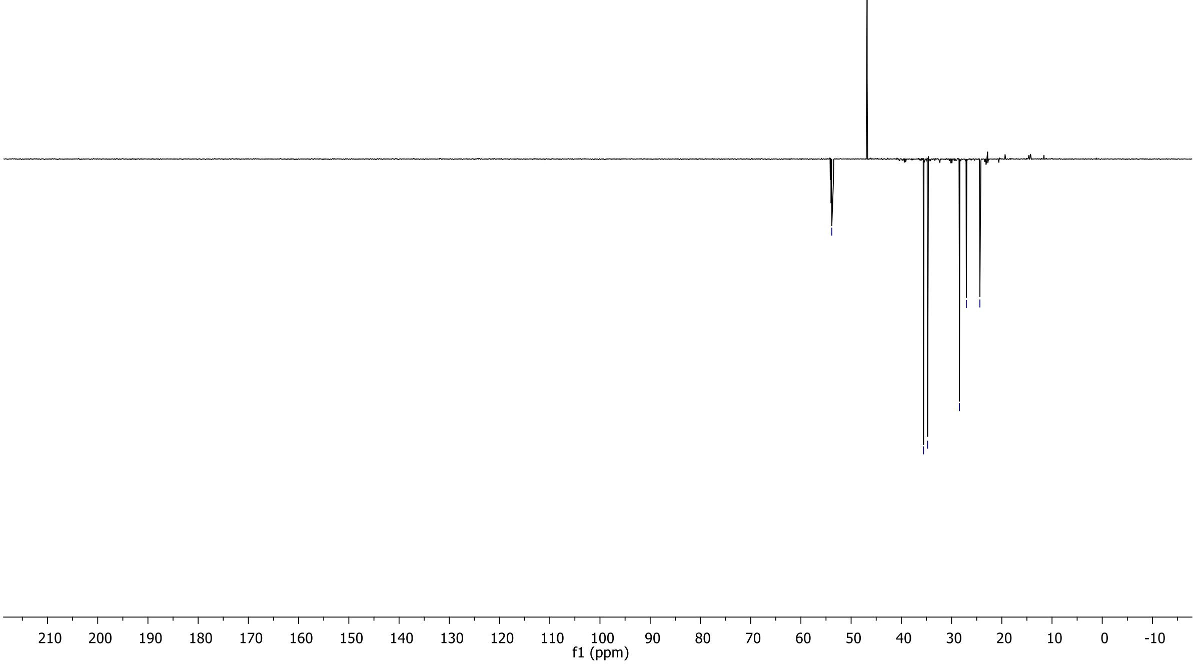

4.9 Functionalization reactions

Epimerization of 12 (*epi*-**12**)

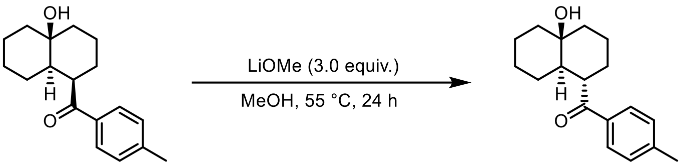

12
(1.0 equiv.)

epi-**12**

A flame-dried Schlenk flask equipped with a stirring bar and a rubber septum was charged with **12** (55 mg, 0.20 mmol, 1.00 equiv.) and transferred into a glovebox, where LiOMe (23 mg, 0.60 mmol, 3.00 equiv.) was added. The flask was closed with a septum, removed from glovebox and then dry MeOH (1 mL) was added at ambient temperature (23 °C). The resulting solution was heated to 55 °C for 24 h, after which the mixture was allowed to cool down to ambient temperature (23 °C). The reaction was stopped by the addition of saturated aqueous ammonium chloride (3 mL), the resulting suspension was transferred into a separation funnel and extracted with Et_2O (3 × 5 mL). The combined organic layers were washed with brine (10 mL), dried over anhydrous sodium sulfate, the dried solution was filtered, and the filtrate was concentrated under reduced pressure. Purification by flash column chromatography (3 – 30% EtOAc in heptanes) afforded *epi*-**12** as a colorless viscous oil (45 mg, 83%).

$\mathbf{R_f}$ = 0.28 in 20% EtOAc/heptanes

^{1}H NMR (400 MHz, $CDCl_3$) δ 7.89 (d, *J* = 8.2 Hz, 2H), 7.25 (d, *J* = 8.9 Hz, 2H), 3.59 – 3.51 (m, 1H), 2.40 (s, 3H), 1.91 – 1.72 (m, 3H), 1.65 – 1.53 (m, 6H), 1.50 – 1.38 (m, 3H), 1.35 – 1.16 (m, 4H).

^{13}C NMR (101 MHz, $CDCl_3$) δ 204.7 (C), 143.9 (C), 135.3 (C), 129.4 (2CH), 128.6 (2CH), 70.4 (C), 45.8 (CH), 45.4 (CH), 40.3 (CH_2), 39.6 (CH_2), 31.2 (CH_2), 26.1 ($2CH_2$), 21.7 (CH_3), 21.6 (CH_2), 21.0 (CH_2).

IR (neat) ν_{max}: 3494, 2925, 2855, 1661, 1605, 1284, 1198, 1181, 1152, 947, 826, 747.

HRMS (ESI$^+$): exact mass calculated for $[M+Na]^+$ ($C_{18}H_{24}O_2Na$) requires *m*/*z* 295.1669, found *m*/*z* 295.1658.

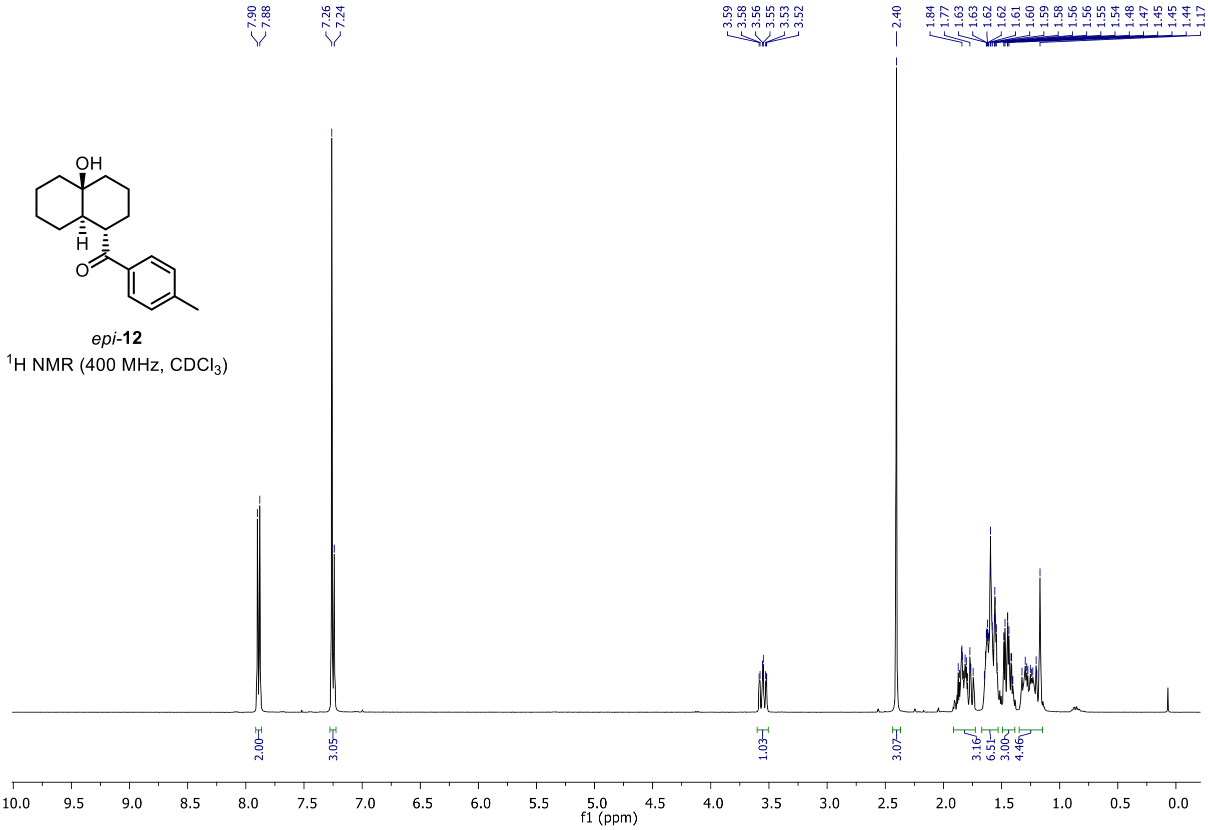

epi-**12**

^{1}H NMR (400 MHz, $CDCl_3$)

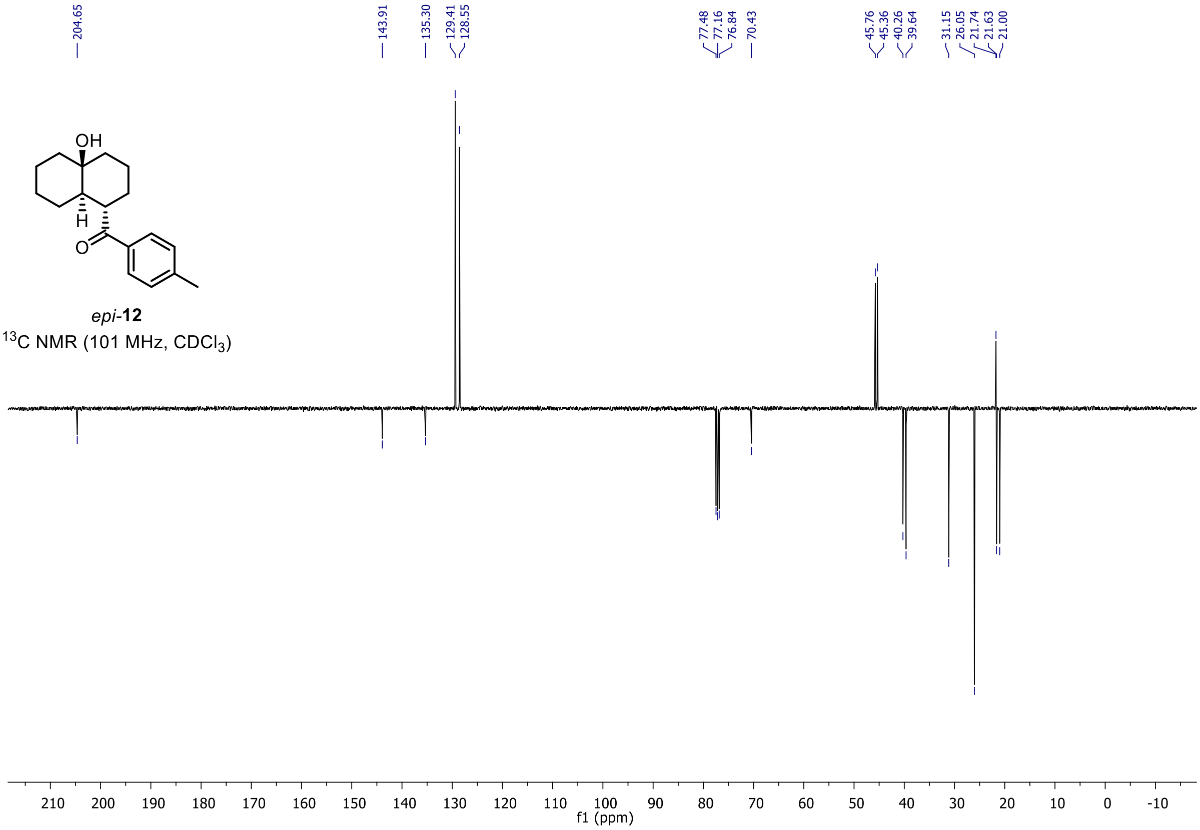

epi-**12**

^{13}C NMR (101 MHz, $CDCl_3$)

Reduction of *epi*-12 with L-selectride (40)

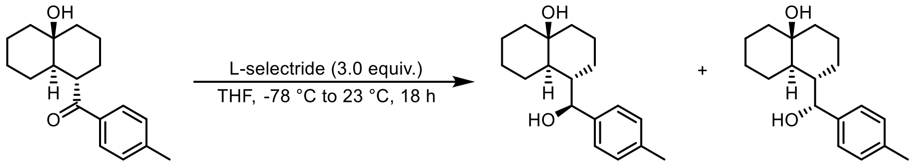

In a small flame-dried vial with a rubber septum cap, *epi*-**12** (13.6 mg, 0.05 mmol, 1.00 equiv.) was dissolved in THF (0.5 mL) and the resulting solution was cooled to -78 °C. L-selectride (1 M in THF, 0.15 mL, 0.15 mmol, 3.00 equiv.) was added under stirring at -78 °C, the mixture was stirred 1 h at -78 °C, then warmed up to ambient temperature (23 °C, cooling bath removed) and stirred for 17 h. The reaction was stopped by the addition of 1 M aqueous NaOH solution (2.5 mL) at ambient temperature (23 °C), followed by the addition of 30% aqueous H_2O_2 (70 µL). After stirring for 30 min, the mixture was extracted with EtOAc (3 × 5 mL), the combined organic layers were washed with aqueous saturated sodium thiosulfate solution (5 mL), dried over anhydrous sodium sulfate, the dried solution was filtered, and the filtrate was concentrated under reduced pressure. *dr* was determined by ^{1}H NMR analysis of the crude reaction product using mesitylene as internal standard (**40**:**40'** > 20:1). Purification by flash column chromatography (10 – 55% EtOAc in heptanes) afforded **40** as a colorless crystalline solid (10.1 mg, 74%).

A crystal of sufficient quality (**40**) for X-ray diffractometry was obtained by slow evaporation from $CHCl_3$ (8 mg dissolved in approx. 2.0 mL $CHCl_3$ at 23 °C, vial loosely capped and stored at 23 °C until complete solvent evaporation).

R_f = 0.19 in 40% EtOAc/heptanes

m.p. (+5 °C/min) = 142.7 – 144.3 °C

^{1}H NMR (600 MHz, $CDCl_3$) δ 7.19 (d, *J* = 8.1 Hz, 2H), 7.14 (d, *J* = 8.0 Hz, 2H), 4.96 (d, *J* = 3.8 Hz, 1H), 2.35 (s, 3H), 2.06 – 2.01 (m, 1H), 1.94 – 1.88 (m, 1H), 1.88 – 1.83 (m, 1H), 1.82 – 1.56 (m, 4H), 1.53 – 1.43 (m, 4H), 1.39 – 1.31 (m, 1H), 1.26 – 1.17 (m, 3H), 1.11 (td, *J* = 13.6, 4.4 Hz, 1H), 0.93 (td, *J* = 11.8, 3.4 Hz, 1H), 0.76 (qd, *J* = 12.9, 3.9 Hz, 1H).

^{13}C NMR (151 MHz, $CDCl_3$) δ 139.3 (C), 136.9 (C), 128.7 (2CH), 126.7 (2CH), 74.2 (CH), 70.7 (C), 46.1 (CH), 44.2 (CH), 40.3 (CH_2), 40.1 (CH_2), 26.6 (CH_2), 26.2 (CH_2), 25.5 (CH_2), 21.3 (CH_2), 21.2 (CH_3), 20.8 (CH_2).

IR (neat) ν_{max}: 3354, 3200, 2921, 2852, 1661, 1039, 1016, 821, 803, 752.

HRMS (EI$^+$): exact mass calculated for $[M-H_2O]^{\cdot+}$ ($C_{18}H_{24}O$) requires *m*/*z* 256.1822, found *m*/*z* 256.1816.

SC-XRD: See Section **5**.

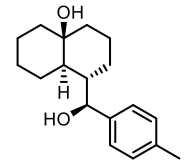

40

^{1}H NMR (600 MHz, $CDCl_3$)

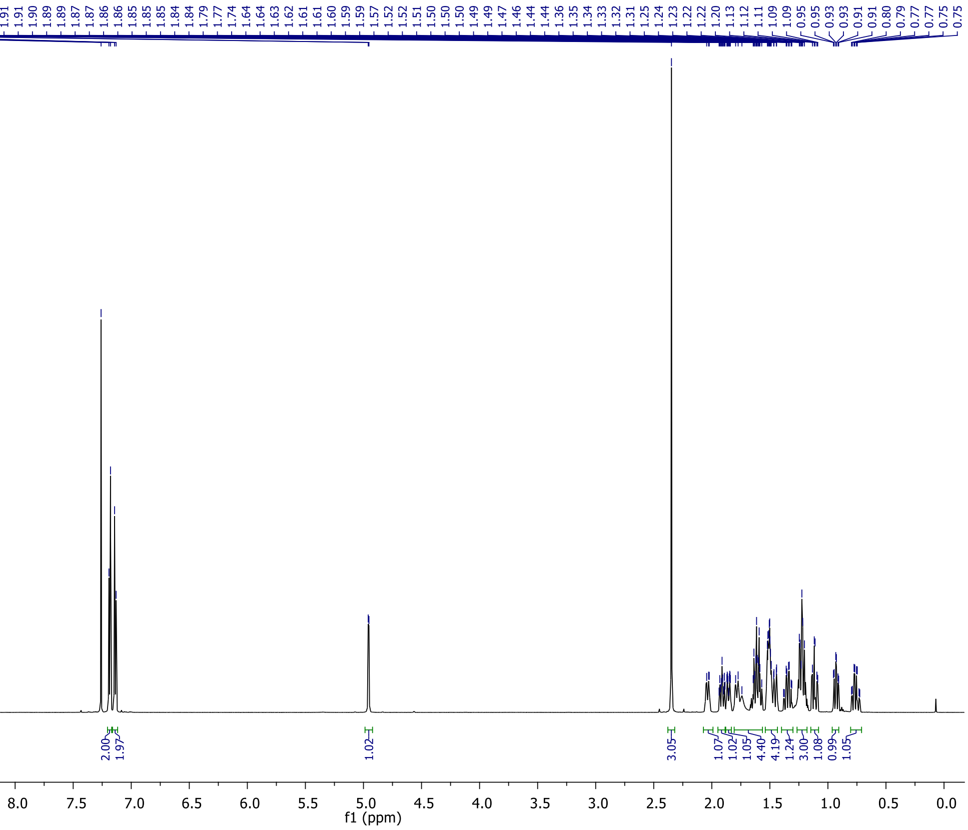

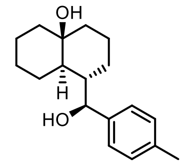

40

^{13}C NMR (151 MHz, $CDCl_3$)

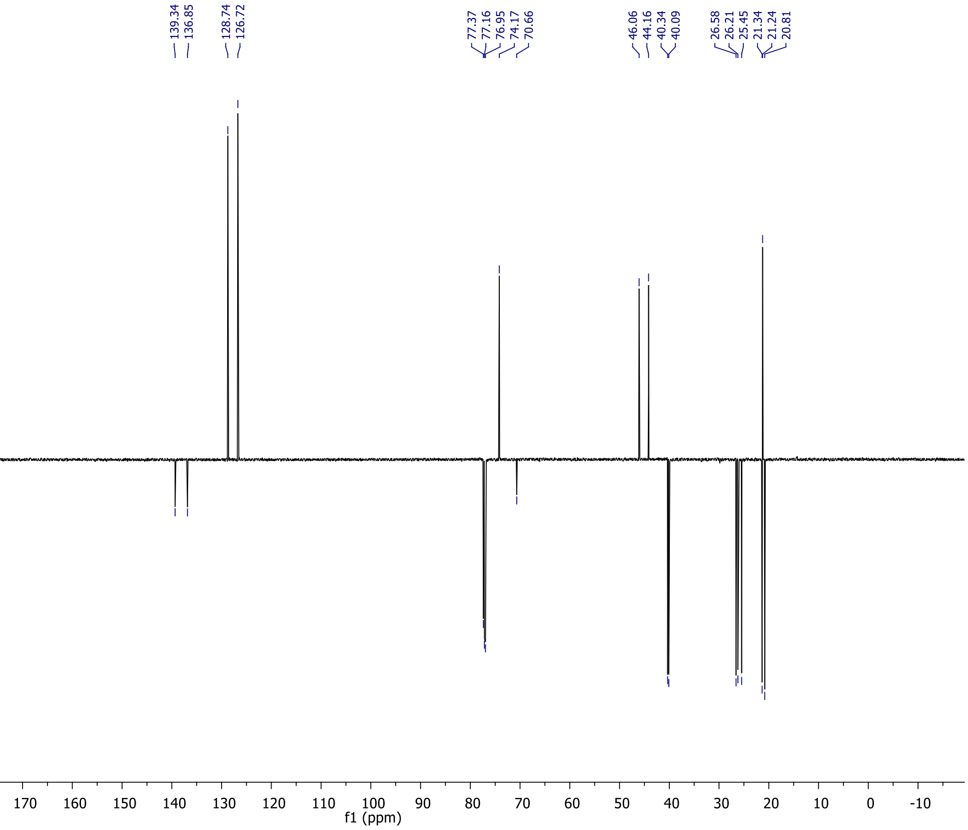

Water elimination to octalin (41)

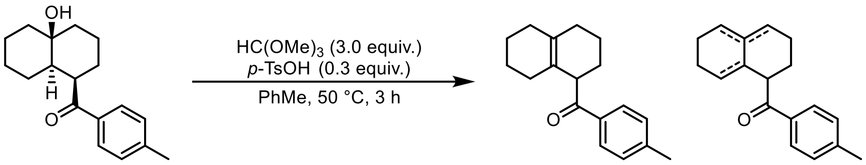

12 (1.0 equiv.) **41a** **41b**

In a flame-dried Schlenk flask, **12** (60 mg, 0.22 mmol, 1.00 equiv.) and *p*-toluenesulfonic acid (12.6 mg, 0.066 mmol, 0.30 equiv.) were dissolved in PhMe (2 mL) at ambient temperature (23 °C). Trimethyl orthoformate (72 µL, 0.66 mmol, 3.00 equiv.) was added and the solution was heated to 50 °C for 3 h. The reaction was stopped by the addition of 1 M aqueous NaOH solution (5 mL) and the mixture was extracted with Et_2O (3 × 5 mL). The combined organic layers were washed with brine (5 mL), dried over anhydrous sodium sulfate, the dried solution was filtered, and the filtrate was concentrated under reduced pressure. Purification by flash column chromatography (0 – 6% EtOAc in heptanes) afforded **41** as a colorless oil and a mixture of olefin isomers (54 mg, quant., **41a:41b** = 19:1).

^{a} denotes signals arising from **41a**, ^{b} denotes signals arising from **41b**. Undesignated signals arise from both isomers.

Due to low abundance, only signals arising from **41a** are reported in ^{13}C NMR.

R$_f$ = 0.53 in 10% EtOAc/heptanes

^{1}H NMR (400 MHz, CDCl$_3$) δ 7.90 (d, *J* = 8.3 Hz, 2H), 7.25 (d, *J* = 7.4 Hz, 2H), 5.47 – 5.43 (m, 0.05H^b), 3.96 – 3.89 (m, 0.95H^a), 3.68 – 3.58 (m, 0.05H^b), 3.30 – 3.25 (m, 0.14H^b), 2.41 (s, 3H), 2.10 – 1.50 (m, 14H).

^{13}C NMR (101 MHz, CDCl$_3$) δ 202.8 (C), 143.6 (C), 134.6 (C), 132.2 (C), 129.4 (2CH), 128.7 (2CH), 126.2 (C), 48.5 (CH), 30.9 (CH_2), 30.4 (CH_2), 29.5 (CH_2), 28.0 (CH_2), 23.3 (CH_2), 23.1 (CH_2), 21.7 (CH_3), 20.5 (CH_2).

IR (neat) ν_{max}: 2924, 2857, 2828, 1675, 1605, 1220, 1202, 1180, 967, 828, 780.

HRMS (EI$^+$): exact mass calculated for $[M]^{\cdot+}$ $(C_{18}H_{22}O)^{\cdot}$ requires *m*/*z* 254.1665, found *m*/*z* 254.1669.

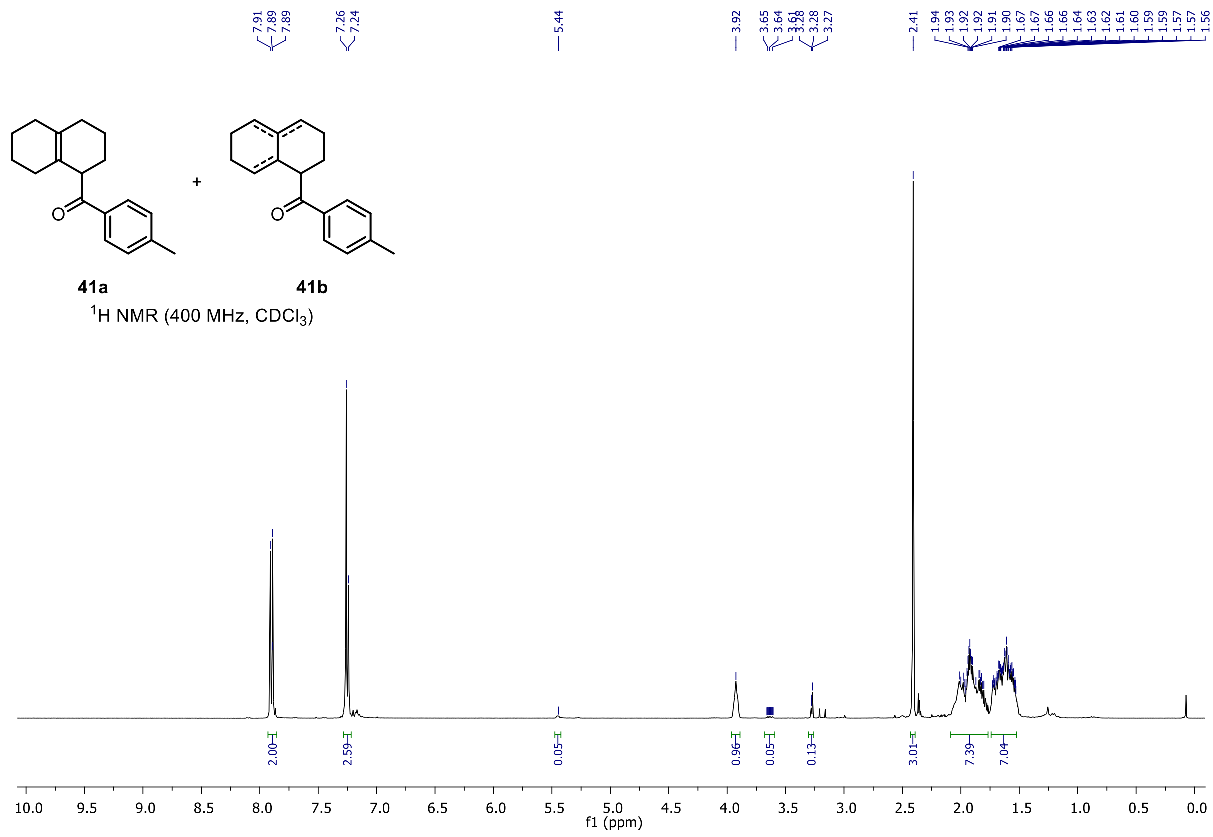
7.91
7.89
7.89
7.26
7.24
5.44
3.92
3.65
3.64
3.61
3.28
3.28
3.27
2.41
1.94
1.93
1.92
1.92
1.91
1.90
1.67
1.67
1.66
1.66
1.64
1.63
1.62
1.61
1.60
1.59
1.59
1.57
1.57
1.56
41a
41b
1H NMR (400 MHz, CDCl3)
2.00
2.59
0.05
0.96
0.05
0.13
3.01
7.39
7.04
10.0 9.5 9.0 8.5 8.0 7.5 7.0 6.5 6.0 5.5 5.0 4.5 4.0 3.5 3.0 2.5 2.0 1.5 1.0 0.5 0.0
f1 (ppm)

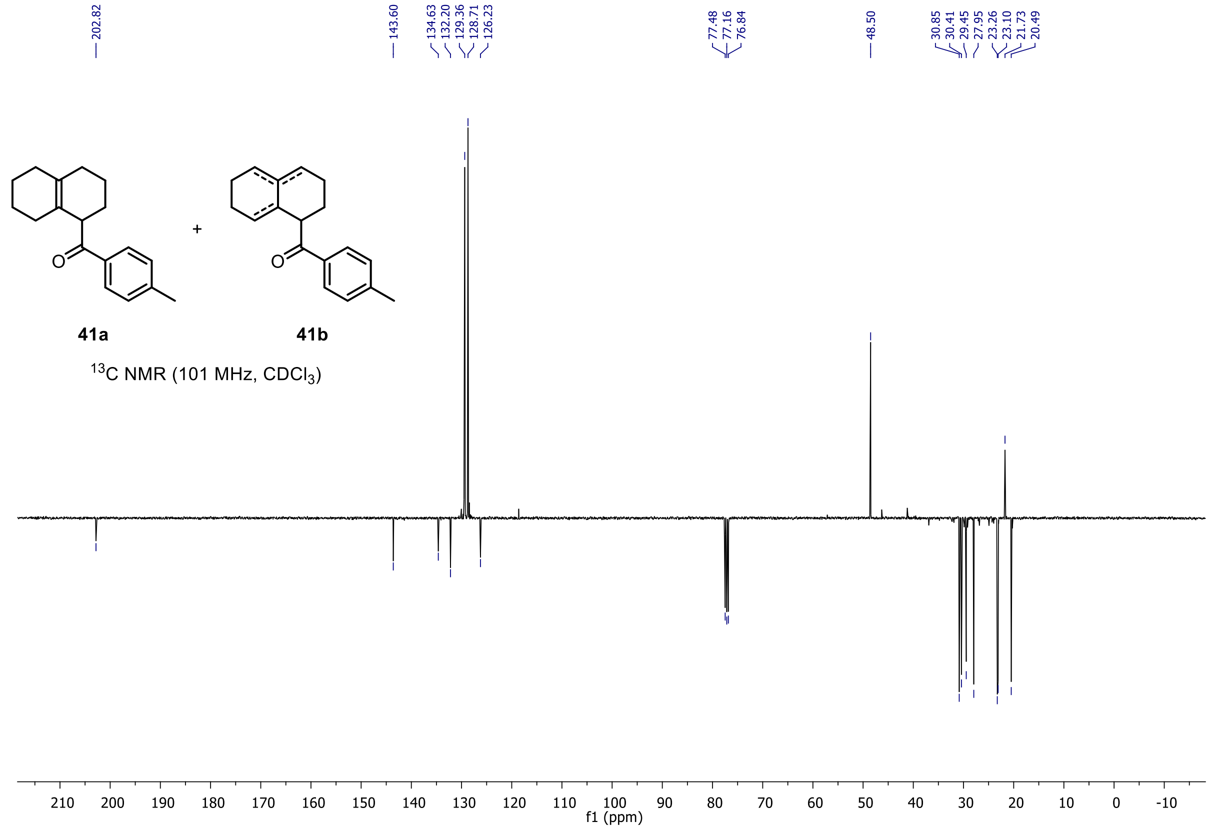
202.82
143.60
134.63
132.20
129.36
128.71
126.23
77.48
77.16
76.84
48.50
30.85
30.41
29.45
27.95
23.26
23.10
21.73
20.49
41a
41b
13C NMR (101 MHz, CDCl3)
210 200 190 180 170 160 150 140 130 120 110 100 90 80 70 60 50 40 30 20 10 0 -10
f1 (ppm)

Saegusa–Ito oxidation (42)

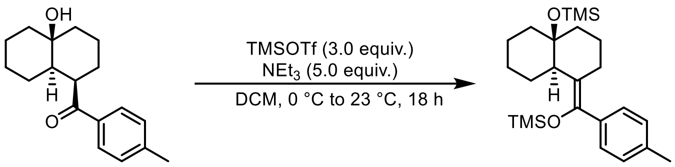

12
(1.0 equiv.)

S33
used without purification

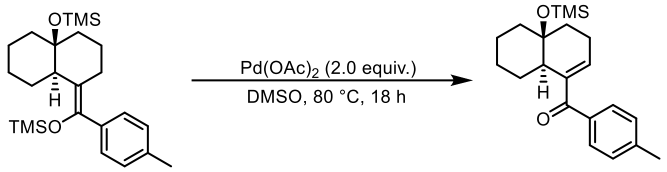

S33
(1.0 equiv.)

42

In a flame-dried Schlenk flask **12** (27.2 mg, 0.10 mmol, 1.00 equiv.) was dissolved in DCM (1.0 mL) and the solution was cooled to 0 °C. TMSOTf (55 µL, 0.30 mmol, 3.00 equiv.) followed by NEt_3 (70 µL, 0.50 mmol, 5.00 equiv.) were added at 0 °C, and the resulting solution was stirred for 18 h with slow warming to ambient temperature (23 °C). The mixture was diluted with Et_2O (2 mL) and the excess reagents were quenched by the addition of aqueous saturated $NaHCO_3$ solution (5 mL). The biphasic mixture was extracted with Et_2O (3 × 5 mL), the combined organic layers were washed with brine (5 mL), dried over anhydrous sodium sulfate, the dried solution was filtered, and the filtrate was concentrated under reduced pressure. The crude product **S33** was used in the next step without further purification.

Crude **S33** (1.00 equiv.) was dissolved in DMSO (1.0 mL) in a Schlenk flask under air at ambient temperature (23 °C). $Pd(OAc)_2$ (45 mg, 0.20 mmol, 2.00 equiv.) was added in one portion and the resulting suspension was heated to 80 °C for 18 h (formation of palladium mirror observed). The mixture was diluted with Et_2O (10 mL), washed with H_2O (2 × 5 mL) and then brine (5 mL). The organic layer was dried over anhydrous sodium sulfate, the dried solution was filtered, and the filtrate was concentrated under reduced pressure. Purification by flash column chromatography (0 – 7% EtOAc in heptanes) afforded **42** as a colorless oil (22.1 mg, 64% over 2 steps).

R_f = 0.31 in 4% EtOAc/heptanes

^{1}H NMR (400 MHz, $CDCl_3$) δ 7.76 (d, *J* = 8.2 Hz, 2H), 7.21 (d, *J* = 7.9 Hz, 2H), 6.00 – 5.93 (m, 1H), 2.48 – 2.37 (m, 5H), 2.20 – 2.09 (m, 1H), 1.82 – 1.65 (m, 5H), 1.63 – 1.49 (m, 2H), 1.45 – 1.29 (m, 3H), 0.21 (s, 9H).

^{13}C NMR (101 MHz, $CDCl_3$) δ 199.0 (C), 143.3 (C), 141.0 (C), 136.2 (C), 134.2 (CH), 130.4 (2CH), 128.9 (2CH), 73.5 (C), 45.5 (CH), 39.0 (CH_2), 34.8 (CH_2), 26.4 (CH_2), 23.3 (CH_2), 23.2 (CH_2), 21.9 (CH_2), 21.8 (CH_3), 2.7 ($3CH_3$).

IR (neat) v_{max}: 2927, 2857, 1648, 1259, 1248, 1110, 1054, 1037, 830, 756.

HRMS (ESI^+): exact mass calculated for $[M\text{-}TMSOH]^{\cdot+}$ ($C_{18}H_{20}O$)$^{\cdot}$ requires *m*/*z* 252.1509, found *m*/*z* 252.1499.

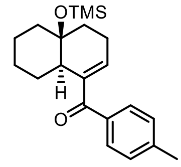

42

^{1}H NMR (400 MHz, $CDCl_3$)

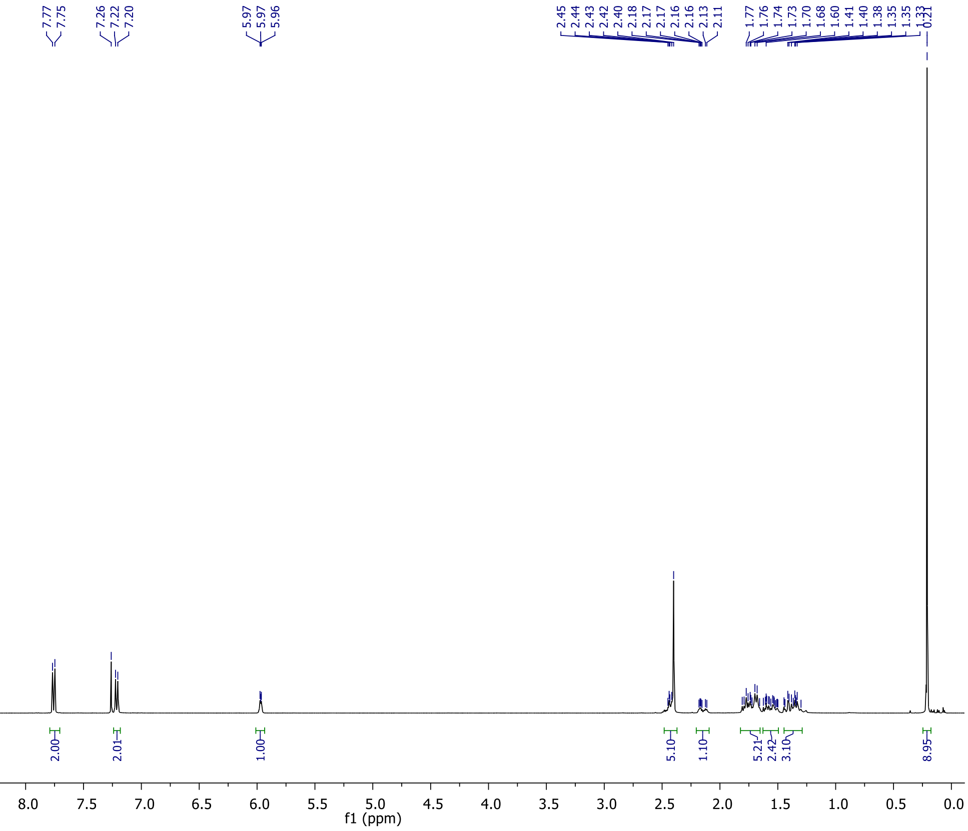

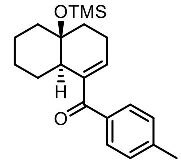

42

^{13}C NMR (101 MHz, $CDCl_3$)

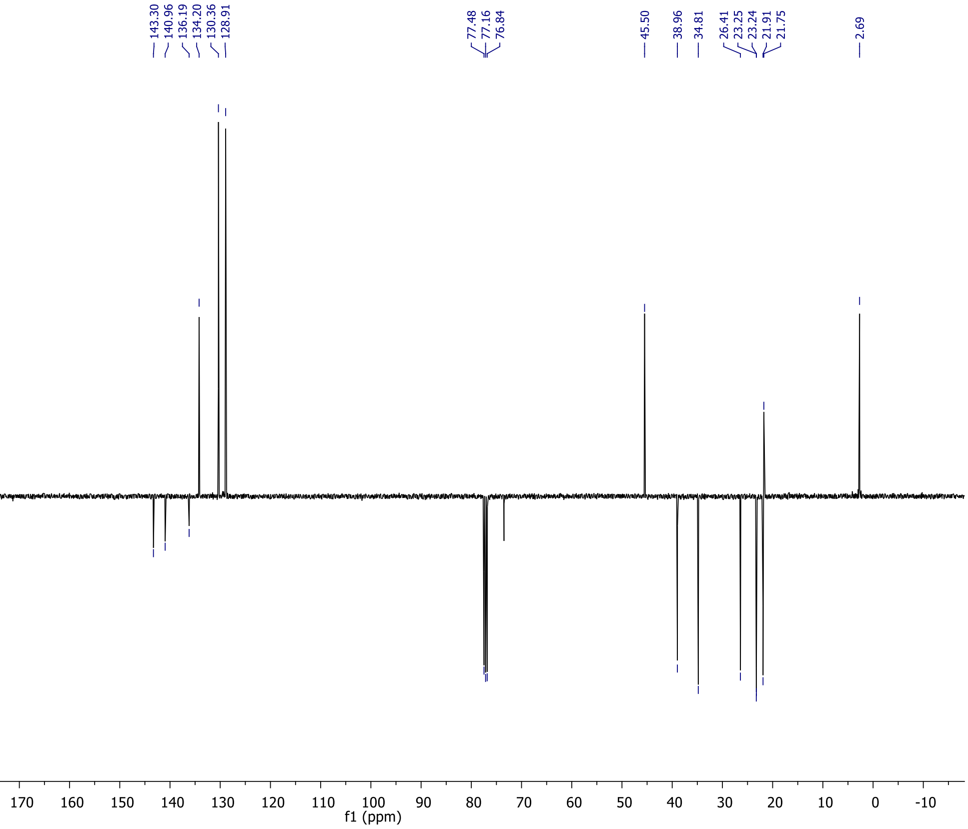

Reduction of 12 with L-selectride (43)

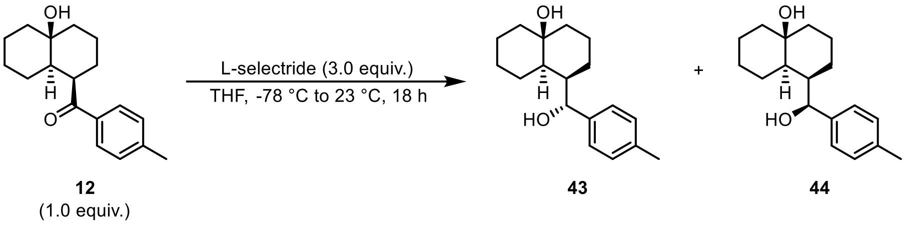

In a small flame-dried vial with a rubber septum cap **12** (13.6 mg, 0.05 mmol, 1.00 equiv.) was dissolved in THF (0.5 mL) and the resulting solution was cooled to -78 °C. L-selectride (1 M in THF, 0.15 mL, 0.15 mmol, 3.00 equiv.) was added under stirring at -78 °C, the mixture was stirred 1 h at -78 °C, then warmed up to ambient temperature (23 °C, cooling bath removed) and stirred for 17 h. The reaction was stopped by the addition of 1 M aqueous NaOH solution (2.5 mL) at ambient temperature (23 °C), followed by the addition of 30% aqueous H_2O_2 (70 µL). After stirring for 30 min, the mixture was extracted with EtOAc (3 × 5 mL), the combined organic layers were washed with aqueous saturated sodium thiosulfate solution (5 mL), dried over anhydrous sodium sulfate, the dried solution was filtered, and the filtrate was concentrated under reduced pressure. *dr* was determined by ^{1}H NMR analysis of the crude reaction product using mesitylene as internal standard (**43**:**44** > 20:1). Purification by flash column chromatography (0 – 25% EtOAc in heptanes) afforded **43** as a colorless crystalline solid (11.1 mg, 81%).

A crystal (**43**) of sufficient quality for X-ray diffractometry was obtained by slow evaporation from $CHCl_3$ (10 mg dissolved in approx. 2.0 mL $CHCl_3$ at 23 °C, vial loosely capped and stored at 23 °C until complete solvent evaporation).

R_f = 0.22 in 20% EtOAc/heptanes

m.p. (+5 °C/min) = 148.1 – 151.3 °C

^{1}H NMR (600 MHz, $CDCl_3$) δ 7.24 (d, *J* = 7.9 Hz, 2H), 7.13 (d, *J* = 7.7 Hz, 2H), 5.04 (s, 1H), 3.06 (br, 2H), 2.33 (s, 3H), 2.13 – 2.03 (m, 1H), 1.95 – 1.87 (m, 2H), 1.79 – 1.66 (m, 4H), 1.64 – 1.54 (m, 3H), 1.51 – 1.45 (m, 1H), 1.43 – 1.31 (m, 4H), 1.13 – 1.05 (m, 1H).

^{13}C NMR (151 MHz, $CDCl_3$) δ 142.0 (C), 136.1 (C), 128.7 (2CH), 126.1 (2CH), 72.7 (CH), 70.2 (C), 46.5 (CH), 44.8 (CH), 41.0 (CH_2), 40.8 (CH_2), 27.3 (CH_2), 26.6 (CH_2), 24.7 (CH_2), 22.0 (CH_2), 21.2 (CH_3), 19.9 (CH_2).

IR (neat) ν_{max}: 3357, 3185, 2920, 2850, 1659, 1632, 1449, 1425, 761, 722.

HRMS (EI$^+$): exact mass calculated for $[M-H_2O]^{\cdot+}$ $(C_{18}H_{24}O)^{\cdot}$ requires *m*/*z* 256.1822, found *m*/*z* 256.1816.

SC-XRD: See Section **5**.

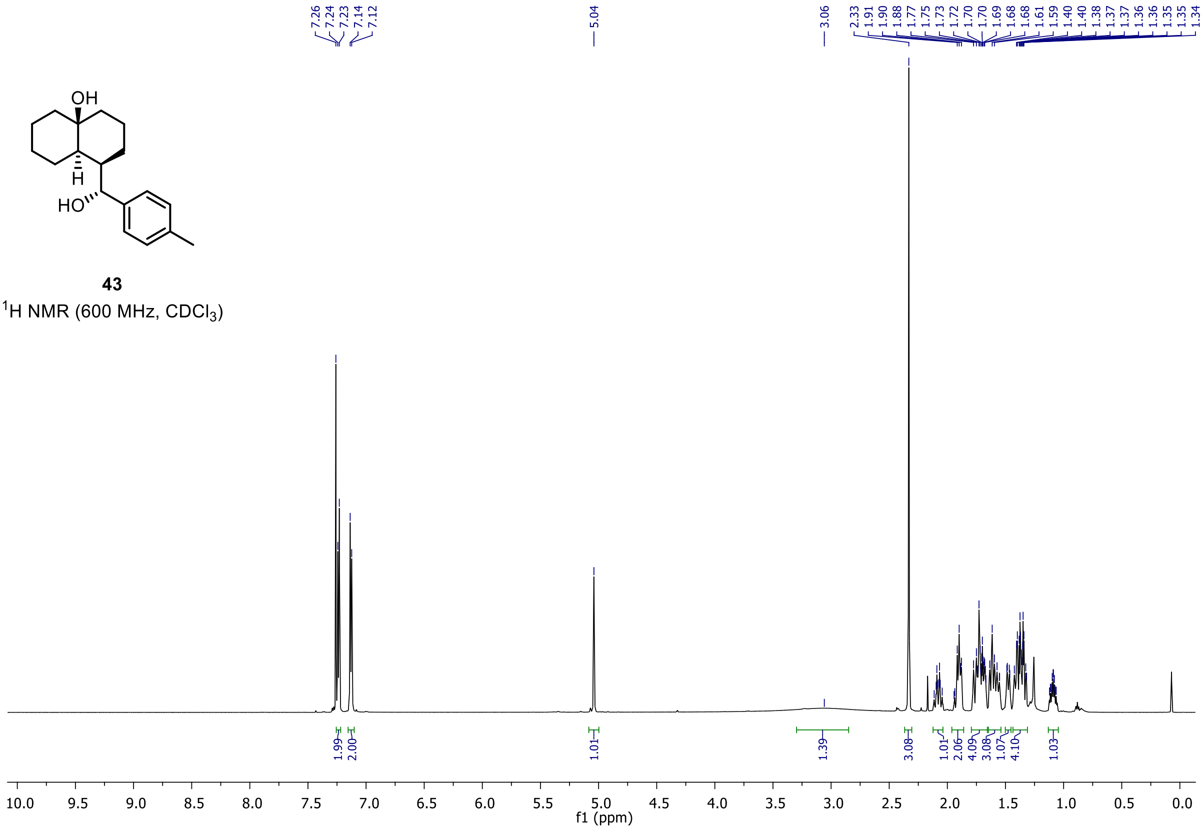

43

^{1}H NMR (600 MHz, $CDCl_3$)

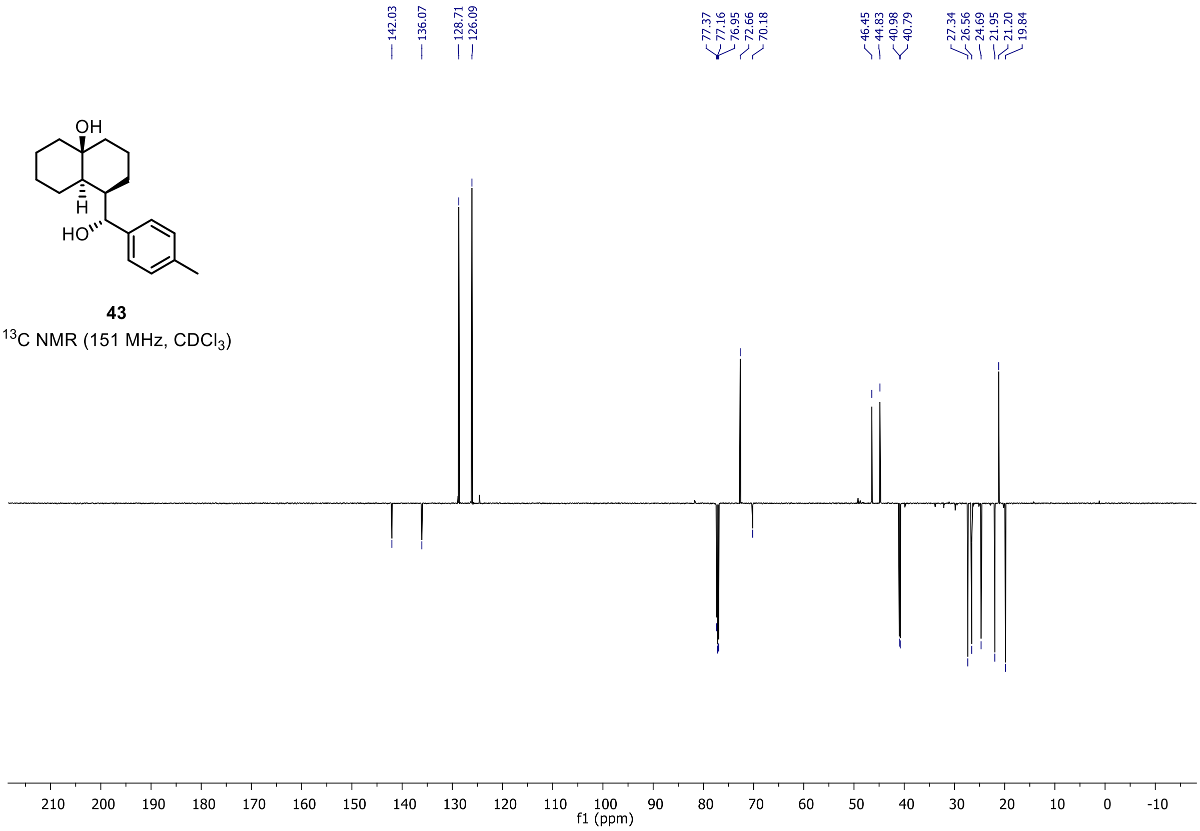

43

^{13}C NMR (151 MHz, $CDCl_3$)

Reduction of 12 with DIBAL-H (44)

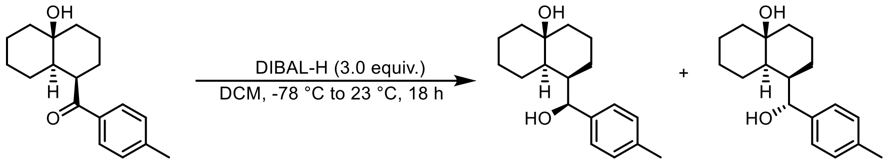

12 (1.0 equiv.) **44** **43**

In a small flame-dried vial with a rubber septum cap, **12** (13.6 mg, 0.05 mmol, 1.00 equiv.) was dissolved in DCM (0.5 mL) and the resulting solution was cooled to -78 °C. DIBAL-H (1 M in DCM, 0.15 mL, 0.15 mmol, 3.00 equiv.) was added under stirring at -78 °C, and the mixture was stirred overnight with slow warming to ambient temperature (23 °C). The resulting solution was diluted with EtOAc (2 mL) and then poured into aqueous saturated sodium bicarbonate solution (5 mL). The biphasic mixture was extracted with EtOAc (3 × 5 mL), the combined organic layers were washed with brine (5 mL), dried over anhydrous sodium sulfate, the dried solution was filtered, and the filtrate was concentrated under reduced pressure. *dr* was determined by ^{1}H NMR analysis of the crude reaction product using mesitylene as internal standard (**44**:**43** = 7:1). Purification by flash column chromatography (0 – 25% EtOAc in heptanes) afforded **44** as a colorless crystalline solid (9.0 mg, 66%) and **43** as a colorless crystalline solid (1.7 mg, 12%).

A crystal (**44**) of sufficient quality for X-ray diffractometry was obtained by slow evaporation of a solution in EtOAc/heptanes (9.0 mg dissolved in approx. 2.0 mL 1:3 EtOAc/heptanes at 23 °C, vial loosely capped and stored at 23 °C until complete solvent evaporation).

Analytical data of **43** were identical with those reported in Section **4.9** – reduction of **12** with L-selectride.

Data for **44**:

$\mathbf{R_f}$ = 0.30 in 20% EtOAc/heptanes

m.p. (+5 °C/min) = 178.7 – 181.2 °C

^{1}H NMR (600 MHz, $CDCl_3$) δ 7.26 (d, *J* = 7.7 Hz, 2H), 7.12 (d, *J* = 7.8 Hz, 2H), 5.16 (d, *J* = 7.0 Hz, 1H), 2.79 (br, 1H), 2.33 (s, 3H), 2.11 – 2.05 (m, 1H), 2.03 – 1.95 (m, 1H), 1.90 – 1.82 (m, 1H), 1.73 – 1.48 (m, 7H), 1.46 – 1.22 (m, 6H), 1.16 – 1.07 (m, 1H).

^{13}C NMR (151 MHz, $CDCl_3$) δ 143.6 (C), 136.6 (C), 128.9 (2CH), 126.3 (2CH), 77.4 (CH), 71.3 (C), 48.1 (CH), 45.0 (CH), 41.7 (CH_2), 41.3 (CH_2), 31.9 (CH_2), 28.9 (CH_2), 27.9 (CH_2), 22.1 (CH_2), 21.2 (CH_3), 18.3 (CH_2).

IR (neat) $\mathbf{v_{max}}$: 3357, 3190, 2923, 2852, 1660, 1632, 1467, 1447, 1411, 769.

HRMS (EI$^+$): exact mass calculated for $[M\text{-}H_2O]^{\cdot+}$ $(C_{18}H_{24}O)^{\cdot}$ requires *m*/*z* 256.1822, found *m*/*z* 256.1819.

SC-XRD: See Section **5**.

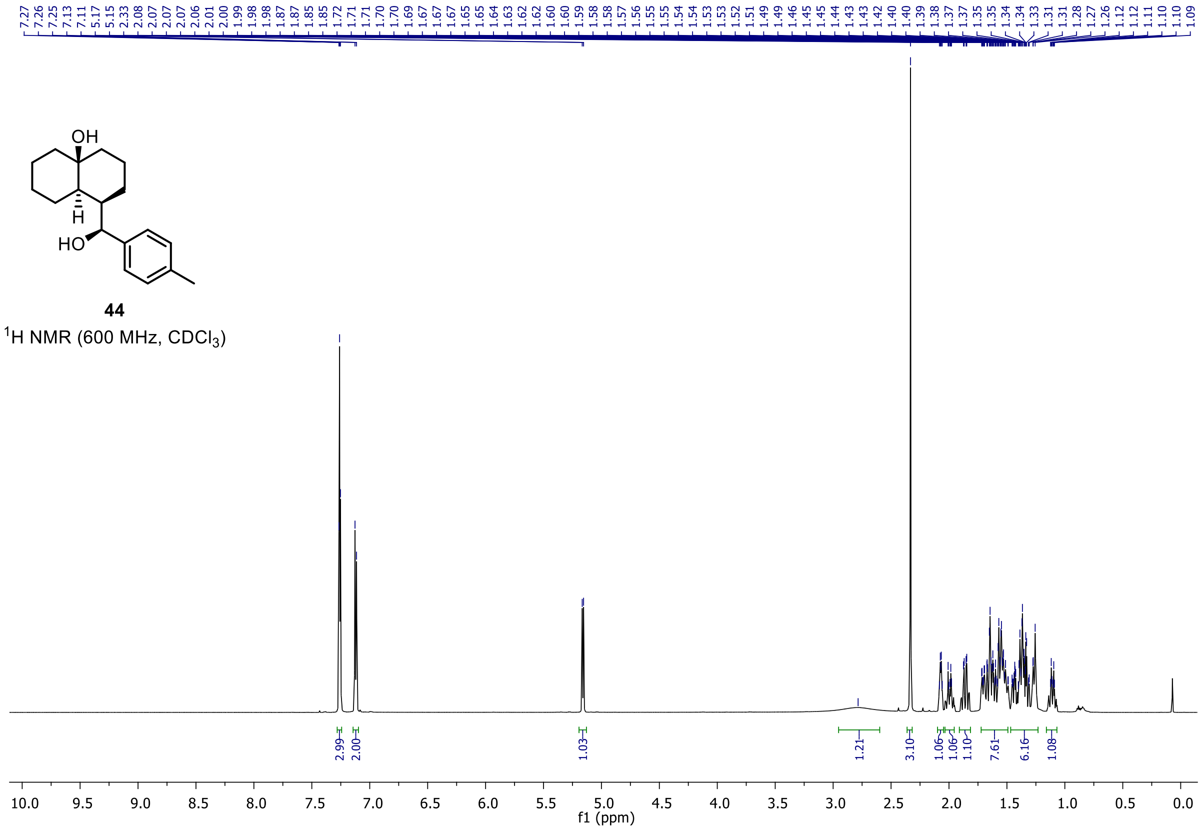
7.27 7.26 7.25 7.13 7.11 5.17 5.15 2.33 2.08 2.07 2.07 2.06 2.01 2.00 1.99 1.98 1.98 1.87 1.87 1.85 1.72 1.71 1.71 1.70 1.69 1.67 1.67 1.67 1.65 1.65 1.64 1.63 1.62 1.62 1.60 1.59 1.58 1.58 1.57 1.56 1.55 1.55 1.54 1.54 1.53 1.53 1.52 1.51 1.49 1.46 1.45 1.45 1.44 1.43 1.43 1.42 1.40 1.40 1.39 1.38 1.37 1.37 1.35 1.35 1.34 1.34 1.33 1.31 1.31 1.28 1.27 1.26 1.12 1.12 1.11 1.10 1.10 1.09
OH
H
HO
44
1H NMR (600 MHz, CDCl3)
2.99 2.00 1.03 1.21 3.10 1.06 1.06 1.10 7.61 6.16 1.08
10.0 9.5 9.0 8.5 8.0 7.5 7.0 6.5 6.0 5.5 5.0 4.5 4.0 3.5 3.0 2.5 2.0 1.5 1.0 0.5 0.0
f1 (ppm)

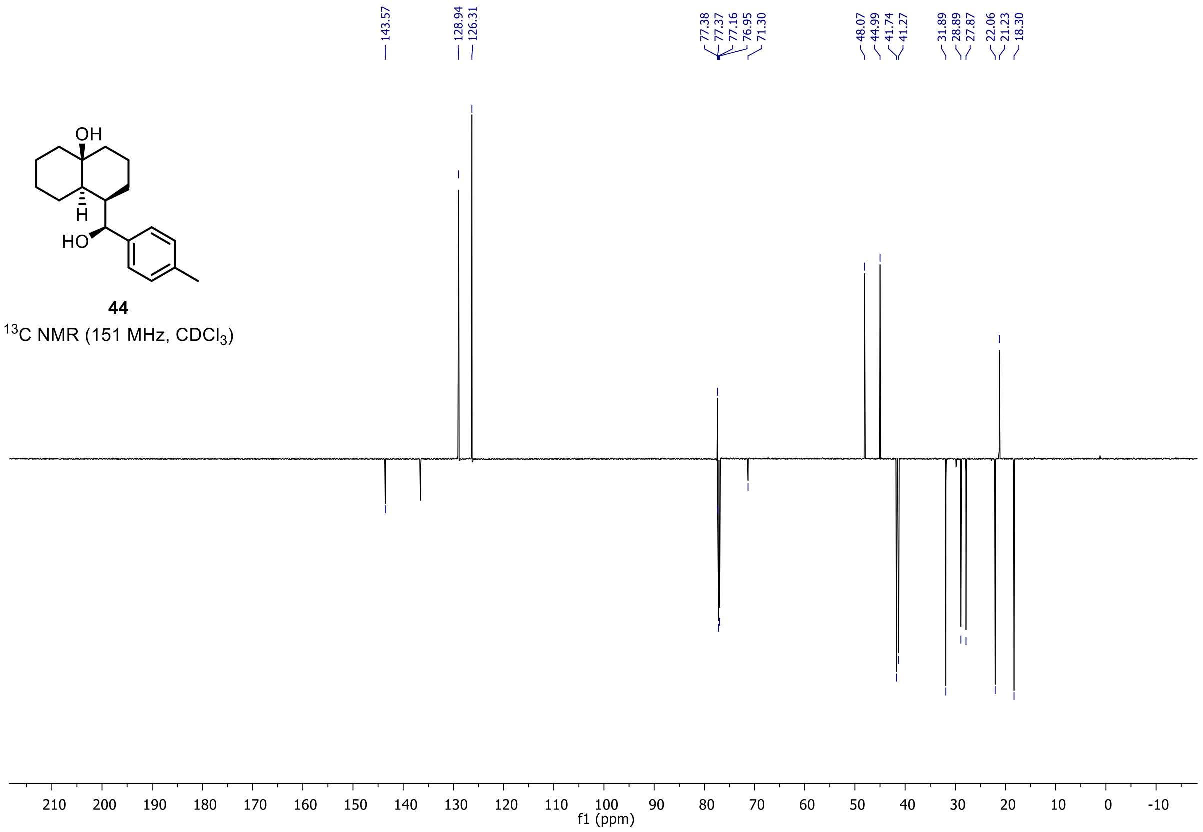
143.57 128.94 126.31 77.38 77.37 77.16 76.95 71.30 48.07 44.99 41.74 41.27 31.89 28.89 27.87 22.06 21.23 18.30
OH
H
HO
44
13C NMR (151 MHz, CDCl3)
210 200 190 180 170 160 150 140 130 120 110 100 90 80 70 60 50 40 30 20 10 0 -10
f1 (ppm)

5. X-ray Analysis

Single crystal X-ray diffraction data were collected with a Stadivari Diffractometer (STOE & Cie GmbH, Germany) equipped with an EIGER2 R500 detector (Dectris Ltd, Switzerland). Data were processed and scaled with the STOE software suite X-Area (STOE & Cie GmbH). Structures were solved with SHELXT[42] and refined with SHELXL[43] or Olex2[44]. Model building was done with Olex2 or ShelXle[45]. The structures were validated with CHECKCIF (https://checkcif.iucr.org/). See the respective CIF file for exact versions and more details.

Experimental data available online: https://www.ccdc.cam.ac.uk/structures/

For compounds **40**, **43** and **44** (three diastereomers of the same compound): The inclusion of asterisks (*) within stereochemical descriptors was used to denote relative stereochemistry, as opposed to absolute stereochemistry. Thus, a compound denoted as (R*,R*) is racemic, with either (R,R)- or (S,S)-configuration.

(4-hydroxydecahydronaphthalen-1-yl)(p-tolyl)methanone (12)

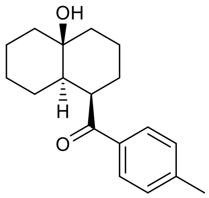

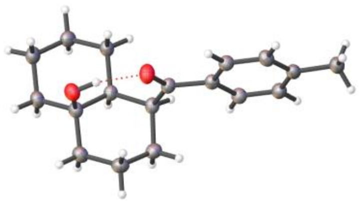

CCDC2339943

Identification code	MIVA-350_a	
Empirical formula	C_{18} H_{24} O_2	
Formula weight	272.38	
Temperature	100 K	
Wavelength	1.54186 Å	
Crystal system	Orthorhombic	
Space group	$Pna2_1$	
Unit cell dimensions	a = 32.5479(12) Å	a= 90°.
	b = 5.8761(2) Å	b= 90°.
	c = 7.7470(2) Å	g = 90°.
Volume	1481.64(9) $Å^3$	
Z	4	
Density (calculated)	1.221 Mg/m^3	
Absorption coefficient	0.605 mm^{-1}	
F(000)	592	
Crystal size	0.180 x 0.080 x 0.030 mm^3	
Theta range for data collection	2.72 to 68.43°.	
Index ranges	-39<=h<=28, -7<=k<=4, -9<=l<=6	
Reflections collected	16828	
Independent reflections	2293 [R(int) = 0.0473]	
Completeness to theta = 67.679°	100.0 %	
Absorption correction	Semi-empirical from equivalents	
Max. and min. transmission	0.8020 and 0.5324	
Refinement method	Full-matrix least-squares on F^2	
Data / restraints / parameters	2293 / 1 / 184	
Goodness-of-fit on F^2	1.083	
Final R indices [I>2sigma(I)]	R1 = 0.0367, wR2 = 0.0891	
R indices (all data)	R1 = 0.0432, wR2 = 0.0932	
Absolute structure parameter	0.38(14)	
Extinction coefficient	n/a	
Largest diff. peak and hole	0.169 and -0.199 e.$Å^{-3}$	

checkCIF/PLATON report

Structure factors have been supplied for datablock(s) MIVA-350_a

THIS REPORT IS FOR GUIDANCE ONLY. IF USED AS PART OF A REVIEW PROCEDURE FOR PUBLICATION, IT SHOULD NOT REPLACE THE EXPERTISE OF AN EXPERIENCED CRYSTALLOGRAPHIC REFEREE.

No syntax errors found. CIF dictionary Interpreting this report

Datablock: MIVA-350_a

```
Bond precision:   C-C = 0.0030 A          Wavelength=1.54186

Cell:              a=32.5479(12)       b=5.8761(2)       c=7.7470(2)
                   alpha=90            beta=90           gamma=90
Temperature:       100 K

                Calculated                  Reported
Volume          1481.65(8)                  1481.64(9)
Space group     P n a 21                    P n a 21
Hall group      P 2c -2n                    P 2c -2n
Moiety formula  C18 H24 O2                  ?
Sum formula     C18 H24 O2                  C18 H24 O2
Mr              272.37                      272.38
Dx,g cm-3       1.221                       1.221
Z               4                           4
Mu (mm-1)       0.605                       0.605
F000            592.0                       592.0
F000'           593.63
h,k,lmax        39,7,9                      7,9,39
Nref            2715[ 1466]                 2293
Tmin,Tmax       0.944,0.982                 0.532,0.802
Tmin'           0.897

Correction method= # Reported T Limits: Tmin=0.532 Tmax=0.802
AbsCorr = MULTI-SCAN

Data completeness= 1.56/0.84        Theta(max)= 68.430

R(reflections)= 0.0367( 2044)
                                                    wR2(reflectio
ns)=
                                                0.0932( 2293)
S = 1.083                    Npar= 184
```

The following ALERTS were generated. Each ALERT has the format
test-name_ALERT_alert-type_alert-level.
Click on the hyperlinks for more details of the test.

Alert level C

```
STRVA01_ALERT_4_C           Flack test results are ambiguous.
           From the CIF: _refine_ls_abs_structure_Flack     0.380
           From the CIF: _refine_ls_abs_structure_Flack_su     0.140
PLAT918_ALERT_3_C Reflection(s) with I(obs) much Smaller I(calc) .          1
Check
```

Alert level G

```
PLAT792_ALERT_1_G Model has Chirality at C8          (Polar  SPGR)          S
Verify PLAT792_ALERT_1_G Model has Chirality at C9   (Polar  SPGR)          S
Verify PLAT792_ALERT_1_G Model has Chirality at C10  (Polar  SPGR)          R
Verify PLAT912_ALERT_4_G Missing # of FCF Reflections Above STh/L=  0.600   6
Note PLAT915_ALERT_3_G No Flack x Check Done: Low Friedel Pair Coverage    67
% PLAT978_ALERT_2_G Number C-C Bonds with Positive Residual Density.        0
Info
```

0 **ALERT level A** = Most likely a serious problem - resolve or explain
0 **ALERT level B** = A potentially serious problem, consider carefully
2 **ALERT level C** = Check. Ensure it is not caused by an omission or oversight
6 **ALERT level G** = General information/check it is not something unexpected

3 ALERT type 1 CIF construction/syntax error, inconsistent or missing data
1 ALERT type 2 Indicator that the structure model may be wrong or deficient
2 ALERT type 3 Indicator that the structure quality may be low
2 ALERT type 4 Improvement, methodology, query or suggestion
0 ALERT type 5 Informative message, check

It is advisable to attempt to resolve as many as possible of the alerts in all categories. Often the minor alerts point to easily fixed oversights, errors and omissions in your CIF or refinement strategy, so attention to these fine details can be worthwhile. In order to resolve some of the more serious problems it may be necessary to carry out additional measurements or structure refinements. However, the purpose of your study may justify the reported deviations and the more serious of these should normally be commented upon in the discussion or experimental section of a paper or in the "special_details" fields of the CIF. checkCIF was carefully designed to identify outliers and unusual parameters, but every test has its limitations and alerts that are not important in a particular case may appear. Conversely, the absence of alerts does not guarantee there are no aspects of the results needing attention. It is up to the individual to critically assess their own results and, if necessary, seek expert advice.

Publication of your CIF in IUCr journals

A basic structural check has been run on your CIF. These basic checks will be run on all CIFs submitted for publication in IUCr journals (*Acta Crystallographica*, *Journal of Applied Crystallography*, *Journal of Synchrotron Radiation*); however, if you intend to submit to *Acta Crystallographica Section C* or *E* or *IUCrData*, you should make sure that full publication checks are run on the final version of your CIF prior to submission.

Publication of your CIF in other journals

Please refer to the *Notes for Authors* of the relevant journal for any special instructions relating to CIF submission.

PLATON version of 28/11/2022; check.def file version of 28/11/2022

Datablock MIVA-350_a - ellipsoid plot

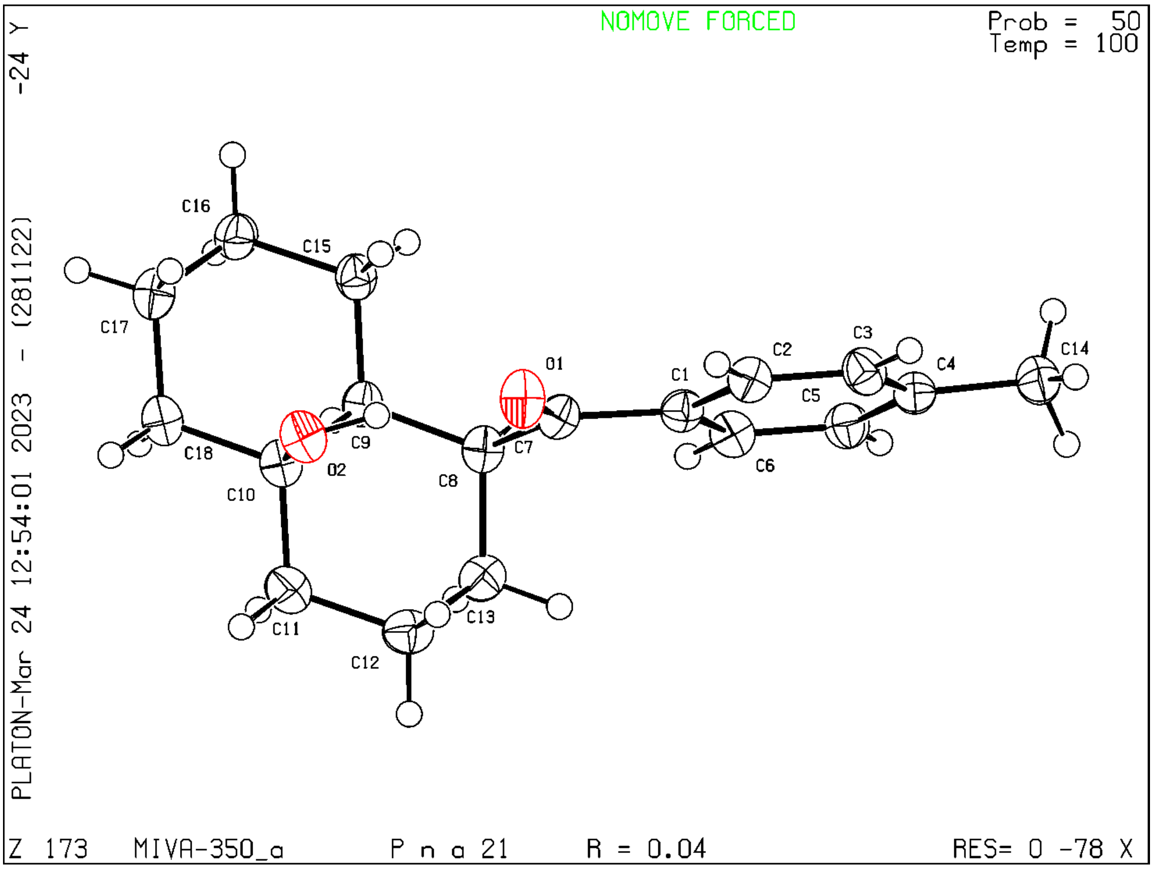

(4-hydroxydecahydronaphthalen-1-yl)(naphthalen-1-yl)methanone (19)

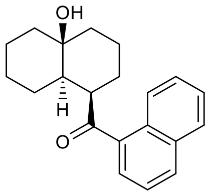

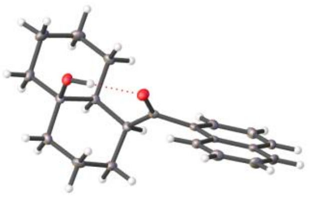

CCDC2339945

Identification code	RAMH-8_a	
Empirical formula	C21 H24 O2	
Formula weight	308.41	
Temperature	100 K	
Wavelength	0.71073 Å	
Crystal system	Triclinic	
Space group	$P2_1/n$	
Unit cell dimensions	a = 13.7550(6) Å	a= 90.000(4)°.
	b = 8.9439(4) Å	b= 105.840(4)°.
	c = 13.8361(7) Å	g = 90.000(4)°.
Volume	1637.53(13) $Å^3$	
Z	4	
Density (calculated)	1.251 Mg/m^3	
Absorption coefficient	0.079 mm^{-1}	
F(000)	664	
Crystal size	0.250 x 0.180 x 0.110 mm^3	
Theta range for data collection	2.93 to 30.92°.	
Index ranges	-19<=h<=19, -12<=k<=12, -19<=l<=19	
Reflections collected	58061	
Independent reflections	5040 [R(int) = 0.0831]	
Completeness to theta = 25.242°	99.9 %	
Absorption correction	Semi-empirical from equivalents	
Max. and min. transmission	0.9967 and 0.6106	
Refinement method	Full-matrix least-squares on F^2	
Data / restraints / parameters	5040 / 0 / 210	
Goodness-of-fit on F^2	0.946	
Final R indices [I>2sigma(I)]	R1 = 0.0381, wR2 = 0.0778	
R indices (all data)	R1 = 0.0674, wR2 = 0.0798	
Extinction coefficient	n/a	
Largest diff. peak and hole	0.348 and -0.220 e.$Å^{-3}$	

checkCIF/PLATON report

Structure factors have been supplied for datablock(s) RAMH-8_a

THIS REPORT IS FOR GUIDANCE ONLY. IF USED AS PART OF A REVIEW PROCEDURE FOR PUBLICATION, IT SHOULD NOT REPLACE THE EXPERTISE OF AN EXPERIENCED CRYSTALLOGRAPHIC REFEREE.

No syntax errors found. CIF dictionary Interpreting this report

Datablock: RAMH-8_a

```
Bond precision:   C-C = 0.0014 A          Wavelength=0.71073

Cell:              a=13.7550(6)     b=8.9439(4)          c=13.8361(7)
                   alpha=90         beta=105.840(4)      gamma=90
Temperature:       100 K

                 Calculated                   Reported
Volume           1637.53(14)                  1637.53(13)
Space group      P 21/n                       P 21/n
Hall group       -P 2yn                       -P 2yn
Moiety formula   C21 H24 O2                   ?
Sum formula      C21 H24 O2                   C21 H24 O2
Mr               308.40                       308.41
Dx,g cm-3        1.251                        1.251
Z                4                            4
Mu (mm-1)        0.079                        0.079
F000             664.0                        664.0
F000'            664.29
h,k,lmax         19,12,20                     12,19,19
Nref             5173                         5040
Tmin,Tmax        0.983,0.991                  0.611,0.997
Tmin'            0.980

Correction method= # Reported T Limits: Tmin=0.611 Tmax=0.997
AbsCorr = MULTI-SCAN

Data completeness= 0.974           Theta(max)= 30.920

R(reflections)= 0.0381( 2959)
                                                  wR2(reflectio
ns)=
                                              0.0798( 5040)
S = 0.946                  Npar= 210
```

The following ALERTS were generated. Each ALERT has the format
test-name_ALERT_alert-type_alert-level.
Click on the hyperlinks for more details of the test.

Alert level C

CRYSC01_ALERT_1_C No recognised color has been given for crystal color.
PLAT147_ALERT_1_C s.u. on Symmetry Constrained Cell Angle(s) .....
Please Check PLAT230_ALERT_2_C
Hirshfeld Test Diff for C11 --C12 .
5.6 s.u.

Alert level G

PLAT793_ALERT_4_G Model has Chirality at C12 (Centro SPGR) S Verify
PLAT793_ALERT_4_G Model has Chirality at C13 (Centro SPGR) S Verify
PLAT793_ALERT_4_G Model has Chirality at C14 (Centro SPGR) R Verify
PLAT910_ALERT_3_G Missing # of FCF Reflection(s) Below Theta(Min). 4 Note
PLAT912_ALERT_4_G Missing # of FCF Reflections Above STh/L= 0.600 129 Note
PLAT978_ALERT_2_G Number C-C Bonds with Positive Residual Density. 19
Info

0 **ALERT level A** = Most likely a serious problem - resolve or explain
0 **ALERT level B** = A potentially serious problem, consider carefully
3 **ALERT level C** = Check. Ensure it is not caused by an omission or oversight
6 **ALERT level G** = General information/check it is not something unexpected

2 ALERT type 1 CIF construction/syntax error, inconsistent or missing data
2 ALERT type 2 Indicator that the structure model may be wrong or deficient
1 ALERT type 3 Indicator that the structure quality may be low
4 ALERT type 4 Improvement, methodology, query or suggestion
0 ALERT type 5 Informative message, check

It is advisable to attempt to resolve as many as possible of the alerts in all categories. Often the minor alerts point to easily fixed oversights, errors and omissions in your CIF or refinement strategy, so attention to these fine details can be worthwhile. In order to resolve some of the more serious problems it may be necessary to carry out additional measurements or structure refinements. However, the purpose of your study may justify the reported deviations and the more serious of these should normally be commented upon in the discussion or experimental section of a paper or in the "special_details" fields of the CIF. checkCIF was carefully designed to identify outliers and unusual parameters, but every test has its limitations and alerts that are not important in a particular case may appear. Conversely, the absence of alerts does not guarantee there are no aspects of the results needing attention. It is up to the individual to critically assess their own results and, if necessary, seek expert advice.

Publication of your CIF in IUCr journals

A basic structural check has been run on your CIF. These basic checks will be run on all CIFs submitted for publication in IUCr journals (*Acta Crystallographica*, *Journal of Applied Crystallography*, *Journal of Synchrotron Radiation*); however, if you intend to submit to *Acta Crystallographica Section C* or *E* or *IUCrData*, you should make sure that full publication checks are run on the final version of your CIF prior to submission.

Publication of your CIF in other journals

Please refer to the *Notes for Authors* of the relevant journal for any special instructions relating to CIF submission.

PLATON version of 28/11/2022; check.def file version of 28/11/2022

Datablock RAMH-8_a - ellipsoid plot

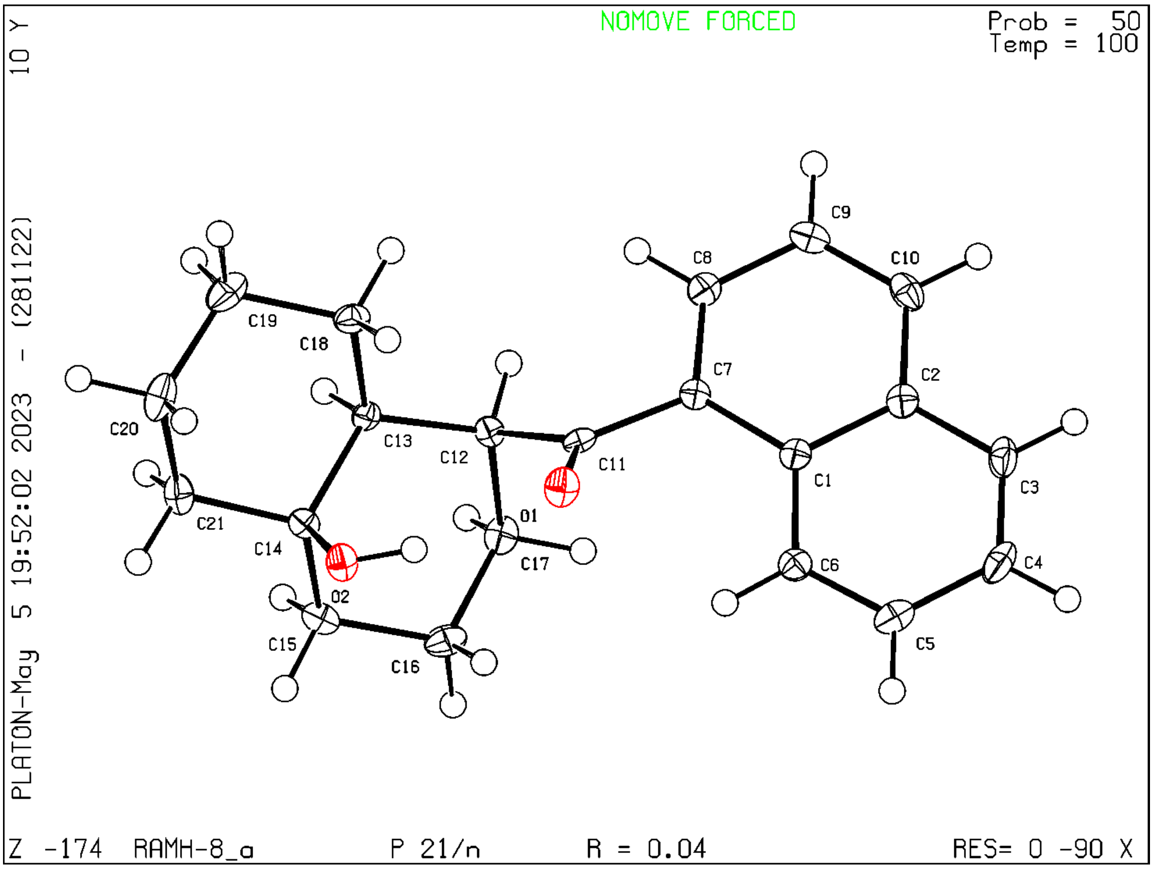

(4-hydroxydecahydronaphthalen-1-yl)(2-iodophenyl)methanone (20)

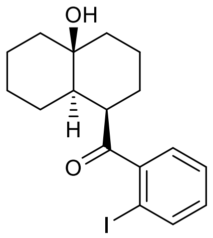

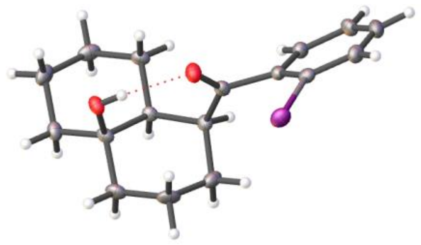

CCDC2339944

Identification code	MIVA432_a	
Empirical formula	C17 H21 I O2	
Formula weight	384.25	
Temperature	100 K	
Wavelength	1.54186 Å	
Crystal system	Monoclinic	
Space group	$P2_1/c$	
Unit cell dimensions	a = 10.7348(3) Å	a= 90°.
	b = 12.5320(3) Å	b= 95.324(2)°.
	c = 11.5682(3) Å	g = 90°.
Volume	1549.54(7) $Å^3$	
Z	4	
Density (calculated)	1.647 Mg/m^3	
Absorption coefficient	16.224 mm^{-1}	
F(000)	768	
Crystal size	0.120 x 0.070 x 0.060 mm^3	
Theta range for data collection	3.53 to 68.49°.	
Index ranges	-12<=h<=12, -13<=k<=15, -13<=l<=9	
Reflections collected	33965	
Independent reflections	2812 [R(int) = 0.0271]	
Completeness to theta = 67.679°	99.4 %	
Absorption correction	Semi-empirical from equivalents	
Max. and min. transmission	0.5976 and 0.3866	
Refinement method	Full-matrix least-squares on F^2	
Data / restraints / parameters	2812 / 0 / 183	
Goodness-of-fit on F^2	1.054	
Final R indices [I>2sigma(I)]	R1 = 0.0229, wR2 = 0.0587	
R indices (all data)	R1 = 0.0277, wR2 = 0.0603	
Extinction coefficient	n/a	
Largest diff. peak and hole	0.293 and -1.016 e.$Å^{-3}$	

checkCIF/PLATON report

Structure factors have been supplied for datablock(s) MIVA432_a

THIS REPORT IS FOR GUIDANCE ONLY. IF USED AS PART OF A REVIEW PROCEDURE FOR PUBLICATION, IT SHOULD NOT REPLACE THE EXPERTISE OF AN EXPERIENCED CRYSTALLOGRAPHIC REFEREE.

No syntax errors found. CIF dictionary Interpreting this report

Datablock: MIVA432_a

```
Bond precision:   C-C = 0.0033 A          Wavelength=1.54186

Cell:             a=10.7348(3)      b=12.5320(3)       c=11.5682(3)
                  alpha=90          beta=95.324(2)     gamma=90
Temperature:      100 K
```

	Calculated	Reported
Volume	1549.54(7)	1549.54(7)
Space group	P 21/c	P 21/c
Hall group	-P 2ybc	-P 2ybc
Moiety formula	C17 H21 I O2	?
Sum formula	C17 H21 I O2	C17 H21 I O2
Mr	384.24	384.25
Dx,g cm-3	1.647	1.647
Z	4	4
Mu (mm-1)	16.224	16.224
F000	768.0	768.0
F000’	768.78	
h,k,lmax	12,15,13	12,15,13
Nref	2842	2812
Tmin,Tmax	0.294,0.378	0.387,0.598
Tmin’	0.124	

```
Correction method= # Reported T Limits: Tmin=0.387 Tmax=0.598
AbsCorr = MULTI-SCAN

Data completeness= 0.989           Theta(max)= 68.490

R(reflections)= 0.0229( 2492)
                                                   wR2(reflectio
ns)=
                                                0.0603( 2812)
S = 1.054                  Npar= 183
```

The following ALERTS were generated. Each ALERT has the format
test-name_ALERT_alert-type_alert-level.
Click on the hyperlinks for more details of the test.

Alert level C

CRYSC01_ALERT_1_C No recognised color has been given for crystal color.
PLAT911_ALERT_3_C Missing FCF Refl Between Thmin & STh/L= 0.600 17 Report

Alert level G

PLAT793_ALERT_4_G Model has Chirality at C8 (Centro SPGR) S Verify
PLAT793_ALERT_4_G Model has Chirality at C9 (Centro SPGR) S Verify
PLAT793_ALERT_4_G Model has Chirality at C10 (Centro SPGR) R Verify
PLAT912_ALERT_4_G Missing # of FCF Reflections Above STh/L= 0.600 14 Note
PLAT978_ALERT_2_G Number C-C Bonds with Positive Residual Density. 7 Info

0 **ALERT level A** = Most likely a serious problem - resolve or explain
0 **ALERT level B** = A potentially serious problem, consider carefully
2 **ALERT level C** = Check. Ensure it is not caused by an omission or oversight
5 **ALERT level G** = General information/check it is not something unexpected

1 ALERT type 1 CIF construction/syntax error, inconsistent or missing data
1 ALERT type 2 Indicator that the structure model may be wrong or deficient
1 ALERT type 3 Indicator that the structure quality may be low
4 ALERT type 4 Improvement, methodology, query or suggestion
0 ALERT type 5 Informative message, check

It is advisable to attempt to resolve as many as possible of the alerts in all categories. Often the minor alerts point to easily fixed oversights, errors and omissions in your CIF or refinement strategy, so attention to these fine details can be worthwhile. In order to resolve some of the more serious problems it may be necessary to carry out additional measurements or structure refinements. However, the purpose of your study may justify the reported deviations and the more serious of these should normally be commented upon in the discussion or experimental section of a paper or in the "special_details" fields of the CIF. checkCIF was carefully designed to identify outliers and unusual parameters, but every test has its limitations and alerts that are not important in a particular case may appear. Conversely, the absence of alerts does not guarantee there are no aspects of the results needing attention. It is up to the individual to critically assess their own results and, if necessary, seek expert advice.

Publication of your CIF in IUCr journals

A basic structural check has been run on your CIF. These basic checks will be run on all CIFs submitted for publication in IUCr journals (*Acta Crystallographica*, *Journal of Applied Crystallography*, *Journal of Synchrotron Radiation*); however, if you intend to submit to *Acta Crystallographica Section C* or *E* or *IUCrData*, you should make sure that full publication checks are run on the final version of your CIF prior to submission.

Publication of your CIF in other journals

Please refer to the *Notes for Authors* of the relevant journal for any special instructions relating to CIF submission.

PLATON version of 28/11/2022; check.def file version of 28/11/2022

Datablock MIVA432_a - ellipsoid plot

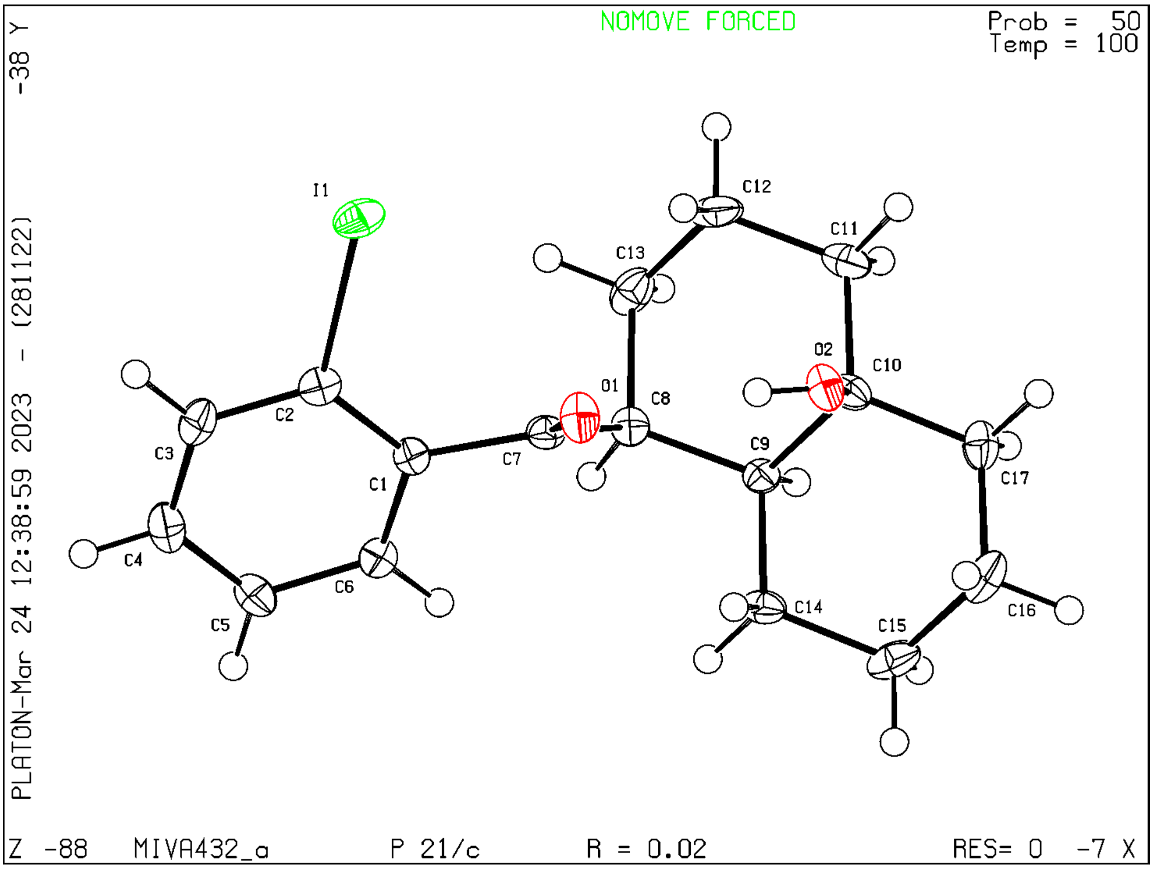

(1S*,4aS*,8aR*)-1-((S*)-hydroxy(p-tolyl)methyl)octahydronaphthalen-4a(2H)-ol (40)

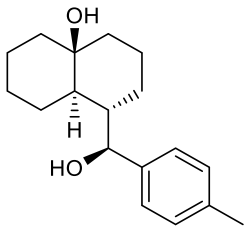

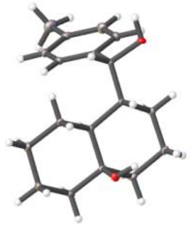

CCDC2339954

Identification code	MIVA611_a	
Empirical formula	C18 H26 O2	
Formula weight	274.39	
Temperature	100(2) K	
Wavelength	0.71073 Å	
Crystal system	Triclinic	
Space group	P-1	
Unit cell dimensions	a = 10.9302(5) Å	a= 81.536(4)°.
	b = 11.0996(6) Å	b= 72.247(4)°.
	c = 13.3785(7) Å	g = 81.769(4)°.
Volume	1520.62(14) $Å^3$	
Z	4	
Density (calculated)	1.199 Mg/m^3	
Absorption coefficient	0.076 mm^{-1}	
F(000)	600	
Crystal size	0.300 x 0.270 x 0.140 mm^3	
Theta range for data collection	2.849 to 35.830°.	
Index ranges	-17<=h<=18, -18<=k<=17, -21<=l<=21	
Reflections collected	98740	
Independent reflections	13719 [R(int) = 0.0844]	
Completeness to theta = 25.242°	99.8 %	
Absorption correction	Semi-empirical from equivalents	
Max. and min. transmission	0.9829 and 0.3215	
Refinement method	Full-matrix least-squares on F^2	
Data / restraints / parameters	13719 / 0 / 371	
Goodness-of-fit on F^2	0.851	
Final R indices [I>2sigma(I)]	R1 = 0.0441, wR2 = 0.0863	
R indices (all data)	R1 = 0.0951, wR2 = 0.0896	
Extinction coefficient	n/a	
Largest diff. peak and hole	0.417 and -0.404 e.$Å^{-3}$	

checkCIF/PLATON report

Structure factors have been supplied for datablock(s) MIVA611_a

THIS REPORT IS FOR GUIDANCE ONLY. IF USED AS PART OF A REVIEW PROCEDURE FOR PUBLICATION, IT SHOULD NOT REPLACE THE EXPERTISE OF AN EXPERIENCED CRYSTALLOGRAPHIC REFEREE.

No syntax errors found.   CIF dictionary   Interpreting this report

Datablock: MIVA611_a

```
Bond precision:   C-C = 0.0014 A          Wavelength=0.71073

Cell:            a=10.9302(5)      b=11.0996(6)      c=13.3785(7)
                 alpha=81.536(4)   beta=72.247(4)    gamma=81.769(4)
Temperature:     100 K

                  Calculated                  Reported
Volume            1520.62(14)                 1520.62(14)
Space group       P -1                        P -1
Hall group        -P 1                        -P 1
Moiety formula    C18 H26 O2                  ?
Sum formula       C18 H26 O2                  C18 H26 O2
Mr                274.39                      274.39
Dx,g cm-3         1.199                       1.199
Z                 4                           4
Mu (mm-1)         0.076                       0.076
F000              600.0                       600.0
F000'             600.25
h,k,lmax          17,18,22                    18,18,21
Nref              14232                       13719
Tmin,Tmax         0.977,0.989                 0.322,0.983
Tmin'             0.977

Correction method= # Reported T Limits: Tmin=0.322 Tmax=0.983
AbsCorr = MULTI-SCAN

Data completeness= 0.964            Theta(max)= 35.830

R(reflections)= 0.0441( 6296)
                                                      wR2(reflectio
ns)=
                                                 0.0896( 13719)
S = 0.851                  Npar= 371
```

The following ALERTS were generated. Each ALERT has the format
test-name_ALERT_alert-type_alert-level.
Click on the hyperlinks for more details of the test.

Alert level C

PLAT026_ALERT_3_C Ratio Observed / Unique Reflections (too) Low .. 46 % Check
PLAT910_ALERT_3_C Missing # of FCF Reflection(s) Below Theta(Min). 8 Note

Alert level G

PLAT154_ALERT_1_G The s.u.'s on the Cell Angles are Equal ..(Note) 0.004 Degree
PLAT380_ALERT_4_G Incorrectly? Oriented X(sp2)-Methyl Moiety ..... C36 Check
PLAT912_ALERT_4_G Missing # of FCF Reflections Above STh/L= 0.600 501 Note
PLAT933_ALERT_2_G Number of HKL-OMIT Records in Embedded .res File 3 Note
PLAT978_ALERT_2_G Number C-C Bonds with Positive Residual Density. 15 Info

0 **ALERT level A** = Most likely a serious problem - resolve or explain
0 **ALERT level B** = A potentially serious problem, consider carefully
2 **ALERT level C** = Check. Ensure it is not caused by an omission or oversight
5 **ALERT level G** = General information/check it is not something unexpected

1 ALERT type 1 CIF construction/syntax error, inconsistent or missing data
2 ALERT type 2 Indicator that the structure model may be wrong or deficient
2 ALERT type 3 Indicator that the structure quality may be low
2 ALERT type 4 Improvement, methodology, query or suggestion
0 ALERT type 5 Informative message, check

It is advisable to attempt to resolve as many as possible of the alerts in all categories. Often the minor alerts point to easily fixed oversights, errors and omissions in your CIF or refinement strategy, so attention to these fine details can be worthwhile. In order to resolve some of the more serious problems it may be necessary to carry out additional measurements or structure refinements. However, the purpose of your study may justify the reported deviations and the more serious of these should normally be commented upon in the discussion or experimental section of a paper or in the "special_details" fields of the CIF. checkCIF was carefully designed to identify outliers and unusual parameters, but every test has its limitations and alerts that are not important in a particular case may appear. Conversely, the absence of alerts does not guarantee there are no aspects of the results needing attention. It is up to the individual to critically assess their own results and, if necessary, seek expert advice.

Publication of your CIF in IUCr journals

A basic structural check has been run on your CIF. These basic checks will be run on all CIFs submitted for publication in IUCr journals (*Acta Crystallographica*, *Journal of Applied Crystallography*, *Journal of Synchrotron Radiation*); however, if you intend to submit to *Acta Crystallographica Section C* or *E* or *IUCrData*, you should make sure that full publication checks are run on the final version of your CIF prior to submission.

Publication of your CIF in other journals

Please refer to the *Notes for Authors* of the relevant journal for any special instructions relating to CIF submission.

PLATON version of 06/07/2023; check.def file version of 30/06/2023

Datablock MIVA611_a - ellipsoid plot

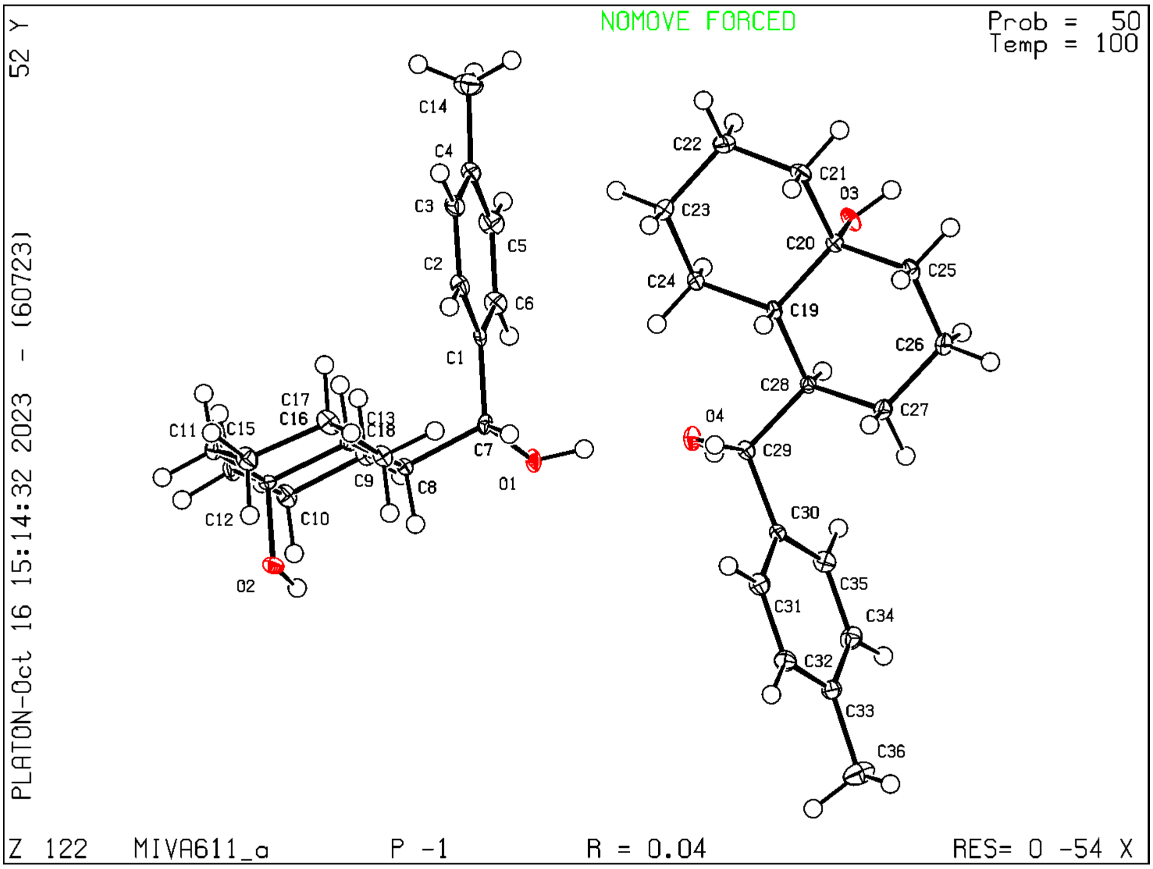

(1R*,4aS*,8aR*)-1-((R*)-hydroxy(p-tolyl)methyl)octahydronaphthalen-4a(2H)-ol (43)

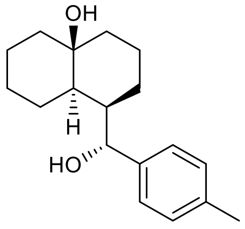

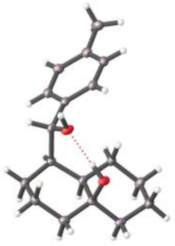

CCDC2339952

Identification code	miva557_f13_15	
Empirical formula	C18 H26 O2	
Formula weight	274.39	
Temperature	100 K	
Wavelength	0.71073 Å	
Crystal system	Orthorhombic	
Space group	$P2_12_12_1$	
Unit cell dimensions	a = 9.1608(4) Å	a= 90°.
	b = 9.6194(5) Å	b= 90°.
	c = 17.0749(12) Å	g = 90°.
Volume	1504.66(15) $Å^3$	
Z	4	
Density (calculated)	1.211 Mg/m^3	
Absorption coefficient	0.077 mm^{-1}	
F(000)	600	
Crystal size	0.28 x 0.277 x 0.27 mm^3	
Theta range for data collection	4.160 to 30.031°.	
Index ranges	-12<=h<=12, -13<=k<=13, -24<=l<=24	
Reflections collected	75858	
Independent reflections	4389 [R(int) = 0.0818]	
Completeness to theta = 25.242°	99.2 %	
Absorption correction	Semi-empirical from equivalents	
Max. and min. transmission	0.9650 and 0.5453	
Refinement method	Full-matrix least-squares on F^2	
Data / restraints / parameters	4389 / 0 / 185	
Goodness-of-fit on F^2	1.021	
Final R indices [I>2sigma(I)]	R1 = 0.0471, wR2 = 0.1241	
R indices (all data)	R1 = 0.0648, wR2 = 0.1388	
Absolute structure parameter	0.0(6)	
Extinction coefficient	0.039(8)	
Largest diff. peak and hole	0.361 and -0.387 e.$Å^{-3}$	

checkCIF/PLATON report

Structure factors have been supplied for datablock(s) miva557_f13_15

THIS REPORT IS FOR GUIDANCE ONLY. IF USED AS PART OF A REVIEW PROCEDURE FOR PUBLICATION, IT SHOULD NOT REPLACE THE EXPERTISE OF AN EXPERIENCED CRYSTALLOGRAPHIC REFEREE.

No syntax errors found. CIF dictionary Interpreting this report

Datablock: miva557_f13_15

```
Bond precision:    C-C = 0.0037 A           Wavelength=0.71073

Cell:               a=9.1608(4)      b=9.6194(5)      c=17.0749(12)
                    alpha=90         beta=90          gamma=90
Temperature:        100 K

                 Calculated                    Reported
Volume           1504.66(15)                   1504.66(15)
Space group      P 21 21 21                    P 21 21 21
Hall group       P 2ac 2ab                     P 2ac 2ab
Moiety formula   C18 H26 O2                    C18 H26 O2
Sum formula      C18 H26 O2                    C18 H26 O2
Mr               274.39                        274.39
Dx,g cm-3        1.211                         1.211
Z                4                             4
Mu (mm-1)        0.077                         0.077
F000             600.0                         600.0
F000'            600.25
h,k,lmax         12,13,24                      12,13,24
Nref             4404[ 2506]                   4389
Tmin,Tmax        0.979,0.979                   0.545,0.965
Tmin'            0.979

Correction method= # Reported T Limits: Tmin=0.545 Tmax=0.965
AbsCorr = MULTI-SCAN

Data completeness= 1.75/1.00        Theta(max)= 30.031

R(reflections)= 0.0471( 3303)
                                                     wR2(reflectio
ns)=
                                                0.1388( 4389)
S = 1.021                    Npar= 185
```

The following ALERTS were generated. Each ALERT has the format
test-name_ALERT_alert-type_alert-level.
Click on the hyperlinks for more details of the test.

Alert level C

STRVA01_ALERT_4_C Flack test results are meaningless. From the CIF: _refine_ls_abs_structure_Flack 0.000
From the CIF: _refine_ls_abs_structure_Flack_su 0.600
PLAT910_ALERT_3_C Missing # of FCF Reflection(s) Below Theta(Min). 8 Note
1 1 0, 1 0 1, 0 1 1, 1 1 1, 0 0 2, 1 0 2,
0 1 2, 1 1 2,
PLAT911_ALERT_3_C Missing FCF Refl Between Thmin & STh/L= 0.600 5 Report
3 5 0, 2 0 1, 0 2 1, 1 0 3, 0 1 4,

Alert level G

PLAT007_ALERT_5_G Number of Unrefined Donor-H Atoms .... 2 Report H1A
H2
PLAT019_ALERT_1_G _diffrn_measured_fraction_theta_full/*_max < 1.0 0.997
Report PLAT032_ALERT_4_G Std. Uncertainty on Flack Parameter Value High . 0.600
Report PLAT850_ALERT_4_G Check Flack Parameter Exact Value 0.00 with s.u. 0.60
Check PLAT933_ALERT_2_G Number of HKL-OMIT Records in Embedded .res File 12
Note
-1 1 1, -1 1 2, 0 1 2, 0 1 4, 0 2 1, 1 0 2,
1 0 3, 1 1 0, 1 1 1, 1 1 2, 2 0 1, 3 5 0,
PLAT969_ALERT_5_G The ’Henn et al.’ R-Factor-gap value .... 5.
46 Note Predicted wR2: Based on SigI**2 2.54 or SHELX Weight 13.90
PLAT978_ALERT_2_G Number C-C Bonds with Positive Residual Density. 8 Info

0 **ALERT level A** = Most likely a serious problem - resolve or explain
0 **ALERT level B** = A potentially serious problem, consider carefully
3 **ALERT level C** = Check. Ensure it is not caused by an omission or oversight
7 **ALERT level G** = General information/check it is not something unexpected

1 ALERT type 1 CIF construction/syntax error, inconsistent or missing data
2 ALERT type 2 Indicator that the structure model may be wrong or deficient
2 ALERT type 3 Indicator that the structure quality may be low
3 ALERT type 4 Improvement, methodology, query or suggestion
2 ALERT type 5 Informative message, check

It is advisable to attempt to resolve as many as possible of the alerts in all categories. Often the minor alerts point to easily fixed oversights, errors and omissions in your CIF or refinement strategy, so attention to these fine details can be worthwhile. In order to resolve some of the more serious problems it may be necessary to carry out additional measurements or structure refinements. However, the purpose of your study may justify the reported deviations and the more serious of these should normally be commented upon in the discussion or experimental section of a paper or in the "special_details" fields of the CIF. checkCIF was carefully designed to identify outliers and unusual parameters, but every test has its limitations and alerts that are not important in a particular case may appear. Conversely, the absence of alerts does not guarantee there are no aspects of the results needing attention. It is up to the individual to critically assess their own results and, if necessary, seek expert advice.

Publication of your CIF in IUCr journals

A basic structural check has been run on your CIF. These basic checks will be run on all CIFs submitted for publication in IUCr journals (*Acta Crystallographica*, *Journal of Applied Crystallography*, *Journal of Synchrotron Radiation*); however, if you intend to submit to *Acta Crystallographica Section C* or *E* or *IUCrData*, you should make sure that full publication checks are run on the final version of your CIF prior to submission.

Publication of your CIF in other journals

Please refer to the *Notes for Authors* of the relevant journal for any special instructions relating to CIF submission.

PLATON version of 06/01/2024; check.def file version of 05/01/2024

Datablock miva557_f13_15 - ellipsoid plot

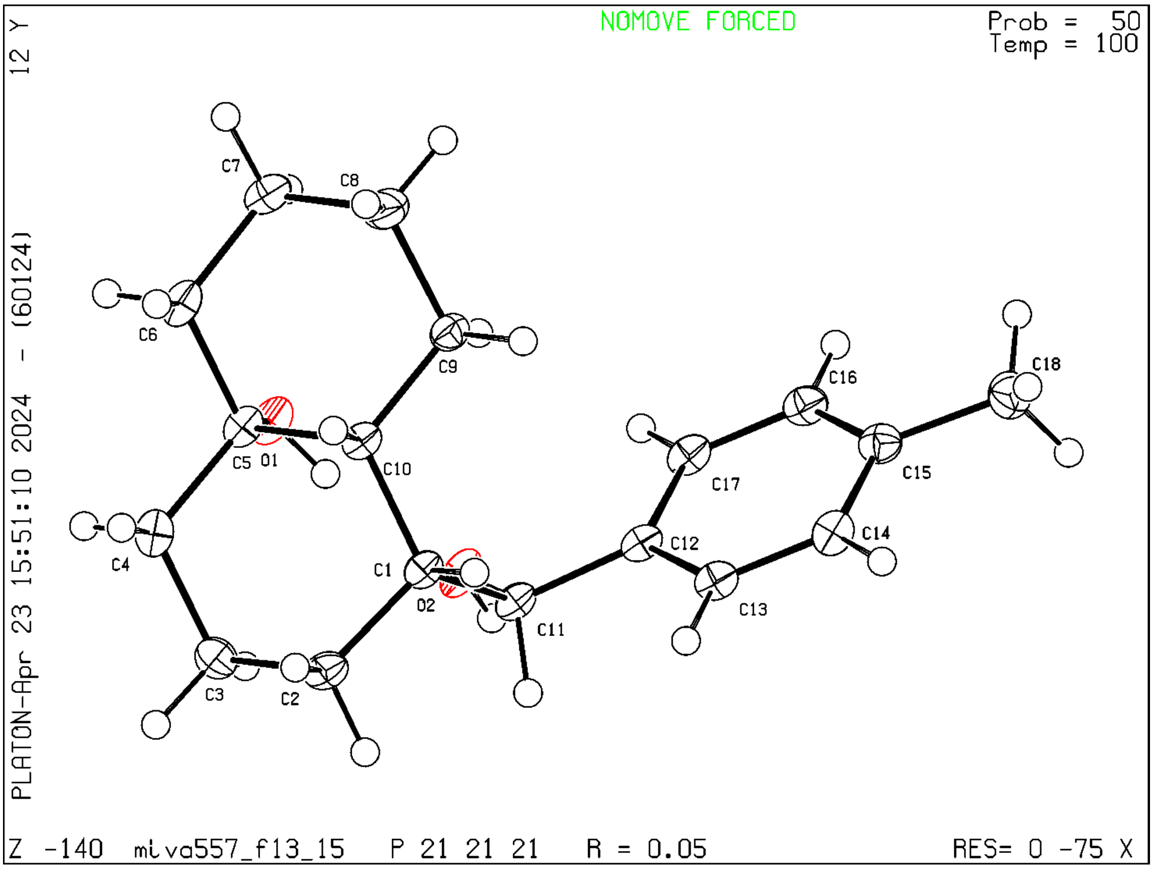

(1R*,4aS*,8aR*)-1-((S*)-hydroxy(p-tolyl)methyl)octahydronaphthalen-4a(2H)-ol (44)

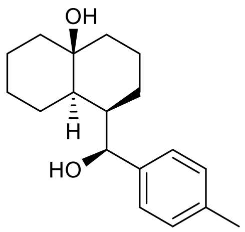

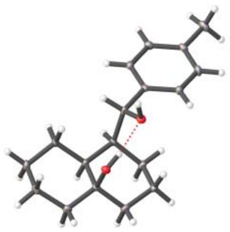

CCDC2339946

Identification code	miva556f1416	
Empirical formula	C18 H26 O2	
Formula weight	274.39	
Temperature	100 K	
Wavelength	0.71073 Å	
Crystal system	Triclinic	
Space group	P-1	
Unit cell dimensions	a = 6.3438(4) Å	a= 72.476(5)°.
	b = 10.9125(7) Å	b= 89.445(5)°.
	c = 11.5912(7) Å	g = 78.015(5)°.
Volume	747.30(8) $Å^3$	
Z	2	
Density (calculated)	1.219 Mg/m^3	
Absorption coefficient	0.077 mm^{-1}	
F(000)	300	
Crystal size	0.27 x 0.13 x 0.04 mm^3	
Theta range for data collection	3.112 to 35.852°.	
Index ranges	-10<=h<=10, -17<=k<=17, -16<=l<=18	
Reflections collected	35169	
Independent reflections	6600 [R(int) = 0.0460]	
Completeness to theta = 25.242°	99.9 %	
Absorption correction	Semi-empirical from equivalents	
Max. and min. transmission	0.9728 and 0.8726	
Refinement method	Full-matrix least-squares on F^2	
Data / restraints / parameters	6600 / 0 / 184	
Goodness-of-fit on F^2	0.930	
Final R indices [I>2sigma(I)]	R1 = 0.0411, wR2 = 0.0914	
R indices (all data)	R1 = 0.0922, wR2 = 0.0966	
Extinction coefficient	n/a	
Largest diff. peak and hole	0.378 and -0.312 e.$Å^{-3}$	

checkCIF/PLATON report

Structure factors have been supplied for datablock(s) miva556f1416

THIS REPORT IS FOR GUIDANCE ONLY. IF USED AS PART OF A REVIEW PROCEDURE FOR PUBLICATION, IT SHOULD NOT REPLACE THE EXPERTISE OF AN EXPERIENCED CRYSTALLOGRAPHIC REFEREE.

No syntax errors found. CIF dictionary Interpreting this report

Datablock: miva556f1416

Bond precision: C-C = 0.0012 A Wavelength=0.71073

Cell:	a=6.3438(4)	b=10.9125(7)	c=11.5912(7)
	alpha=72.476(5)	beta=89.445(5)	gamma=78.015(5)
Temperature:	100 K		

	Calculated	Reported
Volume	747.30(9)	747.30(8)
Space group	P -1	P -1
Hall group	-P 1	-P 1
Moiety formula	C18 H26 O2	C18 H26 O2
Sum formula	C18 H26 O2	C18 H26 O2
Mr	274.39	274.39
Dx,g cm-3	1.219	1.219
Z	2	2
Mu (mm-1)	0.077	0.077
F000	300.0	300.0
F000’	300.13	
h,k,lmax	10,17,19	10,17,18
Nref	7005	6600
Tmin,Tmax	0.988,0.997	0.873,0.973
Tmin’	0.979	

Correction method= # Reported T Limits: Tmin=0.873 Tmax=0.973 AbsCorr = MULTI-SCAN

Data completeness= 0.942 Theta(max)= 35.852

R(reflections)= 0.0411(3421) wR2(reflections)= 0.0966(6600)

S = 0.930 Npar= 184

The following ALERTS were generated. Each ALERT has the
format **test-name_ALERT_alert-type_alert-level**.
Click on the hyperlinks for more details of the test.

Alert level G

```
PLAT007_ALERT_5_G  Number of Unrefined Donor-H Atoms .............          2 Report
              H1    H2
PLAT154_ALERT_1_G  The  s.u.'s on the Cell Angles are Equal ..(Note)      0.005 Degree
PLAT910_ALERT_3_G  Missing # of FCF Reflection(s) Below Theta(Min).          3 Note
                0   1  0,   0  0  1,   0  1  1,
PLAT912_ALERT_4_G  Missing # of FCF Reflections Above STh/L=  0.600        394 Note
PLAT933_ALERT_2_G  Number of HKL-OMIT Records in Embedded .res File          1 Note
                3  -1  2,
PLAT969_ALERT_5_G The  'Henn et al.' R-Factor-gap value ..........       3.17 Note
              Predicted wR2: Based on SigI**2  3.04 or SHELX Weight 10.53
PLAT978_ALERT_2_G Number C-C Bonds with Positive Residual Density.          18 Info
```

0 **ALERT level A** = Most likely a serious problem - resolve or explain
0 **ALERT level B** = A potentially serious problem, consider carefully
0 **ALERT level C** = Check. Ensure it is not caused by an omission or oversight
7 **ALERT level G** = General information/check it is not something unexpected

1 ALERT type 1 CIF construction/syntax error, inconsistent or missing data
2 ALERT type 2 Indicator that the structure model may be wrong or deficient
1 ALERT type 3 Indicator that the structure quality may be low
1 ALERT type 4 Improvement, methodology, query or suggestion
2 ALERT type 5 Informative message, check

It is advisable to attempt to resolve as many as possible of the alerts in all categories. Often the minor
alerts point to easily fixed oversights, errors and omissions in your CIF or refinement strategy, so attention to these fine details can be worthwhile. In order to resolve some of the more serious problems it may be necessary to carry out additional measurements or structure refinements. However, the purpose of your study may justify the reported deviations and the more serious of these should normally be commented upon in the discussion or experimental section of a paper or in the "special_details" fields of the CIF. checkCIF was carefully designed to identify outliers and unusual parameters, but every test has its limitations and alerts that are not important in a particular case may
appear. Conversely, the absence of alerts does not guarantee there are no aspects of the results needing
attention. It is up to the individual to critically assess their own results and, if necessary, seek expert advice.

Publication of your CIF in IUCr journals

A basic structural check has been run on your CIF. These basic checks will be run on all CIFs submitted for publication in IUCr journals (*Acta Crystallographica*, *Journal of Applied Crystallography*, *Journal of Synchrotron Radiation*); however, if you intend to submit to *Acta Crystallographica Section C* or *E* or *IUCrData*, you should make sure that full publication checks are run on the final version of your CIF prior to submission.

Publication of your CIF in other journals

Please refer to the *Notes for Authors* of the relevant journal for any special instructions relating to CIF submission.

PLATON version of 06/01/2024; check.def file version of 05/01/2024

Datablock miva556f1416 - ellipsoid plot

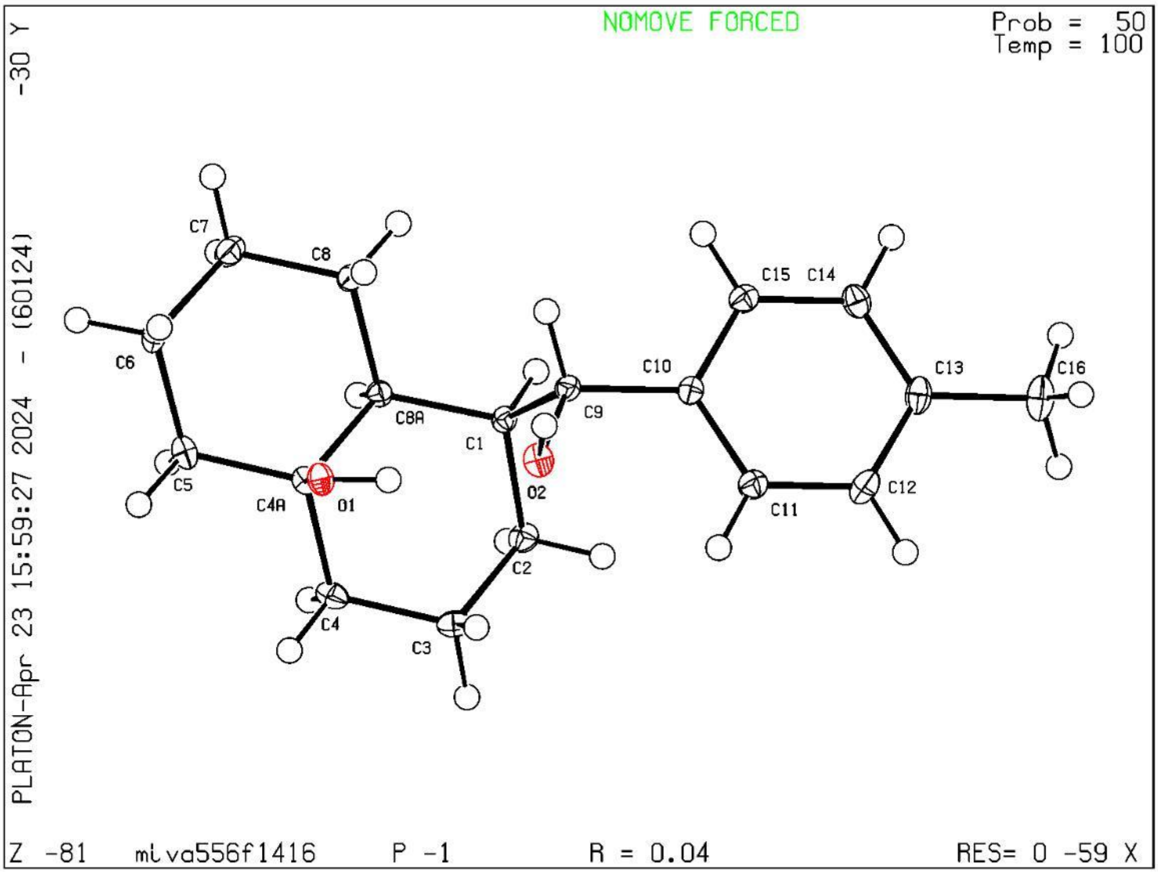

6. References

[13] G. Baddeley, E. Wrench, *J. Chem. Soc.* **1959**, 1324–1327.

[14] G. Baddeley, B. G. Heaton, J. W. Rasburn, *J. Chem. Soc.* **1960**, 4713–4719.

[15] M. S. Ahmad, G. Baddeley, *J. Chem. Soc.* **1961**, 4303–4306.

[16] G. Baddeley, B. G. Heaton, *J. Chem. Soc.* **1961**, 4306–4307.

[20] C. L. Lyall, M. Sato, M. Uosis-Martin, S. F. Asghar, M. D. Jones, I. H. Williams, S. E. Lewis, *J. Am. Chem. Soc.* **2014**, *136*, 13745–13753.

[21] M. Uosis-Martin, M. F. Mahon, M. Yevglevskis, S. E. Lewis, *Synlett* **2011**, *2011*, 2211–2213.

[25] G. Baddeley, B. G. Heaton, J. W. Rasburn, *J. Chem. Soc.* **1961**, 3835–3838.

[26] B. R. Brutiu, G. Iannelli, M. Riomet, D. Kaiser, N. Maulide, *Nature* **2024**, *626*, 92–97.

[35] G. Baddeley, B. G. Heaton, J. W. Rasburn, *J. Chem. Soc.* **1961**, 3828–3835.

[36] C. L. Lyall, M. Uosis-Martin, J. P. Lowe, M. F. Mahon, G. D. Pantoş, S. E. Lewis, *Org. Biomol. Chem.* **2013**, *11*, 1468–1475.

[37] G. R. Fulmer, A. J. M. Miller, N. H. Sherden, H. E. Gottlieb, A. Nudelman, B. M. Stoltz, J. E. Bercaw, K. I. Goldberg, *Organometallics* **2010**, *29*, 2176–2179.

[38] T.-S. Li, J.-T. Li, H.-Z. Li, *J. Chromatogr. A* **1995**, *715*, 372–375.

[39] J. Waser, B. Gaspar, H. Nambu, E. M. Carreira, *J. Am. Chem. Soc.* **2006**, *128*, 11693–11712.

[40] M. Farizyan, A. Mondal, S. Mal, F. Deufel, M. van Gemmeren, *J. Am. Chem. Soc*. **2021**, *143*, 16370–16376.

[41] K. Li, R. Li, Y. Cui, C. Liu, *Org. Biomol. Chem*. **2024**, *22*, 1693-1698

[42] Y.-X. Chen, J.-T. He, M.-C. Wu, Z.-L. Liu, K. Tang, P.-J. Xia, K. Chen, H.-Y. Xiang, X.-Q. Chen, H. Yang, *Org. Lett.* **2022**, *24*, 3920–3925.

[43] M. Steurer, C. Bolm, *J. Org. Chem.* **2010**, *75*, 3301–3310.

[44] P. Müller, E. Maîtrejean, *Collect. Czech. Chem. Commun.* **1999**, *64*, 1807–1826.

[45] D. D. Günbaş, F. Algı, T. Hökelek, W. H. Watson, M. Balci, *Tetrahedron* **2005**, *61*, 11177–11183.

[46] S. Lee, P. L. Fuchs, *J. Am. Chem. Soc.* **2002**, *124*, 13978–13979.

[47] H. O. House, J. H. C. Lee, D. VanDerveer, J. E. Wissinger, *J. Org. Chem.* **1983**, *48*, 5285–5288.

[48] D. P. Lubov, M. V. Shashkov, A. A. Nefedov, K. P. Bryliakov, *Org. Lett.* **2023**, *25*, 1359–1363.

[49] F. Saito, J. Becker, P. R. Schreiner, *J. Org. Chem*. **2020**, *85*, 4441–4447.

[50] G. M. Sheldrick, *Acta Crystallogr. A Found. Adv.* **2015**, *71*, 3–8.

[51] G. M. Sheldrick, *Acta Crystallogr. C Struct. Chem.* **2015**, *71*, 3–8.

[52] O. V. Dolomanov, L. J. Bourhis, R. J. Gildea, J. A. K. Howard, H. Puschmann, *J. Appl. Crystallogr.* **2009**, *42*, 339–341.

[53] C. B. Hübschle, G. M. Sheldrick, B. Dittrich, *J. Appl. Crystallogr.* **2011**, *44*, 1281–1284.
